# Synthesis and Structure of Mono-, Di-, and Trinuclear Fluorotriarylbismuthonium Cations

**DOI:** 10.1021/acs.organomet.2c00135

**Published:** 2022-05-09

**Authors:** Jennifer Kuziola, Marc Magre, Nils Nöthling, Josep Cornella

**Affiliations:** Max-Planck-Institut für Kohlenforschung, Kaiser-Wilhelm-Platz 1, Mülheim an der Ruhr 45470, Germany

## Abstract

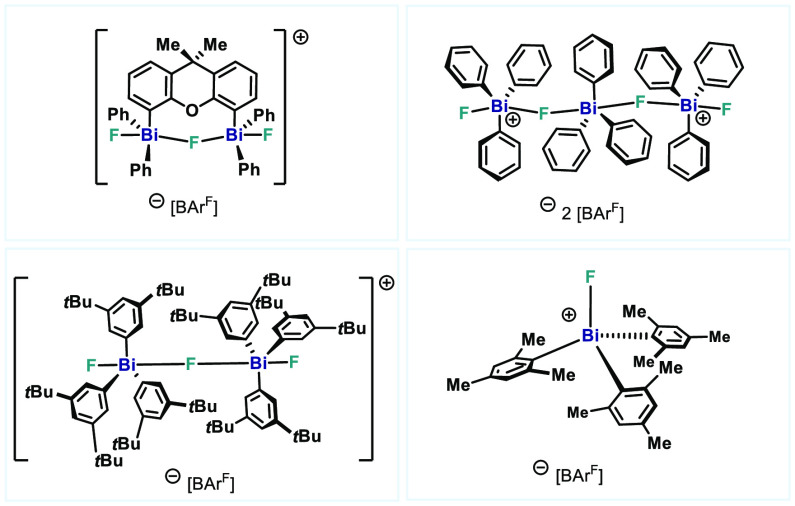

A series of cationic fluorotriarylbismuthonium salts bearing differently substituted aryl groups (Ar = 9,9-Me_2_-9H-xanthene, Ph, Mes, and 3,5-*t*Bu-C_6_H_3_) have been synthesized and characterized. While the presence of simple phenyl substituents around the Bi center results in a polymeric structure with three Bi centers in the repeating monomer, substituents at the *ortho*- and *meta*-positions lead to cationic mono- and dinuclear fluorobismuthonium complexes, respectively. Preparation of all compounds is accomplished by fluoride abstraction from the parent triaryl Bi(V) difluorides using NaBAr^F^ (BAr^F^^–^ = B[C_6_H_3_-3,5-(CF_3_)_2_]_4_^–^). Structural parameters were obtained via single crystal X-ray diffraction (XRD), and their behavior in solution was studied by NMR spectroscopy. Trinuclear and binuclear complexes are held together through one bridging fluoride (μ-F) between two Bi(V) centers. In contrast, the presence of Me groups in both *ortho*-positions of the aryl ring provides the adequate steric encumbrance to isolate a unique mononuclear nonstabilized fluorotriarylbismuthonium cation. This compound features a distorted tetrahedral geometry and is remarkably stable at room temperature both in solution (toluene, benzene and THF) and in the solid state.

## Introduction

**Figure 1:**
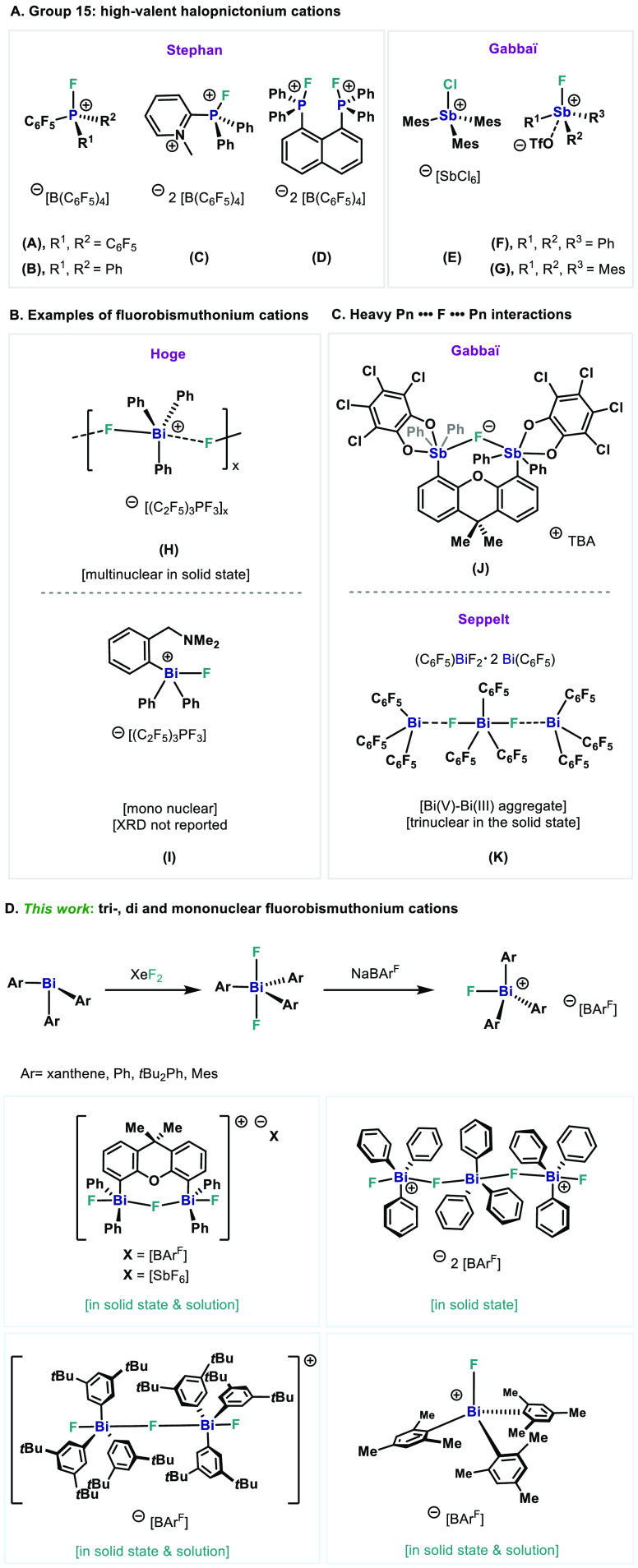
(A) Representative examples of high-valent halopnictonium cations. (B) Examples of fluorobismuthonium cations. (C) Examples of intra- and intermolecular heavy Pn···F···Pn interactions. (D) This work: Synthesis of high-valent fluorobismuthonium cations.

The development of cationic halogenated organopnictogen(V) compounds has witnessed a revival in recent years, due to their application as Lewis acids in catalysis.^1^ For example, the pioneering work from Stephan on cationic fluorotriarylphosphonium complexes (e.g., [(C_6_F_5_)_3_PF]^+^[(C_6_F_5_)_4_B]^−^) has demonstrated their usefulness as Lewis acid catalysts for organic synthesis, finding applications in a wide variety of contexts (Figure 1A, (A) and (B)).^2^ Related polycationic monodentate as well as bidentate fluorophosphonium cations have also been reported (Figure 1A, (C) and (D)) to find applications in hydrodefluorination,^3^ hydrosilylation of alkenes and alkynes,^4^ Friedel–Crafts dimerization of 1,1-diphenylethylene,^5^ dehydrocoupling,^6^ and deoxygenation of ketones.^7^ Besides the rich chemistry of the fluorophosphonium cations, heavier congeners of this family were also reported earlier with various halides; yet, their widespread applications are comparatively more limited. For Sb analogues, Sowerby reported in 1983 the synthesis of chlorotriphenylstibonium cation bearing hexachloroantimonate as anion.^8^ Subsequently, Gabbaï capitalized on their strong Lewis acidity, and applications of halotriarylstibonium cations as catalysts on Friedel–Crafts dimerizations ensued.^9^ In the same front, Gabbaï also isolated the chlorotrimesitylstibonium hexachloroantimonate salt (Figure 1A, (E)) and a fluorotriphenylstibonium cation (Figure 1A, (F)); interestingly, the latter contained an OTf anion in the inner coordination sphere of the antimony center. More recently, Adonin reported on the structure of [(2-MeO-5-BrC_6_H_4_)_3_SbI]^+^ with I_3_^–^ as counteranion.^10^ Heavier cationic halotriarylbismuthonium salts have been relatively less explored, and only few examples exist in the literature.^11,12^ In 1989, Klapötke reported a series of hexafluoroarsenates of group 15 elements of the type [Ph_3_PnI]^+^ [AsF_6_]^−^ (Pn = P, As, Sb, and Bi). Although these complexes were characterized by ^1^H NMR and IR spectroscopy,^11^ structural characterization by X-ray diffraction (XRD) eluded experimentalists. In this work, the cationic iodotriphenylbismuthonium structure was reported to be thermally unstable, rapidly leading to decomposition. More than 30 years later, Hoge reported the reaction of Ph_3_BiF_2_ with (C_2_F_5_)_3_PF_2_ resulting in a fluorotriphenylbismuthonium cation, which forms a polymeric structure in the solid state, as characterized by XRD analysis (Figure 1B, (H)).^12^ Such long chains feature bridging μ-F between Bi(V) centers, uniting the triarylbismuthonium cations in a polymeric linear disposition (F–Bi–F angles close to linearity [175.5(2)°]). It was also demonstrated that replacing the Ph group in Ph_3_BiF_2_ with 2-[(dimethylamino)methyl]phenyl leads to isolation of a monomeric fluorobismuthonium cation (Figure 1B, (I)), although its solid state structure was not provided. Regardless, compound H is a seminal example of how the Lewis acidity of the fluorotriarylbismuthonium(V) cation brings the lone pair of the F atom into the coordination sphere, leading to μ-F bridges.^12,13^ Other examples of fluoride-bridging compounds can be found in the xanthene-based distiborane complex reported by Gabbaï (Figure 1C, (J)).^14^ In subsequent work, the same research group demonstrated that changing the ligand scaffold to a 1,8-triptycenediyl backbone bearing a methine group supported the fluoride chelation by forming C–H···F hydrogen bonding.^15^ Bi–F···Bi–F interactions can be also found in neutral organobismuth complexes. Studies on the synthesis of (C_6_F_5_)_3_BiF_2_ by Seppelt resulted in a solid state structure of (C_6_F_5_)_3_BiF_2_·2Bi(C_6_F_5_)_3_, formed by association of a Bi(V) unit with two trigonal-pyramidal Bi(III) molecules (Figure 1C, (K)).^16^ This situation highlights the high Lewis acidity of Bi(III) bearing electron-withdrawing groups, to the point that allows coordination of the F at the σ* C–Bi bonds. Another example of such neutral halogen···Bi interactions can be found in the study reported by Auer and Mehring in Bi(III) complexes.^17^ In there, intermolecular donor–acceptor Bi–Cl···Bi interactions of Bi–Cl were observed for Ar_2_BiCl (Ar = 3,5-*t*Bu_2_-C_6_H_3_).

Our group has recently been interested in the chemistry of organobismuth compounds, from the point of view of both catalysis^18^ and structure.^19^ Due to the limited number of triarylhalobismuthonium cations and the even more limited structural information, we decided to fill this gap in the area by preparing a family of novel cationic fluorotriarylbismuthonium salts, whose structures were analyzed both in solution (NMR) and in solid state (XRD). Variation of the substituents on the aryl ring resulted in the isolation of tri-, di-, or mononuclear fluorobismuthonium species. This systematic study culminated with the preparation and isolation of a genuine fluorotriarylbismuthonium cation, without hypervalent coordination or additional stabilization (Figure 1D).

## Results and Discussion

**Scheme 1:**
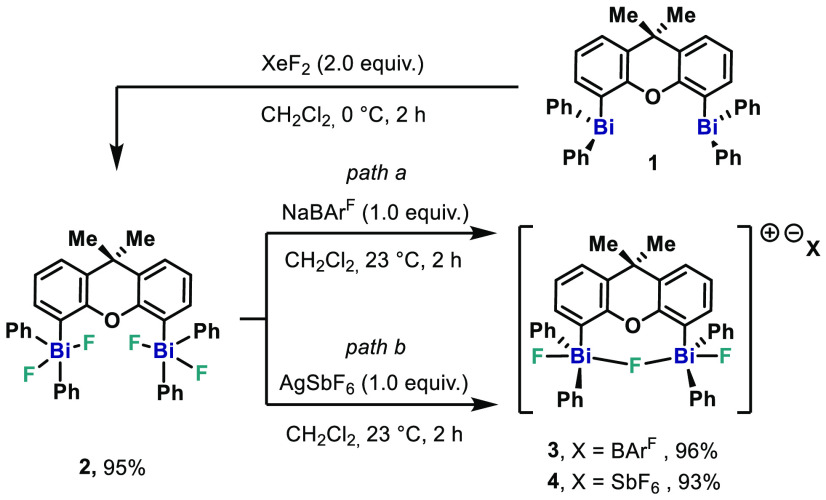
Synthesis of Cationic Fluorine-Bridged XantBis(V) **3** and **4** with NaBAr^F^ and AgSbF_6_

**Figure 2:**
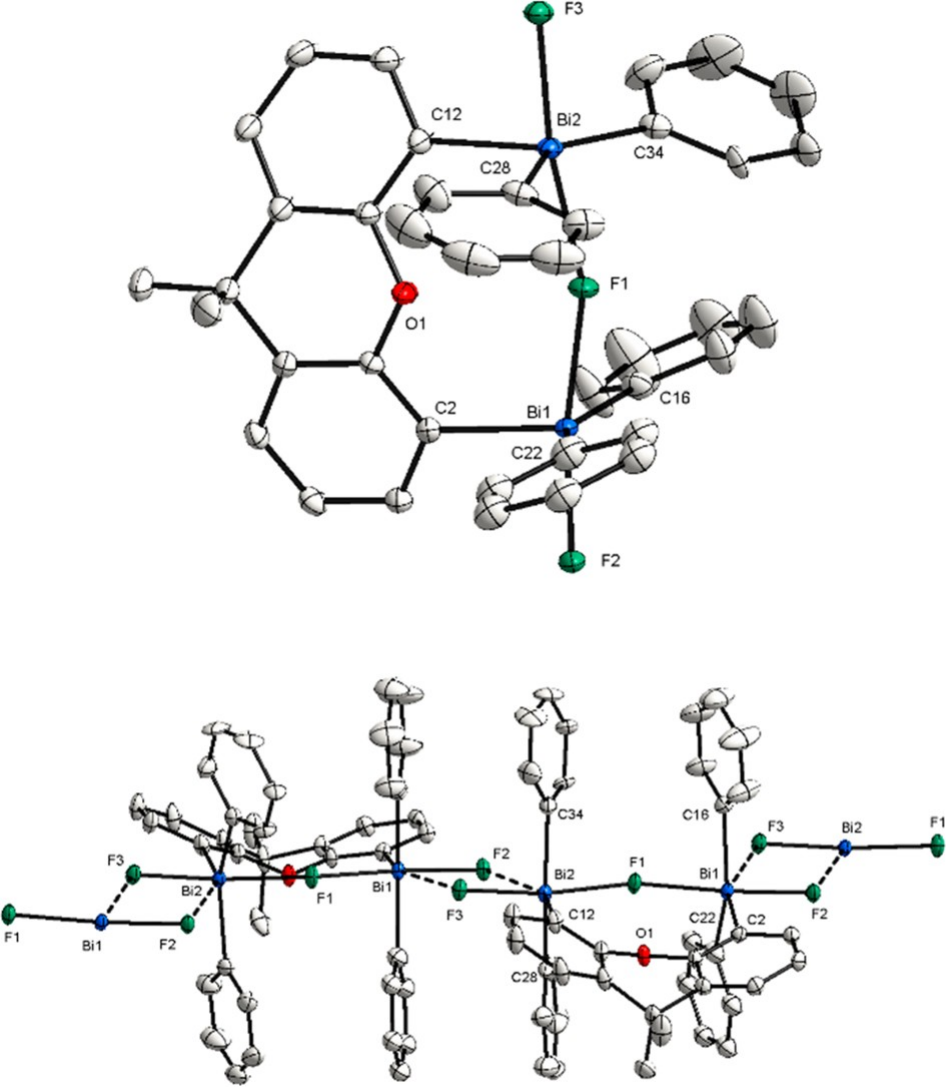
(Top) XRD structure of **3**. Ellipsoids are drawn at the 50% probability level, and H atoms, [BAr^F^]^−^, and disordered parts are omitted for clarity. Selected bond lengths (Å) and angles (°): Bi1–F1, 2.2699(15); Bi1–F2, 2.1134(15); Bi2–F1, 2.2648(15); Bi2–F3, 2.1212(15); C16–Bi1–C22, 147.70(9)°; C34–Bi2–C28, 143.14(11); Bi2–F1–Bi1, 159.68(8). (Bottom) XRD structure of the repeating monomeric unit **3**. Intermolecular interactions between single units are shown with dashed lines. Selected bond lengths (Å) and angles (°): Bi1···F3, 2.6526(15); Bi2···F2, 2.7132(15); F1–Bi1···F3, 102.62(5); Bi1–F2···Bi2, 111.86(6); F2–Bi1···F3, 67.71(5); F3–Bi2···F2, 66.38(5); F1–Bi2···F2, 108.08(5).

We have previously reported the synthesis of dibismuthanes such as XantBis (4,5-bis(diphenylbismuthino)-9,9-dimethylxanthene) (**1**) and its pentavalent tetrachlorinated bismuth(V).^19^ In order to investigate the behavior of the corresponding fluorinated analog, oxidation of XantBis (**1**) was attempted using XeF_2_. Gratifyingly, compound **2** was formed exclusively and could be isolated in 95% yield (Scheme 1). Although single crystals were not formed, and therefore the structure remains unknown, characterization of **2** was conducted by NMR and HRMS. When **2** was treated with 1.0 or 2.0 equiv of NaBAr^F^ (BAr^F–^ = B[C_6_H_3_-3,5-(CF_3_)_2_]_4_^–^), one of the fluorine atoms was abstracted, leading to the cationic dinuclear bismuthonium salt **3** in 96% isolated yield (Scheme 1, *path a*). Interestingly, despite the use of 2 equiv of NaBAr^F^, only one fluorine is removed to form **2**, suggesting a high stability of the corresponding salt. Colorless single crystals of compound **3** suitable for XRD analysis were obtained by layering a concentrated dichloromethane solution of **3** with hexane at +5 °C (Figure 2, top).

The XRD analysis reveals that complex **3** involves intermolecular interactions leading to the formation of a one-dimensional cationic coordination polymer (Figure 2, bottom). This coordination network of the complex is based on the intermolecular F–Bi···F–Bi interactions, which can be observed in the Bi1···F3 distances of 2.652(15) Å and Bi2···F2 of 2.713(15) Å. Furthermore, it can be seen that the C16–Bi1–C22 (147.70(9)°) and C28–Bi2–C34 (143.14(11)°) angles are widened compared to neutral Ph_3_BiF_2_,^20^ allowing a closer interaction between the terminal fluorine and the bismuth atoms. Incorporating the intermolecular interaction, the Bi atoms exhibit a distorted octahedral geometry (for further details see the Supporting Information (SI), X-ray Single Crystals Analysis section). However, when looking at the single units of this polymer (Figure 2, top), each Bi atom adopts a slightly distorted trigonal-bipyramidal geometry with two fluoride ligands in the apical position and the aromatic rings in the equatorial position. More importantly, complex **3** features a fluorine atom bridging the two Bi atoms, reminiscent of complex **J** reported by Gabbaï (Figure 1C).^14^ The Bi–C distances are in the range of those observed for neutral Ph_3_BiF_2_.^20^ Terminal fluorine atoms exhibit a slightly shorter bond length [Bi1–F2, 2.1134(15) Å; Bi2–F3, 2.1212(15) Å] compared to the distance between the bridging fluorine and Bi atoms [Bi1–F1, 2.2699(15) Å; Bi2–F1, 2.2648(15) Å]. Based on the close bond lengths of Bi1–F1 and Bi2–F1, the positive charge is likely to be shared by both bismuth centers. A deviation from the linearity can be observed for the Bi1–F1–Bi2 angle of 159.68(8)°. The bridging and terminal fluorides can be clearly observed in the ^19^F NMR spectrum as a triplet at −105.36 ppm and a doublet at −156.04 ppm with ^2^*J*_*F–F*_ = 98.2 Hz and ^2^*J*_*F–F*_ = 98.0 Hz coupling constants, respectively.

**Figure 3:**
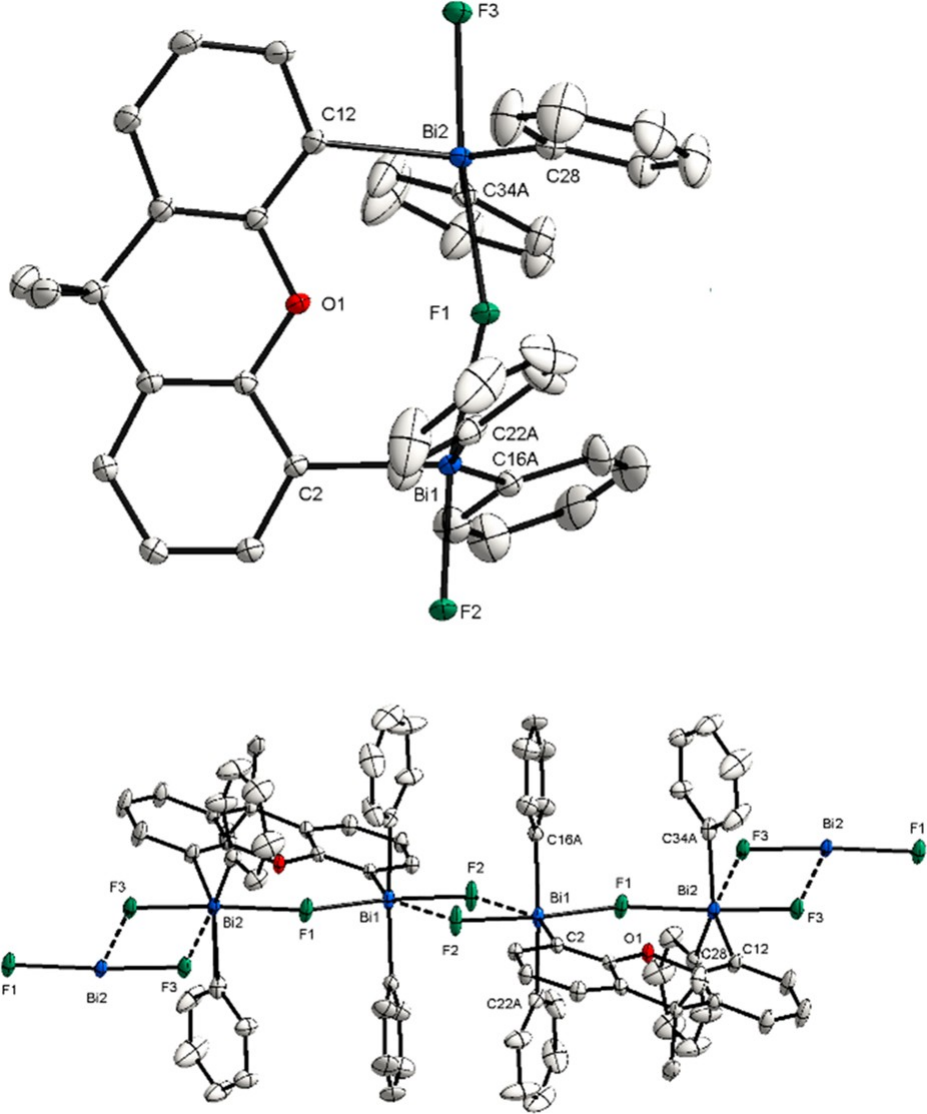
(Top) XRD structure of **4**. Ellipsoids are drawn at the 50% probability level, and H atoms, [SbF_6_]^−^, and disordered parts are omitted for clarity. Selected bond lengths (Å) and angles (°): Bi1–F1, 2.2715(14); Bi1–F2, 2.1186(14); Bi2–F1, 2.2996(14); Bi2–F3, 2.1158(14); F2–Bi1–F1, 169.68(6); C16A–Bi1–C22A, 148.2(2); F3–Bi2–F1, 172.48(5); C34A–Bi2–C28, 141.1(2); Bi1–F1–Bi2, 158.12(7). (Bottom) XRD structure of the repeating monomeric unit **4**. Intermolecular interactions between single units are shown with dashed bonds. Selected bond lengths (Å) and angles (°): Bi1···F2, 2.6735(15); Bi2···F3, 2.6613(14); F2–Bi1···F2, 66.76(6); F1–Bi1···F2, 103.16(5); F1–Bi2···F3, 108.06(5); F3–Bi2···F3, 64.60(6).

In order to study the influence of the anion, we carried out the fluoride abstraction with silver hexafluoroantimonate (AgSbF_6_) (Scheme 1, *path b*), leading to a 93% yield of **4**. Colorless single crystals suitable for X-ray diffraction were obtained upon diffusion of hexane into a dichloromethane solution of **4**. This compound exhibits a similar polymeric structure as the cationic complex **3**, albeit with some interesting differences in the single units (Figure 3). While the Bi–C and Bi–F distances and C–Bi–C, F–Bi–F, and Bi1–F1–Bi2 angles are practically identical, differences in the geometry of the xanthene backbone are noticeable. In compound **3**, the xanthene backbone adopts a strong bent geometry (Ar–C–Ar, 135.89°), while the three rings in structure **4** are disposed in a nearly planar geometry (Ar–C–Ar, 165.63°).

**Scheme 2:**
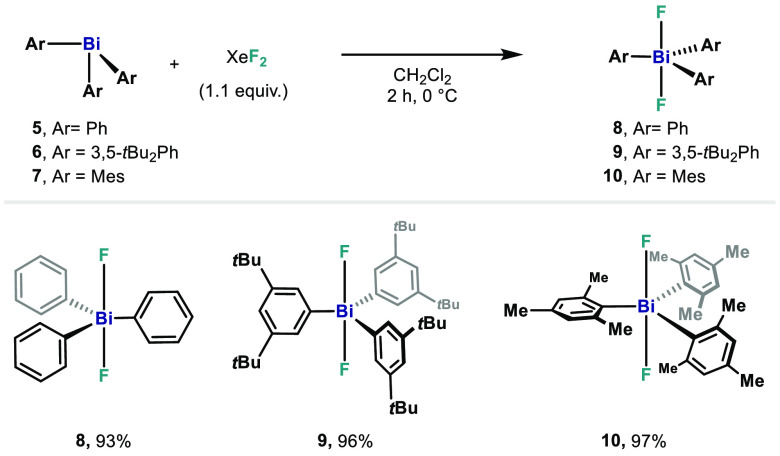
Synthesis of Triarylbismuthdifluorides **8-10**

**Figure 4:**
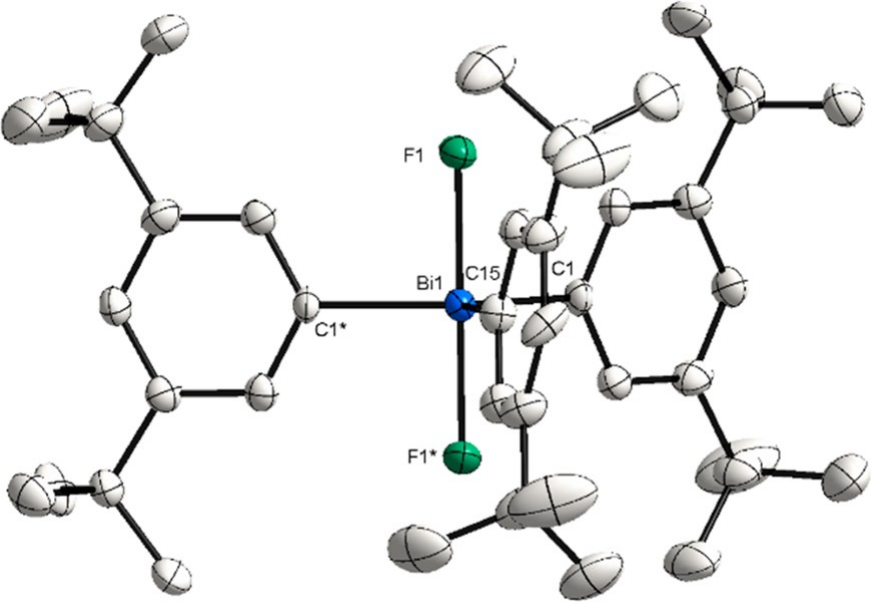
XRD structure of **9**. Ellipsoids are drawn at the 50% probability level, and H atoms and disordered parts are omitted for clarity. Selected bond lengths (Å) and angles (°): Bi1–F1, 2.129(3); Bi1–C1, 2.209(5); Bi1–C15, 2.192(8); F1*–Bi1–F1, 179.1(2).

**Figure 5:**
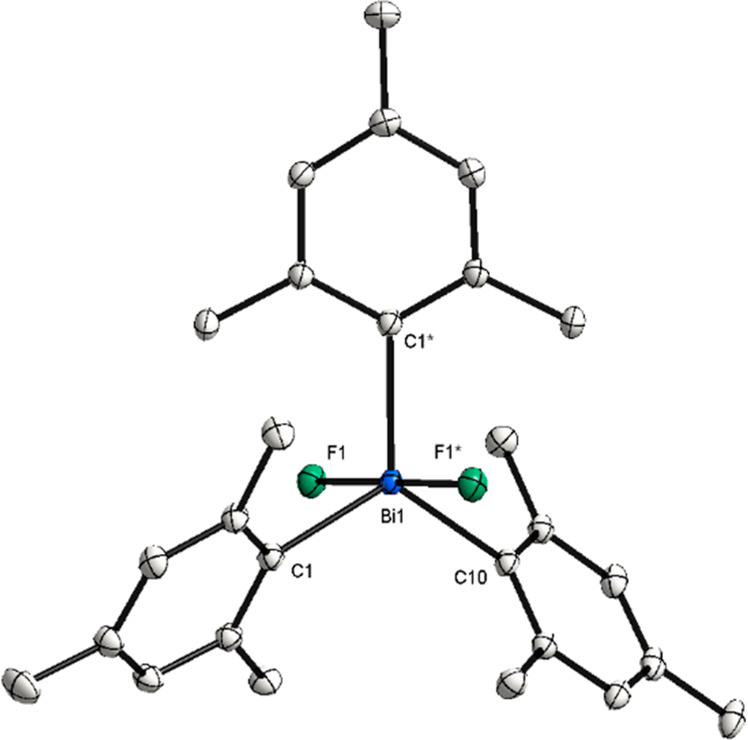
XRD structure of **10**. Ellipsoids are drawn at the 50% probability level, and H atoms are omitted for clarity. Selected bond lengths (Å) and angles (°): Bi1–F1, 2.1222(10); Bi1–C1, 2.2144(14); Bi1–C10, 2.2141(18); F1–Bi1–F1*, 179.00(5).

In both **3** and **4**, the F abstraction using NaBAr^F^ and AgSbF_6_ has led to F-bridged intramolecular connections. However, with the precedents from Seppelt and Hoge,^12,16^ we hypothesized that such behavior could also be observed in an intermolecular fashion. In order to have a broader spectrum beyond the simple unsubstituted difluorotriphenylbismuth (**8**), aryls substituted with *t*Bu- and Me-substituents have also been synthesized. Based on literature described protocols for the syntheses of complexes **6** and **7**,^17,21^ the respective bismuth(III) species were obtained as colorless solids in 60% and 45% yield, respectively (Scheme 2). At this point, the corresponding triarylbismuth(V) difluoride analogs were obtained using XeF_2_, obtaining high yields of **8**–**10** (Scheme 2). Whereas **8** and **10** are known, **9** represents a novel triarylbismuthdifluoride compound. Colorless single crystals suitable for X-ray crystallography of **9** and **10** were isolated upon crystallization from diffusion of hexane into a solution of the complex in CH_2_Cl_2_ at ambient temperature (Figures 4 and 5).

The Bi center in complexes **8**–**10** adopts a trigonal bipyramidal geometry with fluorides in the apical positions. In the case of novel complex **9**, the distances of Bi–F are similar to those in **8**:^20^ [Bi1–F1, 2.129(3) Å] (Figure 5). As a result of steric hindrance and C–(*ortho*)H···F hydrogen interactions, the *t*Bu groups in the *meta*-position are in the plane delineated by the quasi-linear F1–Bi–F1 axis [F1–Bi–F1*, 179.1(2)°]. The Bi–C bonds of Bi complex **9** are similar to **8** and only slightly shorter to compared to **6** [Bi1–C1, 2.27(2) Å].^17^ Complex **9** shows a singlet at −160.92 ppm in the ^19^F NMR spectrum, similar to that of **8**. This suggests that in **9**, the *t*Bu groups exert no significant electronic influence on the F atoms.

Whereas attempts to synthesize triarylbismuth(III) bearing *t*Bu groups at the *ortho*-position failed, a decrease in steric demand was envisaged. Hence, trimesitylbismuthine (**7**),^21^ bearing methyl groups in the *ortho*- and *para*-positions, was synthesized instead (Figure 4). Oxidation of **7** with XeF_2_ smoothly afforded complex **10** in 97% yield, whose structure was confirmed by XRD analysis. The molecular structure exhibits the classic trigonal bipyramidal geometry around the bismuth atom with nearly identical structural parameters as those for tris(3,5-di-*tert*-butylphenyl)bismuthdifluoride (**9**). The F1–Bi–F1* angle of 179.00(5)° deviates slightly from linearity. Comparing trimesitylbismuthdifluoride with its lighter isostructural analogs,^9,22^ it can be observed that all complexes crystallize in the monoclinic space group *C2*/*c*. However, a slight trend can be observed. The solid state structure of compound **10** reveals a Bi–F bond distance of 2.1222(10) Å, which is longer than the reported Sb and P analogues (Sb–F, 1.982(1) Å;^9^ P–F, 1.673(2) Å).^22^ The ^19^F NMR spectrum of **10** shows a singlet at a higher chemical shift (−100.41 ppm) compared to **8** and **9**, presumably due to the influence of the methyl group in the *ortho*-position. Considering the (*ortho*-Me)H···F distance (2.329, 2.338, and 2.375 Å) of complex **10**, it may be assumed that the interaction between H and F is contributing to the increase in the chemical shift compared to those of complexes **8** [(Ph)*ortho*-H···F1, 2.418 Å]^17^ and **9** [(Ar)*ortho*-H···F1, 2.405 Å; (*t*Bu)H···F1, 3.981 Å], in which the H···F distances are longer. Interestingly, whereas the same chemical shift can also be observed for the fluorine atoms in the lighter Mes_3_SbF_2_,^9^ the signal for Mes_3_PF_2_ appears at −25.7 ppm.^22^

**Scheme 3:**
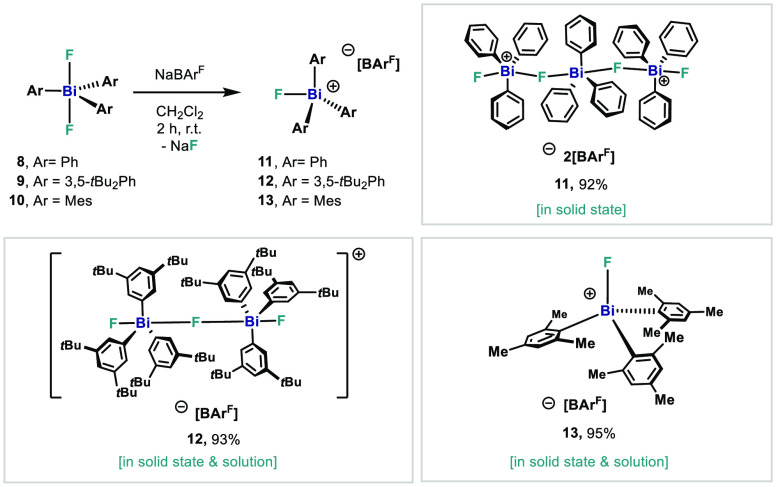
Synthesis of Fluorobismuthonium Cations **11–13**

Treating Ph_3_BiF_2_ with 1.0 or 0.5 equiv of NaBAr^F^ in CH_2_Cl_2_ at 25 °C resulted in clear ^19^F NMR spectra of the crude: a triplet at −123.01 ppm (^2^*J*_F–F_ = 91.0 Hz) and a doublet at −162.01 ppm (^2^*J*_F–F_ = 88.9 Hz) in addition to the [BAr^F^]^−^ signal at −62.87 ppm (Scheme 3).^23^

**Figure 6:**
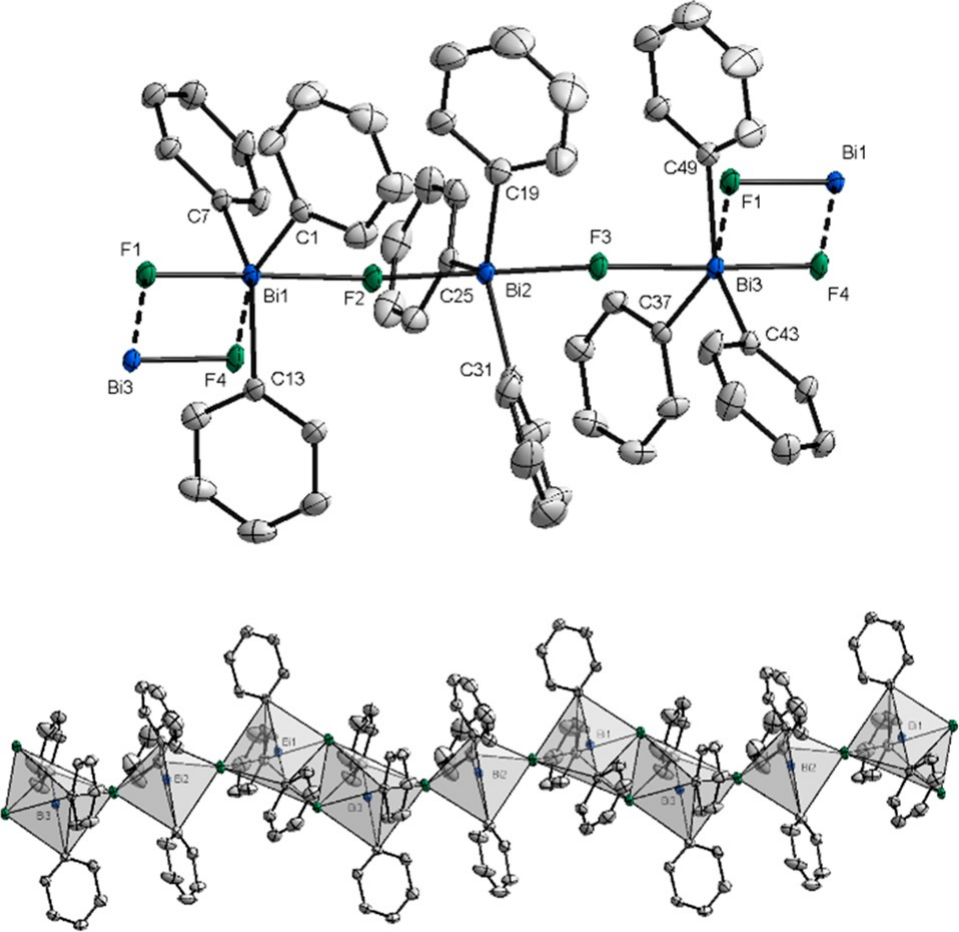
(Top) XRD structure of **11**. Ellipsoids are drawn at the 50% probability level, and H atoms and the counteranion BAr^F^ are omitted for clarity. Intermolecular interactions between single trinuclear units are shown with dashed bonds. Selected bond lengths (Å) and angles (°): Bi1–F1, 2.078(2); Bi1–F2, 2.376(2); Bi1···F4, 2.796 (2); Bi2–F2, 2.187(2); Bi2–F3, 2.173(2); Bi3–F3, 2.329(19); Bi3–F4, 2.068(2); Bi3···F1, 2.855(2); F1–Bi1–F2, 178.50(8); F3–Bi2–F2, 179.44(9); F4–Bi3–F3, 177.35(8); C37–Bi3–C43, 106.62(13); C49–Bi3–C43, 115.18(14); Bi2–F3–Bi3, 171.72(11); Bi2–F2–Bi1, 175.11(12). (Bottom) Polyhedral representation of the Bi atoms in complex **11**. H atoms, disordered parts, and BAr^F^ are omitted for clarity.

The chemical shifts and the pattern observed resembled those obtained for complexes **3** and **4**, suggesting the possibility of having an intermolecular dinuclear bismuth cation, bridged in an intermolecular fashion through one F atom (*vide infra*). Crystallization of the compound from the reaction mixture resulted in the isolation of a 1D coordination polymer, which is assembled via intermolecular Bi···F interactions of single cationic trinuclear bismuth units such as complex **11** (Figure 6, top).

This monomeric unit represents a dicationic bismuthonium salt with three BiPh_3_ moieties united through two consecutive biconnective fluoride ligands and two BAr^F^ moieties as counteranions. The central bismuth atom in **11** adopts a trigonal-bipyramidal geometry with two fluoride ligands in the apical positions and the phenyl rings in the equatorial. The Bi–F bonds in **11** show lengths of Bi2–F2, 2.187(2) Å, and Bi2–F3, 2.173(2) Å, which are shorter compared to the polymeric structure reported by Hoge et al. [Bi–F1, 2.267(6) Å] and to Ph_3_BiF_2_ [Bi–F, 2.59 Å].^12,20^ With respect to the central Ph_3_BiF_2_ subunit, the terminal Bi–F bonds are significantly shorter with Bi2–F2 of 2.078(2) Å and Bi1–F1 of 2.068(2) Å, respectively. Such distances manifest that the dicationic character of **11** is located at the terminal Ph_3_BiF moieties, while the central Ph_3_BiF_2_ unit remains rather neutral. The cationic FBiPh_3_ moieties are also evident by the longer bond lengths between the terminal Bi atoms and the bridging fluorine atoms [Bi1–F2, 2.376(2) Å; Bi3–F3, 2.329(19) Å]. The F2–Bi2–F3 angle of 179.44(9)° of the central Ph_3_BiF_2_ moiety deviates slightly from linearity, whereas the F1–Bi1–F2 angle of 178.50(8)° and F3–Bi3–F4 of 177.35(8)° of the Ph_3_BiF moieities are slightly more distorted. The angles of the bismuth atoms bridged by fluorine can be described by Bi1–F2–Bi2 of 175.11(12)° and Bi2–F3–Bi3 of 171.72(11)° as slightly bent. In the analysis of the entire polymer (Figure 6, bottom), the terminal bismuth atoms Bi1 and Bi2 adopt a distorted octahedral geometry, while the central Bi adopts a trigonal-bipyramidal geometry as depicted in Figure 6. Complex **11** shows similar features in C–Bi–C angles and in intermolecular Bi–F distances in comparison with the polymeric structures **3** and **4**. However, the intermolecular Bi···F bond distances between the single units are longer [Bi1–F4, 2.796(2) Å; Bi3–F1, 2.855(2) Å] than in complexes **3** and **4**, indicating that the intermolecular interactions are weaker.

**Scheme 4:**
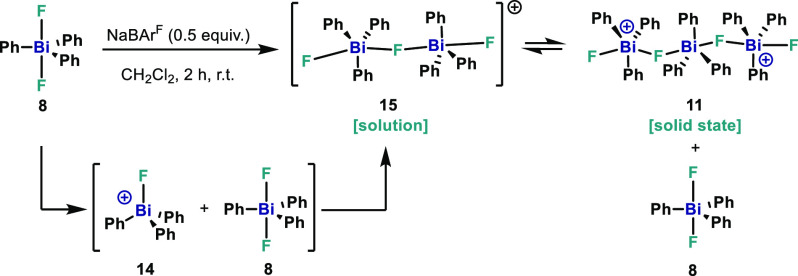
Proposed Behavior of Complex **11** in Solution and Solid State

Reaction of **8** using either 0.5 or 1 equiv of NaBAr^F^ led to the isolation of the same compound **11** (Scheme 3). These results evidence that the same cationic species are formed in both solution and solid state regardless of the amount of F scavenger, and higher amounts of halide scavenger do not lead to further fluoride abstraction. As depicted in Scheme 4, we hypothesized that after abstraction of 0.5 equiv of F atoms from neutral **8**, half of the complex is converted into cationic species **14**, which engages with the remaining neutral **8**, resulting in the formation of the dinuclear cationic **15**. Compound **15** was the sole product identified in the CD_2_Cl_2_ solution, as confirmed by NMR and HRMS. However, during crystallization, dinuclear monocation **15** undergoes equilibration, leading to the formation of **11** and **8**. Differently than Hoge’s polymeric structure (Figure 1B, (H)), formation of **11** represents a distinct structural outcome for a seemingly similar fluorotriphenylbismuthonium cation by simply modifying the solvent and the anion.

When the F abstraction is carried out with complex **9**, a completely different behavior is observed. Treatment of compound **9** with either 1.0 or 0.5 equiv of NaBAr^F^ in CH_2_Cl_2_ resulted in the formation of cationic dimer **12** in 93% isolated yield (Scheme 3). Here, the structure in the solid state coincides with the structure in solution, and no dicationic trinuclear structures were observed. Furthermore, the solid state analysis indicates no intermolecular F–Bi···F–Bi interactions between single units, resulting in a discrete dimeric structure in solid state. Presumably, the presence of the *t*Bu groups in the *meta*-position prevents their formation, stabilizing the dication in solid state through London dispersion forces (LDFs).^24^ The symmetric F–Bi–F–Bi–F arrangement is evident by its characteristic signals in ^19^F NMR. The two terminal fluorine ligands appear as a sharp doublet signal at −162.06 ppm with a ^2^*J*_*F–F*_ = 102.9 Hz, similar to that obtained for **3**, **4**, and **11**. The bridging fluorine atom can be observed as a triplet peak at −152.78 ppm in the ^19^F NMR.

**Figure 7:**
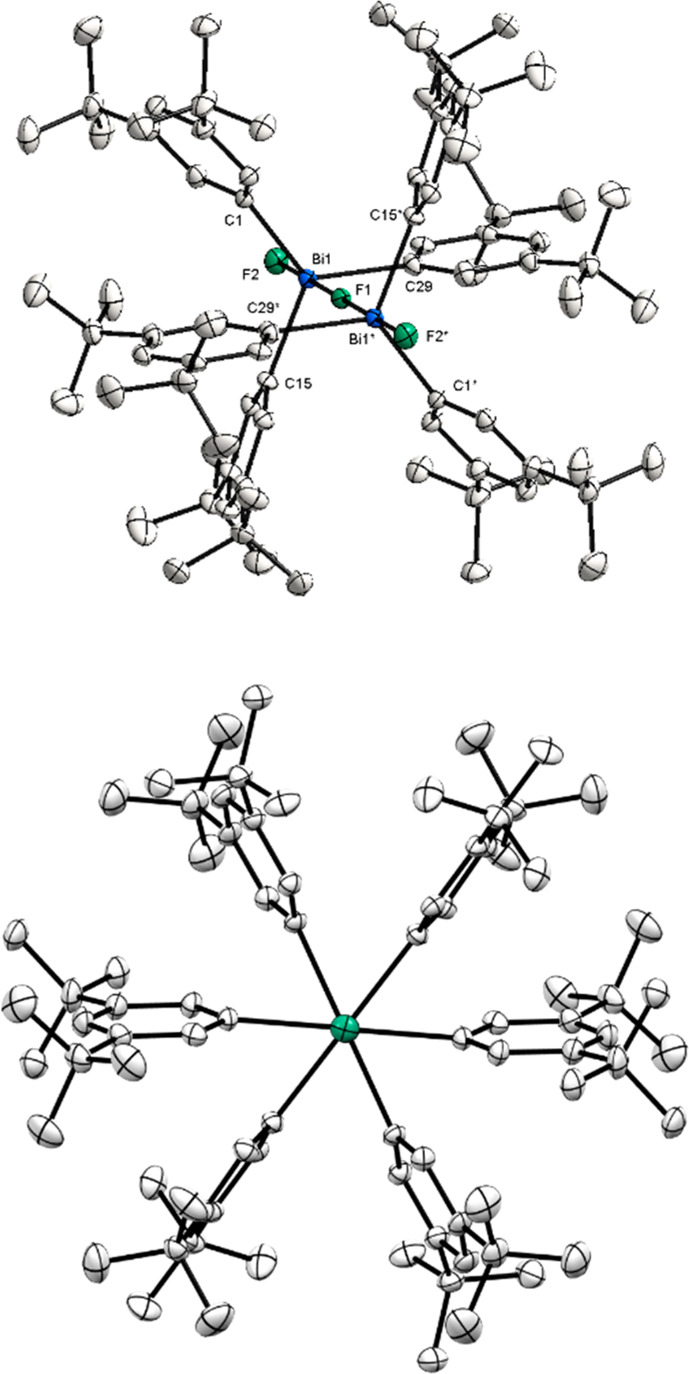
(Top) XRD structure of **12**. Ellipsoids are drawn at the 50% probability level, and H atoms and the counteranion BAr^F^ are omitted for clarity. Selected bond lengths (Å) and angels (°): Bi1–F1, 2.2820(3); Bi1–F2, 2.0565(19); F2–Bi1–F1, 179.31(5); Bi1–F1–Bi1*, 180.00. (Bottom) View of the structure along the F–Bi–F–Bi–F axis.

Solid state analysis of **12** reveals that each Bi center adopts a trigonal-bipyramidal geometry with two fluorides *trans* to each other in apical positions and aromatic rings in equatorial positions (Figure 7 , top). As already identified by NMR, fluoride ligand bridging two Bi centers is observed. The Bi1–F1–B1* angle is 180.00°. Analogously, the bond length between the terminal fluoride ligands and the Bi centers is shorter in comparison to the bridging fluorine atom. Based on the bond length of Bi1–F1 [2.282(3) Å], the positive charge is distributed to both bismuth atoms, in a similar manner as in **3** and **4**. By locating *t*Bu substituents in the *meta*-position, a perpendicular orientation of the aryl groups to the F–Bi–F axis can be observed. This arrangement has already been seen in the neutral parent compound **9**. As a result of the dimerization and the *t*Bu groups, complex **12** appears in a staggered conformation along the F–Bi–F axis, unlike the unsubstituted complex **11**. Owing to the steric repulsion of the *t*Bu groups, this conformation forms a snowflake-like structure (Figure 7, bottom).

**Figure 8:**
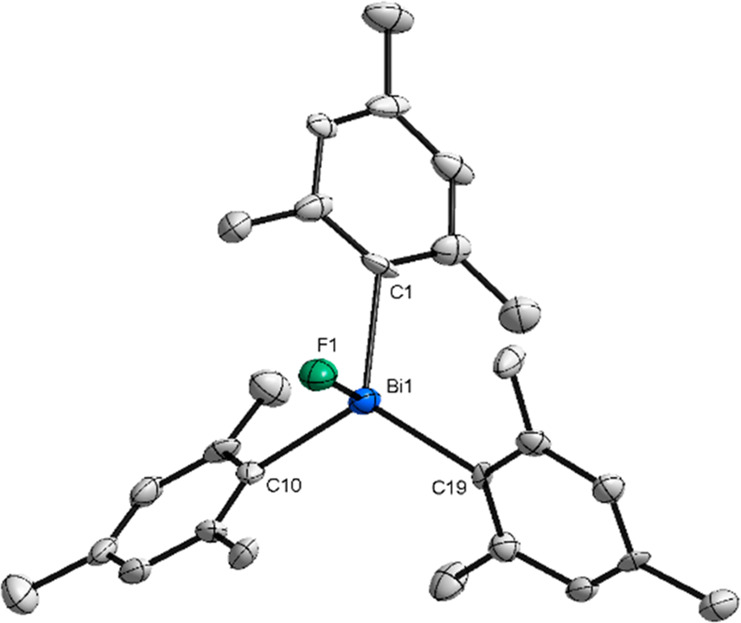
XRD structure of **13**. Ellipsoids are drawn at the 50% probability level, and H atoms and the counteranion BAr^F^ are omitted for clarity. Selected bond lengths (Å) and angels (°): Bi1–F1, 2.009(5); Bi1–C1, 2.222(8); Bi1–C10, 2.223(8); Bi1–C19, 2.213(7); Bi1–F1, 2.009(5).

Up until now, F abstraction on monomeric triarylbismuthdifluorides has led to isolation of di- or trinuclear cationic species. Based on the precedent from Gabbaï on the isolation of [Mes_3_SbCl]^+^[SbCl_6_]^−^ as a monomeric species,^9^ we speculated that probably, the *ortho*-Me would exert a similar effect on the Bi, enabling the isolation of a monomeric fluorotriarylbismuthonium cation. To this end, we subjected Mes_3_BiF_2_ (**10**) to 1.0 equiv of NaBAr^F^. This time, monomeric complex **13** was isolated in 92% as an off white solid (Scheme 3). The structure of **13** was characterized by both NMR and single crystal X-ray diffraction (Figure 8).

The XRD analysis reveals that the Bi atom adopts a pseudotetrahedral geometry. The Bi–F bond distance of 2.009(5) Å is consistent with the distances between the terminal fluorine atoms and the bismuth center of compounds **11** and **12** [Bi1–F1, 2.078(2) Å; Bi1–F2, 2.056(2) Å], which highlights the cationic nature of this structure. While the Bi–F bond lengths are only slightly shorter compared to the neutral parent compound **10** [Bi1–F1, 2.122(3) Å; Bi1–F1*, 2.122(2) Å], an increase in the (*ortho*-Me)H···F bond distances can be noticed [(*ortho*-Me)H···F1, 2.400 Å; (*ortho*-Me)H···F1, 2.549 Å; (*ortho*-Me)H···F1, 2.634 Å], considering the *ortho*-Me substituents, which are oriented parallel to the fluorine. As a result of the elongation and the reduced H···F electron interaction, a singlet signal can be observed at −177.11 ppm in CDCl_3_, CD_2_Cl_2_, and toluene-*d*_*8*_. Interestingly, by using CD_3_CN as solvent, the singlet appears at −148 ppm, probably due to possible coordination of the solvent. However, the singlet signal in both ^19^F NMR and XRD analyses indicates that no polymeric chain or further interactions with another Bi complex are formed (see the SI for details, X-ray Single Crystal Analysis section). The use of Me groups in the *ortho*- and *para*-positions gives the structure a high steric demand compared to complexes **11** and **12**. In contrast to compound **12**, in which the aryl groups bearing *t*Bu substituents in the *meta*-position are arranged perpendicular to the F–Bi–F axis, the mesityl ligands exhibit an oblique orientation to the Bi–F bond. This described propeller-like orientation leads to the prevention of further polymerization. As already indicated, the peculiarity of this structure results from its steric congestion, unlike base stabilization. However, comparison of the monomeric cationic Bi species **13** with its lighter analogs, such as [Mes_3_PF][FB(C_6_F_5_)_3_]^25^ and [Mes_3_SbF][OTf],^9^ reveals an increase in the Pn–F bond length as the pnictogen becomes heavier [P–F, 1.561(1) Å; Sb–F, 1.948(7) Å]. An interesting observation to note is that the Bi–F bond is only slightly longer than the Sb–F bond [Bi1–F1, 2.009(5) Å; Sb–F, 1.947(2) Å]. However, this result must be treated with caution since OTf as anion is weakly coordinated to the antimony center and consequently noninnocent. Unfortunately, a comparison cannot be made with the arsenic analog, as this structure is not yet known in the literature. Furthermore, the monomeric Bi(V) cation **13** cannot be compared with the monomeric fluorobismuthonium salt reported by Hoge et al.,^12^ since no single crystal structure has been reported. However, when comparing complex **13** with the single unit of the polymeric bismuthonium cation reported by Hoge, differences in the Bi–C and Bi–F bond lengths can be observed. Whereas the Bi–C bond distances of the polymeric structure by Hoge are shorter [Bi–C, 2.178(3) Å and 2.172(0) Å], the Bi–F bond lengths are elongated [Bi–F, 2.267(3) Å] and even longer than the Bi–F distances in the triarylbismuthdifluoride complexes **9** and **10** (for further details see the SI, X-ray Single Crystal Analysis section).

## Conclusion

In summary, a series of mono-, di-, and trinuclear fluorobismuthonium cations **11**–**13** were synthesized and fully characterized. In the trinuclear and dinuclear complexes, μ-F bridges are observed connecting two different Bi(V) atoms and holding the complex together in the solid state. In the case of the polymeric structure **13**, consisting of trinuclear bismuth units, we observed a dinuclear species in solution. By introducing steric *t*Bu groups in the *meta*-position, trimerization could be prevented and a snowflake-like dinuclear structure was obtained, which exhibits the same pattern both in solution and in the solid state. By further fine-tuning the ligand scaffold, the presence of Me groups in both *ortho*-positions of the aryl led to the isolation of a mononuclear fluorotriarylbismuthonium cation that is not stabilized by the introduction of additional electronic effects. With this unique mononuclear fluorotrimesitylbismuthonium cation, we provide a fundamental contribution to the spectrum of halopnictonium cations. Overall, these results represent a step forward in understanding the subtle differences between solid state and in solution analysis of high-valent arylbismuth–fluoride complexes. The study presented here sheds light onto the influence of substituents on the aryl groups in speciation of the complexes. This structural study serves as a roadmap for catalyst design and is essential for the efficient development of catalytic C–F bond formation strategies.^18b^

## Supporting Information Available

Experimental procedures and analytical data (^1^H, ^19^F, and ^13^C NMR and HRMS) for new compounds (PDF)

## Accession Codes

CCDC 2154888–2154894 contain the supplementary crystallographic data for this paper. These data can be obtained free of charge via www.ccdc.cam.ac.uk/data_request/cif, or by emailing data_request@ccdc.cam.ac.uk, or by contacting The Cambridge Crystallographic Data Centre, 12 Union Road, Cambridge CB2 1EZ, UK; fax: +44 1223 336033.
