# Supplementary material for: Synthesis and Structure of Mono-, Di-, and Trinuclear Fluorotriarylbismuthonium Cations
Source: Organometallics. 2022 May 9;41(14):1754–62. doi: 10.1021/acs.organomet.2c00135 (PMC9490813; doi:10.1021/acs.organomet.2c00135)
Supplement: Supplementary file 1 — om2c00135_si_001.pdf [file om2c00135_si_001.pdf]

Supporting Information

Synthesis and Structure of Mono-, Di- and Trinuclear Fluorotriarylbismuthonium Cations

Jennifer Kuziola, Marc Magre, Nils Nöthling and Josep Cornella*

Max-Planck-Institut für Kohlenforschung, D-45470 Mülheim/Ruhr, Germany

cornella@kofo.mpg.de

Table of Contents

1. General Methods 3

2. Syntheses of Triarylbismuthane **6** and **7** 4

    2.1 Synthesis of tris(3,5-di-tert-butylphenyl)bismuthane **6** 4

    2.2 Synthesis of Trimesitylbismuthane **7** 5

3. Synthesis of Pentavalent Dibismuthane **2** and Triarylbismuthdifluorides **8-10** 6

    3.1 Pentavalent Dibismuthane **2** 6

    3.2 Triarylbismuthdifluoride **8** 7

    3.2 Triarylbismuthdifluoride **9** 7

    3.2 Triarylbismuthdifluoride **10** 8

4. Synthesis of Fluorobismuthonium Cations **3, 4** and **11-13** 9

    4.1 Synthesis of **3** 9

    4.2 Synthesis of **4** 10

    4.3 Synthesis of **11** 11

    4.4 Synthesis of **12** 12

    4.5 Synthesis of **13** 12

5. NMR spectra 14

6. X-ray Single Crystal Analysis 39

7. References 124

1. General Methods

Unless otherwise stated, all manipulations were performed using standard Schlenk techniques under dry argon in flame-dried glassware. Anhydrous solvents were distilled from appropriate drying agents and were transferred under argon (Ar): tetrahydrofuran (Na/K) and tetrahydrofuran-d_8 (Na), acetonitrile (CaH) and acetonitrile-d_3 (MS), dichloromethane (CaH_2/P_4O_{10}) and dichloromethane-d_2 (MS), chloroform-*d* (MS). Commercially available xenon difluoride, bismuthtrichloride and triphenylbismuth were obtained from STREM, sodium *tetrakis*[3,5-*bis*(trifluoromethyl)phenyl]borate ($NaBAr^F$) was obtained from Alfa Aesar.

ESI-MS: ESQ 3000 (Bruker). High-resolution mass determinations: Bruker APEX III FT-MS (7 T magnet) or MAT 95 (Finnigan).

NMR spectra were recorded using 300 and 400 MHz Bruker Avance III and 600 MHz Bruker Avance III NMR spectrometers. ^1H NMR spectra (300.13 MHz, 400 MHz and 600.1 Hz) were referenced to the residual protons of the deuterated solvent and are reported to tetramethylsilane (δ_{TMS} = 0 ppm): chloroform-*d* (δ_{TMS} = 7.26 ppm), dichloromethane-d_2 (δ_{TMS} = 5.32 ppm), acetonitrile-d_3 (δ_{TMS} = 1.94 ppm), toluene-d_8 (δ_{TMS} = 2.09ppm), benzene-d_6 (δ_{TMS} = 7.16ppm), tetrahydrofurane-d_8 (δ_{TMS} = 3.58 ppm). $^{13}C\{^1H\}$ NMR spectra (75 MHz, 101 MHz, 125 MHz) were referenced internally to the D-coupled ^{13}C resonances of the NMR solvent and are reported to tetramethylsilane (δ_{TMS} = 0 ppm), chloroform-*d* (δ_{TMS} = 77.16 ppm) and dichloromethane-d_2 (δ_{TMS} = 53.84 ppm. ^{19}F NMR spectra were measured at 282 MHz and 585 MHz. Chemical shifts (δ) are given in ppm, relative to deuterated solvent residual peak, and coupling constants (*J*) provided in Hz.

The X-ray intensity data were collected using two different single crystal X-ray diffractometers: 1) Bruker-AXS Kappa Mach3 four cycle goniometer with APEX-II area detector and IµS microfocus Mo-anode X-ray source with focussing Incoatec Helios mirrors. 2) Bruker AXS Enraf-Nonius KappaCCD four cycle diffractometer and FR591 rotating Mo-anode X-ray source with Incoatec Helios focusing multilayer optics. The crystal structures were solved using SHELXT-2018/1 (Intrinsic phasing) [1] and refined with SHELXL-2018/3 (full-matrix least-squares against F^2) [2].

XantBis **1** was prepared according to the reported method.[3]

2. Synthesis of Triarylbismuthanes 6 and 7

2.1 Synthesis of *tris*(3,5-di-*tert*-butylphenyl)bismuthane (6)

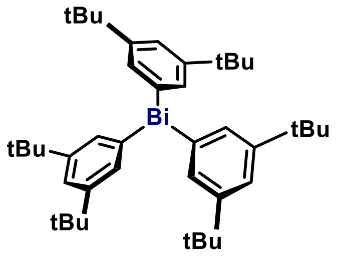

6

A flame-dried Schlenk flask was charged with magnesium turnings (325 mg, 13.4 mmol, 1.2 equiv.) activated with iodine (0.1 mg), and anhydrous THF (0.5 mL) under Ar atmosphere. In a separate flask under Ar atmosphere, a solution of 1-bromo-3,5-di-*tert*-butylbenzene (3.0 g, 11.14 mmol, 1.0 equiv.) in 15 mL of anhydrous THF was prepared. 1 mL of the 1-bromo-3,5-di-*tert*-butylbenzene solution in THF was added to the flask containing Mg turnings, and the mixture was gently heated with a heat gun. The remaining 1-bromo-3,5-di-*tert*-butylbenzene solution was then slowly added. Upon complete addition, the mixture was placed in an oil bath and stirred at 75 °C for 3 h. The magnesium turnings were filtered off under Ar and the Grignard solution was added dropwise to a solution of $BiCl_3$ (1.16 g, 3.7 mmol, 0.33 equiv.) in anhydrous THF (10 mL) at 0 °C. The mixture was allowed to warm up to room temperature and left to stir overnight. The solvent was then removed under vacuum and the crude solid was extracted with anhydrous *n*-pentane (3× 10 mL). After removal of anhydrous *n*-pentane, the remaining solid was washed with acetonitrile yielding to the desired complex **6** as a white solid.

Yield: 1.7 g (60%)

^{1}H NMR (300 MHz, $CDCl_3$): δ 7.53 (d, *J* = 1.9 Hz, 6H), 7.33 (t, *J* = 1.9 Hz, 3H), 1.21 (s, 54H).

^{13}C NMR (75 MHz, $CDCl_3$): δ 155.1, 152.1, 131.6, 121.4, 34.9, 31.6.

HRMS (ESI): calc'd for $C_{42}H_{63}Bi$ $[M+Na]^+$ 799.46191, found 799.46260.

Analytic data are in accordance with the values reported in the literature.[4]

2.2 Synthesis of Trimesitylbismuthane (7)

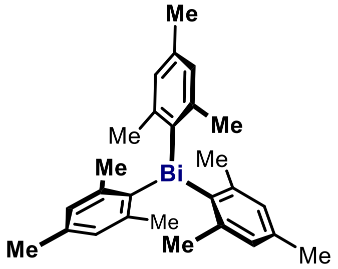

7

$BiCl_3$ (1.0 g, 3.17 mmol, 1 equiv.) was placed in a flame-dried Schlenk flask under Ar atmosphere and dissolved in 10 mL of anhydrous THF. The solution was cooled to 0 °C and a solution of 1 M of mesitylmagnesiumbromide in THF (9.5 mL, 9.5 mmol, 3 equiv.) was added dropwise while stirring. The mixture was then allowed to warm up to room temperature and left to stir overnight. The solvent was removed under vacuum and the crude solid was extracted with anhydrous *n*-pentane (3× 10 mL). After removal of *n*-pentane, the remaining solid was washed with acetonitrile yielding to the desired complex **7** as a white solid.

Yield: 810 mg (45%)

^{1}H NMR (300 MHz, $CDCl_3$): δ 6.97 (s, 6H), 2.32 (s, 18H), 2.27 (s, 9H).

^{13}C NMR (75 MHz, $CDCl_3$): δ 155.0, 145.9, 137.2, 129.0, 27.6, 20.9.

HRMS (ESI): calc'd for $C_{27}H_{32}Bi$ $[M-H]^+$ 565.23002, found 565.23025.

Analytic data are in accordance with the values reported in the literature.[5]

3. Synthesis of Pentavalent Dibismuthane Complex 2 and Triarylbismuthdifluorides 8-10

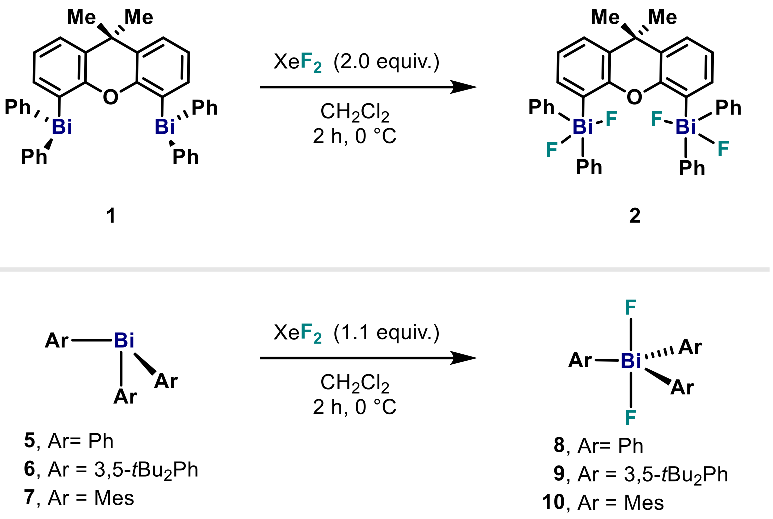

Scheme 1. Synthesis of complexes **2** (top) and **8-10** (bottom).

General Procedure: In a flame-dried Schlenk flask under Ar atmosphere, the triarylbismuth(III) was dissolved in anhydrous CH_2Cl_2 and cooled to 0 °C. Then, XeF_2 was added in one portion and the solution was stirred for 2 h at this temperature. The solvent was evaporated under vacuum, affording the corresponding pentavalent triarylbismuthdifluoride as white solids.

3.1 (9,9-Dimethyl-9*H*-xanthene-4,5-diyl)bis(difluorodiphenyl-λ^5-bismuthane) (2)

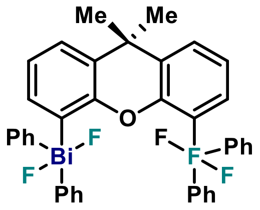

2

Compound **2** was prepared following the general procedure from XantBis[1] **1** (60 mg), 0.06 mmol, 1.0 equiv.) and XeF_2 (21.7 mg, 0.01 mmol, 2. equiv.) in 6 mL of anhydrous dichloromethane.

Yield: 62 mg (95%).

^{1}H NMR (300 MHz, $CDCl_3$): δ 8.06 (dd, *J* = 8.3, 1.3 Hz, 8H), 7.89 (dd, *J* = 7.9, 1.4 Hz, 2H), 7.57 (dd, *J* = 7.7, 1.4 Hz, 2H), 7.53 – 7.44 (m, 8H), 7.41 – 7.30 (m, 6H), 1.71 (s, 6H).

^{13}C NMR (75 MHz, $CDCl_3$): δ 159.3, 153.2, 136.1, 134.0, 132.7, 130.9, 130.9, 127.8, 126.4, 37.1, 29.1.[a]

^{19}F NMR (282 MHz, $CDCl_3$): δ –127.23.

HRMS (ESI): calc. for $C_{39}H_{32}Bi_2F_3O$ $[M-F]^+$ 991.201290, found 991.20074.

[a]Note: One carbon signal was not observed in the ^{13}C NMR spectra.

3.2 Triphenylbismuthdifluoride (8)

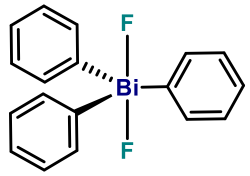

8

Compound **8** was prepared following the general procedure from triphenylbismuth (100 mg, 0.23 mmol, 1.0 equiv.) and XeF_2 (42.3 mg, 0.24 mmol, 1.1 equiv.) in 10 mL of anhydrous dichloromethane.

Yield: 101 mg (93%).

^1H NMR (300 MHz, $CDCl_3$): δ 8.24 – 8.20 (m, 6H), 7.65 (dd, *J* = 8.4, 7.1 Hz, 6H), 7.51 – 7.45 (m, 3H).

^{13}C NMR (75 MHz, $CDCl_3$): δ 153.6, 134.3, 131.9, 131.4.

^{19}F NMR (300 MHz, $CDCl_3$): δ –160.92.

HRMS (ESI): calc'd for $C_{18}H_{15}Bi_1F_2$ Na $[M+Na]^+$ 501.08351, found 501.08380.

Analytic data are in accordance with the values reported in the literature. [6]

3.3 Tris(3,5-di-*tert*-butylphenyl)bismuthdifluoride (9)

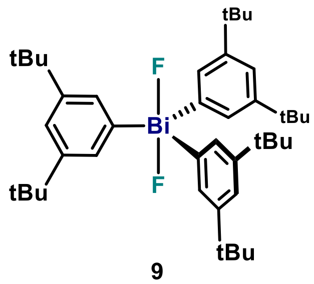

9

Compound **9** was prepared following the general procedure from *tris*(3,5-di-*tert*-butylphenyl)bismuthane **6** (70 mg, 0.09 mmol, 1.0 equiv.) and XeF_2 (16.7 mg, 0.09 mmol, 1.1 equiv.) in 7 mL of anhydrous dichloromethane.

Yield: 70.5 mg (96%).

^1H NMR (300 MHz, $CDCl_3$): δ 8.09 (d, *J* = 1.8 Hz, 6H), 7.51 (t, *J* = 1.7 Hz, 3H), 1.36 (s, 54H).

^{13}C NMR (75 MHz, $CDCl_3$): δ 155.1, 152.1, 131.6, 121.4, 34.9, 31.4.

^{19}F NMR (300 MHz, $CDCl_3$): δ –160.92.

HRMS (ESI): calc'd for $C_{42}H_{63}BiF$ $[M-F]^+$ 795.471540 , found 795.471210.

X-ray quality crystals were obtained via a phase transfer diffusion (5:1) of hexane into a concentrated solution of complex **9** in dichloromethane at ambient temperature.

3.4 Trimesitylbismuthdifluoride (10)

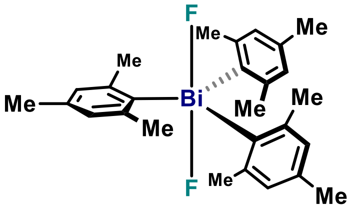

10

Compound **10** was prepared following the general procedure from trimesitylbismuthane **7** (120 mg, 0.21 mmol, 1.0 equiv.) and XeF_2 (39.5 mg, 0.23 mmol, 1.1 equiv.) in 10 mL of anhydrous dichloromethane.

Yield: 125 mg (97%)

^{1}H NMR (300 MHz, $CDCl_3$): δ 7.11 (s, 6H), 2.59 (s, 18H), 2.33 (s, 9H).

^{13}C NMR (75 MHz, $CDCl_3$): δ 160.9, 142.7, 140.9, 131.2, 23.2, 21.0.

^{19}F NMR (300 MHz, $CDCl_3$): δ –100.41.

HRMS (ESI): calc'd for $C_{27}H_{33}BiF_2Na$ $[M+Na]^+$ 627.22427, found 627.22465.

X-ray quality crystals were obtained via a phase transfer diffusion (5:1) of hexane into a concentrated solution of complex **10** in dichloromethane at ambient temperature.

4 Synthesis of Fluorobismuthonium Cations 3,4 and 11-13

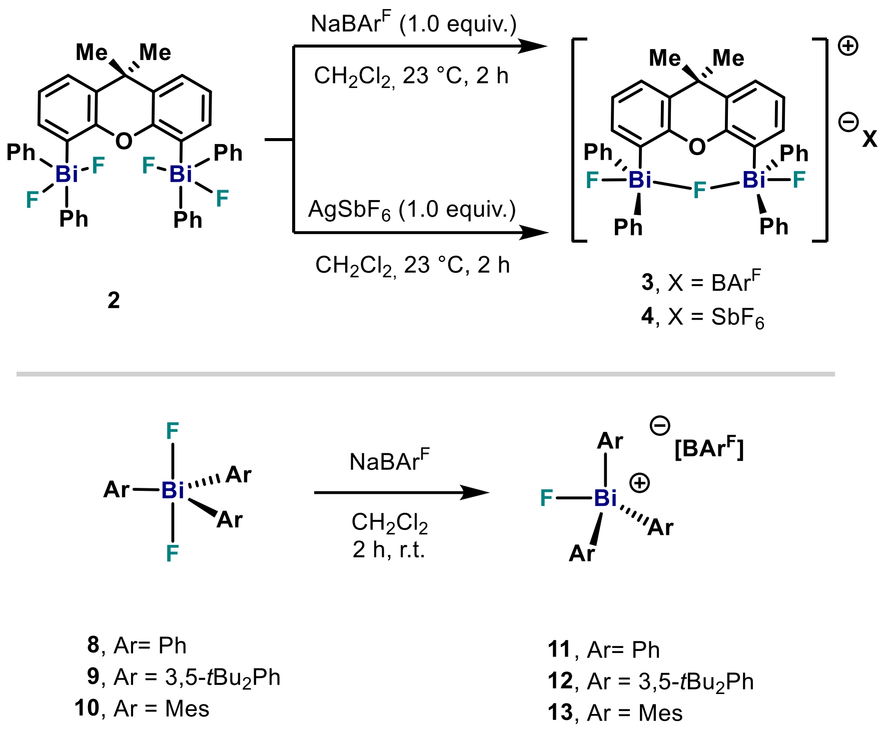

Scheme 2. Synthesis of fluorobismuthonium cations **3, 4** (top) and **11-13** (bottom).

General Procedure: In a flame-dried Schlenk flask under Ar atmosphere, the corresponding triarylbismuthdifluoride was dissolved in anhydrous CH_2Cl_2 and $NaBAr^F$ was added in one portion. The reaction was stirred for 2 h at ambient temperature and the precipitate was filtered off. The solvent was evaporated affording the corresponding fluorobismuthonium cations **3, 11-13** a white solid. For the synthesis of complex **4** $AgSbF_6$ was used instead of $NaBAr^F$.

4.1 5,7-Difluoro-13,13-dimethyl-5,5,7,7-tetraphenyl-5*H*,7*H*-5λ^5,6λ^2,7λ^5-1,11-methano-dibenzo[*c*,*f*][1,5,2,8]fluoraoxadibismocine *tetrakis*[3,5-*bis*(trifluoromethyl)-phenyl]-borat (3)

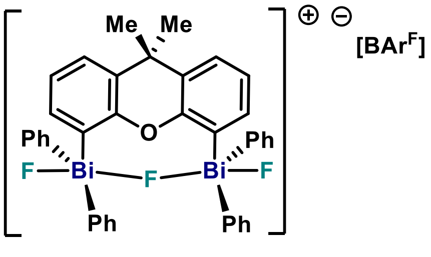

3

Compound **3** was prepared following the general procedure from (9,9-dimethyl-9*H*-xanthene-4,5-diyl)bis-difluorodiphenyl-bismuthane **2** (50 mg, 0.05 mmol, 1.0 equiv.) and $NaBAr^F$ (43.8 mg, 0.05 mmol, 1.0 equiv.) in 5 mL of anhydrous dichloromethane.

Yield: 88 mg (96%)

^{1}H NMR (300 MHz, $CDCl_3$): δ 8.19 (dt, J = 98.1, 1.7 Hz, 2H), 7.79 (dd, *J* = 7.7, 1.3 Hz, 2H), 7.71 – 7.68 (m, 16H), 7.66 (t, *J* = 7.9 Hz, 3H), 7.48 – 7.44 (m, 16H), 1.73 (s, 6H).

^{13}C NMR (151 MHz, $CDCl_3$): δ 155.80 (t, *J* = 9.5 Hz), 152.69, 137.19 (d, *J* = 6.7 Hz), 135.95, 134.77 (dd, *J* = 4.4, 2.5 Hz), 133.20 (dd, *J* = 3.4, 1.8 Hz), 132.92, 132.86, 132.48, 130.82, 128.82 (ddd, *J* = 31.4, 5.8, 2.8 Hz), 128.56, 124.49 (q, *J* = 272.6 Hz), 117.41 (p, *J* = 4.1 Hz), 37.38, 29.99.

^{19}F NMR (565 MHz, $CDCl_3$): δ –60.48, –105.36 (t, *J* = 98.2 Hz), –156.04 (d, *J* = 98.0 Hz).

HRMS (ESI): calc. for $C_{39}H_{32}Bi_2F_3O$ [M-BAr^F]$^+$ 991.202570, found 991.20074.

X-ray quality crystals were obtained from a phase transfer diffusion (5:1) of pentane into a concentrated solution of compound **3** in dichloromethane at +5 °C.

4.2 5,7-Difluoro-13,13-dimethyl-5,5,7,7-tetraphenyl-5*H*,7*H*-5λ^5,6λ^2,7λ^5-1,11-methano-dibenzo[*c*,*f*][1,5,2,8]fluoraoxadibismocine hexafluoroantimonate (4)

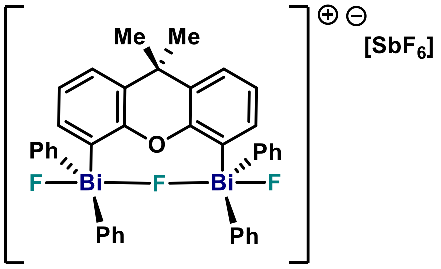

4

Compound **4** was prepared following the general procedure from (9,9-Dimethyl-9*H*-xanthene-4,5-diyl)bis-difluorodiphenyl-bismuthane **2** (50 mg, 0.05 mmol, 1.0 equiv.) and $AgSbF_6$ (17.0 mg, 0.05 mmol, 1.0 equiv.) in 5 mL of anhydrous dichloromethane.

Yield: 56.5 mg (93%)

^{1}H NMR (300 MHz, $CDCl_3$): δ 8.15 (d, *J* = 7.9 Hz, 2H), 7.88 – 7.73 (m, 10H), 7.65 (t, *J* = 7.8 Hz, 2H), 7.59 – 7.33 (m, 12H), 1.80 (s, 6H).

^{13}C NMR (75 MHz, $CDCl_3$): δ 157.4, 152.9, 138.0, 136.0, 133.4, 132.7, 132.3, 130.5, 128.0, 37.4, 30.2.[a]

^{19}F NMR (282 MHz, $CDCl_3$): δ –106.92 (t, *J* = 97.3 Hz), –156.58 (d, *J* = 97.2 Hz).[b]

HRMS (ESI): calc. for $C_{39}H_{32}Bi_2F_3O$ [M-SbF_6]$^+$ 991.201760, found 991.20074.

X-ray quality crystals were obtained from a phase transfer diffusion (5:1) of pentane into a concentrated solution of compound **4** in dichloromethane at +5 °C.

[a] Note: One carbon signal was not observed in the ^{13}C NMR spectra.

[b] Note: The fluorine peak of the anion $[SbF_6]^-$ could not be observed in the ^{19}F NMR analysis. However, the corresponding counter anion was detected by mass analysis as sole negatively charged species.

4.3 Bis((fluorotriphenyl-λ⁵-bismuthaneyl)-λ²-fluoraneyl)triphenyl- λ⁵-bismuthonium (11) & bis(fluorotriphenyl-λ⁵-bismuthaneyl)-λ²-fluorane *tetrakis*[3,5-*bis*(trifluoromethyl)-phenyl]borat (15) (dimer)

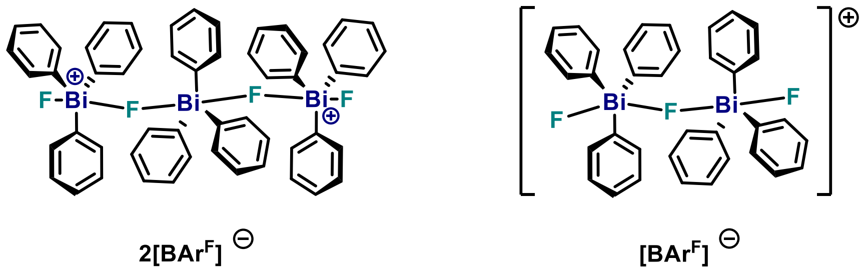

11 [in solid state] **15** [in solution]

Compound **11** was prepared following the general procedure from triphenylbismuthdifluoride **8** (50 mg, 0.1 mmol, 1 equiv.) and $NaBAr^F$ (46.32 mg, 0.05 mmol, 0.5 equiv.) in 5 mL of anhydrous dichloromethane. The analytical data refer to the dinuclear fluorobismuthonium species **15** in solution and the yield is based on the trinuclear structure **11** in solid state.

Yield: 99 mg (92%)

^{1}H NMR (600 MHz, CD_2Cl_2): δ 7.72 (p, *J* = 2.4 Hz, 8H), 7.62 (d, *J* = 6.8 Hz, 30H), 7.55 (dt, *J* = 2.1, 1.1 Hz, 4H).

^{13}C NMR (151 MHz, CD_2Cl_2): δ 162.2 (dd, *J* = 99.7, 49.8 Hz), 153.2, 135.2, 134.0, 133.7, 133.2, 129.6 – 129.0 (m), 125.00 (q, *J* = 272.4 Hz), 117.9 (p, *J* = 4.1 Hz).

^{19}F NMR (470 MHz, CD_2Cl_2, 25 °C): δ –62.87, –123.62 (t, *J* = 91.1 Hz), –162.21 (d, *J* = 90.3 Hz). **^{19}F NMR (470 MHz, CD_2Cl_2, –80°C):** δ –62.37, –127.94 (t, *J* = 93.8 Hz), –163.55 (d, *J* = 93.7 Hz).

HRMS (ESI): calc'd for $C_{36}H_{30}Bi_2F_3$ $[M\text{-}BAr^F]^+$ 937.18929 found 937.19021.

X-ray quality crystals were obtained a phase transfer diffusion (5:1) of hexane into a concentrated solution of complex **11** in dichloromethane at +5 °C.

4.4 Bis(tris(3,5-di-tert-butylphenyl)fluoro- λ5-bismuthaneyl)- λ2-fluorane *tetrakis*[3,5-*bis*(trifluoromethyl)phenyl]borat (12)

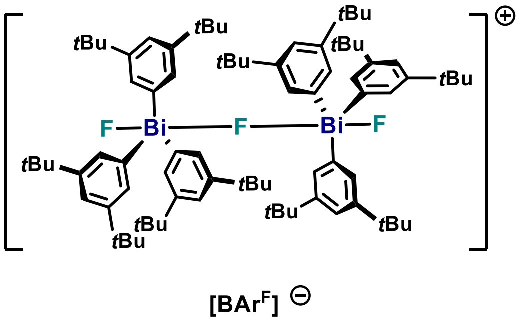

12

Compound **12** was prepared following the general procedure from triarylbismuthdifluoride **9** (50 mg, 0.06 mmol, 1.0 equiv.) and NaBAr^F (27.1 mg, 0.03 mmol, 0.5 equiv.) in 5 mL of anhydrous dichloromethane.

Yield: 71 mg (93%)

^{1}H NMR (600 MHz, $CDCl_3$): δ 7.76 (d, *J* = 1.6 Hz, 12H), 7.73 – 7.67 (m, 8H), 7.58 (t, *J* = 1.6 Hz, 6H), 7.52 (d, *J* = 2.0 Hz, 4H), 1.08 (s, 108H).

^{13}C NMR (151 MHz, $CDCl_3$): δ 161.9 (dd, *J* = 99.7, 49.9 Hz), 156.4, 155.8, 135.0, 129.4 – 128.6 (m), 128.4, 127.8 (d, *J* = 3.4 Hz), 124.7 (q, *J* = 272.5 Hz), 117.6 (q, *J* = 4.1 Hz), 36.1, 31.2.

^{19}F NMR (565 MHz, $CDCl_3$): δ –62.46, –152.78(t, *J* = 102.7 Hz), –162.06 (d, *J* = 102.9 Hz).

HRMS (ESI): calc'd for $C_{84}H_{126}Bi_2F_3$ [M-BAr^F]$^+$ 1609.94116, found 1609.94141.

X-ray quality crystals were obtained a phase transfer diffusion (5:1) of hexane into a concentrated solution of complex **12** in dichloromethane at ambient temperature.

4.1.5 Fluorotrimesityl-λ4-bismuthonium *tetrakis*[3,5-*bis*(trifluoromethyl)phenyl]borate (13)

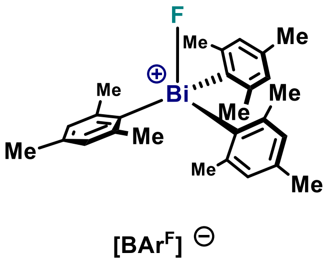

13

Compound **13** was prepared following the general procedure from trimesitylbismuthdifluoride **10** (50 mg, 0.08 mmol, 1.0 equiv.) and NaBAr^F (73.3 mg, 0.08 mmol, 1.0 equiv.) in 5 mL of anhydrous dichloromethane.

Yield: 114 mg (95%)

^{1}H NMR (300 MHz, $CDCl_3$): δ 7.69 (p, *J* = 2.2 Hz, 8H), 7.49 (s, 4H), 7.30 (s, 6H), 2.39 (s, 18H), 2.34 (s, 9H).

^{13}C NMR (151 MHz, $CDCl_3$): δ 161.80 (q, *J* = 49.8 Hz), 158.48 (d, *J* = 8.0 Hz), 145.99, 141.82, 134.95, 133.83, 129.04 (qq, *J* = 31.4, 2.8 Hz), 124.67 (q, *J* = 272.5 Hz), 117.59 (m, *J* = 4.0 Hz), 23.11 (d, *J* = 2.2 Hz), 21.27.

^{19}F NMR (300 MHz, $CDCl_3$): δ –62.46, –177.11.

HRMS (ESI): calc'd for $C_{27}H_{33}BiF$ [M-BAr^F]$^+$ 585.236390, found 585.236502.

X-ray quality crystals were obtained a phase transfer diffusion (5:1) of pentane into a concentrated solution of complex **13** in dichloromethane at -18 °C.

5. NMR Spectra

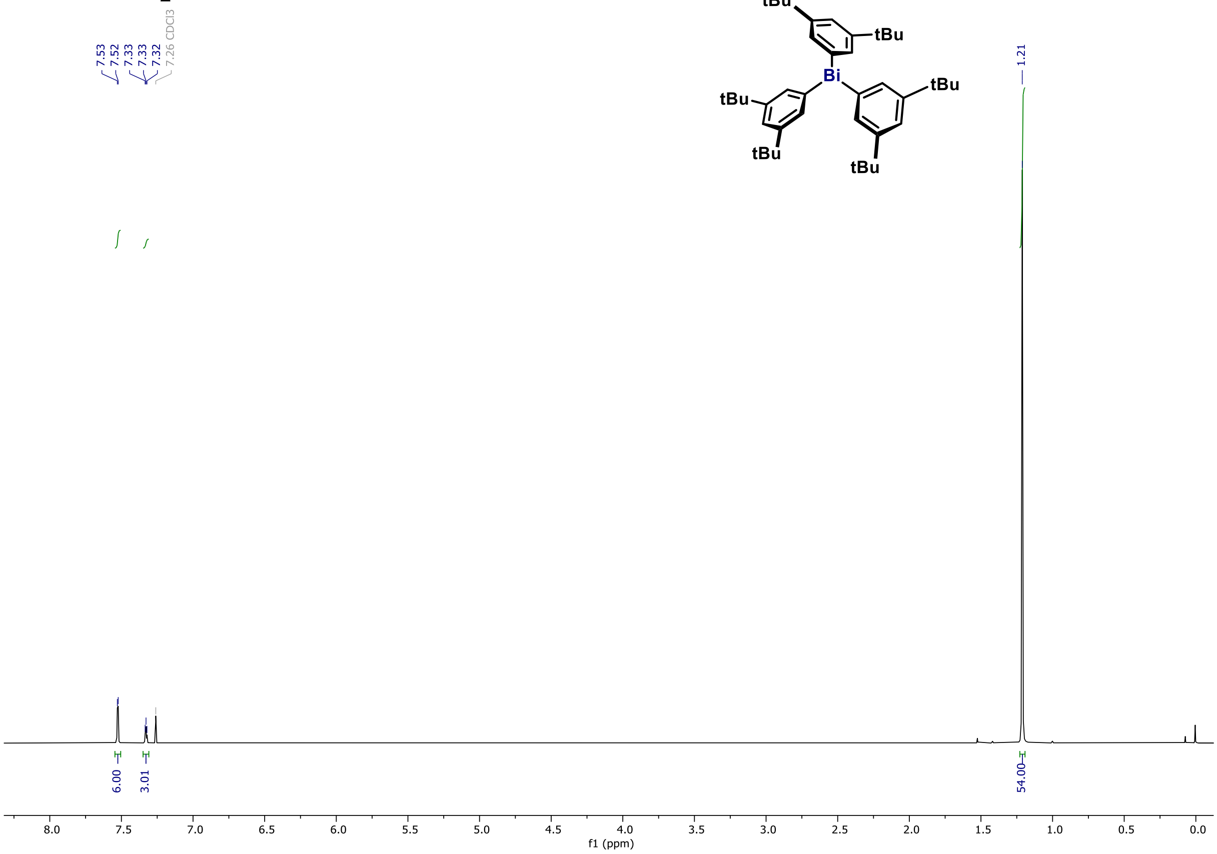

Figure S1. ^{1}H NMR (300 MHz, $CDCl_3$) of **6.**

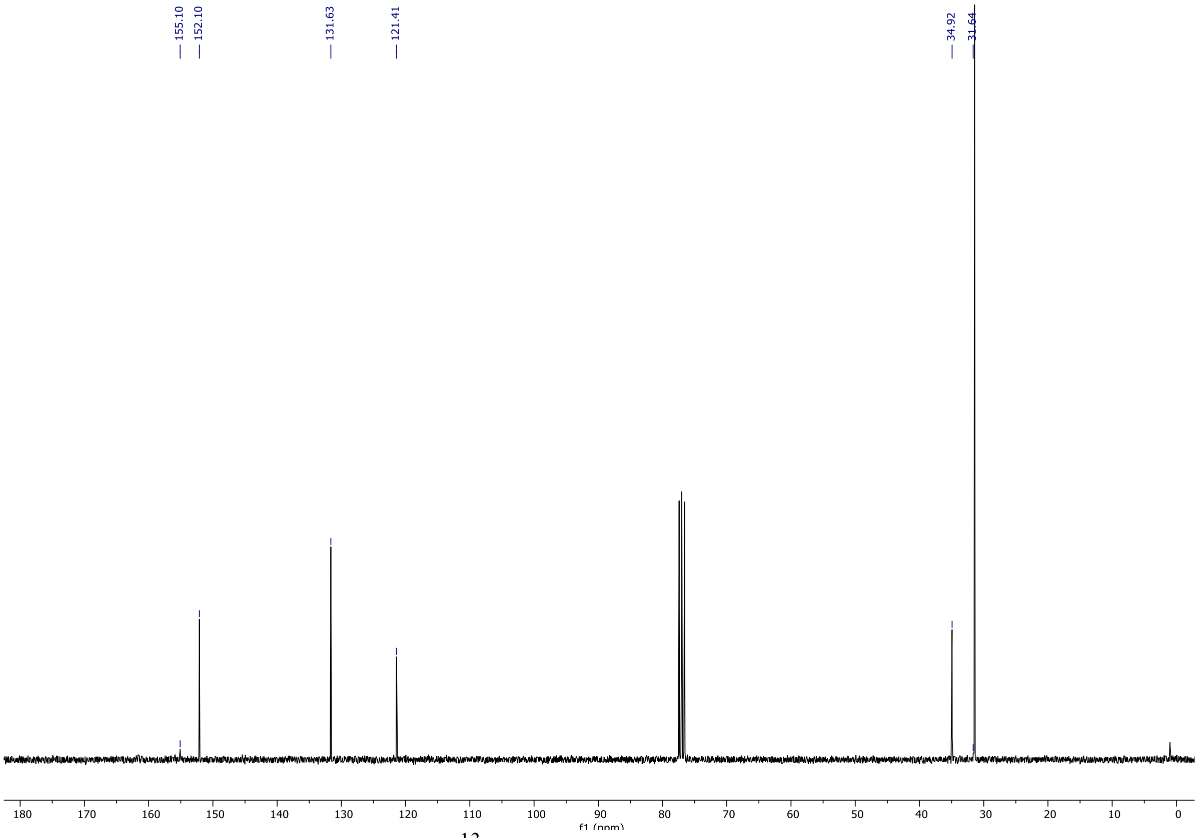

Figure S2. ^{13}C NMR (75 MHz, $CDCl_3$) of **6.**

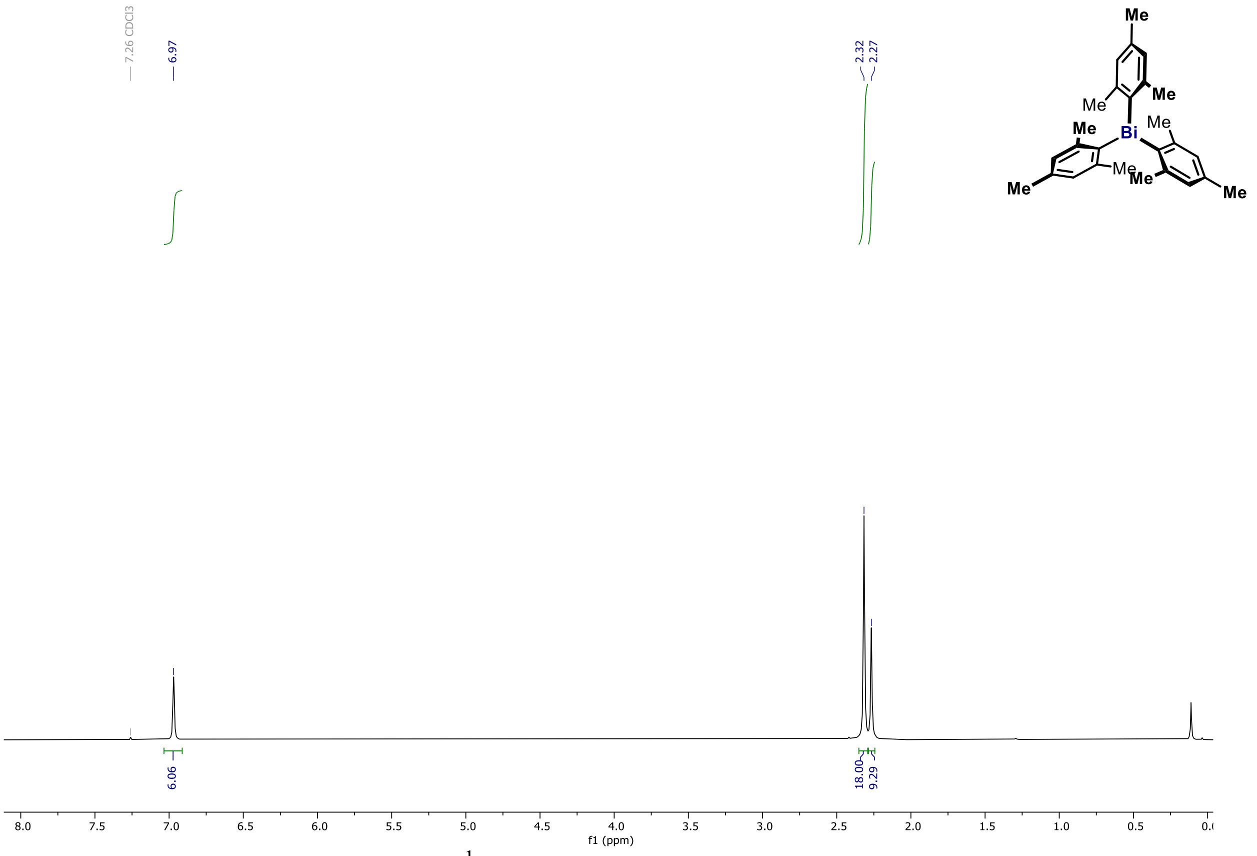

Figure S3. ^{1}H NMR (300 MHz, $CDCl_3$) of **7.**

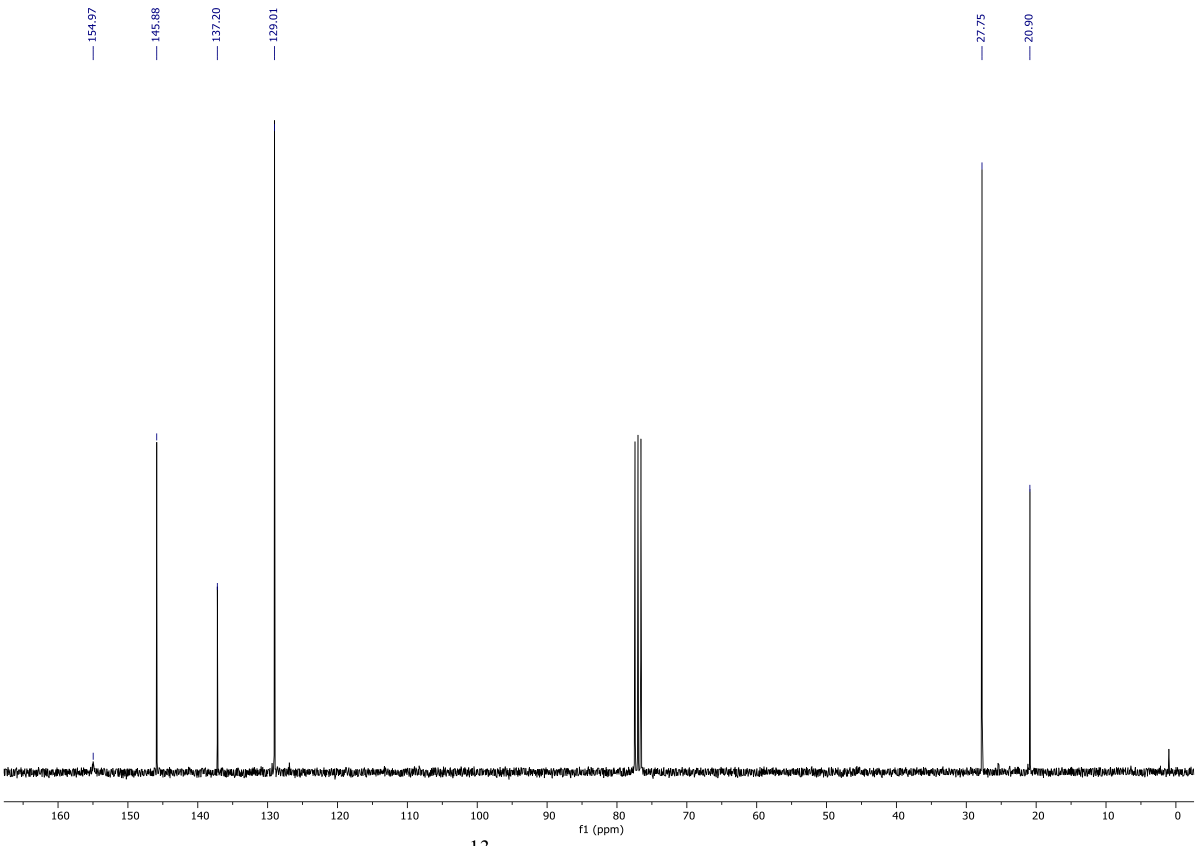

Figure S4. ^{13}C NMR (75 MHz, $CDCl_3$) of **7.**

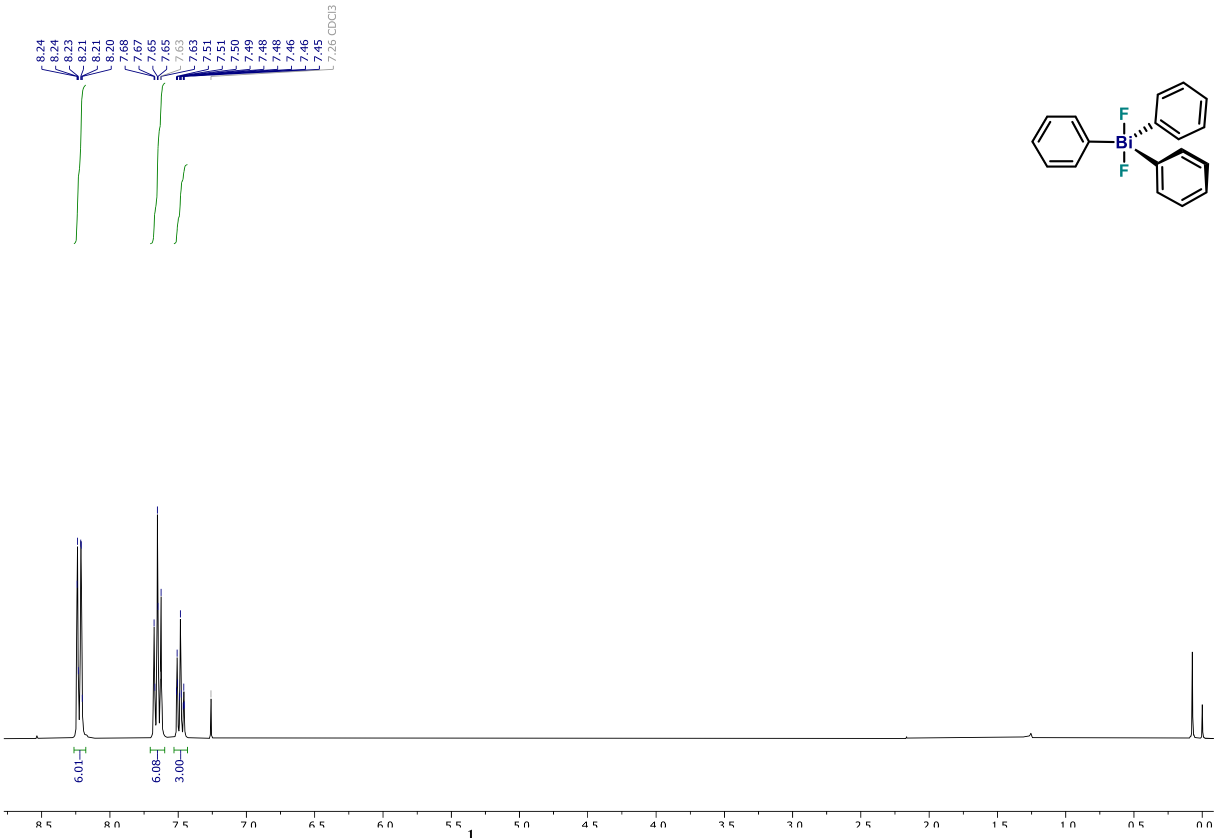

Figure S5. ^{1}H NMR (300 MHz, $CDCl_3$) of **8.**

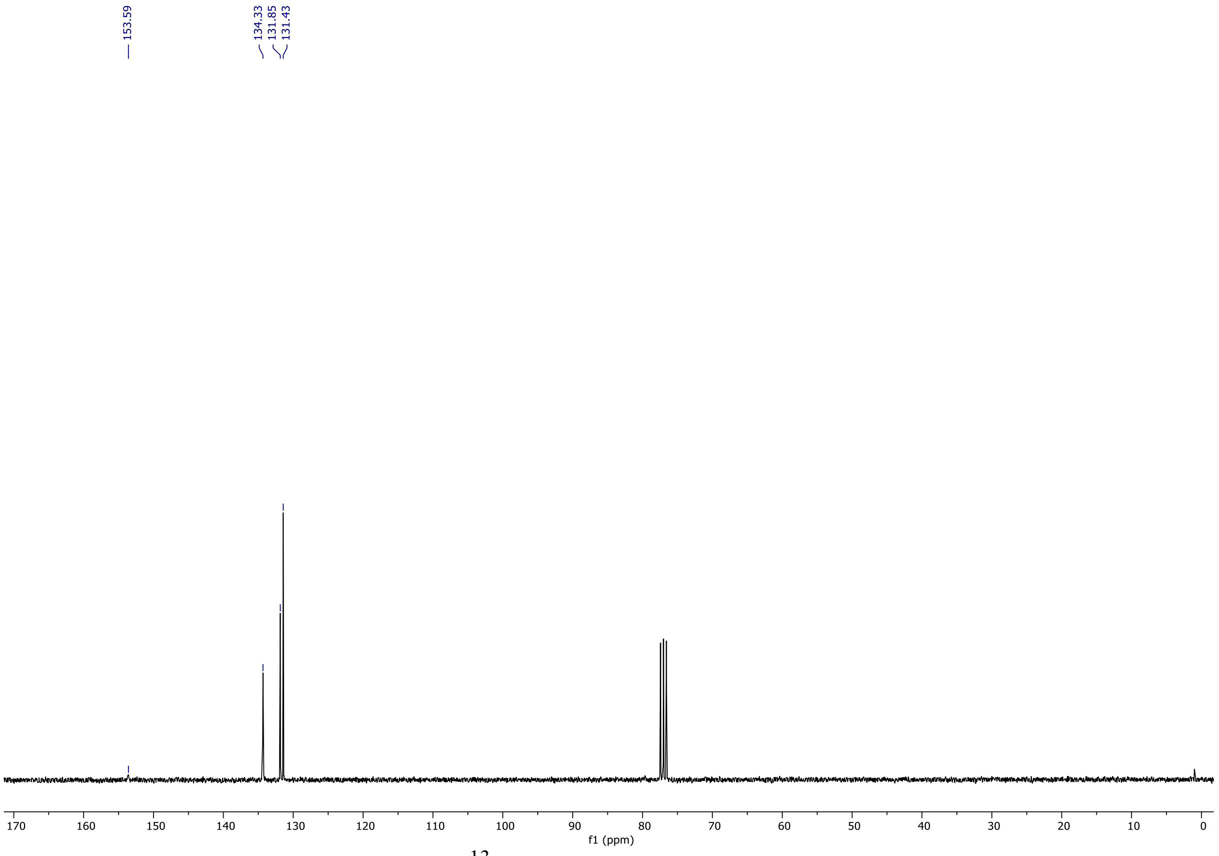

Figure S6. ^{13}C NMR (75 MHz, $CDCl_3$) of **8.**

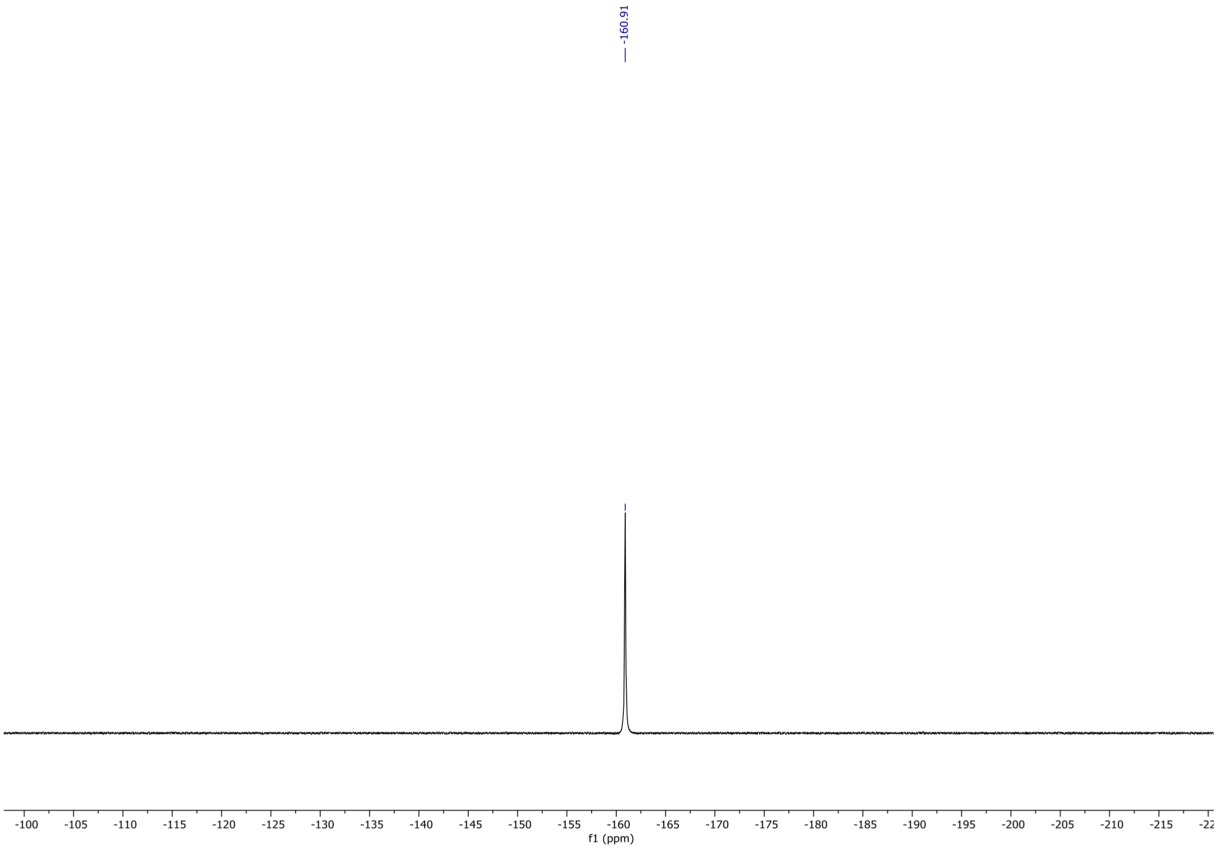

Figure S7. ^{19}F NMR (282 MHz, $CDCl_3$) of **8.**

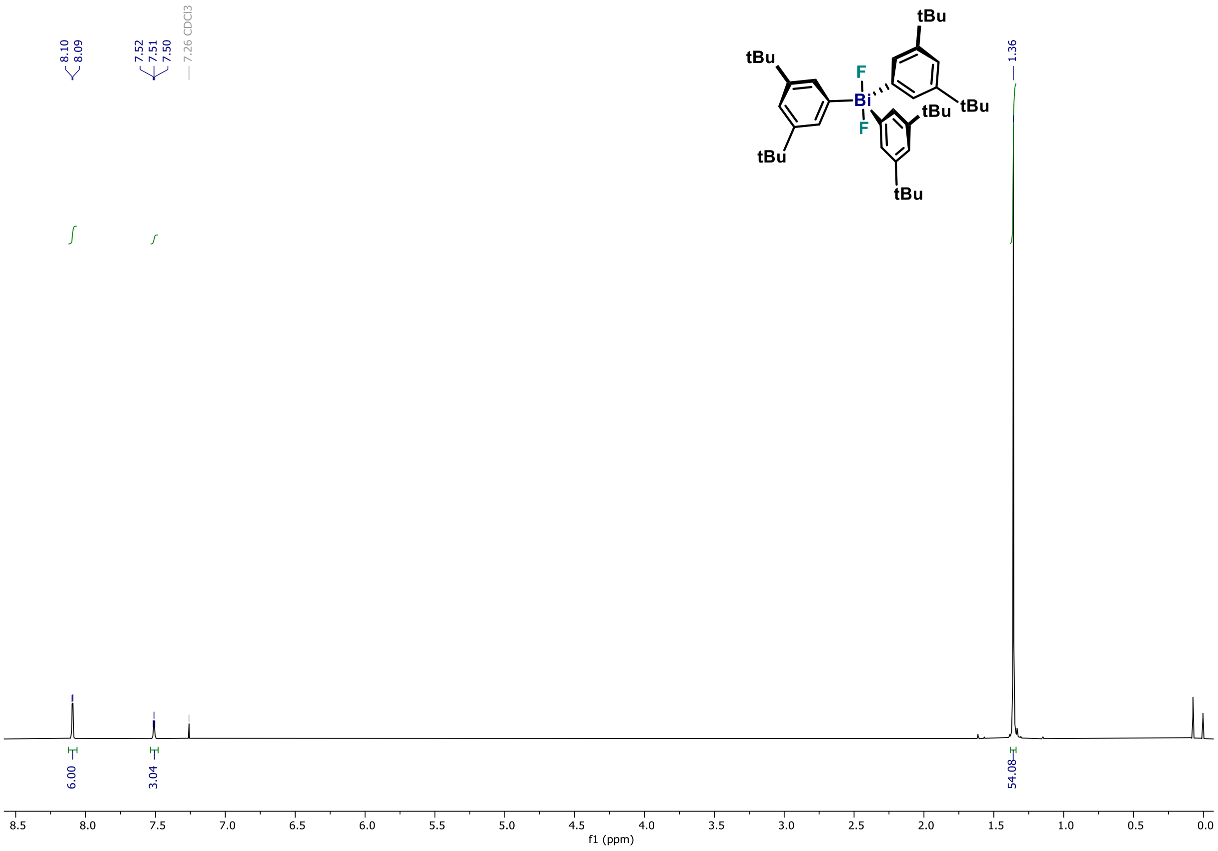

Figure S8. ^{1}H NMR (300 MHz, $CDCl_3$) of **9.**

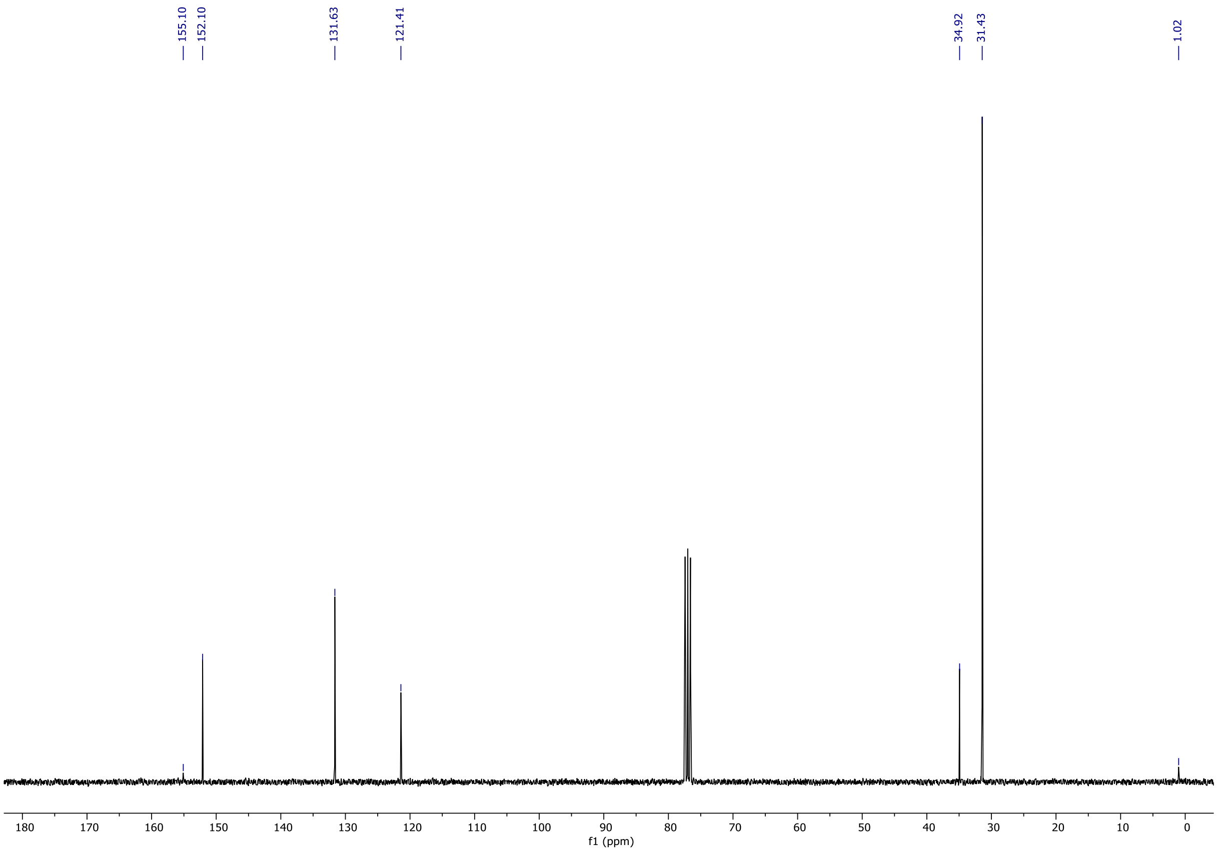

Figure S9. ^{13}C NMR (75 MHz, $CDCl_3$) of **9.**

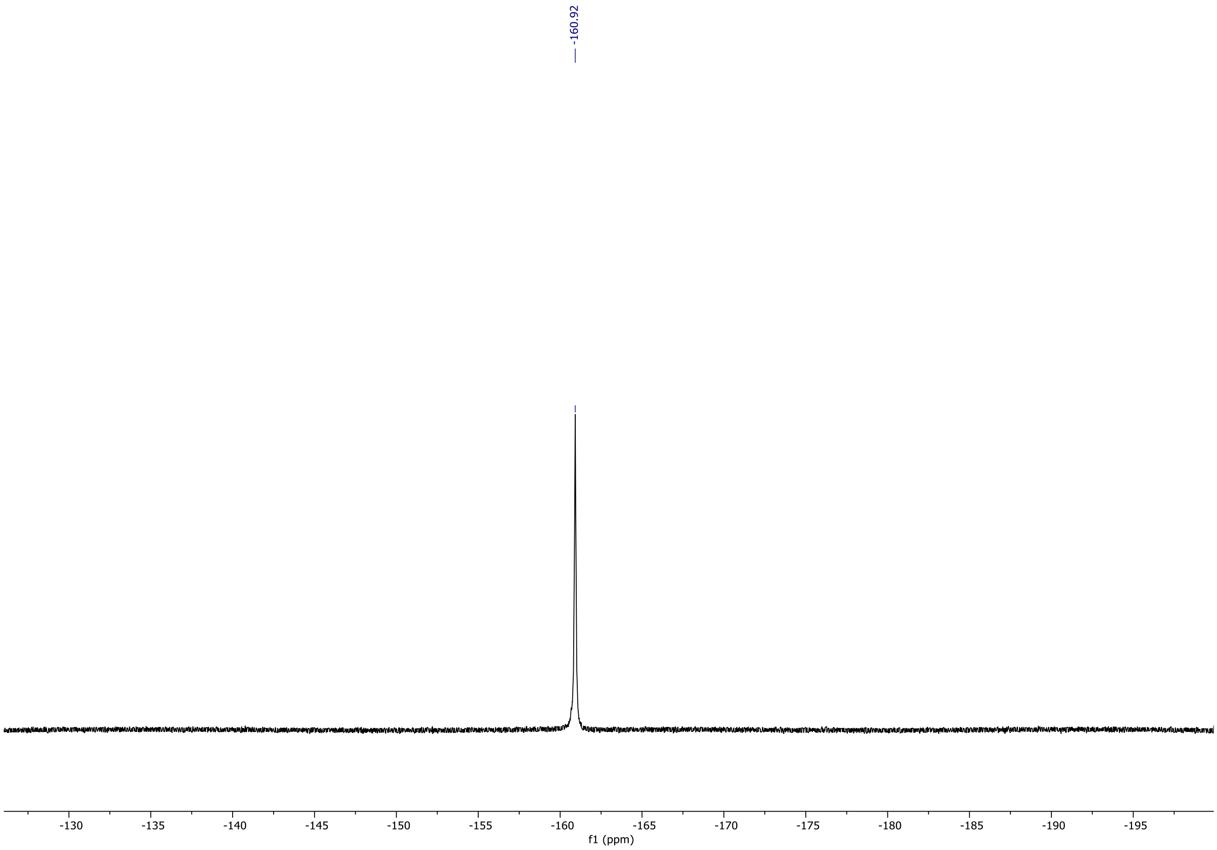

Figure S10. ^{19}F NMR (282 MHz, $CDCl_3$) of **9.**

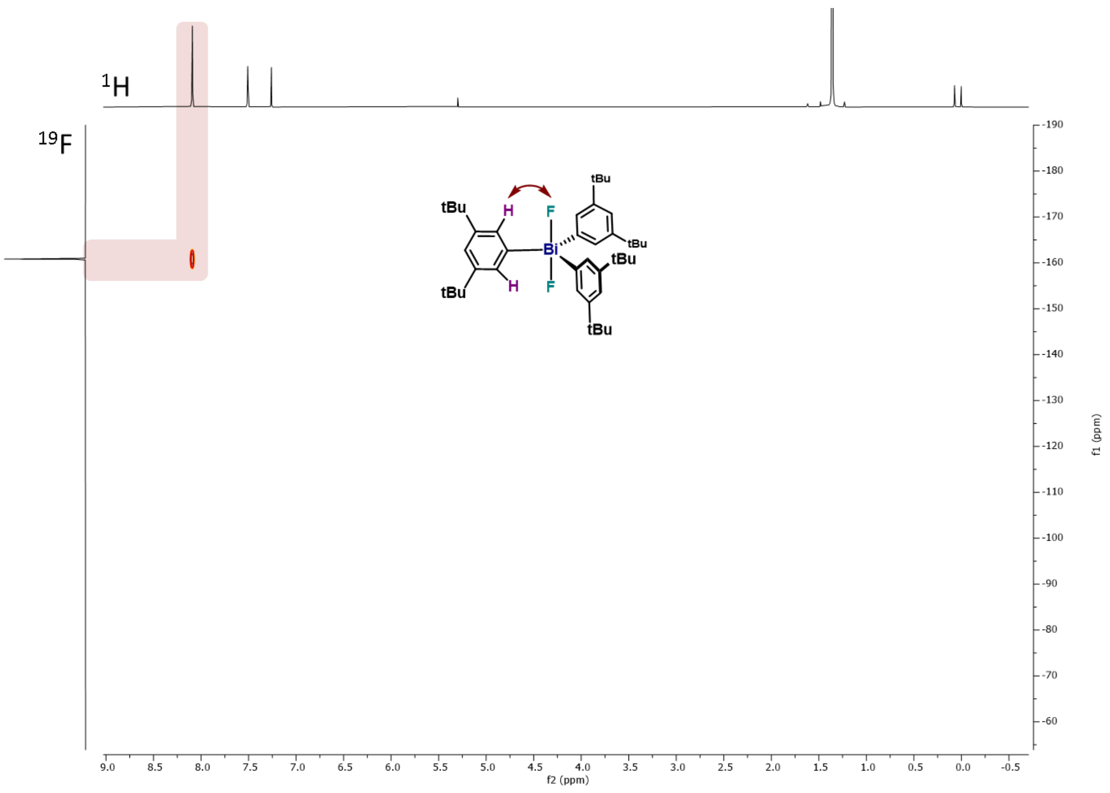

Figure S11. ^{1}H-^{19}F HOESY NMR (470 MHz, $CDCl_3$) of compound **9.**
H···F correlation in compound 9 (highlighted in red).

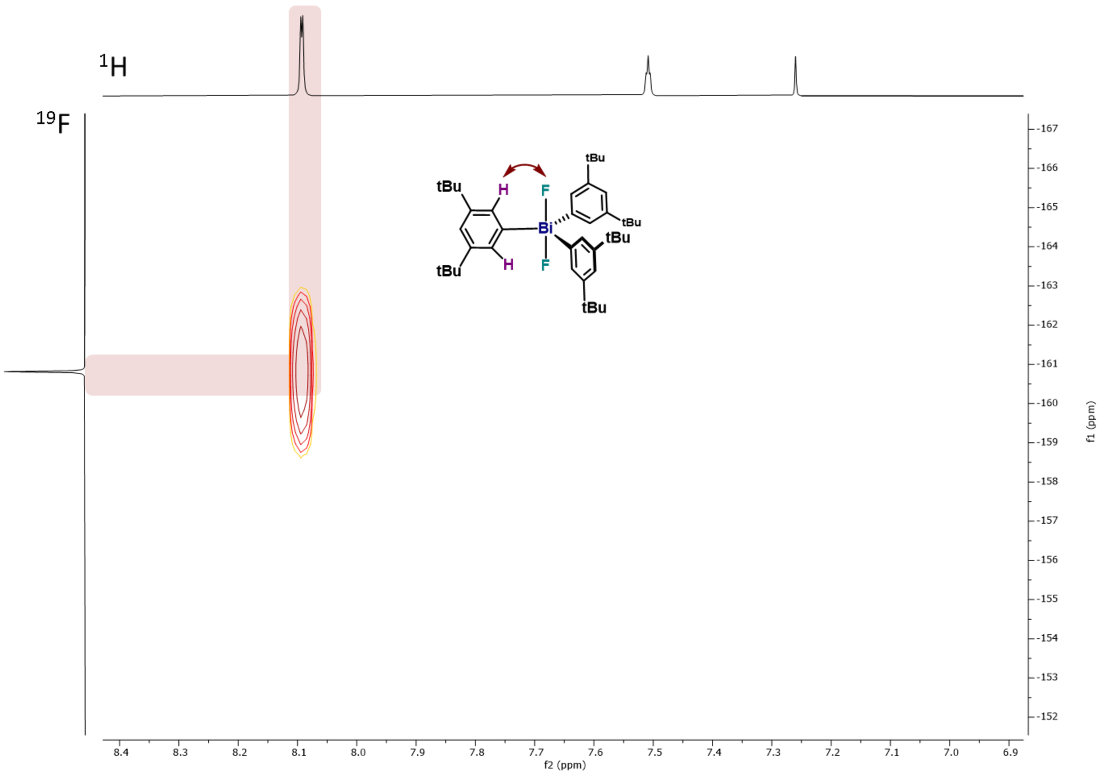

Figure S12. ^{1}H-^{19}F HOESY NMR (470 MHz, $CDCl_3$) of compound **9.**
Zoom area: (6.80 to 8.50 ppm / –151 to –168 ppm).

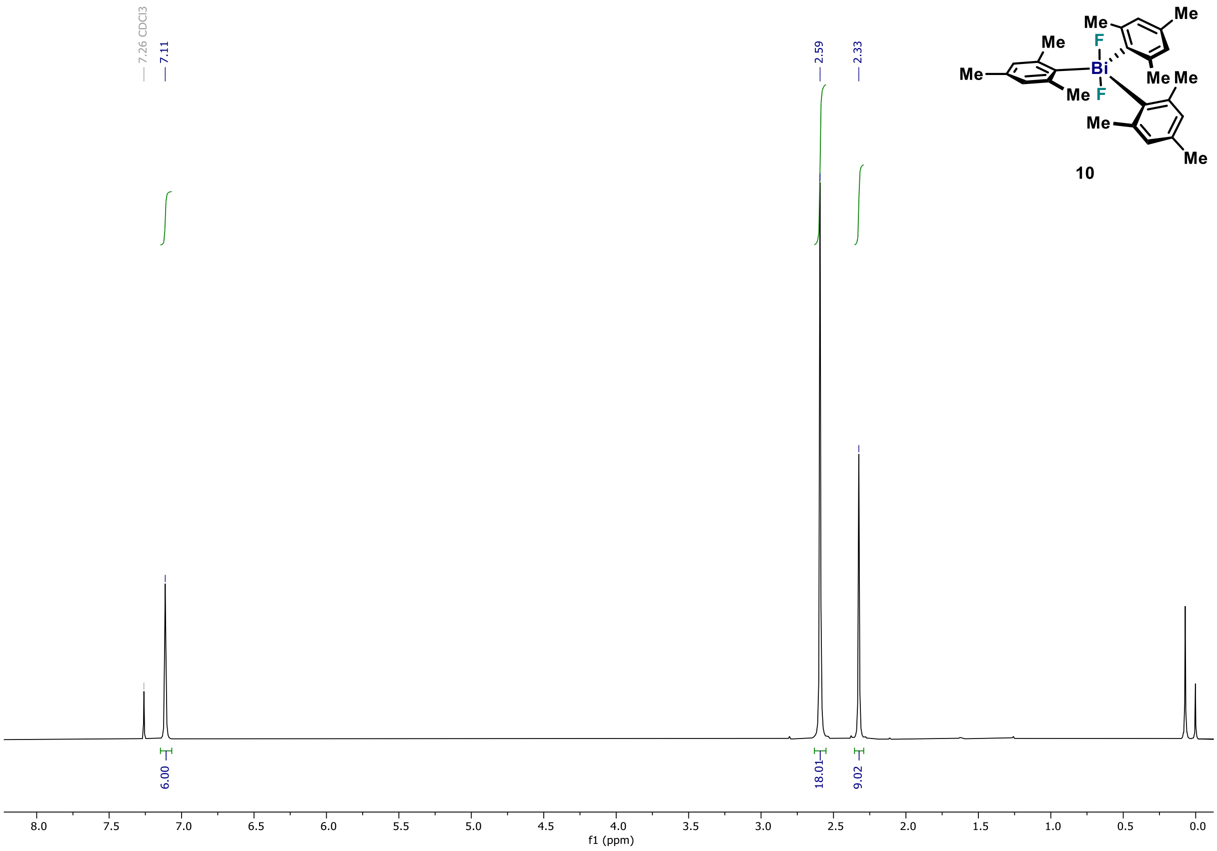

Figure S13. ^{1}H NMR (300 MHz, $CDCl_3$) of **10.**

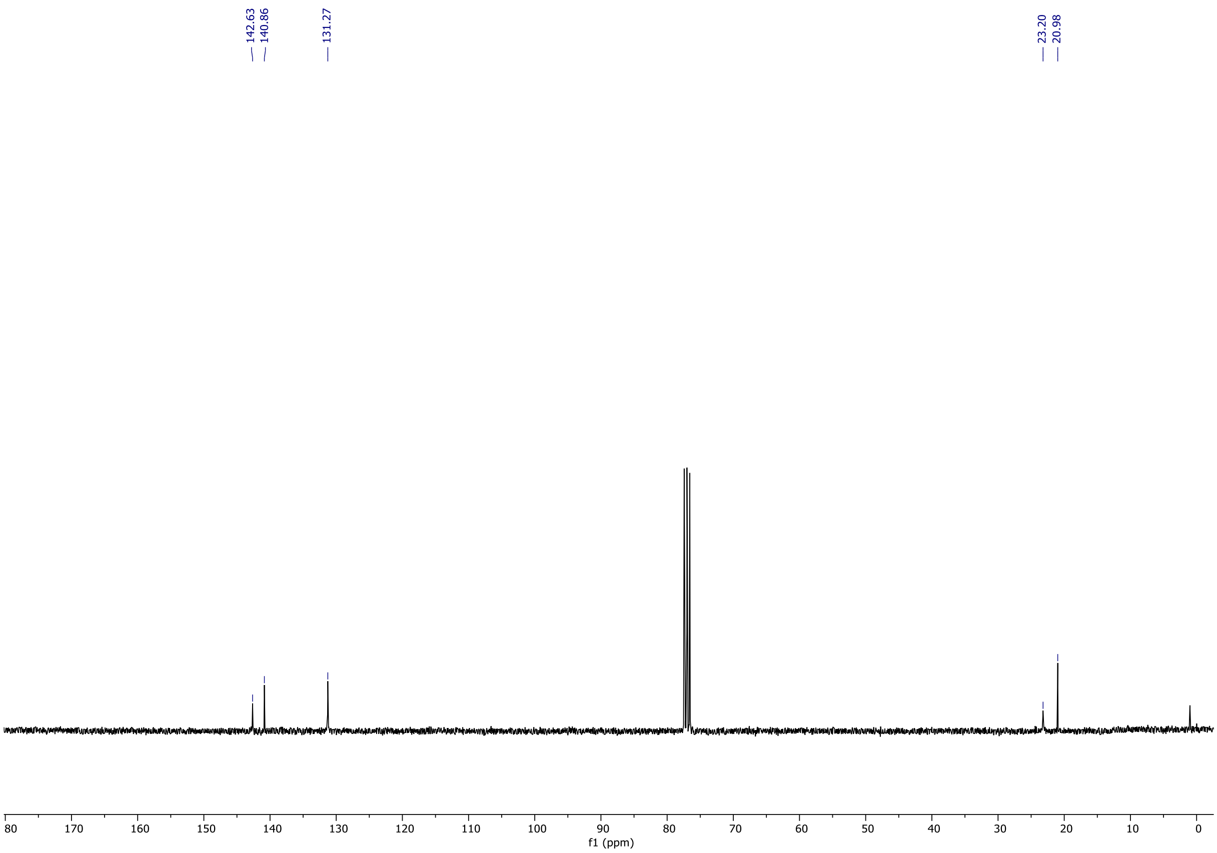

Figure S14. ^{13}C NMR (101 MHz, $CDCl_3$) of **10.**

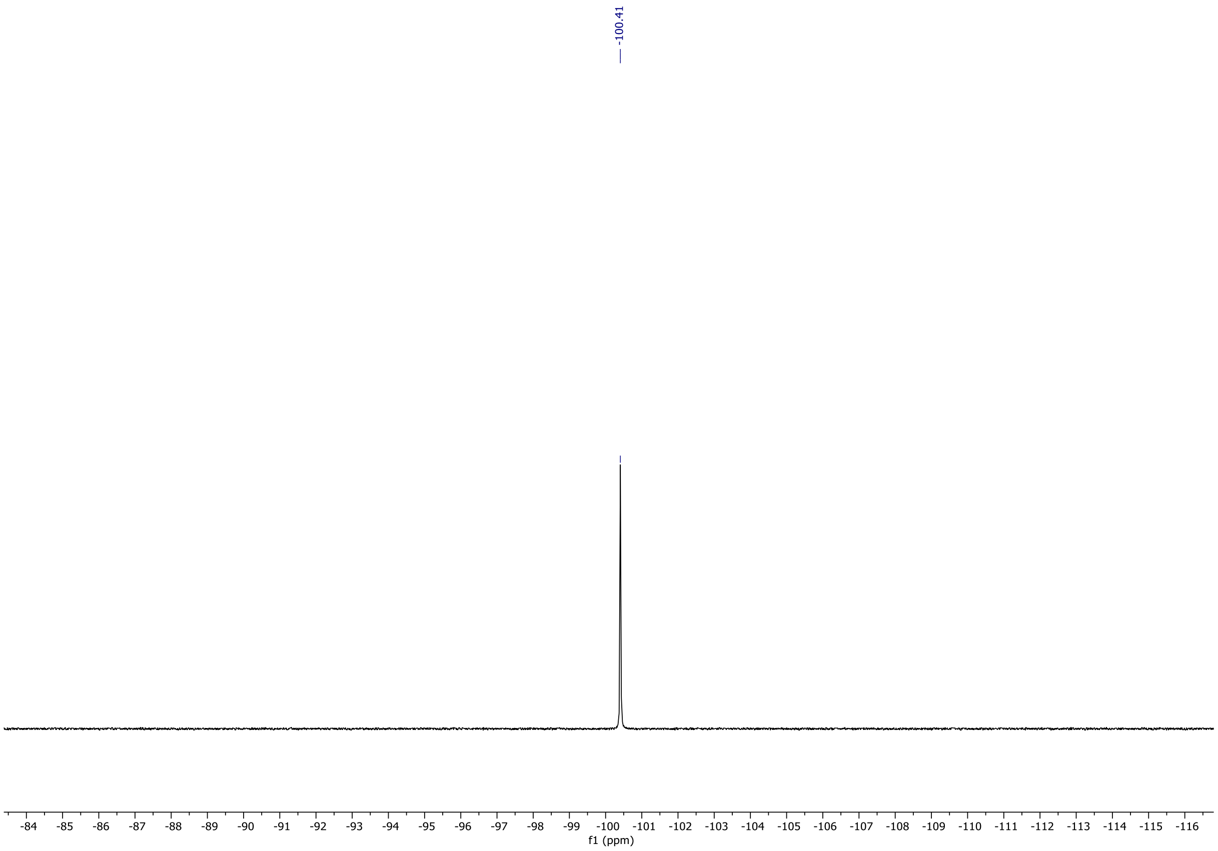

Figure S15. ^{19}F NMR (282 MHz, $CDCl_3$) of **10.**

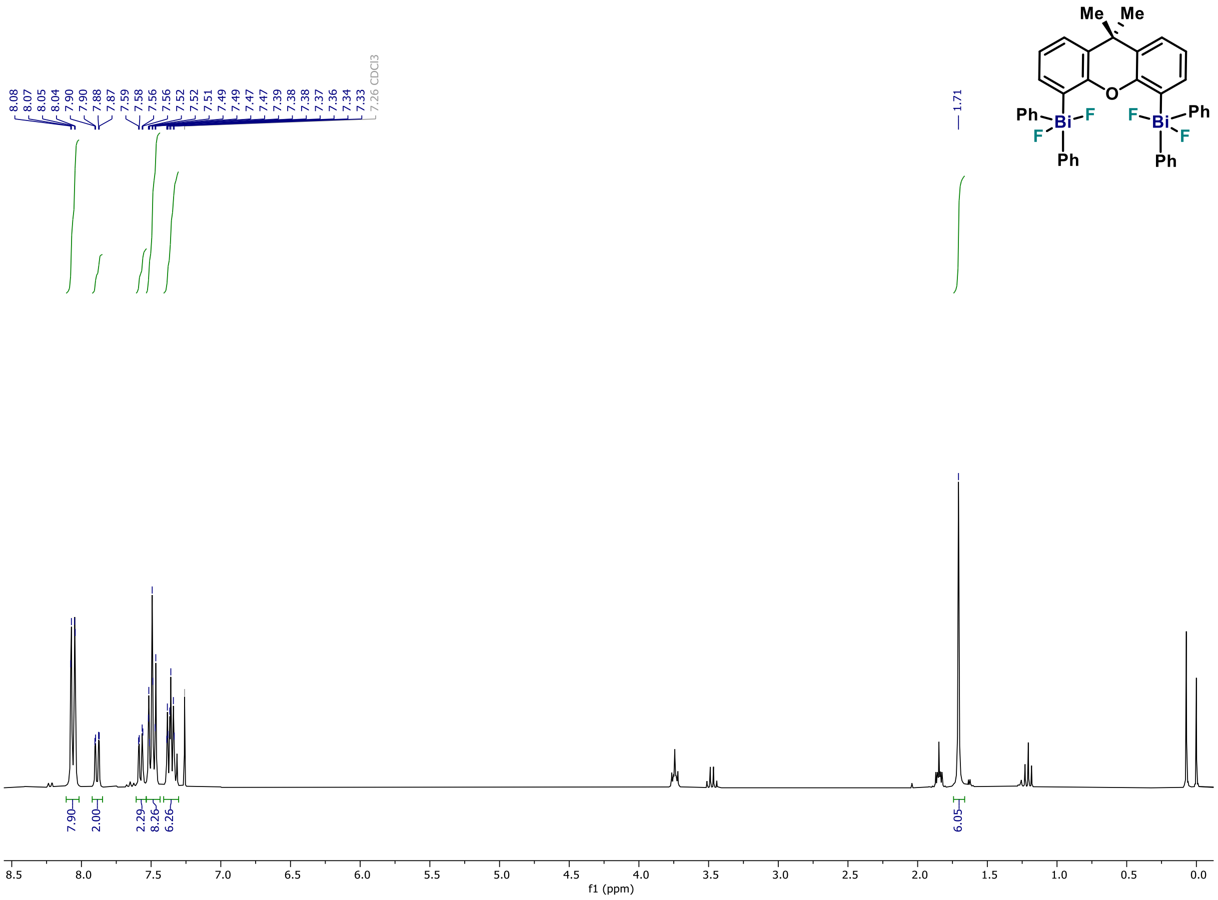

Figure S16. ^{1}H NMR (300 MHz, $CDCl_3$) of **2.**

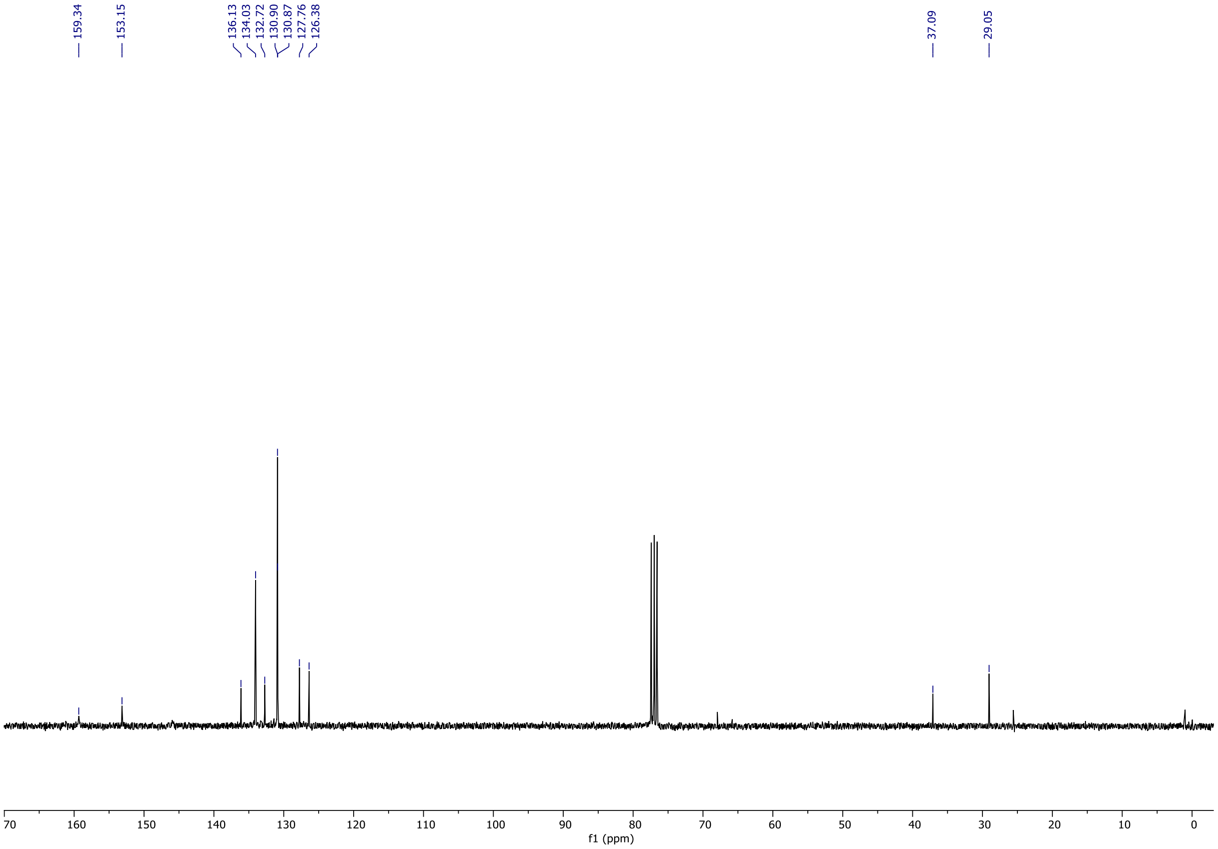

Figure S17. ^{13}C NMR (75 MHz, $CDCl_3$) of **2.**

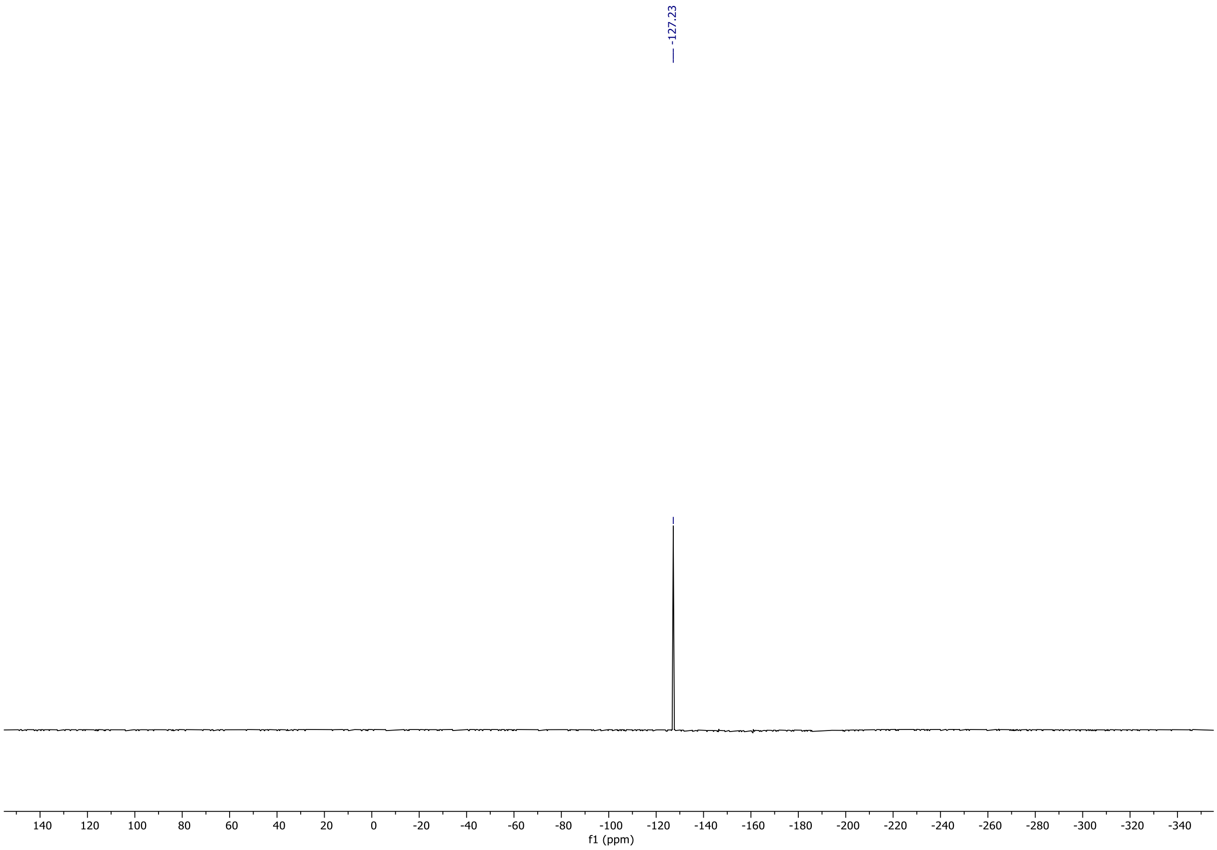

Figure S18. ^{19}F NMR (282 MHz, $CDCl_3$) of **2.**

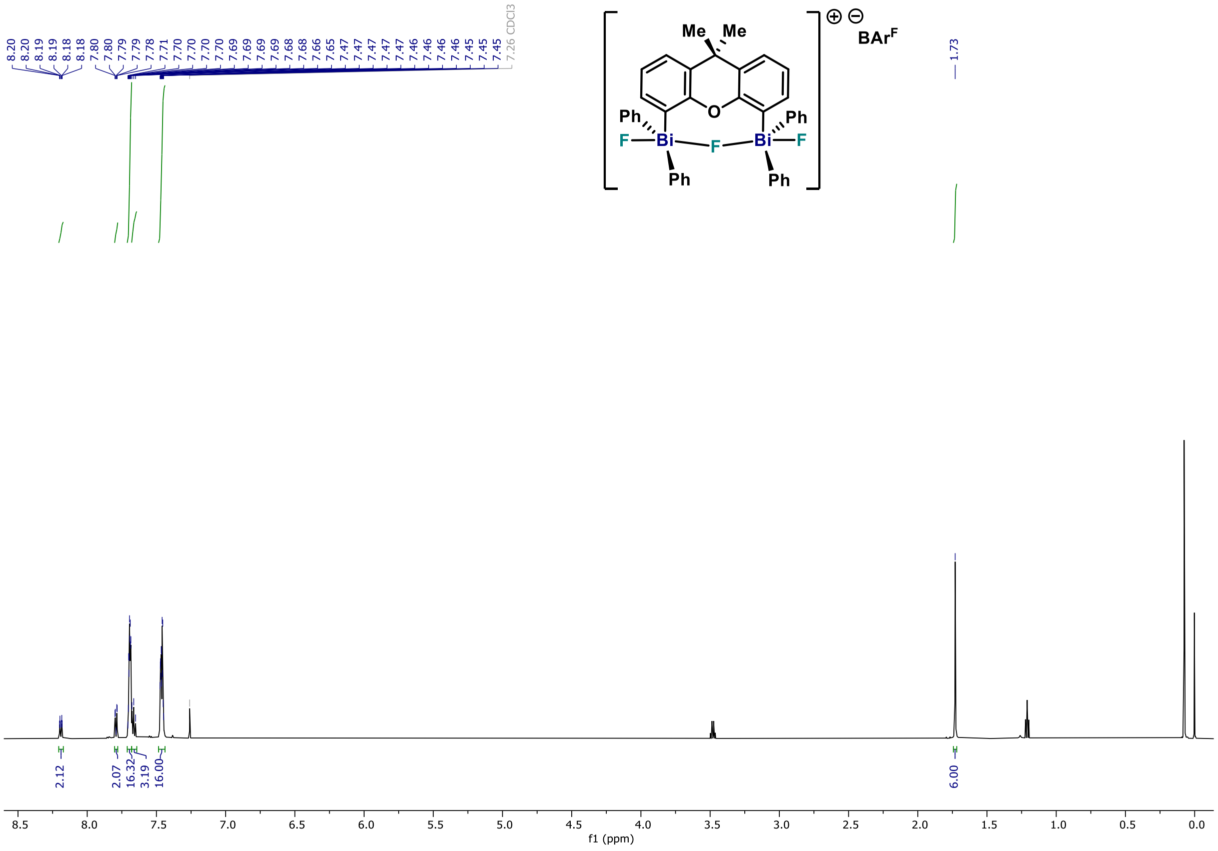

Figure S19. ^{1}H NMR (600 MHz, $CDCl_3$) of **3.**

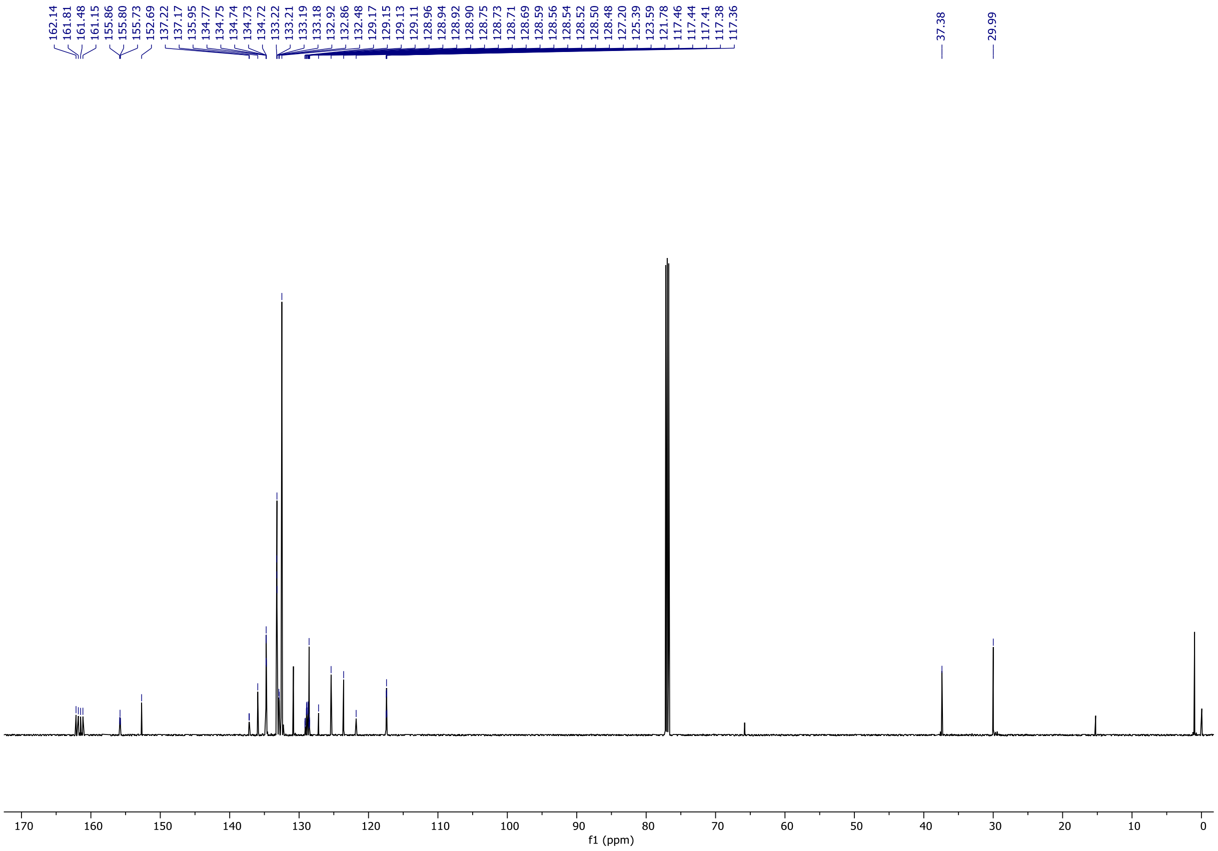

Figure S20. ^{13}C NMR (151 MHz, $CDCl_3$) of **3.**

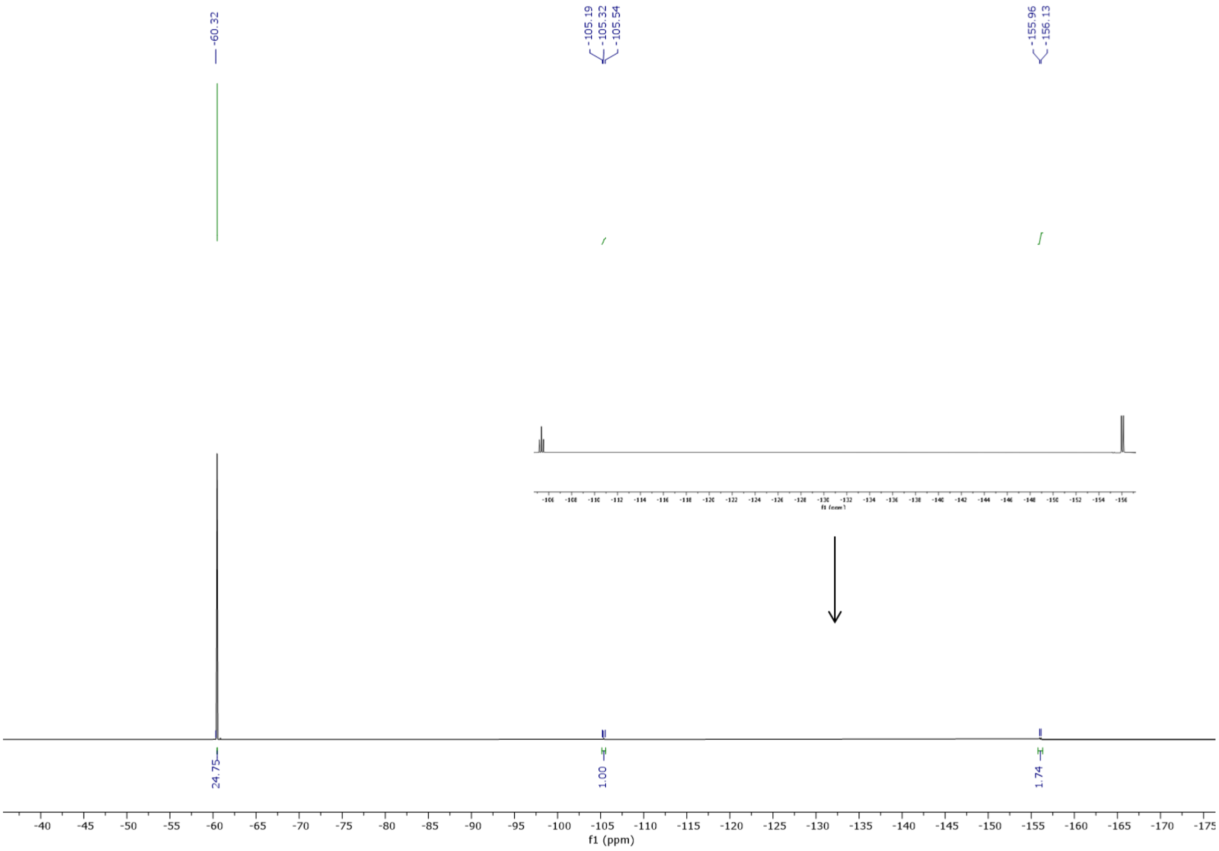

Figure S21. ^{19}F NMR (565 MHz, $CDCl_3$) of **3.**

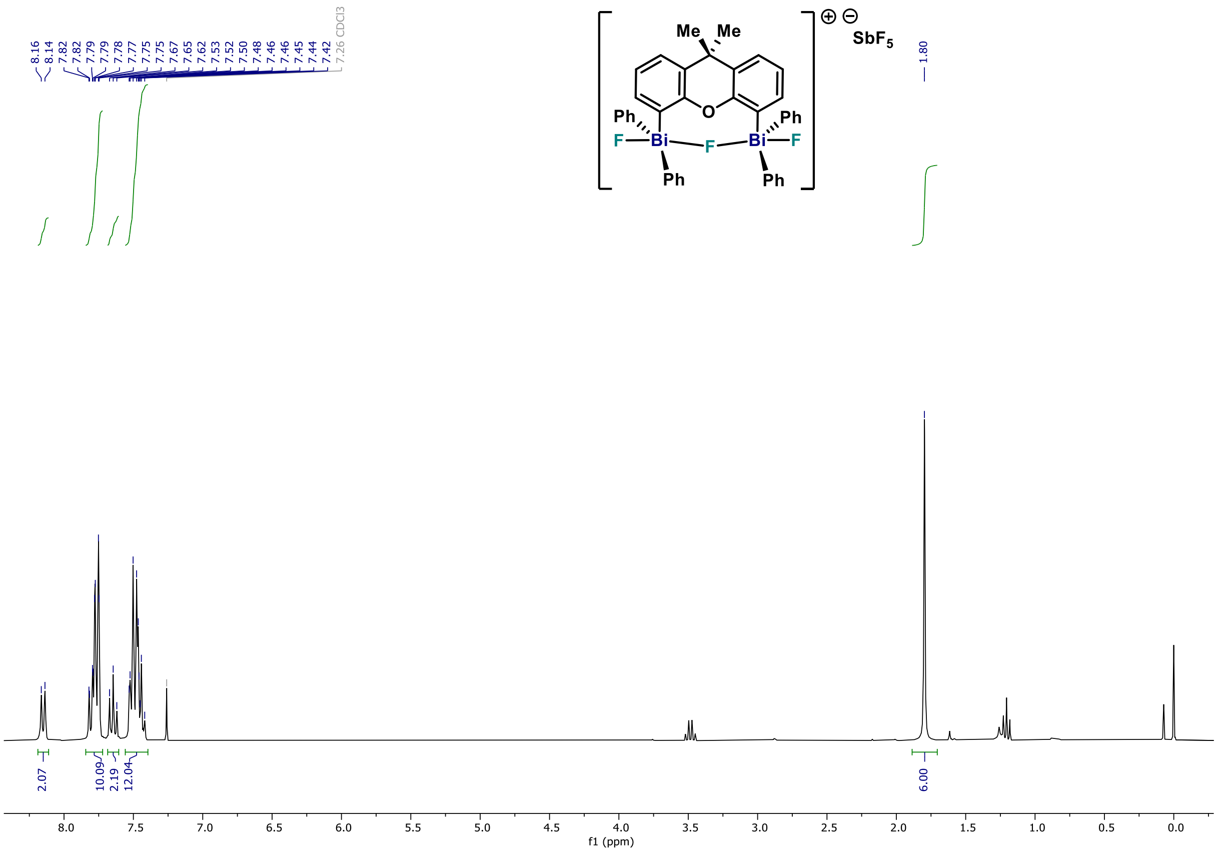

Figure S22. ^{1}H NMR (600 MHz, $CDCl_3$) of **4.**

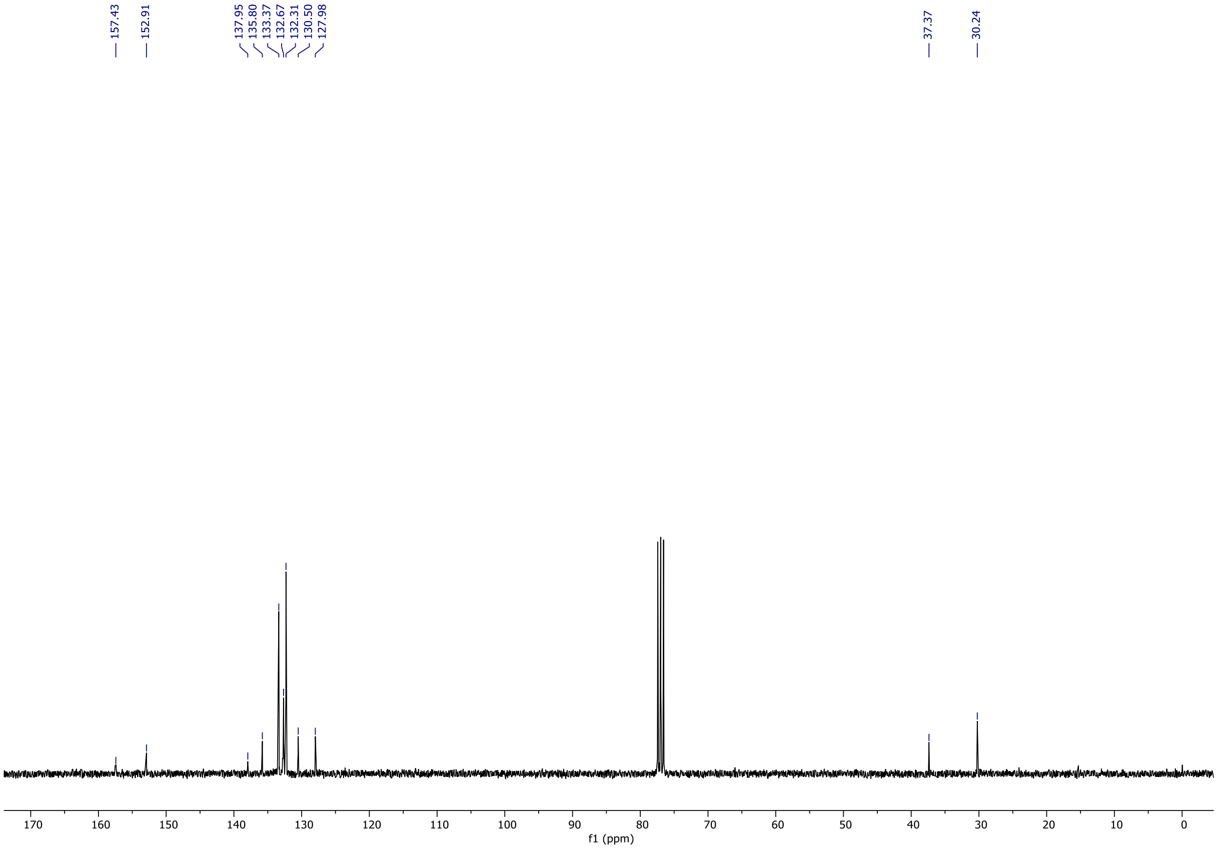

Figure S23. ^{13}C NMR (75 MHz, $CDCl_3$) of **4.**

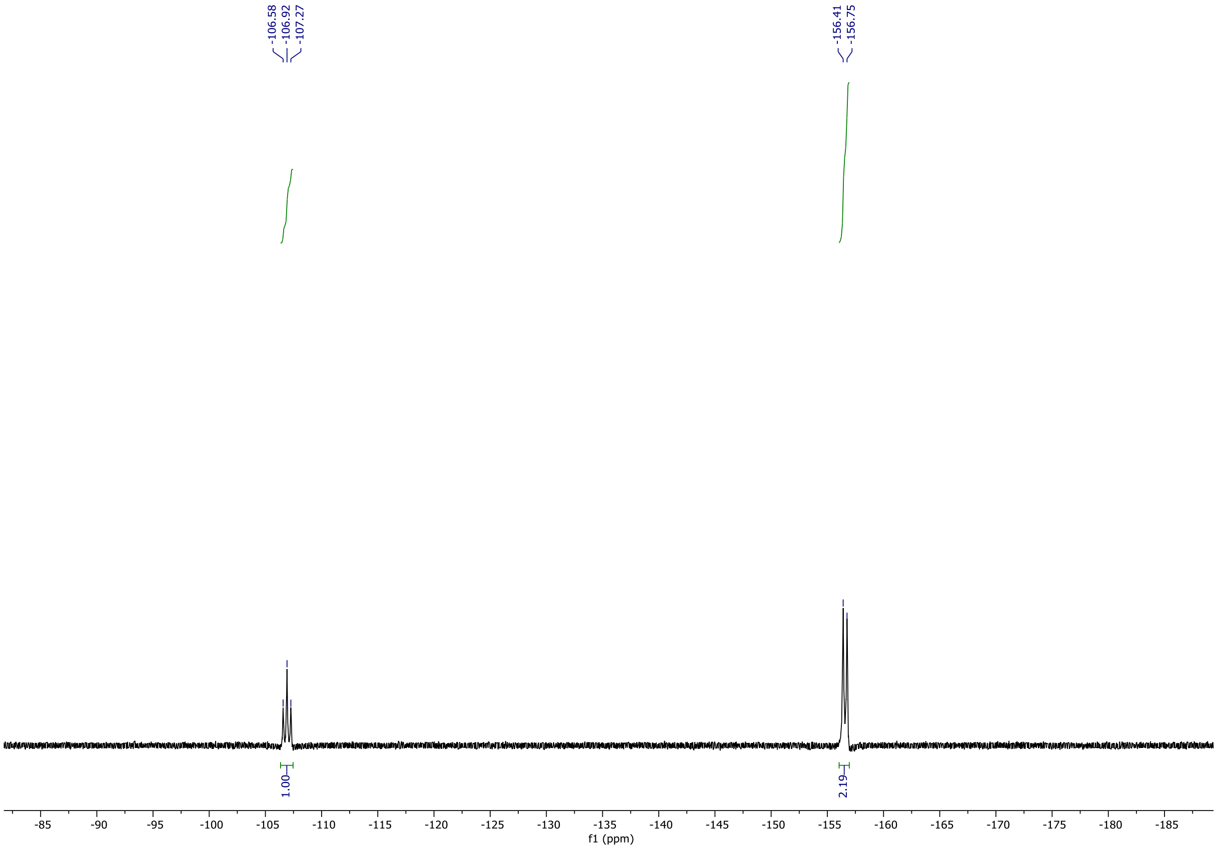

Figure S24. ^{19}F NMR (282 MHz, $CDCl_3$) of **4.**

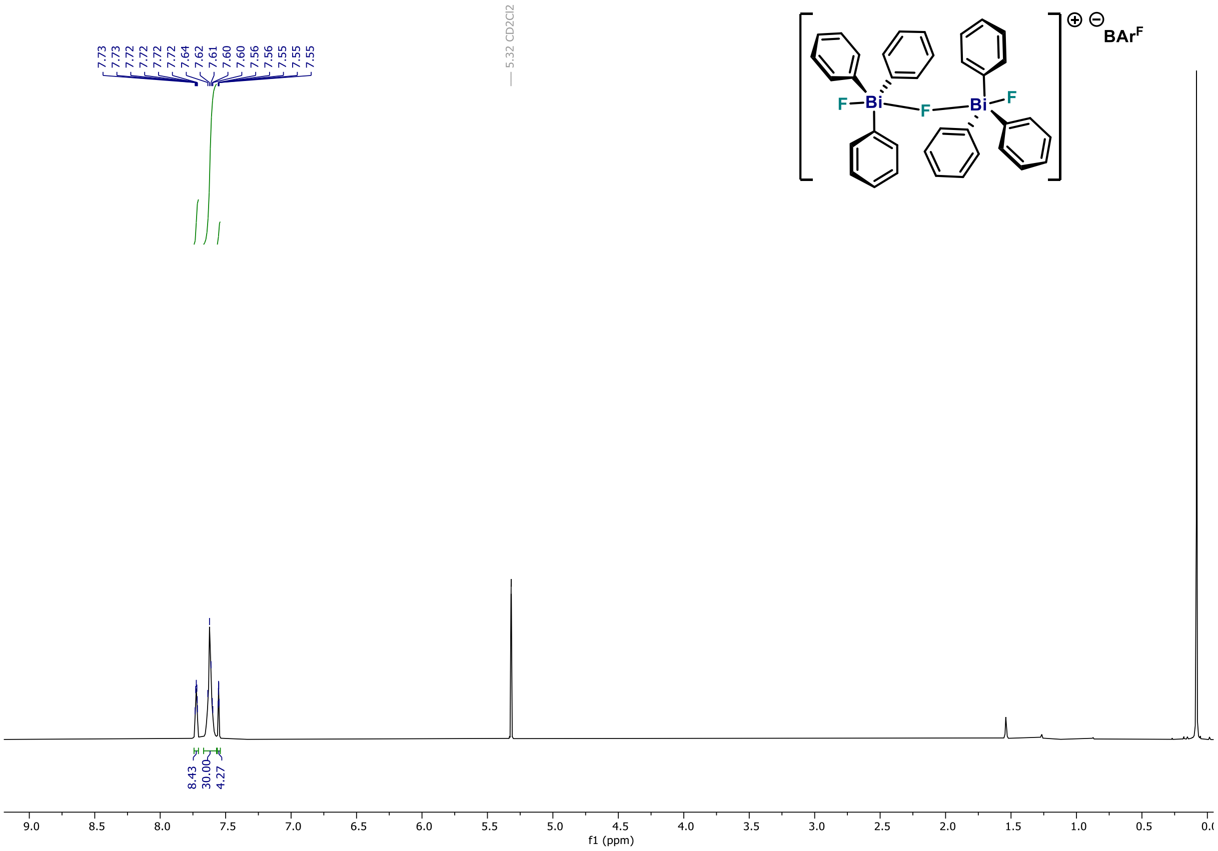

Figure S25. ^{1}H NMR (600 MHz, CD_2Cl_2) of **11** (dimer in solution **(15)**).

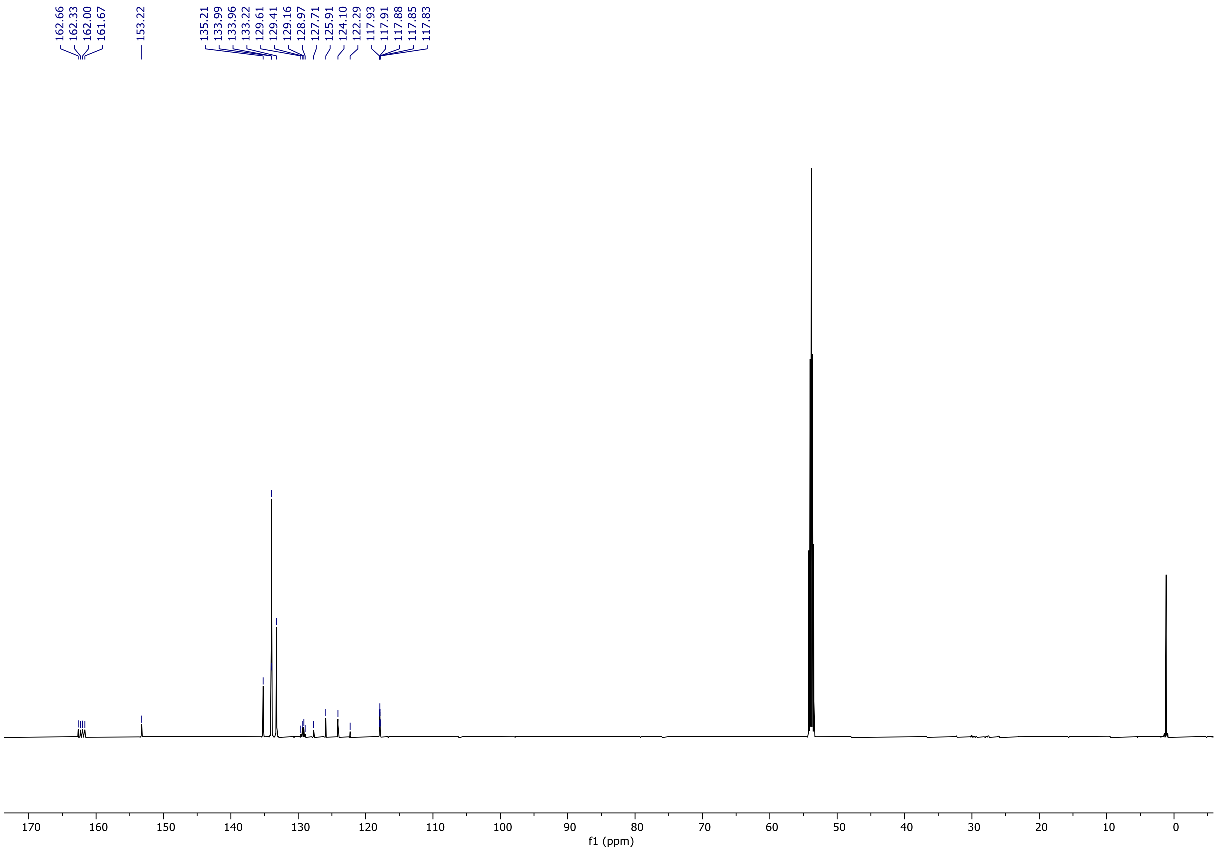

Figure S26. ^{13}C NMR (151 MHz, CD_2Cl_2) of **11** (dimer in solution **(15)**).

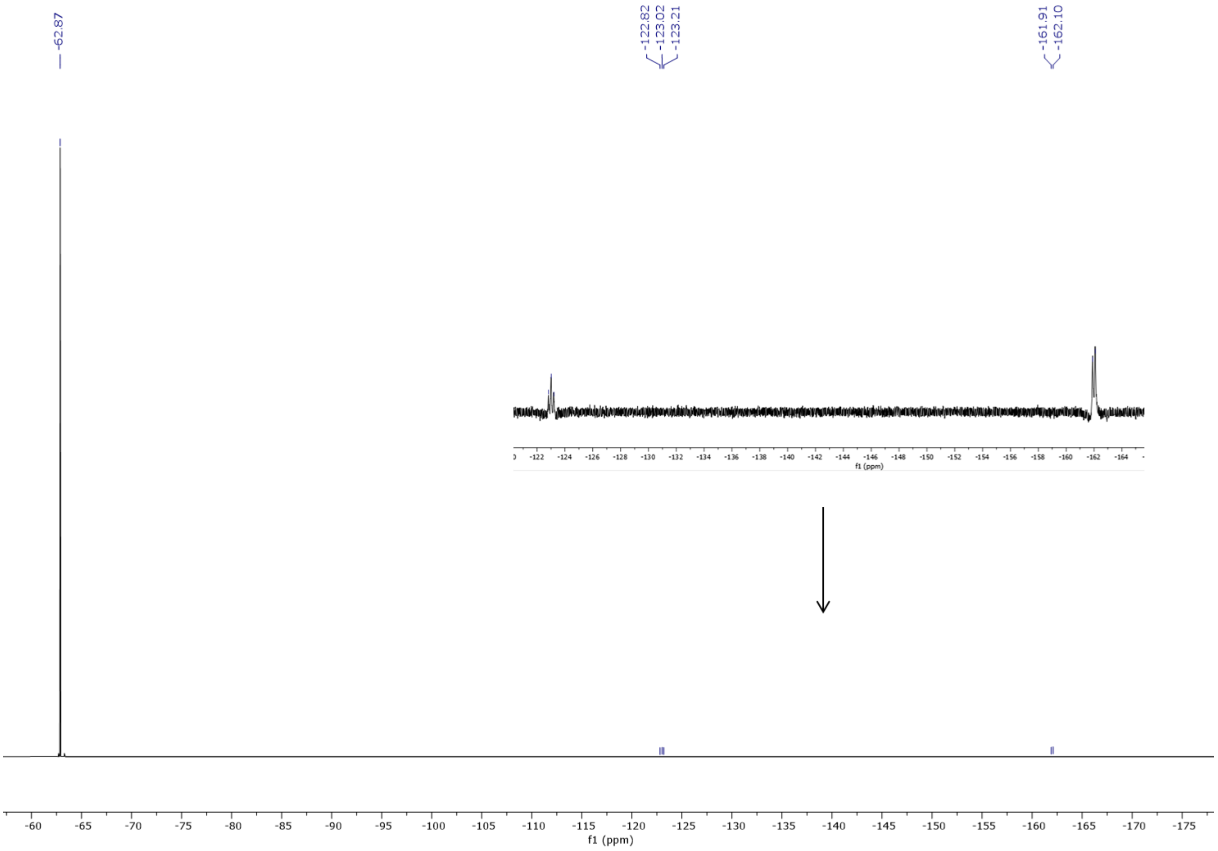

Figure S27. ^{19}F NMR (470 MHz, CD_2Cl_2) at 25 °C of **11** (dimer in solution **(15)**).

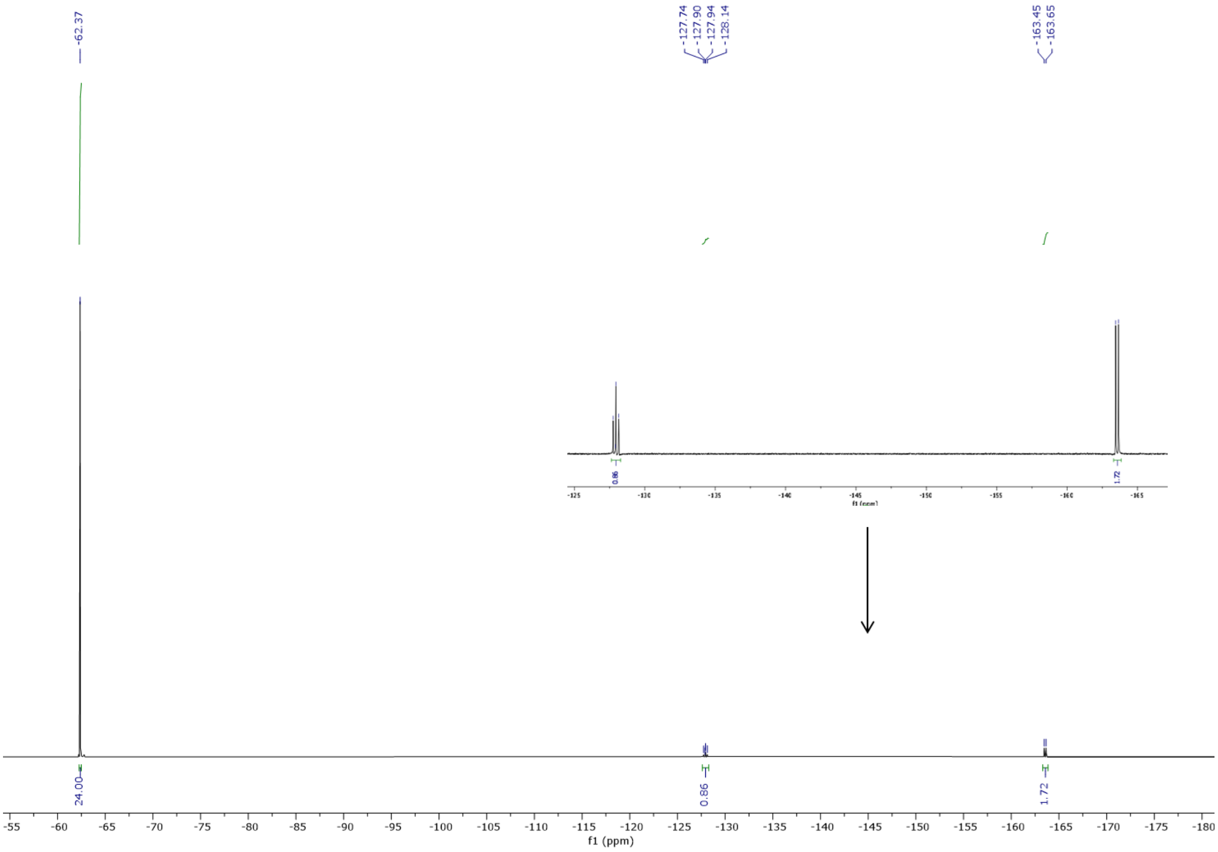

Figure S28. ^{19}F NMR (470 MHz, CD_2Cl_2) at -80 °C of **11** (dimer in solution (**15)**).

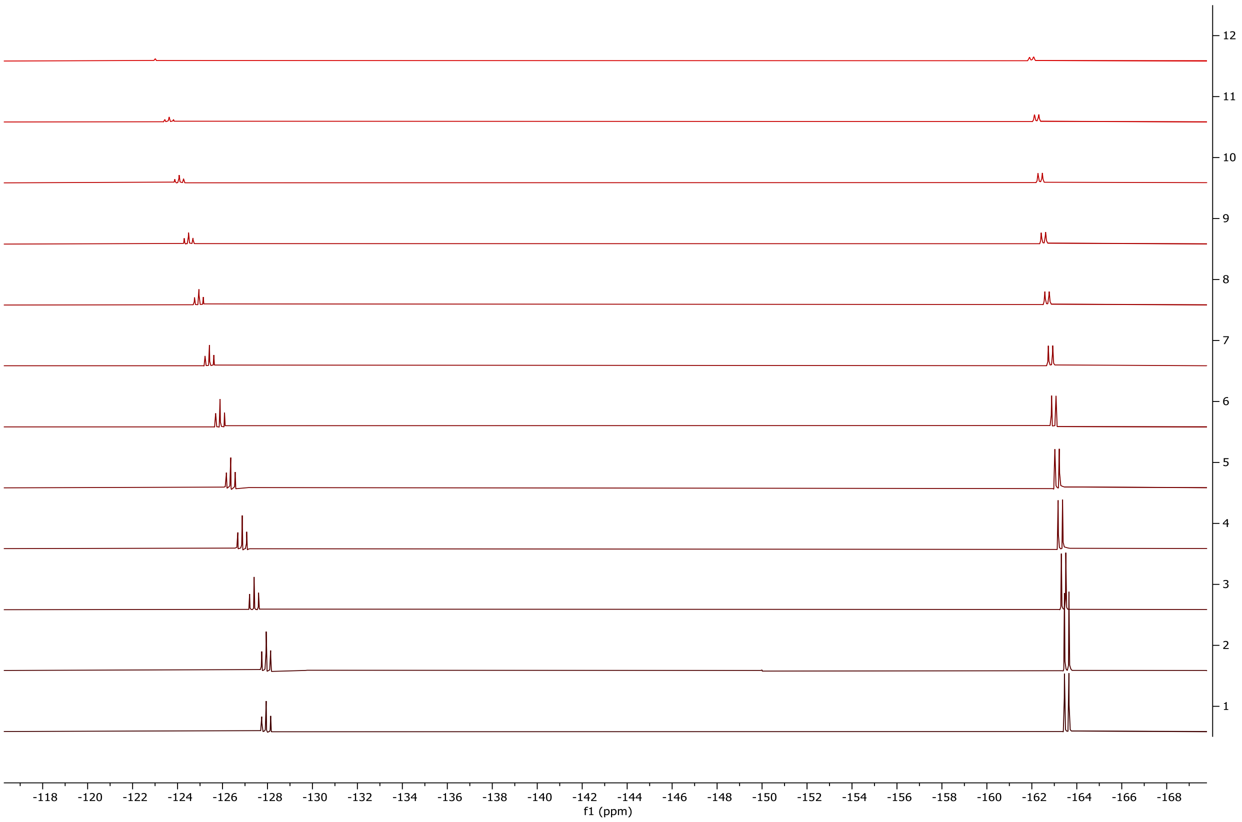

Figure S29. VT ^{19}F NMR (470 MHz, CD_2Cl_2) from -80°C (bottom) to 25 °C (top) of **11** (dimer in solution **(15)**).

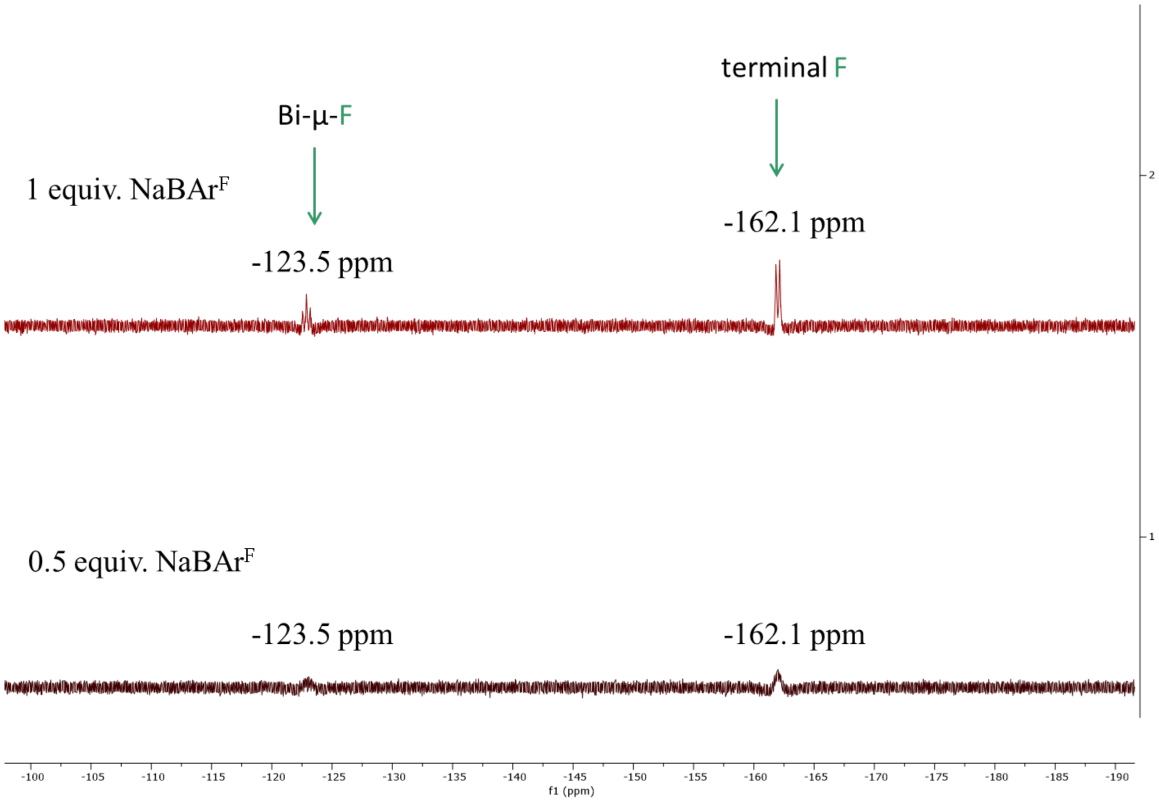

Figure S30. ^{19}F NMR (282 MHz, CD_2Cl_2) of Fluorobismuthonium **15** using 1.0 and 0.5 equiv. $NaBAr^F$

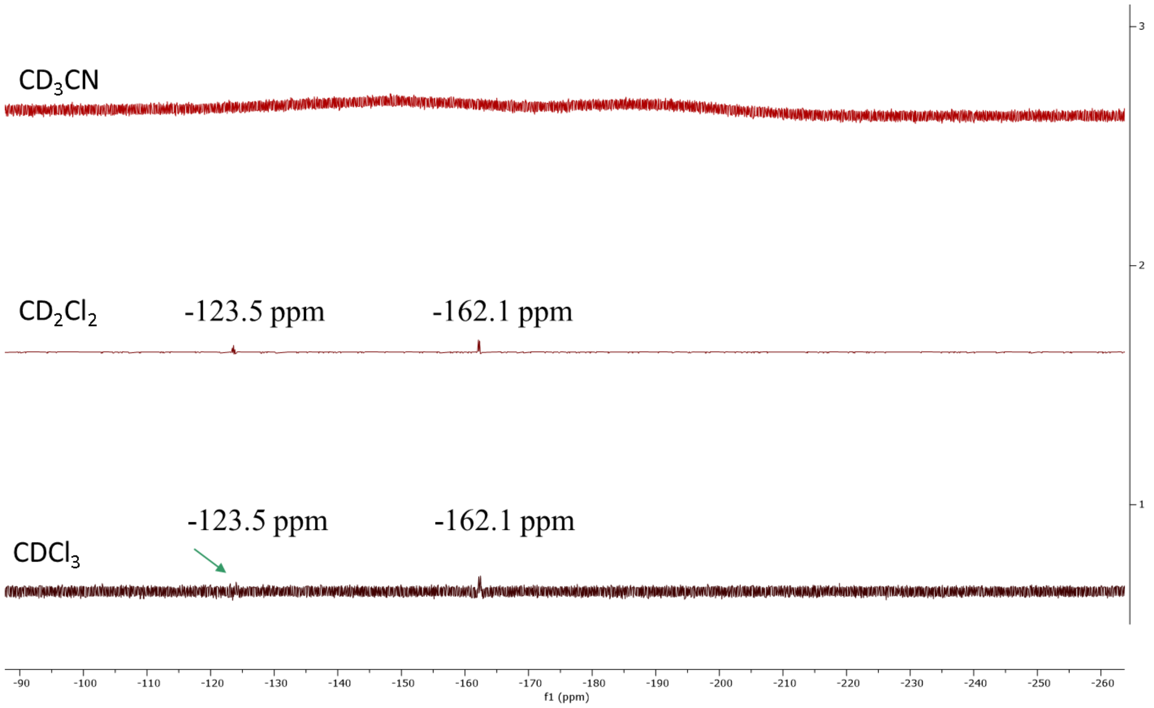

Figure S31. ^{19}F NMR (282 MHz) of Fluorobismuthonium **15** in different deuterated solvents.

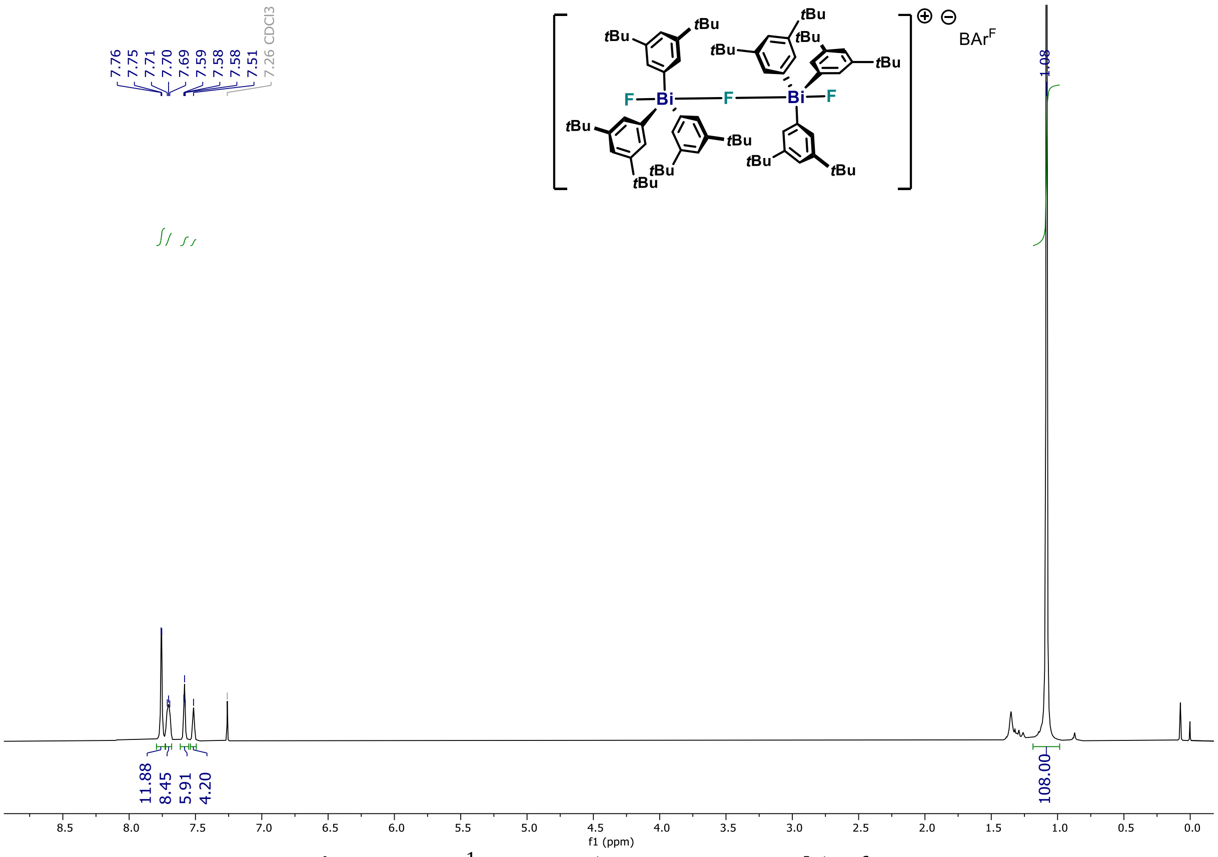

Figure S32. ^{1}H NMR (600 MHz, $CDCl_3$) of **12.**

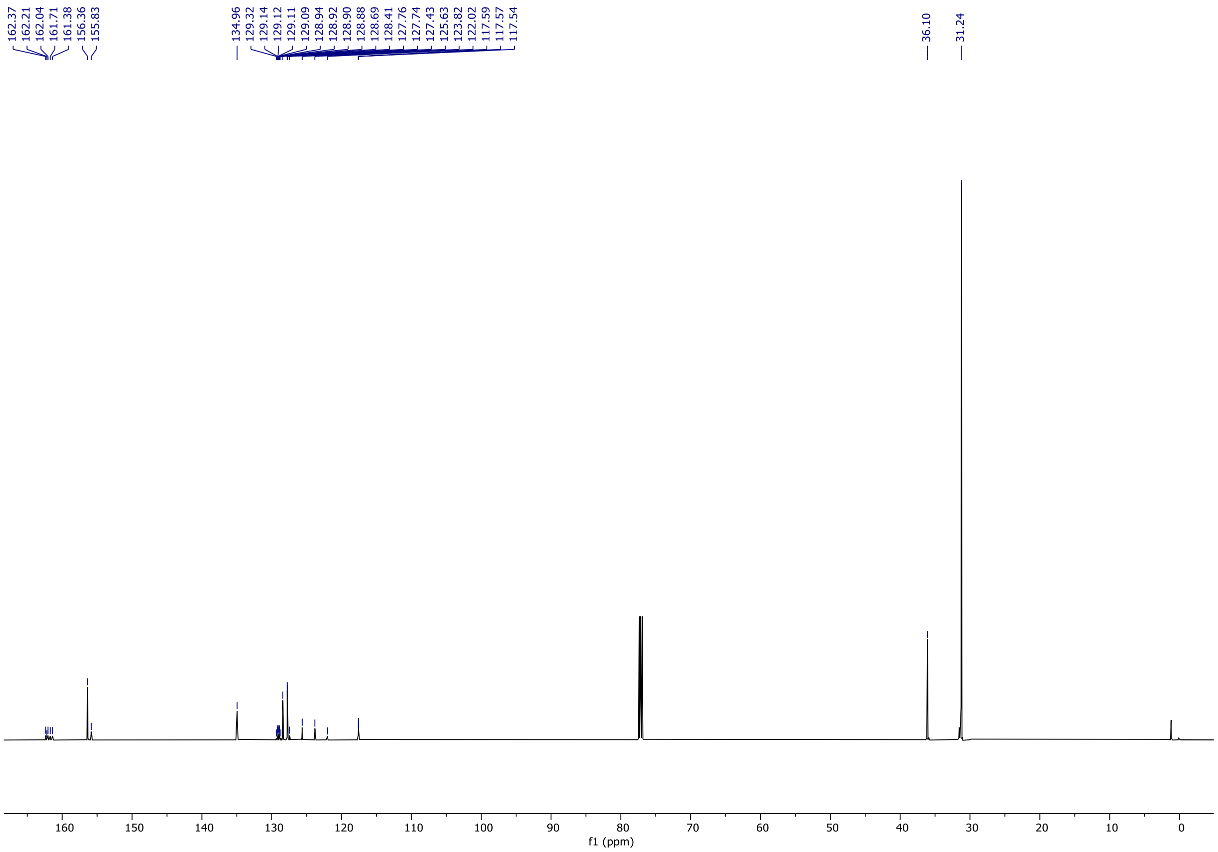

Figure S33. ^{13}C NMR (151 MHz, $CDCl_3$) of **12.**

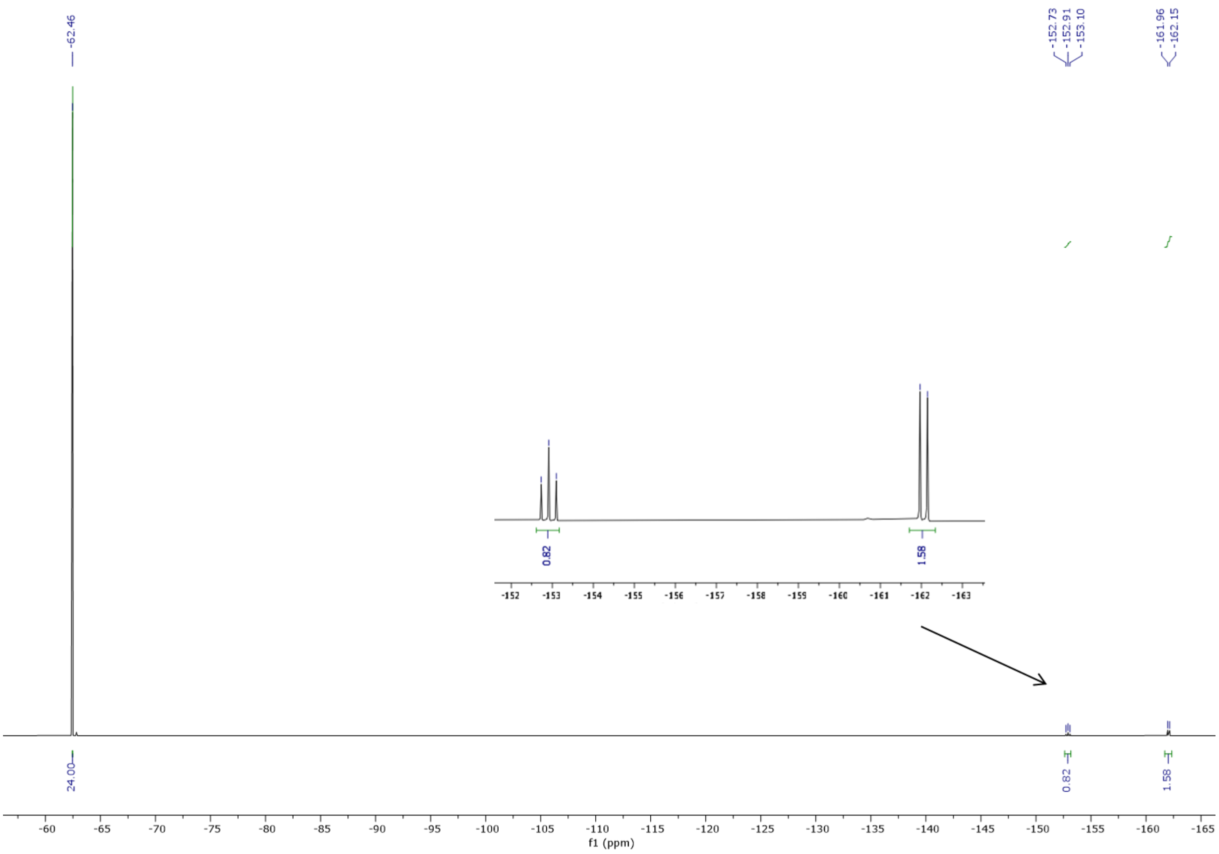

Figure S34. ^{19}F NMR (282MHz, $CDCl_3$) of **12.**

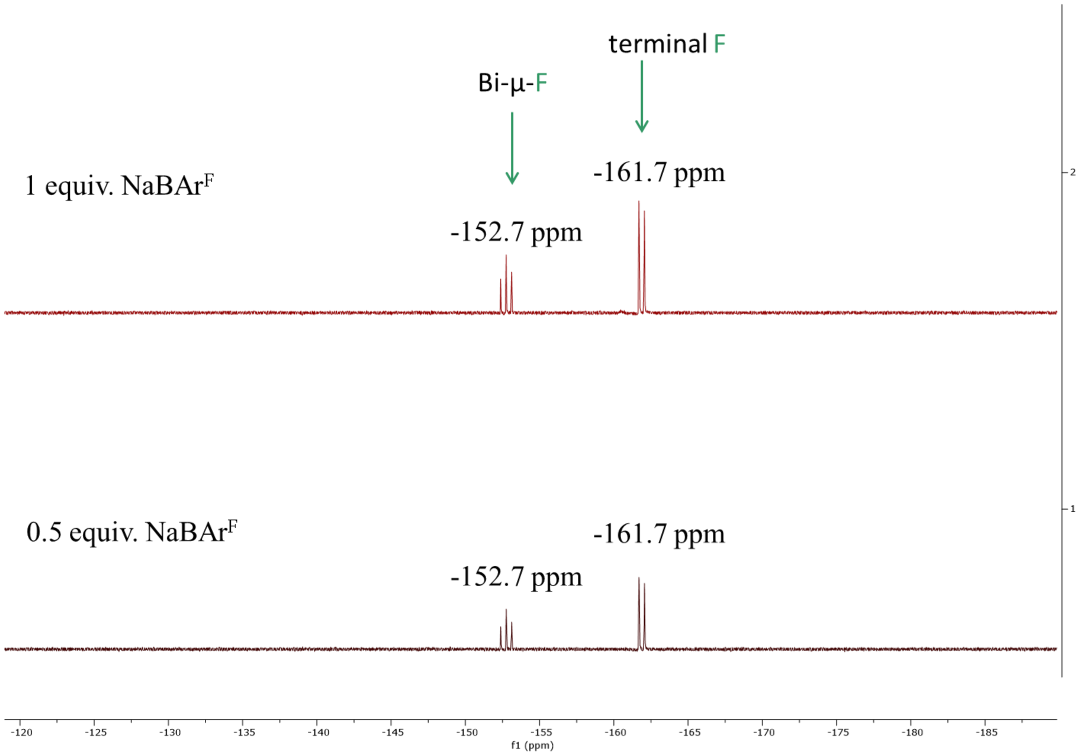

Figure S35. ^{19}F NMR (282 MHz, CD_2Cl_2) of Fluorobismuthonium **12** using 1.0 and 0.5 equiv. NaBAr^F.

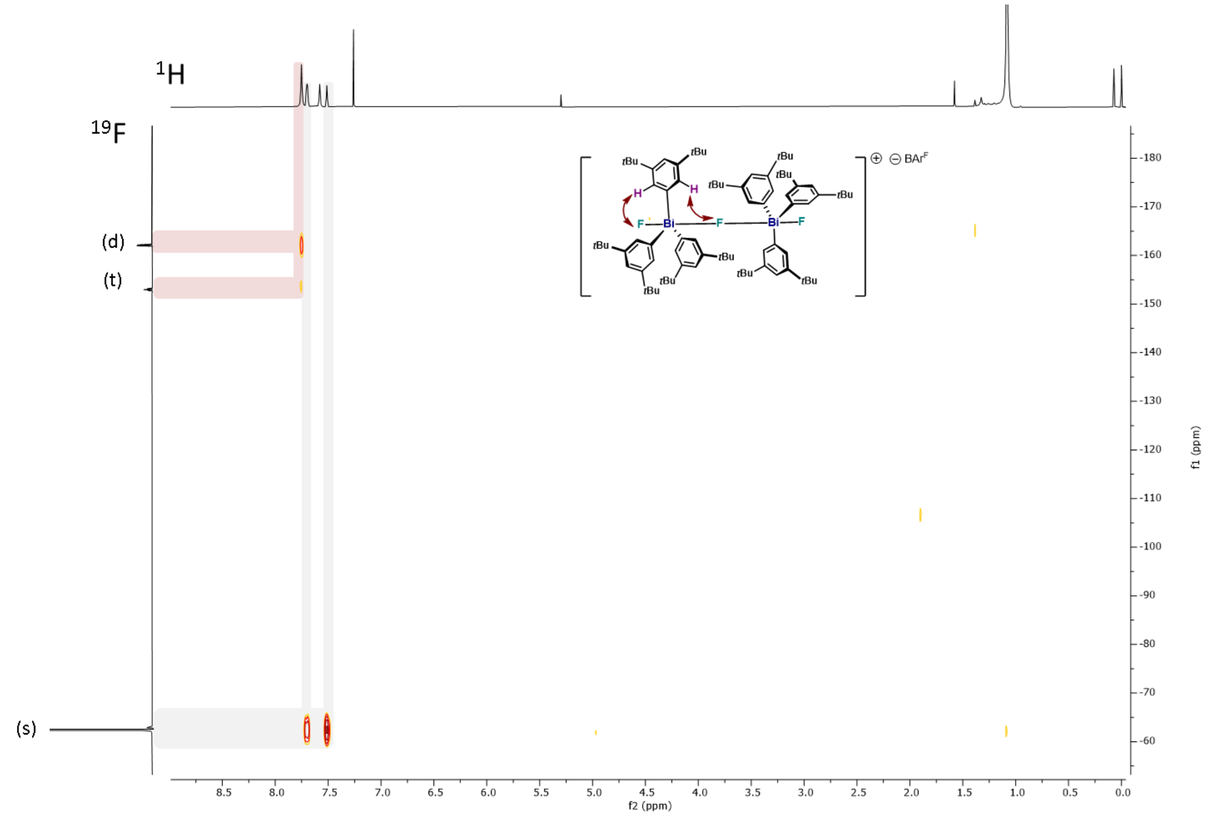

Figure S36. ^{1}H -^{19}F HOESY NMR (470 MHz, $CDCl_3$) of **12**. H···F correlation in fluorobismuthonium cation 12 is highlighted in red and H···F correlation in the counteranion BAr^F is highlighted in grey.

The correlation between the H atom in *ortho* position of the aryl group and the terminal F atom can be observed (highlighted in red). In addition, a correlation between the CF_3 substituents and the H in *ortho* and *para* position of the BAr^F anion are clearly visible (highlighted in grey).

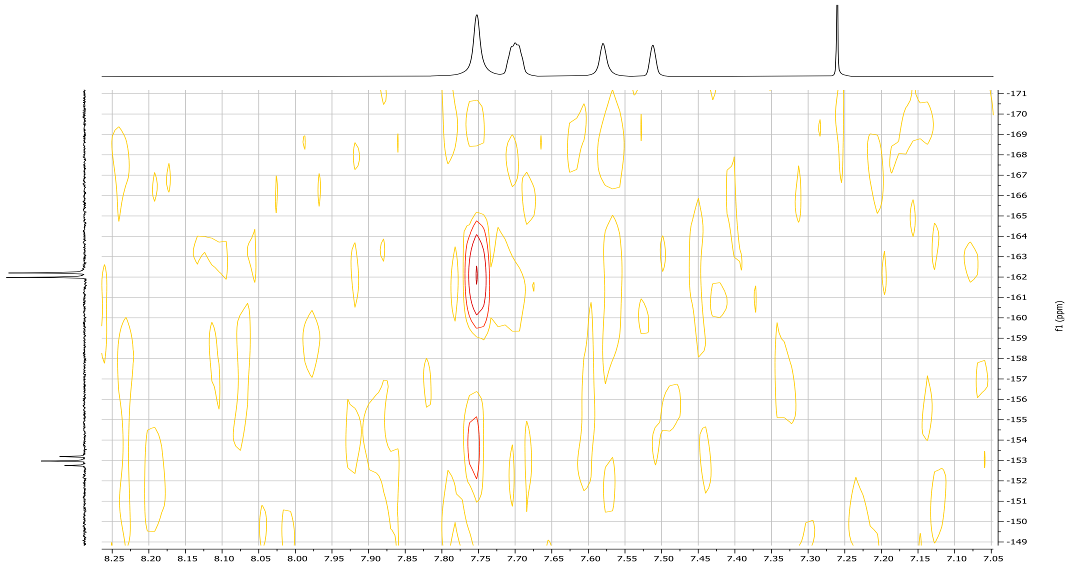

Figure S37. ^{1}H -^{19}F HOESY NMR (470 MHz, $CDCl_3$) of **12**.
Zoom area: (7.05 to 8.25 ppm / –149 to –171 ppm).

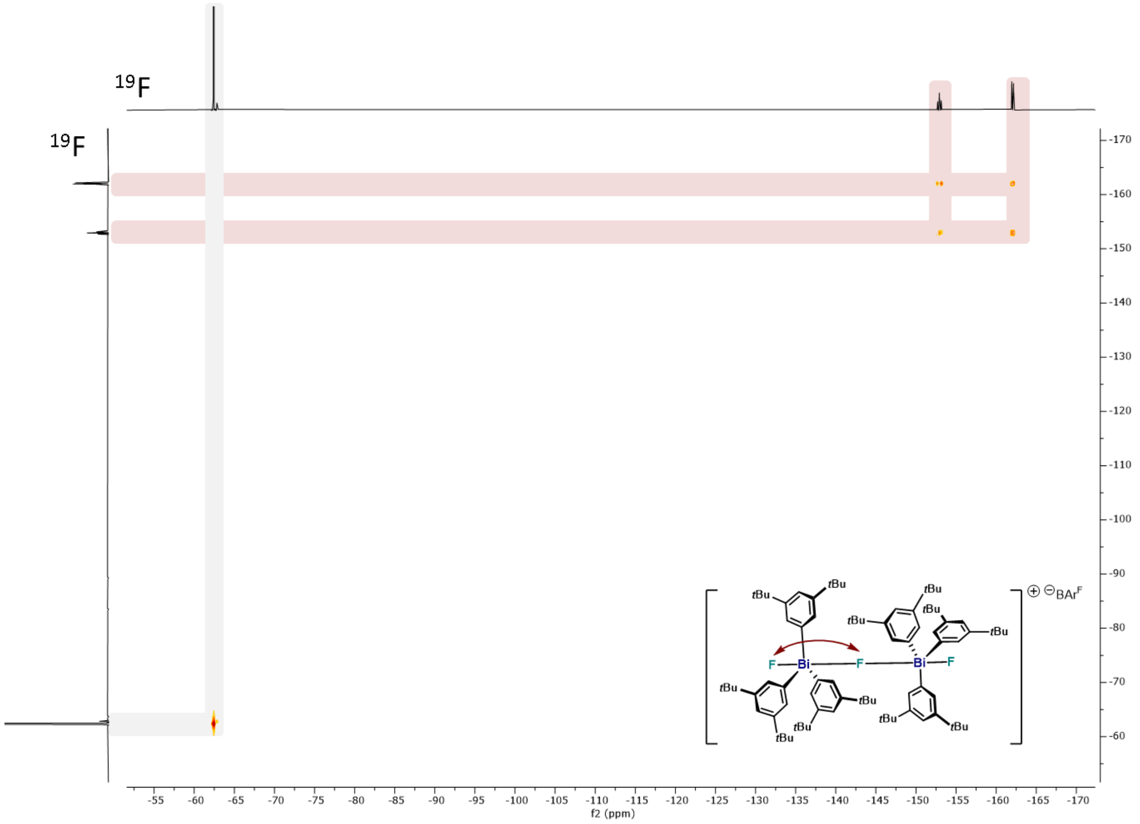

Figure S38. ^{19}F-^{19}F COSY NMR (470 MHz, $CDCl_3$) of **12.** F···F correlation in Fluorobismuthonium cation **12** (highlighted in red) and counteranion BAr^F (highlighted in grey).

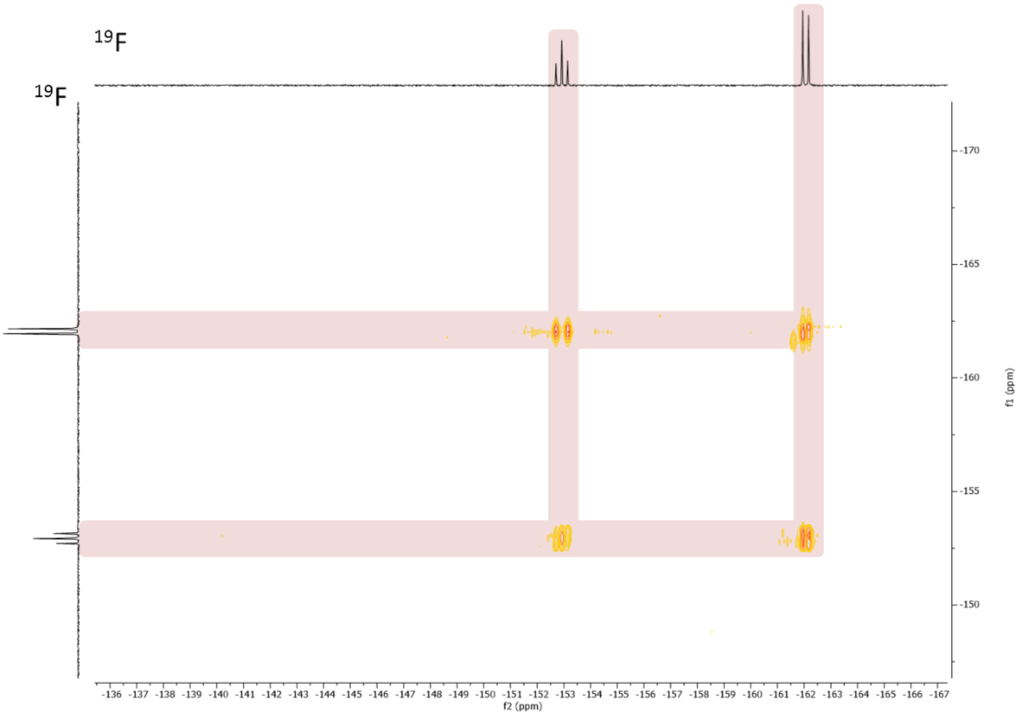

Figure S39. ^{19}F-^{19}F COSY NMR (470 MHz, $CDCl_3$) of **12**.
Zoom area: (–136 to –167 ppm / –145 to –173 ppm).

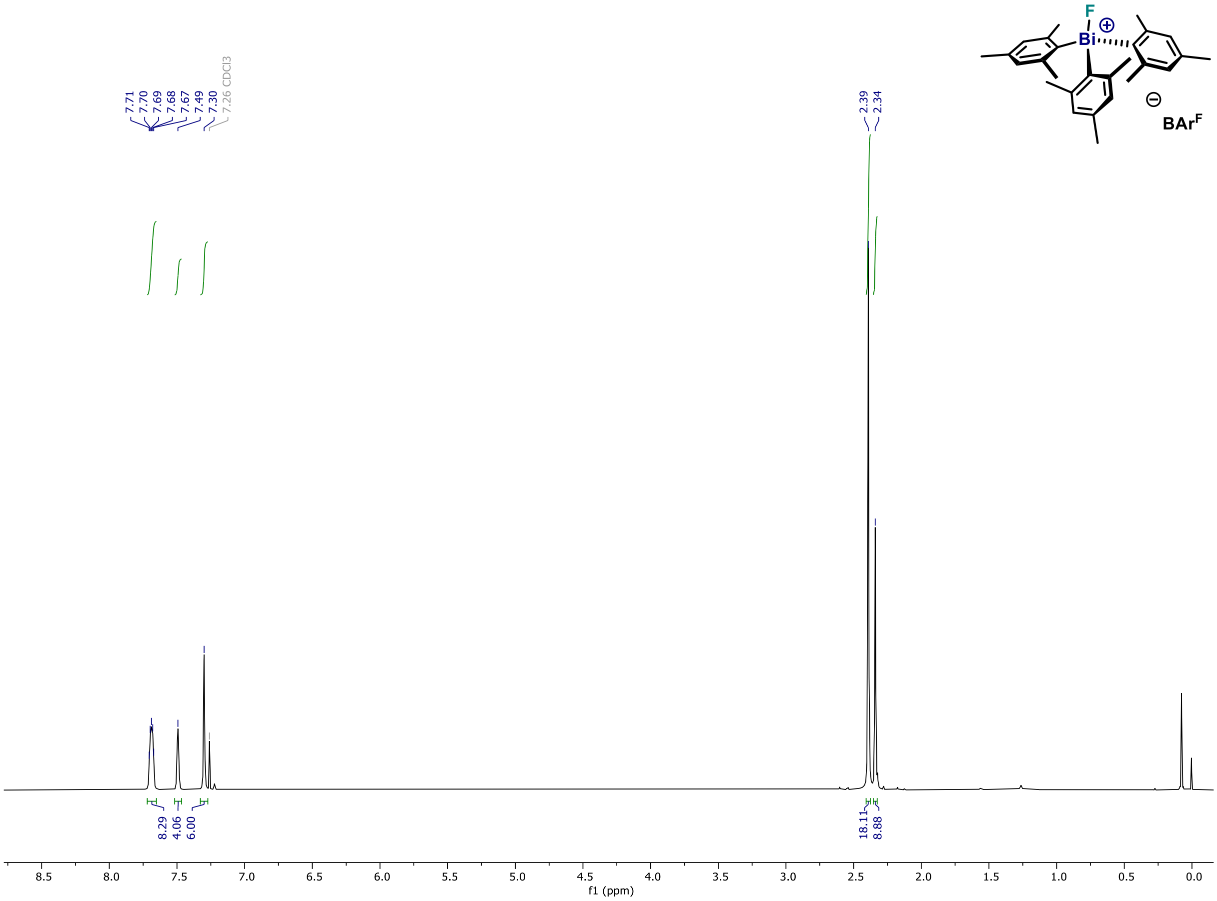

Figure S40. ^{1}H NMR (300 MHz, $CDCl_3$) of **13.**

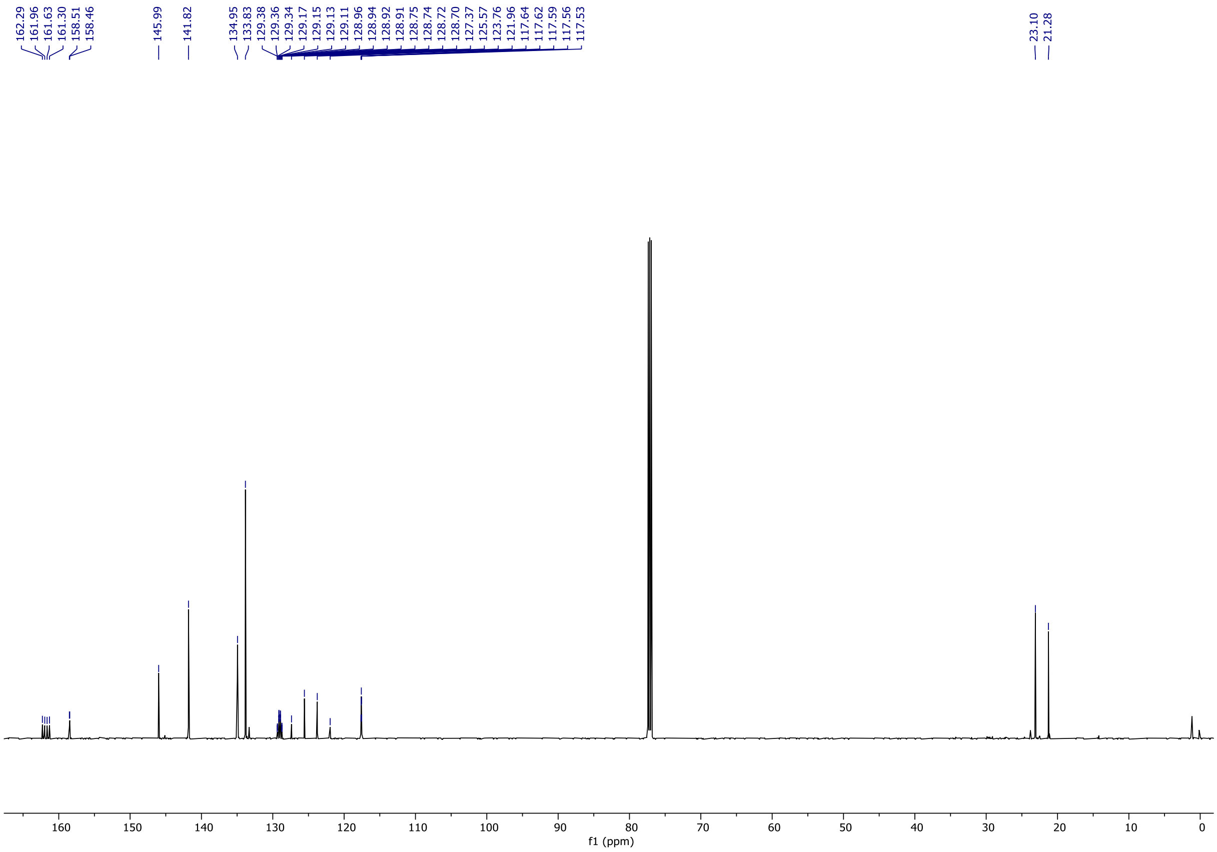

Figure S41. ^{13}C NMR (151 MHz, $CDCl_3$) of **13.**

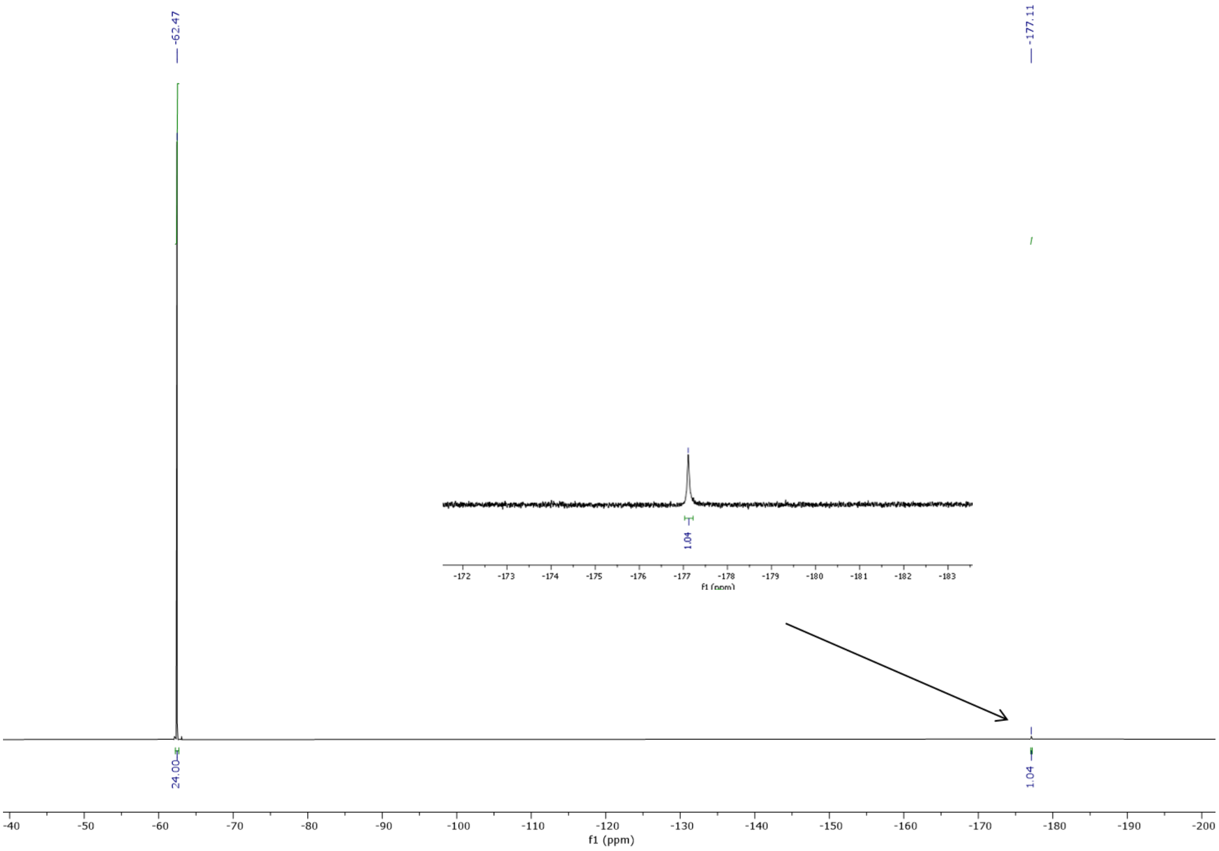

Figure S42. ^{19}F NMR (282 MHz, $CDCl_3$) of **13**.

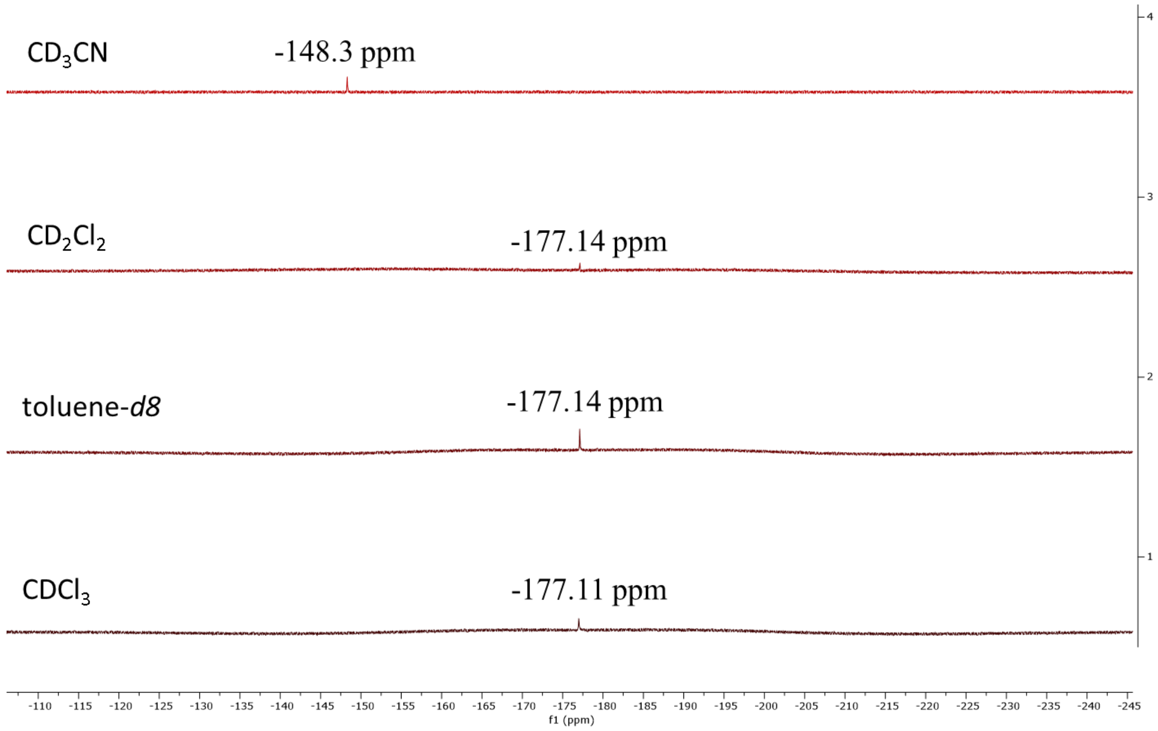

Figure S43. ^{19}F NMR (282 MHz) of Fluorobismuthonium **13** in different deuterated solvents.

6. X-ray single crystal analysis

Single crystal structure analysis of complex 3 (13153)

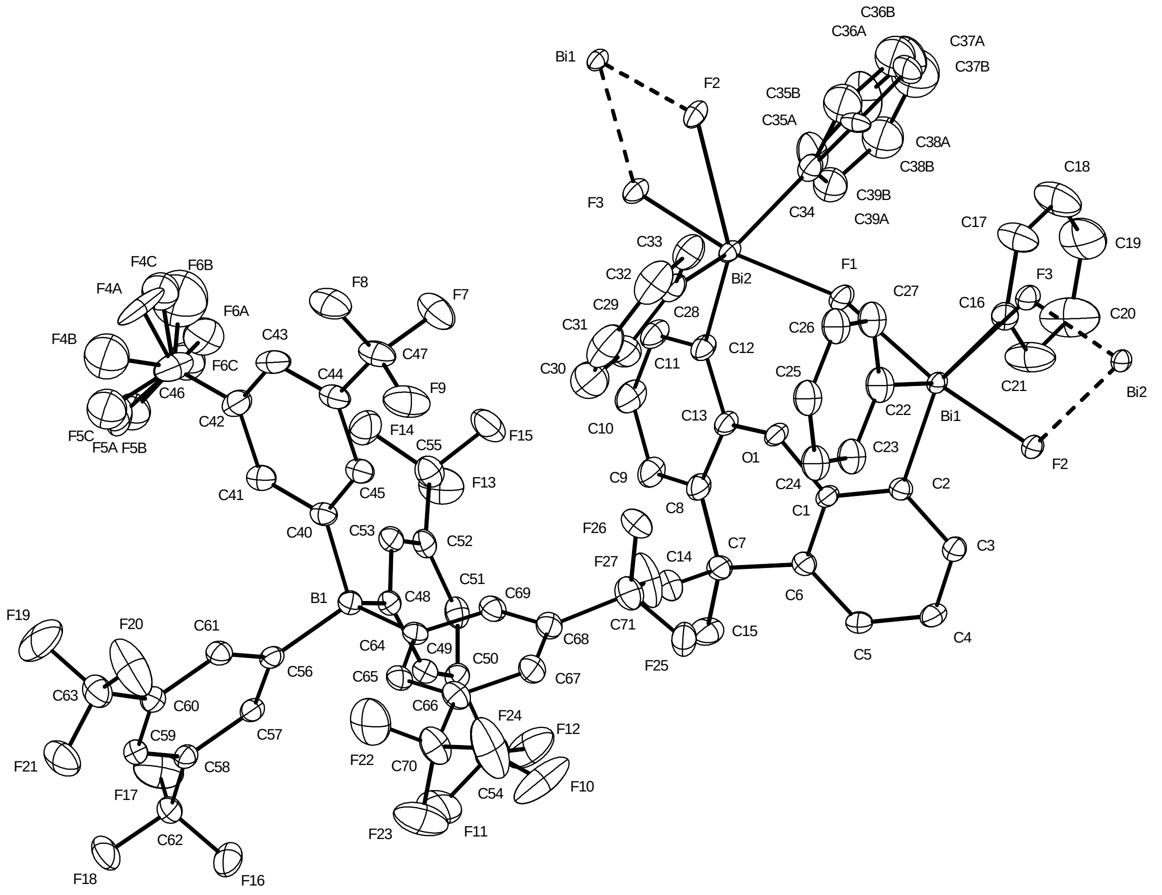

Figure S44. The molecular structure of complex **3**. H atoms have been removed for clarity. Dashed lines indicate polymeric extensions. Ellipsoids are drawn at the 50% probability level.

X-ray Crystal Structure Analysis of complex 3:
C_{71} H_{44} B Bi_2 F_{27} O, M_r = 1854.83 g mol^{-1}, colorless plate, crystal size 0.097 x 0.041 x 0.032 mm^3, monoclinic, space group $P2_1$/c [14], a = 17.2475(7) Å, b = 27.5458(10) Å, c = 15.4288(6) Å, β = 106.599(2)°, V = 7024.7(5) Å^3, T = 100(2) K, Z = 4, D_{calc} = 1.754 g·cm^3, λ = 0.71073 Å, $\mu(Mo\text{-}K_\alpha)$ = 5.118 mm^{-1}, Gaussian absorption correction ($T_{\min}$ = 0.71883, $T_{\max}$ = 0.90049), Bruker-AXS Kappa Mach3 with APEX-II detector and IµS microfocus Mo-anode X-ray source, 1.437 < θ < 30.507°, 230929 measured reflections, 21415 independent reflections, 17912 reflections with $I > 2\sigma(I)$, R_{int} = 0.0519. The structure was solved by *SHELXT* and refined by full-matrix least-squares (*SHELXL*) against F^2 to R_1 = 0.0273 [$I > 2\sigma(I)$], wR_2 = 0.0612 [all data], 911 parameters and 9 restraints.

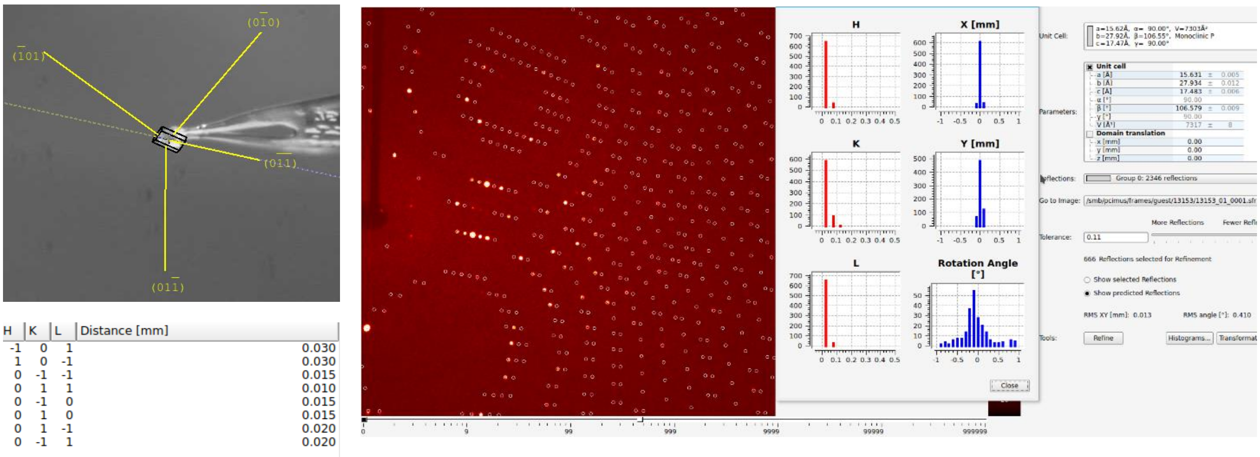

Figure S45. Crystal faces and unit cell determination/refinement of complex **3**.

INTENSITY STATISTICS FOR DATASET

Resolution	#Data	#Theory	%Complete	Redundancy	Mean I	Mean I/s	Rmerge	Rsigma
Inf - 2.44	541	541	100.0	17.70	43.32	59.92	0.0399	0.0134
2.44 - 1.62	1281	1281	100.0	18.58	30.15	56.04	0.0302	0.0136
1.62 - 1.28	1821	1821	100.0	18.53	19.20	48.63	0.0334	0.0147
1.28 - 1.12	1768	1768	100.0	17.69	13.24	41.23	0.0432	0.0167
1.12 - 1.01	1935	1935	100.0	13.29	11.47	31.10	0.0556	0.0233
1.01 - 0.94	1752	1752	100.0	10.17	9.77	24.46	0.0667	0.0298
0.94 - 0.88	1972	1972	100.0	8.51	7.62	18.84	0.0803	0.0390
0.88 - 0.84	1656	1656	100.0	7.78	6.47	15.66	0.0946	0.0478
0.84 - 0.80	2004	2004	100.0	7.40	5.41	13.33	0.1091	0.0570
0.80 - 0.77	1721	1721	100.0	7.06	4.99	11.94	0.1213	0.0644
0.77 - 0.74	2090	2090	100.0	6.72	4.43	10.46	0.1392	0.0755
0.74 - 0.72	1569	1569	100.0	6.43	4.21	9.50	0.1546	0.0838
0.72 - 0.70	1750	1750	100.0	6.25	3.81	8.37	0.1726	0.0950
0.70 - 0.68	1998	1998	100.0	5.99	3.15	7.12	0.1972	0.1168
0.68 - 0.66	2228	2228	100.0	5.72	2.96	6.43	0.2222	0.1316
0.66 - 0.65	1200	1200	100.0	5.57	2.53	5.45	0.2592	0.1576
0.65 - 0.63	2655	2655	100.0	5.35	2.47	5.11	0.2751	0.1701
0.63 - 0.62	1461	1463	99.9	5.14	2.19	4.47	0.3069	0.2002
0.62 - 0.61	1566	1573	99.6	5.01	2.11	4.17	0.3252	0.2140
0.61 - 0.60	1661	1673	99.3	4.79	1.77	3.45	0.3703	0.2706
0.60 - 0.59	1318	1971	66.9	1.59	1.21	1.61	0.4492	0.6821
0.69 - 0.59	13131	13805	95.1	4.81	2.31	4.75	0.2746	0.2004
Inf - 0.59	35947	36621	98.2	8.35	7.26	16.69	0.0621	0.0512

Sixteen reflections show high I/σI and have been omitted from data set before final refinement cycles. A resolution cut-off (SHEL 99 0.7) was applied to exclude poor determined intensities at higher diffraction angles. A phenyl ligand attached to Bi2 is disordered over two positions with an occupancy of 60:40%. Another phenyl ligand attached to Bi1 shows slight disorder with less occupancy as well. This could not be resolved in an accurate way, therefore atoms where fixed with 100% occupancy and thermal ellipsoids are equalized using EADP. One terminal CF_3 group in the anionic entity shows a rotational disorder over three positions with

occupancies of 60:20:20%. DFIX instructions were used to restrain C-F bond distances in the disordered part. The structure additionally shows a high residual density which belongs to a disordered pentane molecule. From the data set, an accurate description was not possible; therefore, the SQUEEZE routine was applied. This procedure led to a structure with a void size of 5.3% of unit cell volume (373.85 Å^3) at 1.2 Å probe radius. Complete .cif-data of the compound are available under the CCDC number **CCDC-2154894.**

Supramolecular features

The solid state of complex **3** involves several intermolecular interactions leading to the formation of a one dimensional cationic coordination polymer. The most important interactions are the F-Bi···F-Bi interactions. Such one dimensional coordination polymers have already been described for Bi compounds in the literature.[7] In complex **3**, the intermolecular distances of Bi1···F3 are 2.652 Å and of Bi2···F2 2.713 Å, which is in consistence with the strong Lewis acidity of Bi(V) compounds. Another indication of the coordinative interaction can be found in the C–Bi–C angles. The angles between C16–Bi1–C22 (147.70°) and C28–Bi2–C34 (143.14°) are significantly widened, allowing a denser packing between the terminal F atoms and the Bi(V) central atom.

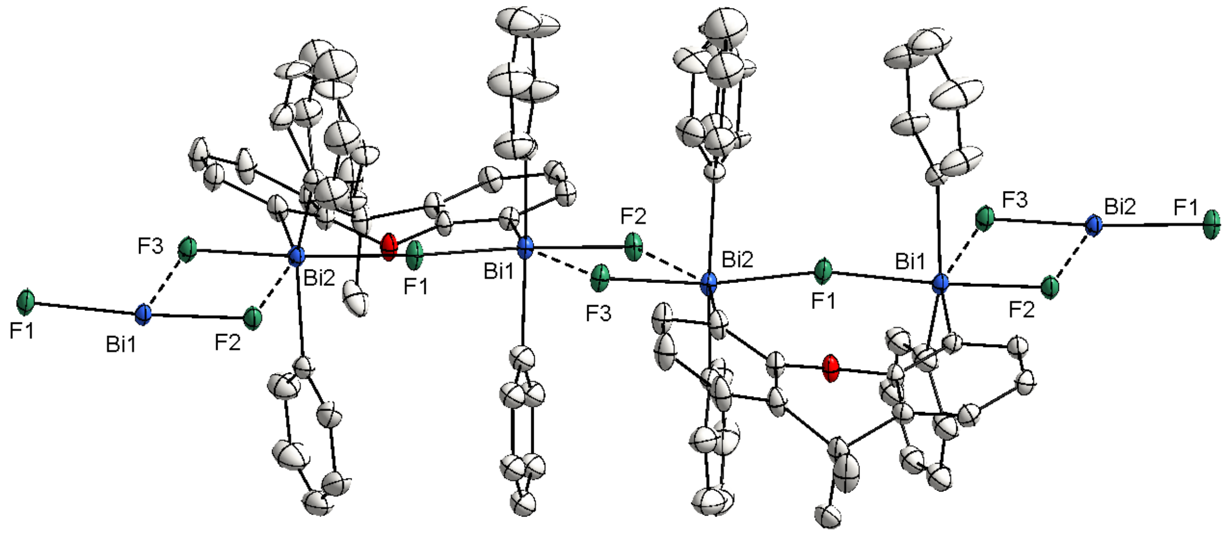

Figure S46. One dimensional coordination network of complex **3** (dashed lines) consisting of intermolecular F–Bi···F–Bi interactions. Ellipsoids are drawn at the 50% probability level.

This conformation is further favored by π-π interactions (sandwich configuration). It is noteworthy that the centroids of the phenyl ligands of two units are ca. 3.7 Å away from each other.

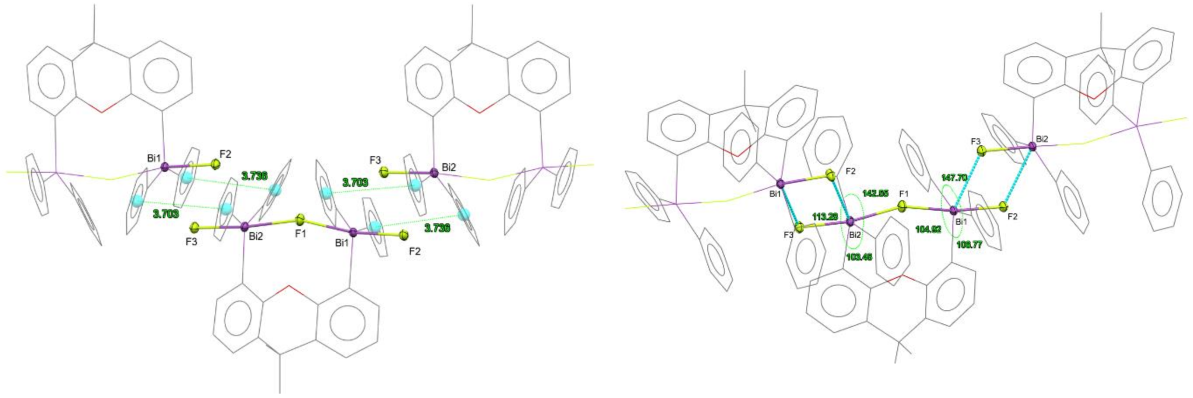

Figure S47. π stacking of phenyl ligands (left) and C–Bi–C angles (right) indicating intermolecular interactions in the solid state of complex 3.

This leads to a distorted octahedral coordination sphere at the Bi(V) central atoms in complex **3**.

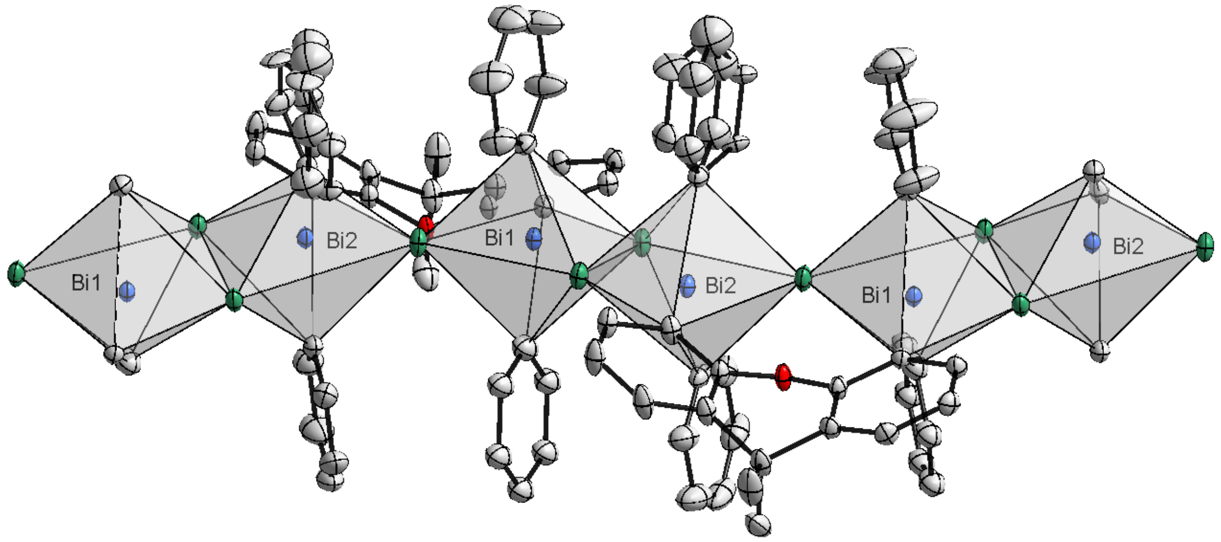

Figure S48. Polyhedral representation of distorted octahedral coordination sphere of the Bi(V) central atoms in complex **3**. Ellipsoids are drawn at the 50% probability level.

It should be noted that the cationic units of **3** build infinite chains along the crystallographic c axis. This contradicts Coulomb's law because of close distances between individual cationic units. The anionic BAr^F units orient themselves around those chains for charge compensation leading to a honeycomb like structure (Figure S49).

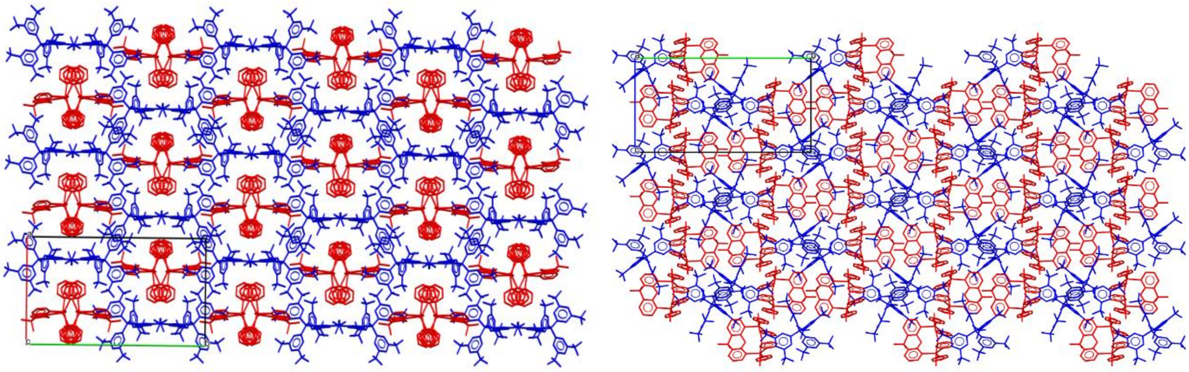

Figure S49. Honeycomb structure of **3** formed by columns of one dimensional, cationic coordination polymer (red) surrounded by weakly coordinating BAr^F (blue) anions. Unit cell is shown and the view direction is along crystallographic c axis (left) as well as a axis (right).

A different structure could be observed in complex **4**. Here the smaller SbF_6 counter ion comes closer to the Bi(V) central unit, leading to a different packing motive. Additionally the SbF_6 anion is sterically less demanding than the BAr^F analogous in **3**. In complex **4** one dimensional coordination polymers of F–Bi···F–Bi type could be found as well.

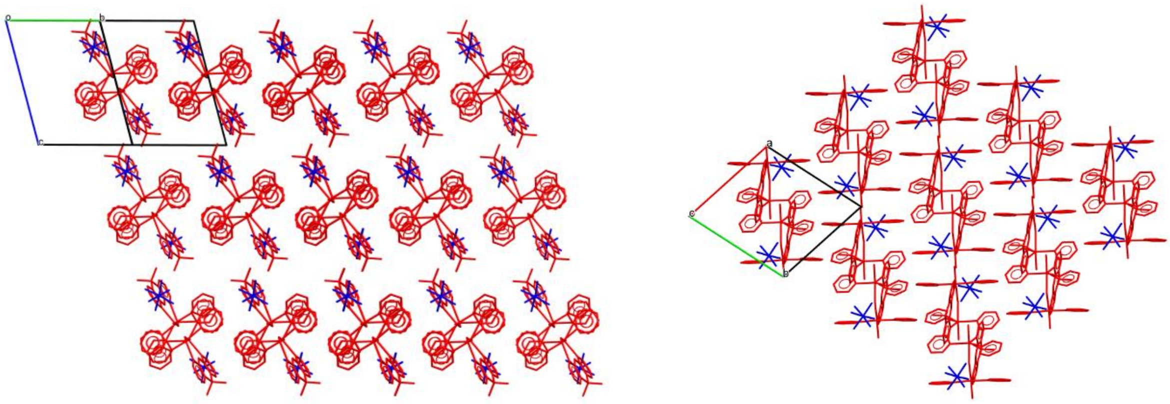

Figure S50. Columns of one dimensional, cationic coordination polymer (red) of **4** including weakly coordinating SbF_6 (blue) anions. Unit cell is shown and the view direction is randomly orientated (left) and along crystallographic c axis (right).

Table S1. Crystal data and structure refinement of complex **3**

Identification code	13153	
Empirical formula	$C_{71} H_{44} B Bi_2 F_{27} O$	
Color	colourless	
Formula weight	1854.83 $g \cdot mol^{-1}$	
Temperature	100(2) K	
Wavelength	0.71073 Å	
Crystal system	Monoclinic	
Space group	$P2_1/c$, (no. 14)	
Unit cell dimensions	a = 17.2475(7) Å	α= 90°.
	b = 27.5458(10) Å	β= 106.599(2)°.
	c = 15.4288(6) Å	γ = 90°.
Volume	7024.7(5) $Å^3$	
Z	4	
Density (calculated)	1.754 $Mg \cdot m^{-3}$	
Absorption coefficient	5.118 mm^{-1}	
F(000)	3568 e	
Crystal size	0.097 x 0.041 x 0.032 mm^3	
θ range for data collection	1.437 to 30.507°.	
Index ranges	$-24 \leq h \leq 24$, $-39 \leq k \leq 39$, $-22 \leq l \leq 21$	
Reflections collected	230929	
Independent reflections	21415 [R_{int} = 0.0519]	
Reflections with $I>2\sigma(I)$	17912	
Completeness to θ = 25.242°	99.9 %	
Absorption correction	Gaussian	
Max. and min. transmission	0.90049 and 0.71883	
Refinement method	Full-matrix least-squares on F^2	
Data / restraints / parameters	21415 / 9 / 911	
Goodness-of-fit on F^2	1.023	
Final R indices [$I>2\sigma(I)$]	R_1 = 0.0273	wR^2 = 0.0569
R indices (all data)	R_1 = 0.0393	wR^2 = 0.0612
Extinction coefficient	n/a	
Largest diff. peak and hole	2.230 and -1.460 $e \cdot Å^{-3}$	

Table S2. Bond lengths [Å] and angles [°] of complex **3**

Bi(1)-F(1)	2.2699(15)	Bi(1)-F(2)	2.1134(15)
Bi(1)-F(3)#1	2.6526(15)	Bi(1)-C(2)	2.212(2)
Bi(1)-C(16)	2.186(3)	Bi(1)-C(22)	2.1380(13)
Bi(2)-F(1)	2.2648(15)	Bi(2)-F(2)#2	2.7132(15)
Bi(2)-F(3)	2.1212(15)	Bi(2)-C(12)	2.210(2)
Bi(2)-C(28)	2.187(3)	Bi(2)-C(34)	2.160(3)
O(1)-C(1)	1.389(3)	O(1)-C(13)	1.385(3)
C(1)-C(2)	1.387(4)	C(1)-C(6)	1.396(3)
C(2)-C(3)	1.392(4)	C(3)-H(3)	0.9500
C(3)-C(4)	1.390(4)	C(4)-H(4)	0.9500
C(4)-C(5)	1.383(4)	C(5)-H(5)	0.9500
C(5)-C(6)	1.390(4)	C(6)-C(7)	1.528(4)
C(7)-C(8)	1.525(4)	C(7)-C(14)	1.546(5)
C(7)-C(15)	1.530(4)	C(8)-C(9)	1.389(4)
C(8)-C(13)	1.395(4)	C(9)-H(9)	0.9500
C(9)-C(10)	1.392(4)	C(10)-H(10)	0.9500
C(10)-C(11)	1.383(4)	C(11)-H(11)	0.9500
C(11)-C(12)	1.392(4)	C(12)-C(13)	1.389(4)
C(14)-H(14A)	0.9800	C(14)-H(14B)	0.9800
C(14)-H(14C)	0.9800	C(15)-H(15A)	0.9800
C(15)-H(15B)	0.9800	C(15)-H(15C)	0.9800
C(16)-C(17)	1.376(4)	C(16)-C(21)	1.370(4)
C(17)-H(17)	0.9500	C(17)-C(18)	1.385(5)
C(18)-H(18)	0.9500	C(18)-C(19)	1.369(6)
C(19)-H(19)	0.9500	C(19)-C(20)	1.373(6)
C(20)-H(20)	0.9500	C(20)-C(21)	1.388(5)
C(21)-H(21)	0.9500	C(28)-C(29)	1.383(4)
C(28)-C(33)	1.379(4)	C(29)-H(29)	0.9500
C(29)-C(30)	1.389(5)	C(30)-H(30)	0.9500
C(30)-C(31)	1.371(6)	C(31)-H(31)	0.9500
C(31)-C(32)	1.373(6)	C(32)-H(32)	0.9500
C(32)-C(33)	1.391(5)	C(33)-H(33)	0.9500
C(34)-C(39B)	1.3900	C(34)-C(35B)	1.3900
C(34)-C(39A)	1.367(6)	C(34)-C(35A)	1.418(6)
C(39B)-H(39B)	0.9500	C(39B)-C(38B)	1.3900
C(38B)-H(38B)	0.9500	C(38B)-C(37B)	1.3900
C(37B)-H(37B)	0.9500	C(37B)-C(36B)	1.3900

C(36B)-H(36B)	0.9500	C(36B)-C(35B)	1.3900
C(35B)-H(35B)	0.9500	C(38A)-H(38A)	0.9500
C(38A)-C(39A)	1.382(8)	C(38A)-C(37A)	1.353(11)
C(39A)-H(39A)	0.9500	F(5A)-C(46)	1.337(5)
F(6C)-C(46)	1.323(9)	F(4A)-C(46)	1.324(4)
F(7)-C(47)	1.342(4)	F(8)-C(47)	1.347(4)
F(9)-C(47)	1.333(4)	F(10)-C(54)	1.321(4)
F(11)-C(54)	1.322(4)	F(12)-C(54)	1.323(4)
F(13)-C(55)	1.339(3)	F(14)-C(55)	1.344(4)
F(15)-C(55)	1.341(3)	F(16)-C(62)	1.339(3)
F(17)-C(62)	1.334(3)	F(18)-C(62)	1.337(3)
F(19)-C(63)	1.333(4)	F(20)-C(63)	1.330(4)
F(21)-C(63)	1.337(4)	F(22)-C(70)	1.335(4)
F(23)-C(70)	1.328(4)	F(24)-C(70)	1.323(4)
F(25)-C(71)	1.332(4)	F(26)-C(71)	1.355(3)
F(27)-C(71)	1.330(4)	C(40)-C(41)	1.396(4)
C(40)-C(45)	1.401(4)	C(40)-B(1)	1.640(4)
C(41)-H(41)	0.9500	C(41)-C(42)	1.392(4)
C(42)-C(43)	1.388(4)	C(42)-C(46)	1.472(5)
C(43)-H(43)	0.9500	C(43)-C(44)	1.382(5)
C(44)-C(45)	1.395(4)	C(44)-C(47)	1.498(4)
C(45)-H(45)	0.9500	C(46)-F(4C)	1.357(9)
C(46)-F(5C)	1.383(9)	C(46)-F(6A)	1.401(5)
C(46)-F(4B)	1.426(8)	C(46)-F(6B)	1.337(9)
C(46)-F(5B)	1.364(9)	C(48)-C(49)	1.404(4)
C(48)-C(53)	1.397(4)	C(48)-B(1)	1.643(4)
C(49)-H(49)	0.9500	C(49)-C(50)	1.389(4)
C(50)-C(51)	1.382(4)	C(50)-C(54)	1.498(4)
C(51)-H(51)	0.9500	C(51)-C(52)	1.386(4)
C(52)-C(53)	1.396(4)	C(52)-C(55)	1.493(4)
C(53)-H(53)	0.9500	C(56)-C(57)	1.395(3)
C(56)-C(61)	1.406(4)	C(56)-B(1)	1.644(4)
C(57)-H(57)	0.9500	C(57)-C(58)	1.396(4)
C(58)-C(59)	1.378(4)	C(58)-C(62)	1.493(4)
C(59)-H(59)	0.9500	C(59)-C(60)	1.390(4)
C(60)-C(61)	1.393(4)	C(60)-C(63)	1.494(4)
C(61)-H(61)	0.9500	C(64)-C(65)	1.400(4)
C(64)-C(69)	1.406(4)	C(64)-B(1)	1.640(4)
C(65)-H(65)	0.9500	C(65)-C(66)	1.388(4)

C(66)-C(67)	1.390(4)	C(66)-C(70)	1.503(4)
C(67)-H(67)	0.9500	C(67)-C(68)	1.389(4)
C(68)-C(69)	1.382(4)	C(68)-C(71)	1.501(4)
C(69)-H(69)	0.9500	C(36A)-H(36A)	0.9500
C(36A)-C(35A)	1.374(6)	C(36A)-C(37A)	1.400(10)
C(35A)-H(35A)	0.9500	C(37A)-H(37A)	0.9500
C(22)-C(27)	1.3900	C(22)-C(23)	1.3900
C(27)-H(27)	0.9500	C(27)-C(26)	1.3900
C(26)-H(26)	0.9500	C(26)-C(25)	1.3900
C(25)-H(25)	0.9500	C(25)-C(24)	1.3900
C(24)-H(24)	0.9500	C(24)-C(23)	1.3900
C(23)-H(23)	0.9500		

F(1)-Bi(1)-F(3)#1	102.62(5)	F(2)-Bi(1)-F(1)	170.26(6)
F(2)-Bi(1)-F(3)#1	67.71(5)	F(2)-Bi(1)-C(2)	87.67(8)
F(2)-Bi(1)-C(16)	94.91(9)	F(2)-Bi(1)-C(22)	92.40(7)
C(2)-Bi(1)-F(1)	102.01(8)	C(2)-Bi(1)-F(3)#1	155.37(7)
C(16)-Bi(1)-F(1)	83.76(9)	C(16)-Bi(1)-F(3)#1	77.89(8)
C(16)-Bi(1)-C(2)	104.93(10)	C(22)-Bi(1)-F(1)	83.81(7)
C(22)-Bi(1)-F(3)#1	75.95(6)	C(22)-Bi(1)-C(2)	106.76(9)
C(22)-Bi(1)-C(16)	147.70(9)	F(1)-Bi(2)-F(2)#2	108.08(5)
F(3)-Bi(2)-F(1)	170.49(6)	F(3)-Bi(2)-F(2)#2	66.38(5)
F(3)-Bi(2)-C(12)	86.65(8)	F(3)-Bi(2)-C(28)	98.78(9)
F(3)-Bi(2)-C(34)	90.41(8)	C(12)-Bi(2)-F(1)	99.40(8)
C(12)-Bi(2)-F(2)#2	152.46(8)	C(28)-Bi(2)-F(1)	87.01(9)
C(28)-Bi(2)-F(2)#2	76.58(8)	C(28)-Bi(2)-C(12)	103.47(11)
C(34)-Bi(2)-F(1)	80.51(8)	C(34)-Bi(2)-F(2)#2	74.68(8)
C(34)-Bi(2)-C(12)	112.70(10)	C(34)-Bi(2)-C(28)	143.14(11)
Bi(2)-F(1)-Bi(1)	159.68(8)	Bi(1)-F(2)-Bi(2)#1	111.86(6)
Bi(2)-F(3)-Bi(1)#2	113.92(6)	C(13)-O(1)-C(1)	114.82(19)
O(1)-C(1)-C(6)	120.3(2)	C(2)-C(1)-O(1)	117.9(2)
C(2)-C(1)-C(6)	121.8(2)	C(1)-C(2)-Bi(1)	121.70(18)
C(1)-C(2)-C(3)	119.7(2)	C(3)-C(2)-Bi(1)	118.61(18)
C(2)-C(3)-H(3)	120.4	C(4)-C(3)-C(2)	119.2(3)
C(4)-C(3)-H(3)	120.4	C(3)-C(4)-H(4)	119.8
C(5)-C(4)-C(3)	120.4(3)	C(5)-C(4)-H(4)	119.8
C(4)-C(5)-H(5)	119.3	C(4)-C(5)-C(6)	121.4(2)
C(6)-C(5)-H(5)	119.3	C(1)-C(6)-C(7)	116.8(2)
C(5)-C(6)-C(1)	117.4(2)	C(5)-C(6)-C(7)	125.7(2) C(6)-

C(7)-C(14)	107.8(2)	C(6)-C(7)-C(15)	111.8(2)
C(8)-C(7)-C(6)	106.5(2)	C(8)-C(7)-C(14)	108.9(2)
C(8)-C(7)-C(15)	112.3(3)	C(15)-C(7)-C(14)	109.4(3)
C(9)-C(8)-C(7)	125.1(2)	C(9)-C(8)-C(13)	117.7(3)
C(13)-C(8)-C(7)	117.1(2)	C(8)-C(9)-H(9)	119.6
C(8)-C(9)-C(10)	120.9(3)	C(10)-C(9)-H(9)	119.6
C(9)-C(10)-H(10)	119.7	C(11)-C(10)-C(9)	120.6(3)
C(11)-C(10)-H(10)	119.7	C(10)-C(11)-H(11)	120.4
C(10)-C(11)-C(12)	119.3(3)	C(12)-C(11)-H(11)	120.4
C(11)-C(12)-Bi(2)	119.6(2)	C(13)-C(12)-Bi(2)	120.31(19)
C(13)-C(12)-C(11)	119.5(2)	O(1)-C(13)-C(8)	120.2(2)
O(1)-C(13)-C(12)	118.0(2)	C(12)-C(13)-C(8)	121.8(2)
C(7)-C(14)-H(14A)	109.5	C(7)-C(14)-H(14B)	109.5
C(7)-C(14)-H(14C)	109.5	H(14A)-C(14)-H(14B)	109.5
H(14A)-C(14)-H(14C)	109.5	H(14B)-C(14)-H(14C)	109.5
C(7)-C(15)-H(15A)	109.5	C(7)-C(15)-H(15B)	109.5
C(7)-C(15)-H(15C)	109.5	H(15A)-C(15)-H(15B)	109.5
H(15A)-C(15)-H(15C)	109.5	H(15B)-C(15)-H(15C)	109.5
C(17)-C(16)-Bi(1)	122.1(2)	C(21)-C(16)-Bi(1)	115.7(2)
C(21)-C(16)-C(17)	122.1(3)	C(16)-C(17)-H(17)	121.0
C(16)-C(17)-C(18)	118.0(3)	C(18)-C(17)-H(17)	121.0
C(17)-C(18)-H(18)	119.4	C(19)-C(18)-C(17)	121.2(4)
C(19)-C(18)-H(18)	119.4	C(18)-C(19)-H(19)	120.1
C(18)-C(19)-C(20)	119.8(4)	C(20)-C(19)-H(19)	120.1
C(19)-C(20)-H(20)	119.8	C(19)-C(20)-C(21)	120.3(4)
C(21)-C(20)-H(20)	119.8	C(16)-C(21)-C(20)	118.7(3)
C(16)-C(21)-H(21)	120.7	C(20)-C(21)-H(21)	120.7
C(29)-C(28)-Bi(2)	115.3(2)	C(33)-C(28)-Bi(2)	121.9(2)
C(33)-C(28)-C(29)	122.7(3)	C(28)-C(29)-H(29)	121.0
C(28)-C(29)-C(30)	117.9(4)	C(30)-C(29)-H(29)	121.0
C(29)-C(30)-H(30)	119.8	C(31)-C(30)-C(29)	120.4(4)
C(31)-C(30)-H(30)	119.8	C(30)-C(31)-H(31)	119.7
C(30)-C(31)-C(32)	120.6(3)	C(32)-C(31)-H(31)	119.7
C(31)-C(32)-H(32)	119.7	C(31)-C(32)-C(33)	120.6(4)
C(33)-C(32)-H(32)	119.7	C(28)-C(33)-C(32)	117.7(3)
C(28)-C(33)-H(33)	121.1	C(32)-C(33)-H(33)	121.1
C(39B)-C(34)-Bi(2)	112.4(2)	C(39B)-C(34)-C(35B)	120.0
C(35B)-C(34)-Bi(2)	127.6(2)	C(39A)-C(34)-Bi(2)	120.8(3)
C(39A)-C(34)-C(35A)	120.5(4)	C(35A)-C(34)-Bi(2)	118.4(3)

C(34)-C(39B)-H(39B)	120.0	C(38B)-C(39B)-C(34)	120.0
C(38B)-C(39B)-H(39B)	120.0	C(39B)-C(38B)-H(38B)	120.0
C(37B)-C(38B)-C(39B)	120.0	C(37B)-C(38B)-H(38B)	120.0
C(38B)-C(37B)-H(37B)	120.0	C(38B)-C(37B)-C(36B)	120.0
C(36B)-C(37B)-H(37B)	120.0	C(37B)-C(36B)-H(36B)	120.0
C(37B)-C(36B)-C(35B)	120.0	C(35B)-C(36B)-H(36B)	120.0
C(34)-C(35B)-H(35B)	120.0	C(36B)-C(35B)-C(34)	120.0
C(36B)-C(35B)-H(35B)	120.0	C(39A)-C(38A)-H(38A)	119.8
C(37A)-C(38A)-H(38A)	119.8	C(37A)-C(38A)-C(39A)	120.5(5)
C(34)-C(39A)-C(38A)	119.4(5)	C(34)-C(39A)-H(39A)	120.3
C(38A)-C(39A)-H(39A)	120.3	C(41)-C(40)-C(45)	115.9(2)
C(41)-C(40)-B(1)	120.5(2)	C(45)-C(40)-B(1)	123.6(3)
C(40)-C(41)-H(41)	118.9	C(42)-C(41)-C(40)	122.2(3)
C(42)-C(41)-H(41)	118.9	C(41)-C(42)-C(46)	119.3(3)
C(43)-C(42)-C(41)	120.7(3)	C(43)-C(42)-C(46)	120.0(3)
C(42)-C(43)-H(43)	120.8	C(44)-C(43)-C(42)	118.3(3)
C(44)-C(43)-H(43)	120.8	C(43)-C(44)-C(45)	120.7(3)
C(43)-C(44)-C(47)	120.2(3)	C(45)-C(44)-C(47)	119.0(3)
C(40)-C(45)-H(45)	118.9	C(44)-C(45)-C(40)	122.1(3)
C(44)-C(45)-H(45)	118.9	F(5A)-C(46)-C(42)	114.8(3)
F(5A)-C(46)-F(6A)	103.3(4)	F(6C)-C(46)-C(42)	114.6(8)
F(6C)-C(46)-F(4C)	101.7(11)	F(6C)-C(46)-F(5C)	111.7(9)
F(4A)-C(46)-F(5A)	109.9(5)	F(4A)-C(46)-C(42)	114.3(4)
F(4A)-C(46)-F(6A)	104.7(4)	F(4C)-C(46)-C(42)	113.4(10)
F(4C)-C(46)-F(5C)	108.2(10)	F(5C)-C(46)-C(42)	107.2(7)
F(6A)-C(46)-C(42)	108.9(3)	F(4B)-C(46)-C(42)	110.9(6)
F(6B)-C(46)-C(42)	124.0(9)	F(6B)-C(46)-F(4B)	103.1(12)
F(6B)-C(46)-F(5B)	103.2(12)	F(5B)-C(46)-C(42)	118.9(8)
F(5B)-C(46)-F(4B)	91.1(9)	F(7)-C(47)-F(8)	106.4(2)
F(7)-C(47)-C(44)	112.3(2)	F(8)-C(47)-C(44)	112.0(3)
F(9)-C(47)-F(7)	106.9(3)	F(9)-C(47)-F(8)	106.5(2)
F(9)-C(47)-C(44)	112.4(2)	C(49)-C(48)-B(1)	119.6(2)
C(53)-C(48)-C(49)	115.5(2)	C(53)-C(48)-B(1)	124.9(2)
C(48)-C(49)-H(49)	118.8	C(50)-C(49)-C(48)	122.5(3)
C(50)-C(49)-H(49)	118.8	C(49)-C(50)-C(54)	118.4(3)
C(51)-C(50)-C(49)	120.8(3)	C(51)-C(50)-C(54)	120.8(3)
C(50)-C(51)-H(51)	120.9	C(50)-C(51)-C(52)	118.1(3)
C(52)-C(51)-H(51)	120.9	C(51)-C(52)-C(53)	120.9(3)
C(51)-C(52)-C(55)	120.1(3)	C(53)-C(52)-C(55)	119.0(3)

C(48)-C(53)-H(53)	118.9	C(52)-C(53)-C(48)	122.2(3)
C(52)-C(53)-H(53)	118.9	F(10)-C(54)-F(11)	105.6(3)
F(10)-C(54)-F(12)	106.3(3)	F(10)-C(54)-C(50)	112.7(3)
F(11)-C(54)-F(12)	106.1(3)	F(11)-C(54)-C(50)	112.3(3)
F(12)-C(54)-C(50)	113.2(3)	F(13)-C(55)-F(14)	106.6(2)
F(13)-C(55)-F(15)	106.1(2)	F(13)-C(55)-C(52)	113.2(3)
F(14)-C(55)-C(52)	112.8(2)	F(15)-C(55)-F(14)	105.8(2)
F(15)-C(55)-C(52)	111.8(2)	C(57)-C(56)-C(61)	115.4(2)
C(57)-C(56)-B(1)	123.8(2)	C(61)-C(56)-B(1)	120.8(2)
C(56)-C(57)-H(57)	118.9	C(56)-C(57)-C(58)	122.1(2)
C(58)-C(57)-H(57)	118.9	C(57)-C(58)-C(62)	117.8(2)
C(59)-C(58)-C(57)	121.4(2)	C(59)-C(58)-C(62)	120.7(2)
C(58)-C(59)-H(59)	121.0	C(58)-C(59)-C(60)	117.9(2)
C(60)-C(59)-H(59)	121.0	C(59)-C(60)-C(61)	120.6(3)
C(59)-C(60)-C(63)	118.7(3)	C(61)-C(60)-C(63)	120.7(2)
C(56)-C(61)-H(61)	118.7	C(60)-C(61)-C(56)	122.6(2)
C(60)-C(61)-H(61)	118.7	F(16)-C(62)-C(58)	112.4(2)
F(17)-C(62)-F(16)	106.1(2)	F(17)-C(62)-F(18)	105.6(2)
F(17)-C(62)-C(58)	112.0(2)	F(18)-C(62)-F(16)	106.8(2)
F(18)-C(62)-C(58)	113.4(2)	F(19)-C(63)-F(21)	105.9(3)
F(19)-C(63)-C(60)	112.6(3)	F(20)-C(63)-F(19)	106.6(3)
F(20)-C(63)-F(21)	105.4(3)	F(20)-C(63)-C(60)	113.2(3)
F(21)-C(63)-C(60)	112.6(2)	C(65)-C(64)-C(69)	115.6(3)
C(65)-C(64)-B(1)	125.0(2)	C(69)-C(64)-B(1)	119.2(2)
C(64)-C(65)-H(65)	119.0	C(66)-C(65)-C(64)	122.0(3)
C(66)-C(65)-H(65)	119.0	C(65)-C(66)-C(67)	121.4(3)
C(65)-C(66)-C(70)	118.8(3)	C(67)-C(66)-C(70)	119.8(3)
C(66)-C(67)-H(67)	121.2	C(68)-C(67)-C(66)	117.6(3)
C(68)-C(67)-H(67)	121.2	C(67)-C(68)-C(71)	119.6(3)
C(69)-C(68)-C(67)	120.8(3)	C(69)-C(68)-C(71)	119.5(3)
C(64)-C(69)-H(69)	118.7	C(68)-C(69)-C(64)	122.6(3)
C(68)-C(69)-H(69)	118.7	F(22)-C(70)-C(66)	112.3(3)
F(23)-C(70)-F(22)	104.6(3)	F(23)-C(70)-C(66)	112.7(3)
F(24)-C(70)-F(22)	106.6(3)	F(24)-C(70)-F(23)	106.8(3)
F(24)-C(70)-C(66)	113.2(3)	F(25)-C(71)-F(26)	106.2(2)
F(25)-C(71)-C(68)	112.9(3)	F(26)-C(71)-C(68)	111.1(2)
F(27)-C(71)-F(25)	107.8(3)	F(27)-C(71)-F(26)	105.7(3)
F(27)-C(71)-C(68)	112.7(2)	C(40)-B(1)-C(48)	110.8(2)
C(40)-B(1)-C(56)	107.3(2)	C(40)-B(1)-C(64)	108.5(2)

C(48)-B(1)-C(56)	110.7(2)	C(64)-B(1)-C(48)	108.0(2)
C(64)-B(1)-C(56)	111.6(2)	C(35A)-C(36A)-H(36A)	120.7
C(35A)-C(36A)-C(37A)	118.7(6)	C(37A)-C(36A)-H(36A)	120.7
C(34)-C(35A)-H(35A)	120.4	C(36A)-C(35A)-C(34)	119.2(5)
C(36A)-C(35A)-H(35A)	120.4	C(38A)-C(37A)-C(36A)	121.5(5)
C(38A)-C(37A)-H(37A)	119.3	C(36A)-C(37A)-H(37A)	119.3
C(27)-C(22)-Bi(1)	123.41(10)	C(27)-C(22)-C(23)	120.0
C(23)-C(22)-Bi(1)	116.57(10)	C(22)-C(27)-H(27)	120.0
C(26)-C(27)-C(22)	120.0	C(26)-C(27)-H(27)	120.0
C(27)-C(26)-H(26)	120.0	C(25)-C(26)-C(27)	120.0
C(25)-C(26)-H(26)	120.0	C(26)-C(25)-H(25)	120.0
C(26)-C(25)-C(24)	120.0	C(24)-C(25)-H(25)	120.0
C(25)-C(24)-H(24)	120.0	C(23)-C(24)-C(25)	120.0
C(23)-C(24)-H(24)	120.0	C(22)-C(23)-H(23)	120.0
C(24)-C(23)-C(22)	120.0	C(24)-C(23)-H(23)	120.0

_

Symmetry transformations used to generate equivalent atoms:

#1 x,-y+1/2,z+1/2   #2 x,-y+1/2,z-1/2

Single crystal structure analysis of complex 4 (13212)

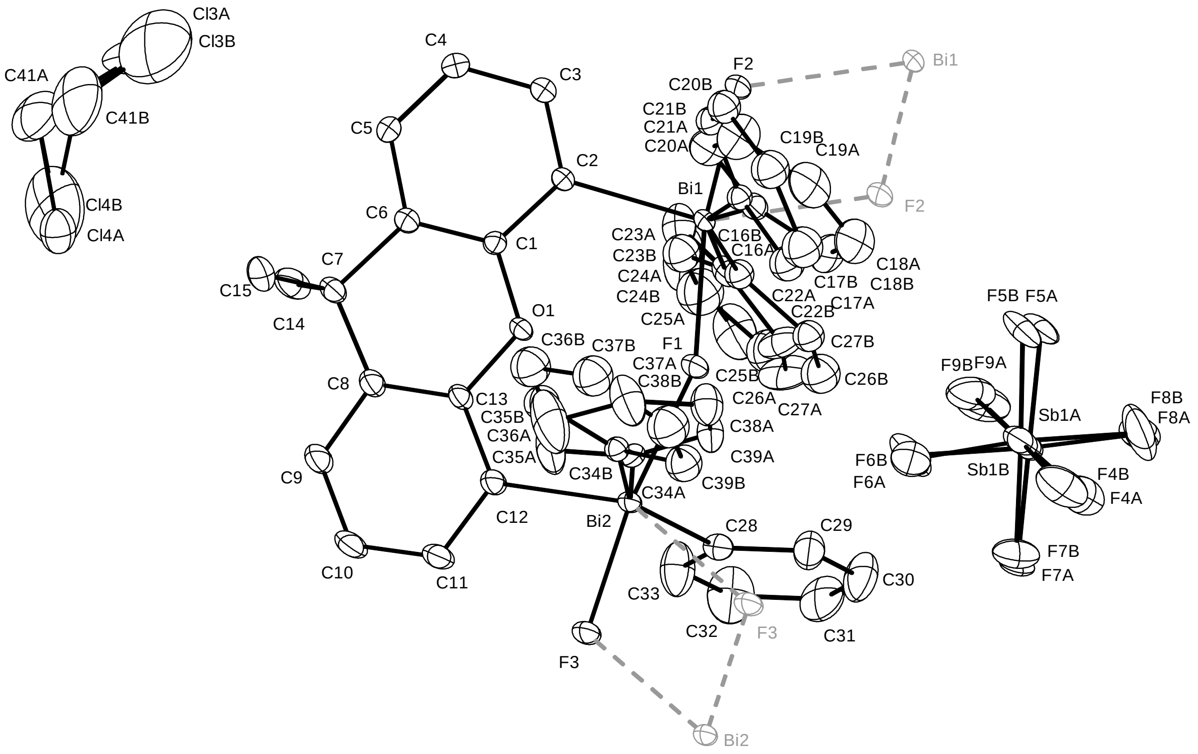

Figure S51. The molecular structure of complex **4**. H atoms have been removed for clarity. Dashed lines indicate polymeric extensions. Ellipsoids are drawn at the 50% probability level.

X-ray Crystal Structure Analysis of complex 4:
C_{41} H_{36} Bi_2 Cl_3 F_9 O Sb, M_r = 1361.76 g mol^{-1}, colorless prism, crystal size 0.095 x 0.075 x 0.020 mm^3, triclinic, space group P-1 [2], a = 12.0057(7) Å, b = 13.7330(8) Å, c = 14.4346(9) Å, α = 68.793(2)°, β = 106.599(2)°, γ = 74.298(2)°, V = 2134.9(2) Å^3, T = 100(2) K, Z = 2, D_{calc} = 2.118 g·cm^3, λ = 0.71073 Å, $\mu(Mo\text{-}K_\alpha)$ = 9.110 mm^{-1}, Gaussian absorption correction ($T_{\min}$ = 0.57176, $T_{\max}$ = 0.84805), Bruker-AXS Kappa Mach3 with APEX-II detector and IµS microfocus Mo-anode X-ray source, 1.514 < θ < 34.337°, 81844 measured reflections, 17783 independent reflections, 14696 reflections with $I > 2\sigma(I)$, R_{int} = 0.0319. The structure was solved by *SHELXT* and refined by full-matrix least-squares (*SHELXL*) against F^2 to R_1 = 0.0240 [$I > 2\sigma(I)$], wR_2 = 0.0575 [all data], 688 parameters and 78 restraints.

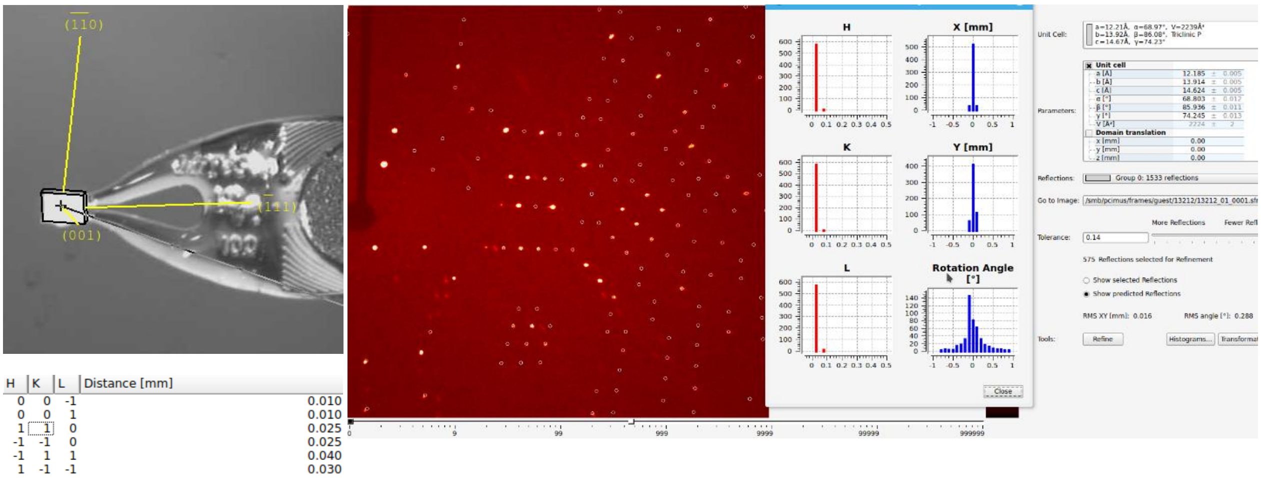

Figure S52. Crystal faces and unit cell determination/refinement of complex **4**.

INTENSITY STATISTICS FOR DATASET

Resolution	#Data	#Theory	%Complete	Redundancy	Mean I	Mean I/s	Rmerge	Rsigma
Inf - 2.45	307	307	100.0	9.09	74.62	80.68	0.0312	0.0114
2.45 - 1.63	731	731	100.0	9.16	53.44	72.06	0.0244	0.0117
1.63 - 1.29	1038	1038	100.0	9.06	36.10	64.54	0.0237	0.0124
1.29 - 1.13	1008	1008	100.0	8.66	24.07	53.89	0.0264	0.0134
1.13 - 1.03	1009	1009	100.0	6.84	20.09	42.65	0.0294	0.0170
1.03 - 0.95	1137	1137	100.0	5.15	17.40	34.25	0.0312	0.0216
0.95 - 0.90	904	904	100.0	4.36	13.89	28.40	0.0360	0.0260
0.90 - 0.85	1151	1151	100.0	3.95	11.81	24.03	0.0379	0.0298
0.85 - 0.81	1127	1127	100.0	3.75	9.61	20.41	0.0436	0.0349
0.81 - 0.78	1020	1020	100.0	3.57	8.98	17.87	0.0454	0.0391
0.78 - 0.75	1161	1162	99.9	3.39	7.92	16.54	0.0516	0.0450
0.75 - 0.73	906	908	99.8	3.26	7.08	14.30	0.0603	0.0511
0.73 - 0.71	983	986	99.7	3.17	6.46	13.46	0.0627	0.0564
0.71 - 0.69	1118	1121	99.7	3.02	5.85	12.11	0.0685	0.0641
0.69 - 0.67	1226	1236	99.2	2.91	4.97	10.64	0.0776	0.0751
0.67 - 0.66	705	710	99.3	2.81	4.59	9.62	0.0836	0.0830
0.66 - 0.64	1490	1521	98.0	2.72	4.43	8.95	0.0904	0.0894
0.64 - 0.63	770	782	98.5	2.59	3.71	7.35	0.1071	0.1085
0.63 - 0.62	894	925	96.6	2.58	3.78	7.57	0.1084	0.1082
0.62 - 0.61	889	929	95.7	2.44	3.68	7.05	0.1174	0.1165
0.61 - 0.60	840	1101	76.3	1.69	2.77	5.21	0.1449	0.1792
0.70 - 0.60	7376	7767	95.0	2.57	4.14	8.44	0.0941	0.0979
Inf - 0.60	20414	20813	98.1	4.25	13.04	24.03	0.0328	0.0303

Eight reflections have been excluded from the data set before final refinement cycles because of high I/σI A resolution cut off (SHEL 99 0.63) was applied to exclude poorly determined intensities at high diffraction angles. The structure contains several disordered sub units. Two phenyl ligands of Bi1 and one of Bi2 occur to be disordered over two positions with fixed occupancies 60:40%. ISOR instruction was used to equalize thermal ellipsoids of the disorder phenyl ring at Bi2 (ISOR 0.01 0.02 C36A C35A C34A C39A C38A C37A). The SbF_6- anion shows a positional disorder over two slightly different positions with a free refined occupancy

of 52:48%. Within in the asymmetric unit, two disordered DCM solute molecules could be found. ISOR instructions was used to treat thermal ellipsoids of Cl and C atoms (ISOR 0.01 0.02 Cl3B Cl3A Cl4A Cl4B and ISOR 0.01 0.02 Cl2 C40 Cl1). Complete .cif-data of the compound are available under the CCDC number **CCDC-2154889**.

Supramolecular features

The solid state of complex **4** involves intermolecular interactions leading to the formation of a cationic 1D coordination polymer. The most important interactions are the F–Bi···F–Bi interactions. Such 1D coordination polymers have already been described for Bi compounds in the literature and are comparable to thus of complex **3**. In complex **4**, the intermolecular distances Bi1–F2 are 2.674 Å and Bi2–F3 2.661 Å, which is in consistence with the strong Lewis acidity of Bi(V) compounds. Another indication of the coordinative interaction can be found in the C–Bi–C angles. The angles between C16A–Bi1–C22A (148.22°) and C28–Bi2–C34A (141.07°) are significantly widened to allow dense packing between the terminal F atoms and the Bi(V) central atoms.

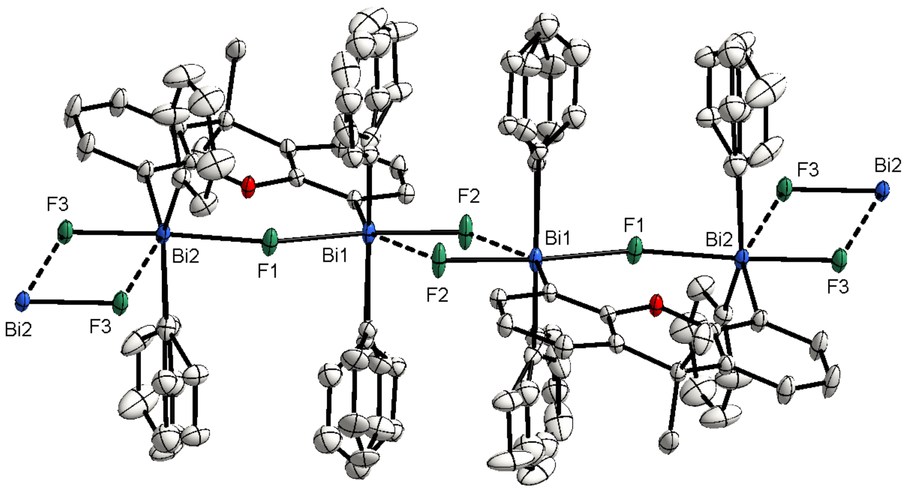

Figure S53. One-dimensional coordination network of complex **4** (dashed lines) consisting of intermolecular F–Bi···F–Bi interactions. Ellipsoids are drawn at the 50% probability level.

This conformation is further favored by π-π interactions (sandwich configuration). It is noteworthy that the centroids of the phenyl ligands of two cationic units are ca. 3.7 Å away from each other.

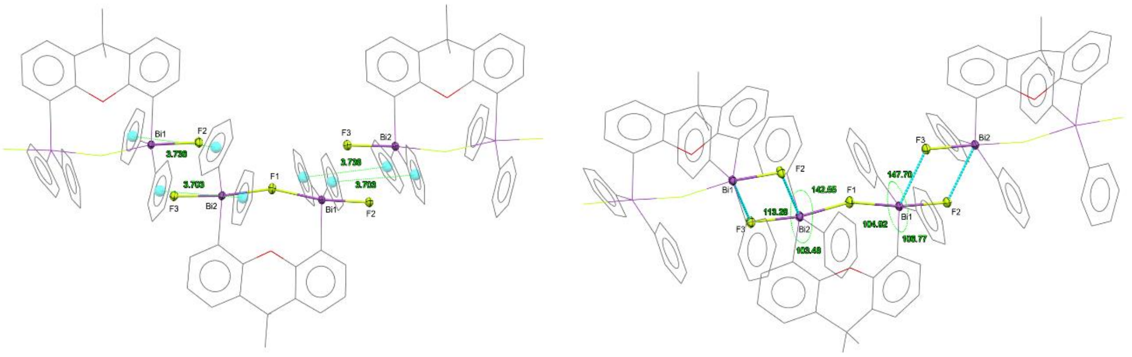

Figure S54. π stacking of phenyl ligands (left) and C-Bi-C angles (right) indicating intermolecular interactions in the solid state of complex **4**.

This leads to a distorted octahedral coordination sphere at the Bi(V) central atoms in complex **4**.

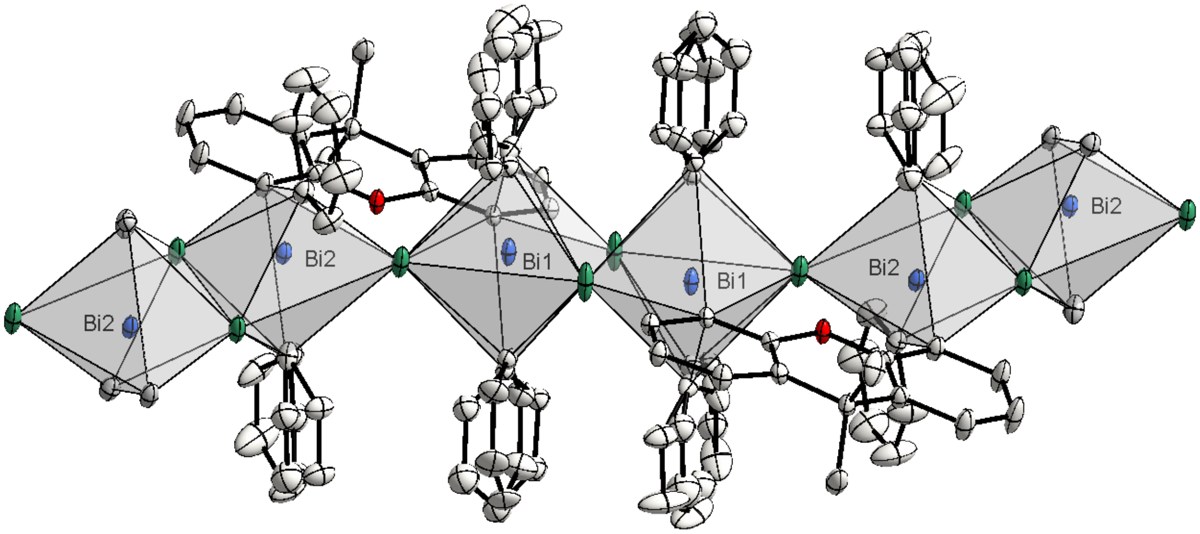

Figure S55. Polyhedral representation of distorted octahedral coordination sphere of the Bi(V) central atoms in complex **4**. Ellipsoids are drawn at the 50% probability level.

Table S3. Crystal data and structure refinement of complex **4**.

Identification code	13212	
Empirical formula	C_{41} H_{36} Bi_2 Cl_3 F_9 O Sb	
Color	colourless	
Formula weight	1361.76 $g{\cdot}mol^{-1}$	
Temperature	100(2) K	
Wavelength	0.71073 Å	
Crystal system	Triclinic	
Space group	*P*-1, (no. 2)	
Unit cell dimensions	a = 12.0057(7) Å	α= 68.793(2)°.
	b = 13.7330(8) Å	β= 85.949(2)°.
	c = 14.4346(9) Å	γ = 74.298(2)°.
Volume	2134.9(2) $Å^3$	
Z	2	
Density (calculated)	2.118 $Mg{\cdot}m^{-3}$	
Absorption coefficient	9.110 mm^{-1}	
F(000)	1278 e	
Crystal size	0.095 x 0.075 x 0.020 mm^3	
θ range for data collection	1.514 to 34.337°.	
Index ranges	$-19 \leq h \leq 19$, $-21 \leq k \leq 21$, $-22 \leq l \leq 22$	
Reflections collected	81844	
Independent reflections	17783 [R_{int} = 0.0319]	
Reflections with $I>2\sigma(I)$	14696	
Completeness to θ = 25.242°	99.9 %	
Absorption correction	Gaussian	
Max. and min. transmission	0.84805 and 0.57176	
Refinement method	Full-matrix least-squares on F^2	
Data / restraints / parameters	17783 / 78 / 688	
Goodness-of-fit on F^2	1.024	
Final R indices [$I>2\sigma(I)$]	R_1 = 0.0240	wR^2 = 0.0533
R indices (all data)	R_1 = 0.0357	wR^2 = 0.0575
Extinction coefficient	n/a	
Largest diff. peak and hole	2.186 and -1.985 $e{\cdot}Å^{-3}$	

Table S4. Bond lengths [Å] and angles [°] of complex **4**.

Bi(1)-F(1)	2.2715(14)	Bi(1)-F(2)	2.1186(14)
Bi(1)-F(2)#1	2.6735(15)	Bi(1)-C(2)	2.222(2)
Bi(1)-C(16A)	2.098(6)	Bi(1)-C(16B)	2.326(9)
Bi(1)-C(22A)	2.282(6)	Bi(1)-C(22B)	2.047(11)
Bi(2)-F(1)	2.2996(14)	Bi(2)-F(3)#2	2.6613(14)
Bi(2)-F(3)	2.1158(14)	Bi(2)-C(12)	2.219(2)
Bi(2)-C(28)	2.184(3)	Bi(2)-C(34A)	2.135(8)
Bi(2)-C(34B)	2.262(12)	O(1)-C(1)	1.379(3)
O(1)-C(13)	1.374(3)	C(1)-C(2)	1.399(3)
C(1)-C(6)	1.389(3)	C(2)-C(3)	1.388(3)
C(3)-H(3)	0.9500	C(3)-C(4)	1.389(3)
C(4)-H(4)	0.9500	C(4)-C(5)	1.385(3)
C(5)-H(5)	0.9500	C(5)-C(6)	1.406(3)
C(6)-C(7)	1.528(3)	C(7)-C(8)	1.533(3)
C(7)-C(14)	1.538(4)	C(7)-C(15)	1.529(4)
C(8)-C(9)	1.398(3)	C(8)-C(13)	1.393(3)
C(9)-H(9)	0.9500	C(9)-C(10)	1.389(4)
C(10)-H(10)	0.9500	C(10)-C(11)	1.389(4)
C(11)-H(11)	0.9500	C(11)-C(12)	1.393(3)
C(12)-C(13)	1.398(3)	C(14)-H(14A)	0.9800
C(14)-H(14B)	0.9800	C(14)-H(14C)	0.9800
C(15)-H(15A)	0.9800	C(15)-H(15B)	0.9800
C(15)-H(15C)	0.9800	C(16A)-C(17A)	1.381(7)
C(16A)-C(21A)	1.384(9)	C(16B)-C(17B)	1.411(12)
C(16B)-C(21B)	1.349(10)	C(17A)-H(17A)	0.9500
C(17A)-C(18A)	1.401(7)	C(17B)-H(17B)	0.9500
C(17B)-C(18B)	1.391(12)	C(18A)-H(18A)	0.9500
C(18A)-C(19A)	1.359(10)	C(18B)-H(18B)	0.9500
C(18B)-C(19B)	1.388(12)	C(19A)-H(19A)	0.9500
C(19A)-C(20A)	1.365(9)	C(19B)-H(19B)	0.9500
C(19B)-C(20B)	1.378(12)	C(20A)-H(20A)	0.9500
C(20A)-C(21A)	1.414(9)	C(20B)-H(20B)	0.9500
C(20B)-C(21B)	1.376(14)	C(21A)-H(21A)	0.9500
C(21B)-H(21B)	0.9500	C(22A)-C(23A)	1.362(11)
C(22A)-C(27A)	1.380(8)	C(22B)-C(23B)	1.400(14)
C(22B)-C(27B)	1.401(14)	C(23A)-H(23A)	0.9500
C(23A)-C(24A)	1.392(9)	C(23B)-H(23B)	0.9500
C(23B)-C(24B)	1.45(2)	C(24A)-H(24A)	0.9500

C(24A)-C(25A)	1.380(12)	C(24B)-H(24B)	0.9500
C(24B)-C(25B)	1.356(18)	C(25A)-H(25A)	0.9500
C(25A)-C(26A)	1.380(13)	C(25B)-H(25B)	0.9500
C(25B)-C(26B)	1.363(16)	C(26A)-H(26A)	0.9500
C(26A)-C(27A)	1.394(10)	C(26B)-H(26B)	0.9500
C(26B)-C(27B)	1.387(13)	C(27A)-H(27A)	0.9500
C(27B)-H(27B)	0.9500	C(28)-C(29)	1.377(4)
C(28)-C(33)	1.383(4)	C(29)-H(29)	0.9500
C(29)-C(30)	1.388(4)	C(30)-H(30)	0.9500
C(30)-C(31)	1.376(5)	C(31)-H(31)	0.9500
C(31)-C(32)	1.377(6)	C(32)-H(32)	0.9500
C(32)-C(33)	1.391(5)	C(33)-H(33)	0.9500
C(34A)-C(35A)	1.403(9)	C(34A)-C(39A)	1.379(9)
C(34B)-C(35B)	1.338(12)	C(34B)-C(39B)	1.385(14)
C(35A)-H(35A)	0.9500	C(35A)-C(36A)	1.395(9)
C(35B)-H(35B)	0.9500	C(35B)-C(36B)	1.373(12)
C(36A)-H(36A)	0.9500	C(36A)-C(37A)	1.372(9)
C(36B)-H(36B)	0.9500	C(36B)-C(37B)	1.348(14)
C(37A)-H(37A)	0.9500	C(37A)-C(38A)	1.371(8)
C(37B)-H(37B)	0.9500	C(37B)-C(38B)	1.381(14)
C(38A)-H(38A)	0.9500	C(38A)-C(39A)	1.387(7)
C(38B)-H(38B)	0.9500	C(38B)-C(39B)	1.396(14)
C(39A)-H(39A)	0.9500	C(39B)-H(39B)	0.9500
Sb(1A)-F(4A)	1.85(2)	Sb(1A)-F(5A)	1.909(15)
Sb(1A)-F(6A)	1.895(15)	Sb(1A)-F(7A)	1.928(14)
Sb(1A)-F(8A)	1.881(15)	Sb(1A)-F(9A)	1.87(2)
Sb(1B)-F(4B)	1.89(2)	Sb(1B)-F(5B)	1.82(2)
Sb(1B)-F(6B)	1.830(18)	Sb(1B)-F(7B)	1.799(16)
Sb(1B)-F(8B)	1.875(16)	Sb(1B)-F(9B)	1.86(2)
Cl(1)-C(40)	1.633(9)	Cl(2)-C(40)	1.376(8)
C(40)-H(40A)	0.9900	C(40)-H(40B)	0.9900
Cl(3A)-C(41A)	1.865(14)	Cl(4A)-C(41A)	1.753(12)
C(41A)-H(41C)	0.9900	C(41A)-H(41D)	0.9900
Cl(3B)-C(41B)	1.633(18)	Cl(4B)-C(41B)	1.748(17)
C(41B)-H(41A)	0.9900	C(41B)-H(41B)	0.9900

F(1)-Bi(1)-F(2)#1	103.16(5)	F(1)-Bi(1)-C(16B)	84.7(2)
F(1)-Bi(1)-C(22A)	86.08(17)	F(2)-Bi(1)-F(1)	169.68(6)
F(2)-Bi(1)-F(2)#1	66.76(6)	F(2)-Bi(1)-C(2)	86.98(7)

F(2)-Bi(1)-C(16B)	91.4(2)	F(2)-Bi(1)-C(22A)	93.93(16)
C(2)-Bi(1)-F(1)	103.23(7)	C(2)-Bi(1)-F(2)#1	153.36(6)
C(2)-Bi(1)-C(16B)	104.5(2)	C(2)-Bi(1)-C(22A)	98.90(18)
C(16A)-Bi(1)-F(1)	83.02(15)	C(16A)-Bi(1)-F(2)	91.70(15)
C(16A)-Bi(1)-F(2)#1	74.24(17)	C(16A)-Bi(1)-C(2)	112.63(18)
C(16A)-Bi(1)-C(22A)	148.2(2)	C(22A)-Bi(1)-F(2)#1	79.37(16)
C(22B)-Bi(1)-F(1)	82.4(3)	C(22B)-Bi(1)-F(2)	96.2(3)
C(22B)-Bi(1)-C(2)	106.6(3)	C(22B)-Bi(1)-C(16B)	148.3(3)
F(1)-Bi(2)-F(3)#2	108.06(5)	F(3)-Bi(2)-F(1)	172.48(5)
F(3)-Bi(2)-F(3)#2	64.60(6)	F(3)-Bi(2)-C(12)	85.51(7)
F(3)-Bi(2)-C(28)	93.58(8)	F(3)-Bi(2)-C(34A)	95.8(2)
F(3)-Bi(2)-C(34B)	95.3(3)	C(12)-Bi(2)-F(1)	101.88(7)
C(12)-Bi(2)-F(3)#2	150.05(7)	C(12)-Bi(2)-C(34B)	102.4(3)
C(28)-Bi(2)-F(1)	82.65(8)	C(28)-Bi(2)-F(3)#2	75.80(7)
C(28)-Bi(2)-C(12)	109.68(10)	C(28)-Bi(2)-C(34B)	147.3(3)
C(34A)-Bi(2)-F(1)	83.3(2)	C(34A)-Bi(2)-F(3)#2	74.41(19)
C(34A)-Bi(2)-C(12)	108.7(2)	C(34A)-Bi(2)-C(28)	141.1(2)
C(34B)-Bi(2)-F(1)	84.6(3)	C(34B)-Bi(2)-F(3)#2	79.8(3)
Bi(1)-F(1)-Bi(2)	158.12(7)	Bi(1)-F(2)-Bi(1)#1	113.24(6)
Bi(2)-F(3)-Bi(2)#2	115.40(6)	C(13)-O(1)-C(1)	118.97(17)
O(1)-C(1)-C(2)	115.05(18)	O(1)-C(1)-C(6)	122.69(19)
C(6)-C(1)-C(2)	122.26(19)	C(1)-C(2)-Bi(1)	120.77(15)
C(3)-C(2)-Bi(1)	119.08(16)	C(3)-C(2)-C(1)	119.7(2)
C(2)-C(3)-H(3)	120.4	C(2)-C(3)-C(4)	119.3(2)
C(4)-C(3)-H(3)	120.4	C(3)-C(4)-H(4)	119.8
C(5)-C(4)-C(3)	120.4(2)	C(5)-C(4)-H(4)	119.8
C(4)-C(5)-H(5)	119.1	C(4)-C(5)-C(6)	121.8(2)
C(6)-C(5)-H(5)	119.1	C(1)-C(6)-C(5)	116.6(2)
C(1)-C(6)-C(7)	122.99(19)	C(5)-C(6)-C(7)	120.4(2)
C(6)-C(7)-C(8)	109.24(19)	C(6)-C(7)-C(14)	109.2(2)
C(6)-C(7)-C(15)	110.1(2)	C(8)-C(7)-C(14)	109.2(2)
C(15)-C(7)-C(8)	109.2(2)	C(15)-C(7)-C(14)	109.9(2)
C(9)-C(8)-C(7)	120.4(2)	C(13)-C(8)-C(7)	122.4(2)
C(13)-C(8)-C(9)	117.1(2)	C(8)-C(9)-H(9)	119.0
C(10)-C(9)-C(8)	121.9(2)	C(10)-C(9)-H(9)	119.0
C(9)-C(10)-H(10)	119.9	C(11)-C(10)-C(9)	120.1(2)
C(11)-C(10)-H(10)	119.9	C(10)-C(11)-H(11)	120.5
C(10)-C(11)-C(12)	119.1(2)	C(12)-C(11)-H(11)	120.5
C(11)-C(12)-Bi(2)	118.49(17)	C(11)-C(12)-C(13)	120.1(2)

C(13)-C(12)-Bi(2)	121.28(16)	O(1)-C(13)-C(8)	123.1(2)
O(1)-C(13)-C(12)	115.26(19)	C(8)-C(13)-C(12)	121.6(2)
C(7)-C(14)-H(14A)	109.5	C(7)-C(14)-H(14B)	109.5
C(7)-C(14)-H(14C)	109.5	H(14A)-C(14)-H(14B)	109.5
H(14A)-C(14)-H(14C)	109.5	H(14B)-C(14)-H(14C)	109.5
C(7)-C(15)-H(15A)	109.5	C(7)-C(15)-H(15B)	109.5
C(7)-C(15)-H(15C)	109.5	H(15A)-C(15)-H(15B)	109.5
H(15A)-C(15)-H(15C)	109.5	H(15B)-C(15)-H(15C)	109.5
C(17A)-C(16A)-Bi(1)	121.7(4)	C(17A)-C(16A)-C(21A)	122.5(6)
C(21A)-C(16A)-Bi(1)	115.7(4)	C(17B)-C(16B)-Bi(1)	119.7(6)
C(21B)-C(16B)-Bi(1)	116.8(7)	C(21B)-C(16B)-C(17B)	123.5(9)
C(16A)-C(17A)-H(17A)	121.2	C(16A)-C(17A)-C(18A)	117.5(5)
C(18A)-C(17A)-H(17A)	121.2	C(16B)-C(17B)-H(17B)	121.6
C(18B)-C(17B)-C(16B)	116.8(7)	C(18B)-C(17B)-H(17B)	121.6
C(17A)-C(18A)-H(18A)	119.4	C(19A)-C(18A)-C(17A)	121.2(5)
C(19A)-C(18A)-H(18A)	119.4	C(17B)-C(18B)-H(18B)	120.2
C(19B)-C(18B)-C(17B)	119.6(8)	C(19B)-C(18B)-H(18B)	120.2
C(18A)-C(19A)-H(19A)	119.6	C(18A)-C(19A)-C(20A)	120.9(5)
C(20A)-C(19A)-H(19A)	119.6	C(18B)-C(19B)-H(19B)	119.3
C(20B)-C(19B)-C(18B)	121.4(9)	C(20B)-C(19B)-H(19B)	119.3
C(19A)-C(20A)-H(20A)	119.9	C(19A)-C(20A)-C(21A)	120.2(6)
C(21A)-C(20A)-H(20A)	119.9	C(19B)-C(20B)-H(20B)	120.1
C(21B)-C(20B)-C(19B)	119.7(8)	C(21B)-C(20B)-H(20B)	120.1
C(16A)-C(21A)-C(20A)	117.7(6)	C(16A)-C(21A)-H(21A)	121.2
C(20A)-C(21A)-H(21A)	121.2	C(16B)-C(21B)-C(20B)	118.9(9)
C(16B)-C(21B)-H(21B)	120.5	C(20B)-C(21B)-H(21B)	120.5
C(23A)-C(22A)-Bi(1)	114.4(4)	C(23A)-C(22A)-C(27A)	124.1(6)
C(27A)-C(22A)-Bi(1)	121.5(5)	C(23B)-C(22B)-Bi(1)	114.6(9)
C(23B)-C(22B)-C(27B)	122.2(10)	C(27B)-C(22B)-Bi(1)	122.7(7)
C(22A)-C(23A)-H(23A)	121.4	C(22A)-C(23A)-C(24A)	117.2(7)
C(24A)-C(23A)-H(23A)	121.4	C(22B)-C(23B)-H(23B)	121.3
C(22B)-C(23B)-C(24B)	117.4(11)	C(24B)-C(23B)-H(23B)	121.3
C(23A)-C(24A)-H(24A)	119.7	C(25A)-C(24A)-C(23A)	120.6(8)
C(25A)-C(24A)-H(24A)	119.7	C(23B)-C(24B)-H(24B)	120.7
C(25B)-C(24B)-C(23B)	118.7(13)	C(25B)-C(24B)-H(24B)	120.7
C(24A)-C(25A)-H(25A)	119.7	C(24A)-C(25A)-C(26A)	120.7(6)
C(26A)-C(25A)-H(25A)	119.7	C(24B)-C(25B)-H(25B)	118.8
C(24B)-C(25B)-C(26B)	122.4(13)	C(26B)-C(25B)-H(25B)	118.8
C(25A)-C(26A)-H(26A)	120.2	C(25A)-C(26A)-C(27A	)119.7(5)

C(27A)-C(26A)-H(26A)	120.2	C(25B)-C(26B)-H(26B)	119.2
C(25B)-C(26B)-C(27B)	121.7(11)	C(27B)-C(26B)-H(26B)	119.2
C(22A)-C(27A)-C(26A)	117.6(7)	C(22A)-C(27A)-H(27A)	121.2
C(26A)-C(27A)-H(27A)	121.2	C(22B)-C(27B)-H(27B)	121.3
C(26B)-C(27B)-C(22B)	117.3(8)	C(26B)-C(27B)-H(27B)	121.3
C(29)-C(28)-Bi(2)	121.6(2)	C(29)-C(28)-C(33)	122.6(3)
C(33)-C(28)-Bi(2)	115.8(2)	C(28)-C(29)-H(29)	121.2
C(28)-C(29)-C(30)	117.6(3)	C(30)-C(29)-H(29)	121.2
C(29)-C(30)-H(30)	119.5	C(31)-C(30)-C(29)	121.1(3)
C(31)-C(30)-H(30)	119.5	C(30)-C(31)-H(31)	119.9
C(30)-C(31)-C(32)	120.3(3)	C(32)-C(31)-H(31)	119.9
C(31)-C(32)-H(32)	120.0	C(31)-C(32)-C(33)	120.0(3)
C(33)-C(32)-H(32)	120.0	C(28)-C(33)-C(32)	118.4(3)
C(28)-C(33)-H(33)	120.8	C(32)-C(33)-H(33)	120.8
C(35A)-C(34A)-Bi(2)	116.7(5)	C(39A)-C(34A)-Bi(2)	123.0(5)
C(39A)-C(34A)-C(35A)	120.4(6)	C(35B)-C(34B)-Bi(2)	113.2(8)
C(35B)-C(34B)-C(39B)	126.2(10)	C(39B)-C(34B)-Bi(2)	120.5(7)
C(34A)-C(35A)-H(35A)	120.8	C(36A)-C(35A)-C(34A)	118.4(6)
C(36A)-C(35A)-H(35A)	120.8	C(34B)-C(35B)-H(35B)	121.8
C(34B)-C(35B)-C(36B)	116.3(9)	C(36B)-C(35B)-H(35B)	121.8
C(35A)-C(36A)-H(36A)	119.4	C(37A)-C(36A)-C(35A	)121.1(6)
C(37A)-C(36A)-H(36A)	119.4	C(35B)-C(36B)-H(36B)	119.2
C(37B)-C(36B)-C(35B)	121.7(9)	C(37B)-C(36B)-H(36B)	119.2
C(36A)-C(37A)-H(37A)	120.2	C(38A)-C(37A)-C(36A)	119.7(5)
C(38A)-C(37A)-H(37A)	120.2	C(36B)-C(37B)-H(37B)	119.7
C(36B)-C(37B)-C(38B)	120.6(10)	C(38B)-C(37B)-H(37B)	119.7
C(37A)-C(38A)-H(38A)	119.5	C(37A)-C(38A)-C(39A)	120.9(5)
C(39A)-C(38A)-H(38A)	119.5	C(37B)-C(38B)-H(38B)	120.0
C(37B)-C(38B)-C(39B)	120.0(9)	C(39B)-C(38B)-H(38B)	120.0
C(34A)-C(39A)-C(38A)	119.5(5)	C(34A)-C(39A)-H(39A)	120.2
C(38A)-C(39A)-H(39A)	120.2	C(34B)-C(39B)-C(38B)	115.1(9)
C(34B)-C(39B)-H(39B)	122.5	C(38B)-C(39B)-H(39B)	122.5
F(4A)-Sb(1A)-F(5A)	91.8(10)	F(4A)-Sb(1A)-F(6A)	95.6(8)
F(4A)-Sb(1A)-F(7A)	90.4(11)	F(4A)-Sb(1A)-F(8A)	91.0(8)
F(4A)-Sb(1A)-F(9A)	174.5(12)	F(5A)-Sb(1A)-F(7A)	177.3(9)
F(6A)-Sb(1A)-F(5A)	91.3(6)	F(6A)-Sb(1A)-F(7A)	90.1(6)
F(8A)-Sb(1A)-F(5A)	88.1(6)	F(8A)-Sb(1A)-F(6A)	173.4(8)
F(8A)-Sb(1A)-F(7A)	90.3(6)	F(9A)-Sb(1A)-F(5A)	90.8(10)
F(9A)-Sb(1A)-F(6A)	89.2(10)	F(9A)-Sb(1A)-F(7A)	86.9(11)

F(9A)-Sb(1A)-F(8A)	84.2(11)	F(5B)-Sb(1B)-F(4B)	88.2(11)
F(5B)-Sb(1B)-F(6B)	89.1(7)	F(5B)-Sb(1B)-F(8B)	92.6(7)
F(5B)-Sb(1B)-F(9B)	89.9(12)	F(6B)-Sb(1B)-F(4B)	83.1(8)
F(6B)-Sb(1B)-F(8B)	171.1(9)	F(6B)-Sb(1B)-F(9B)	92.5(11)
F(7B)-Sb(1B)-F(4B)	88.1(12)	F(7B)-Sb(1B)-F(5B)	175.6(11)
F(7B)-Sb(1B)-F(6B)	88.1(8)	F(7B)-Sb(1B)-F(8B)	89.7(8)
F(7B)-Sb(1B)-F(9B)	93.6(13)	F(8B)-Sb(1B)-F(4B)	88.3(9)
F(9B)-Sb(1B)-F(4B)	175.3(12)	F(9B)-Sb(1B)-F(8B)	96.2(12)
Cl(1)-C(40)-H(40A)	105.9	Cl(1)-C(40)-H(40B)	105.9
Cl(2)-C(40)-Cl(1)	125.9(7)	Cl(2)-C(40)-H(40A)	105.9
Cl(2)-C(40)-H(40B)	105.9	H(40A)-C(40)-H(40B)	106.2
Cl(3A)-C(41A)-H(41C)	110.2	Cl(3A)-C(41A)-H(41D)	110.2
Cl(4A)-C(41A)-Cl(3A)	107.7(6)	Cl(4A)-C(41A)-H(41C)	110.2
Cl(4A)-C(41A)-H(41D)	110.2	H(41C)-C(41A)-H(41D)	108.5
Cl(3B)-C(41B)-Cl(4B)	113.6(8)	Cl(3B)-C(41B)-H(41A)	108.8
Cl(3B)-C(41B)-H(41B)	108.8	Cl(4B)-C(41B)-H(41A)	108.8
Cl(4B)-C(41B)-H(41B)	108.8	H(41A)-C(41B)-H(41B)	107.7

Symmetry transformations used to generate equivalent atoms:

#1 -x+1,-y+1,-z+1 #2 -x+2,-y,-z+1

Ligand bending

The xanthene backbone is featuring two different conformations in structure **3** (bent) and structure **4** (planar). To see the potential influence of the anion towards the xanthene backbone a database (CSD Version 5.41, November 2019) survey was performed on 19th February 2022 in a search for related structures. The following search motive was used in ConQuest (Version 2020.1) program:

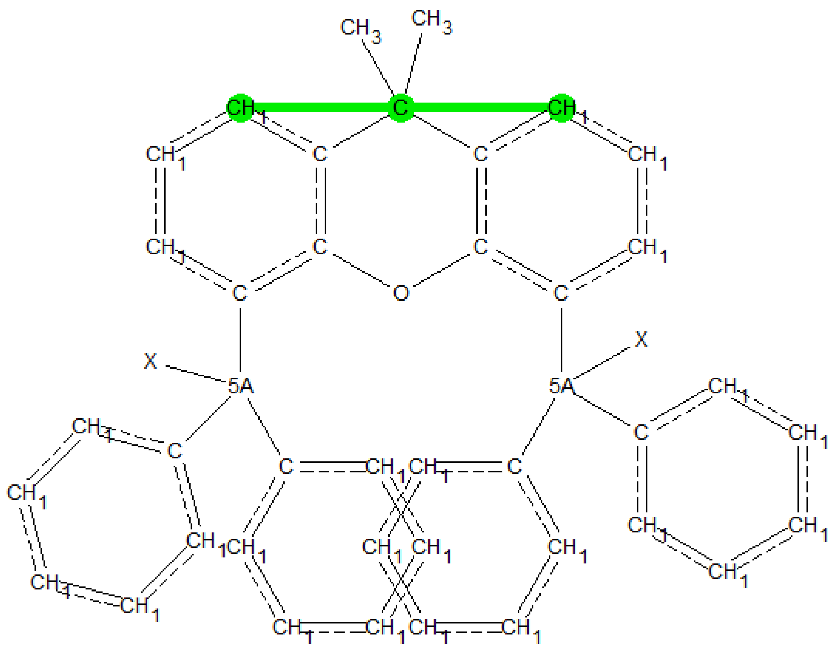

The shown Ar–C–Ar angle was used as a 3D parameter, to compare the found conformations of the xanthene backbone. The initial search found 59 structures containing the predefined xanthene-aryl-pnictogen-halogen entity.

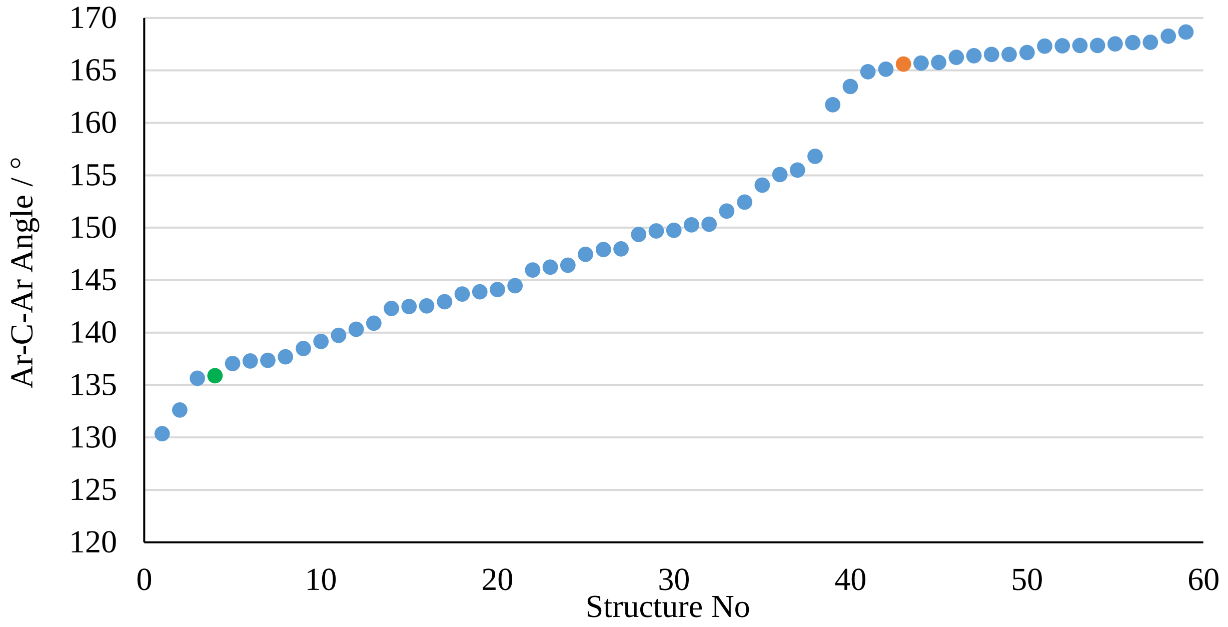

Figure S56. Plot of Ar–C–Ar angles found in the database survey. Green dot indicates the position of structure **3**, red dot indicates the position of structure **4**.

It could be shown, that the xanthene backbone of **3** is strongly bended in the presence of BAr^F as a counter-ion. An Ar–C–Ar angle of 135.89° is one of the most bent xanthene backbones in the whole dataset (most bent is MUWJEV: 130.374°). The survey showed that the bending angle of structure **4** with 165.63° is flat, but not unusually flat (most flattened is XUKBAJ: 168.686°).

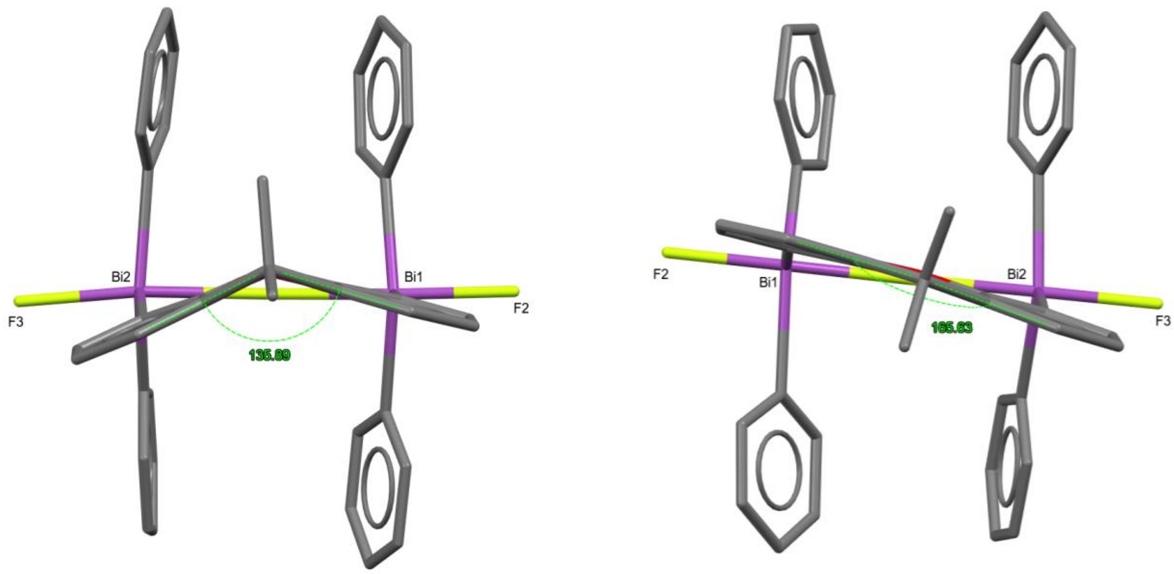

Figure S57. Comparison of xanthene backbone conformations of complex **3** (left) and **4** (right). Structural investigation have been made for main components of disordered parts.

Anion interaction

In comparison, slightly differences in complex **3** and **4** could be identified. The sterically more demanding BAr^F anion in **3** adopts a larger distance to the Bi(V) centers (Bi1–B1 distance is 10.717 Å and Bi2–B1 distance is 9.926 Å). The smaller SbF_6 anion comes in a closer distance (Bi1–Sb1A distance is 6.177 Å and Bi2–Sb1A distance is 6.082 Å) to the Bi(V) centers.

In both cases, the F atoms (shown as centroids) are pointing towards the Bi(V) centers with distances of 5.304 Å and 5.235 Å in **3** as well as 4.889 Å and 4.956 Å in **4**. The found distances are too big to have a coordinative effect onto the Bi(V) central unit. No short contacts (contact < sum of vdW radii) between the F atoms of the anion and the central Bi atoms of the cationic unit could be found.

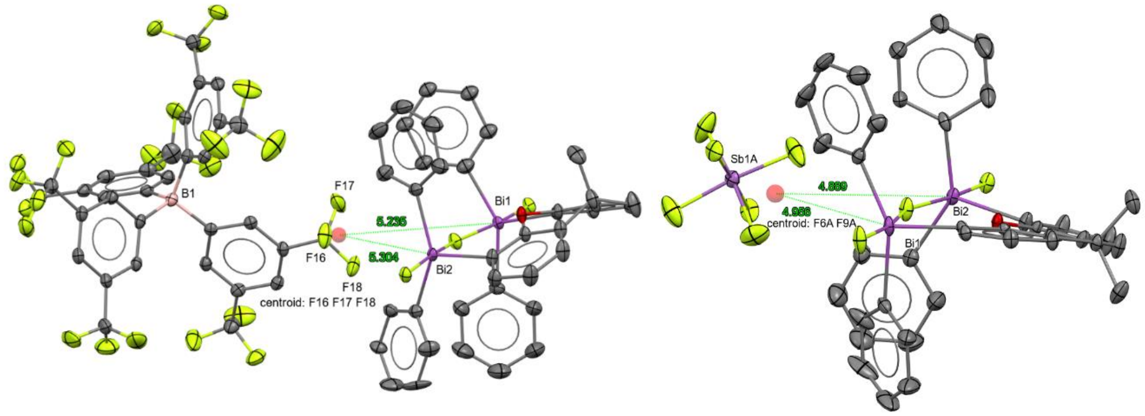

Figure S58. Comparison of distances between Bi(V) centers and corresponding anions of **3** (left) and **4** (right). Ellipsoids are drawn at the 50% probability level.

Single crystal structure analysis of complex 9 (13784)

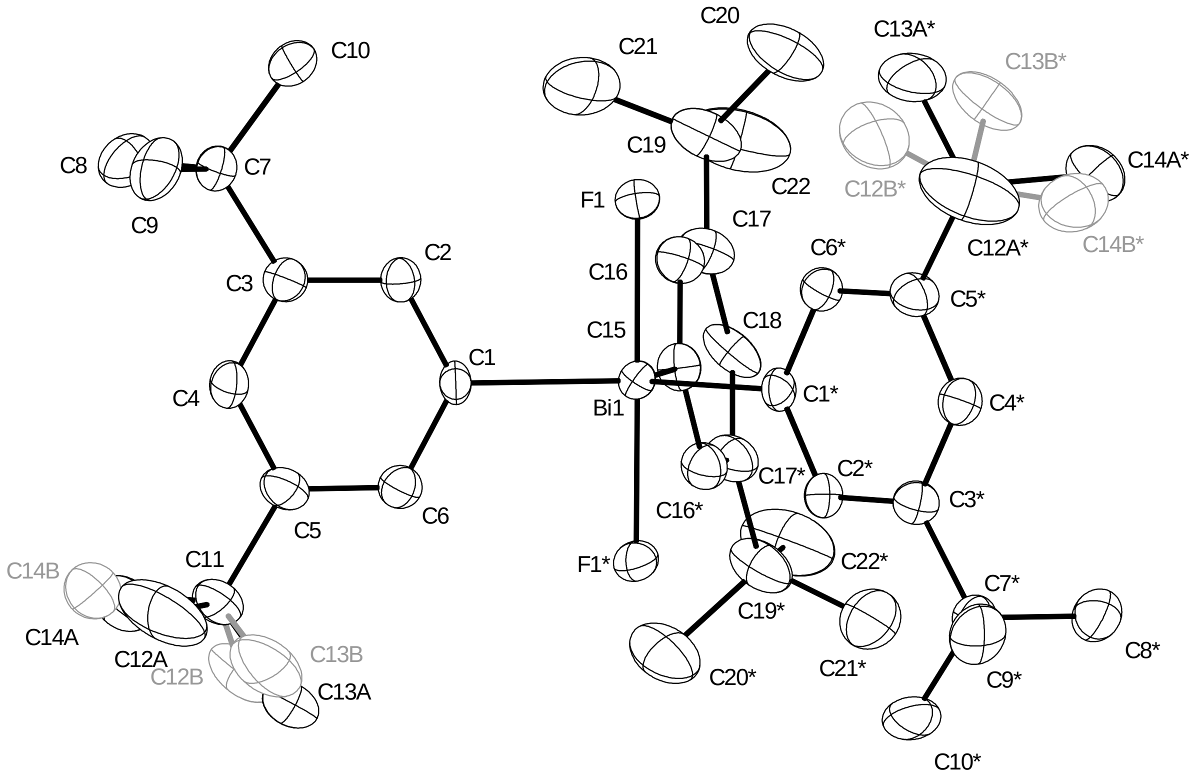

Figure S59. The molecular structure of complex **9**. H atoms have been removed for clarity. Disordered parts shown in gray. Ellipsoids are drawn at the 50% probability level.

X-ray Crystal Structure Analysis of complex 9:
C_{42} H_{63} Bi F_2, M_r = 814.90 g mol^{-1}, colorless plate, crystal size 0.24 x 0.22 x 0.04 mm^3, orthorhombic, space group *F*dd2 [43], a = 20.1325(11) Å, b = 38.257(3) Å, c = 10.5641(7) Å, V = 8136.6(10) Å^3, T = 200(2) K, Z = 8, D_{calc} = 1.330 g·cm^3, λ = 0.71073 Å, $\mu(Mo\text{-}K_\alpha)$ = 4.368 mm^{-1}, Gaussian absorption correction ($T_{\min}$ = 0.40587, $T_{\max}$ = 0.77245), Bruker AXS Enraf-Nonius KappaCCD diffractometer with a FR591 rotating Mo-anode X-ray source, 2.700 < θ < 33.074°, 21701 measured reflections, 7273 independent reflections, 5745 reflections with I > 2σ(I), R_{int} = 0.0579. The structure was solved by *SHELXS* and refined by full-matrix least-squares (*SHELXL*) against F^2 to R_1 = 0.0361 [I > 2σ(I)], wR_2 = 0.0785 [all data], 245 parameters and 37 restraints.

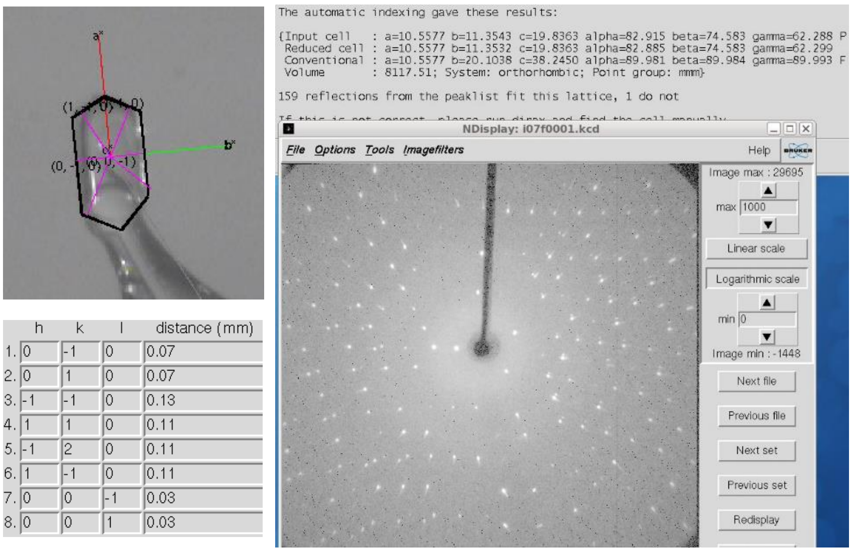

Figure S60. Crystal faces and unit cell determination/refinement of complex **9**.

```
INTENSITY STATISTICS FOR DATASET
```

Resolution	#Data	#Theory	%Complete	Redundancy	Mean I	Mean I/s	Rmerge	Rsigma
Inf - 2.76	63	68	92.6	6.82	141.55	36.04	0.0641	0.0334
2.76 - 1.84	146	146	100.0	6.98	95.18	35.83	0.0536	0.0258
1.84 - 1.44	210	210	100.0	7.28	63.37	33.82	0.0491	0.0255
1.44 - 1.25	213	213	100.0	6.96	44.63	30.82	0.0473	0.0270
1.25 - 1.13	207	207	100.0	6.84	32.14	28.74	0.0614	0.0289
1.13 - 1.05	206	206	100.0	6.36	29.74	27.61	0.0592	0.0305
1.05 - 0.98	238	238	100.0	6.19	23.24	23.86	0.0621	0.0334
0.98 - 0.93	205	205	100.0	5.98	19.44	22.44	0.0618	0.0364
0.93 - 0.89	195	195	100.0	5.71	17.54	20.92	0.0659	0.0393
0.89 - 0.85	246	246	100.0	5.38	13.74	18.10	0.0750	0.0461
0.85 - 0.82	214	214	100.0	5.21	12.28	16.88	0.0868	0.0505
0.82 - 0.80	153	153	100.0	5.02	10.52	15.17	0.0891	0.0571
0.80 - 0.77	273	273	100.0	4.87	9.79	14.39	0.1034	0.0625
0.77 - 0.75	201	201	100.0	4.57	7.79	11.46	0.1270	0.0816
0.75 - 0.73	229	229	100.0	4.49	7.61	10.90	0.1337	0.0880
0.73 - 0.71	237	240	98.8	4.34	6.89	9.52	0.1561	0.1054
0.71 - 0.70	156	156	100.0	4.12	6.16	7.82	0.1765	0.1276
0.70 - 0.68	294	298	98.7	4.13	5.48	6.88	0.1958	0.1514
0.68 - 0.67	142	146	97.3	4.03	4.78	5.43	0.2033	0.1957
0.67 - 0.66	176	182	96.7	3.74	4.36	4.53	0.2426	0.2407
0.66 - 0.65	167	167	100.0	3.69	3.69	3.42	0.2623	0.3215
0.75 - 0.65	1401	1418	98.8	4.11	5.72	7.23	0.1797	0.1508
Inf - 0.65	4171	4193	99.5	5.32	21.87	17.72	0.0667	0.0436

The studied crystal appears to be twinned by inversion (TWIN -1 0 0 0 -1 0 0 0 -1) with an occupancy of ca. 3.5%. One terminal *tert*-butyl group at *meta*-position of the aryl ligand is rotationally disordered over two positions with occupancy of 50:50%. Thermal displacement parameters of this sub-unit where treated with ISOR instruction (ISOR 0.01 0.02 C14A C13B C13A C12B C12A C14B). Complete .cif-data of the compound are available under the CCDC number **CCDC-2154888**.

Supramolecular features and coordination geometry

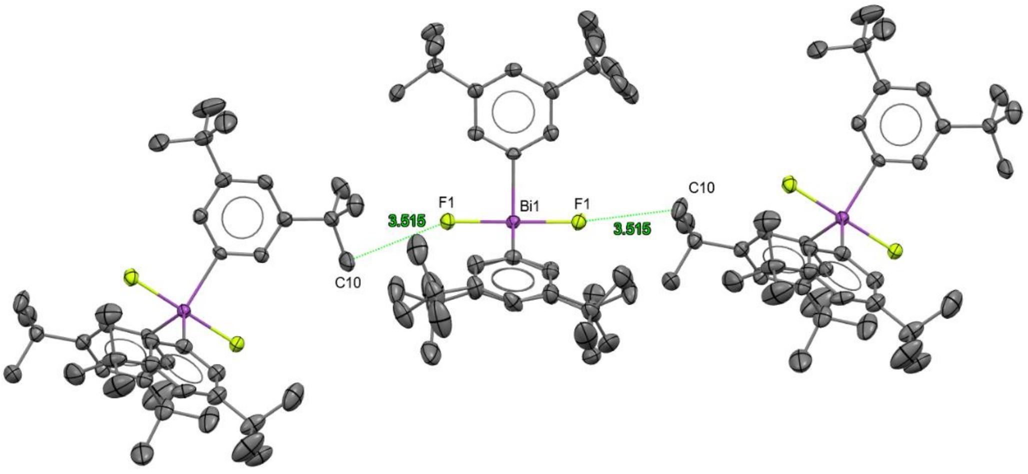

Figure S61. Neutral molecules of complex **9** do not undergo F–Bi···F–Bi interactions in the solid state. Ellipsoids are drawn at the 50% probability level.

The neutral complex **9** does not undergo significant intermolecular interactions F–Bi···F–Bi leading to a truly monomeric structure in solid state. Both terminal F atoms are surrounded by *tert-butyl* units of two neighboring molecules with a closest F1–C10 distance of 3.515 Å.

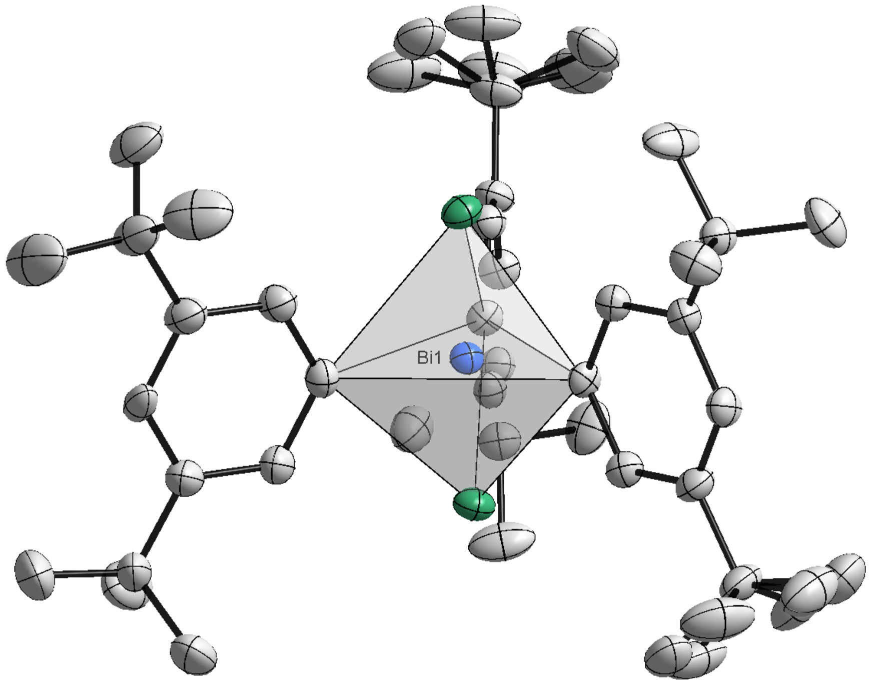

Figure S62. Polyhedral representation of trigonal bipyramidal coordination sphere of complex **9**. Ellipsoids are drawn at the 50% probability level.

The absence of F–Bi···F–Bi interaction lead to a nearly perfect trigonal bipyramidal coordination sphere.

Ligand torsion of aryl rings

Complex **9** is featuring a unique conformation of aryl substituents. The aromatic ring planes are almost parallel to the central F–Bi–F axis with small torsion angles. This geometry is comparable to a paddle wheel like complex. The driving force here might be the steric influence of sterically demanding *tert*-butyl groups in *meta*-position of the aromatic rings as well as week C–H···F interactions.

A different geometry could be found in complex **10**. The mesityl ligands show a larger torsion angle and are not in parallel to the F–Bi–F central axis. This lead to propeller like complex structure wich is compatible to other similar substituted Aryl-Bi(V)-difluoride structures found in the database.

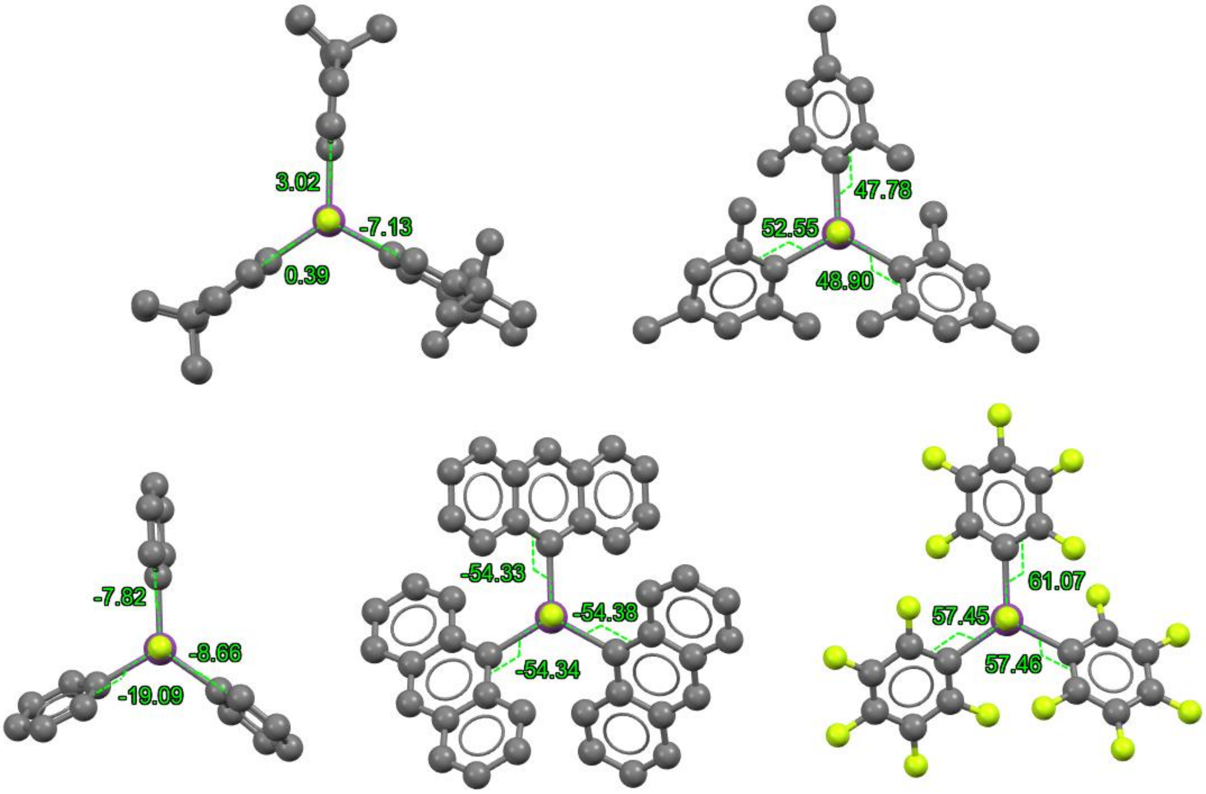

Figure S63. Comparison of ligand torsion angles of complex **9** and **10** (top left and right) with other Aryl-Bi(V)- difluorides (bottom left: XULFUF01, middle: EGESAK, right: SETNOU).

Table S5. Crystal data and structure refinement of complex **9**.

Identification code	13784	
Empirical formula	$C_{42} H_{63} Bi F_2$	
Color	colourless	
Formula weight	814.90 g·mol^{-1}	
Temperature	200(2) K	
Wavelength	0.71073 Å	
Crystal system	Orthorhombic	
Space group	*F*dd2, (no. 43)	
Unit cell dimensions	a = 20.1325(11) Å	α= 90°.
	b = 38.257(3) Å	β= 90°.
	c = 10.5641(7) Å	γ = 90°.
Volume	8136.6(10) Å^3	
Z	8	
Density (calculated)	1.330 Mg·m^{-3}	
Absorption coefficient	4.368 mm^{-1}	
F(000)	3328 e	
Crystal size	0.24 x 0.22 x 0.04 mm^3	
θ range for data collection	2.700 to 33.074°.	
Index ranges	$-29 \leq h \leq 30$, $-58 \leq k \leq 58$, $-15 \leq l \leq 16$	
Reflections collected	21701	
Independent reflections	7273 [R_{int} = 0.0579]	
Reflections with $I>2\sigma(I)$	5745	
Completeness to θ = 25.242°	99.8 %	
Absorption correction	Gaussian	
Max. and min. transmission	0.77245 and 0.40587	
Refinement method	Full-matrix least-squares on F^2	
Data / restraints / parameters	7273 / 37 / 245	
Goodness-of-fit on F^2	1.038	
Final R indices [$I>2\sigma(I)$]	R_1 = 0.0361	wR^2 = 0.0732
R indices (all data)	R_1 = 0.0571	wR^2 = 0.0785
Absolute structure parameter	0.034(13)	
Extinction coefficient	n/a	
Largest diff. peak and hole	1.816 and -1.381 e·Å^{-3}	

Table S6. Bond lengths [Å] and angles [°] of complex **9**.

Bi(1)-F(1)#1	2.129(3)	Bi(1)-F(1)	2.129(3)
Bi(1)-C(1)	2.209(5)	Bi(1)-C(1)#1	2.209(5)
Bi(1)-C(15)	2.192(8)	C(1)-C(2)	1.384(6)
C(1)-C(6)	1.391(8)	C(2)-H(2)	0.9500
C(2)-C(3)	1.388(7)	C(3)-C(4)	1.399(8)
C(3)-C(7)	1.550(7)	C(4)-H(4)	0.9500
C(4)-C(5)	1.399(8)	C(5)-C(6)	1.389(9)
C(5)-C(11)	1.549(9)	C(6)-H(6)	0.9500
C(7)-C(8)	1.534(9)	C(7)-C(9)	1.531(9)
C(7)-C(10)	1.527(8)	C(8)-H(8A)	0.9800
C(8)-H(8B)	0.9800	C(8)-H(8C)	0.9800
C(9)-H(9A)	0.9800	C(9)-H(9B)	0.9800
C(9)-H(9C)	0.9800	C(10)-H(10A)	0.9800
C(10)-H(10B)	0.9800	C(10)-H(10C)	0.9800
C(11)-C(12A)	1.42(2)	C(11)-C(12B)	1.590(19)
C(11)-C(13A)	1.522(19)	C(11)-C(13B)	1.453(19)
C(11)-C(14A)	1.60(2)	C(11)-C(14B)	1.49(2)
C(12A)-H(12A)	0.9800	C(12A)-H(12B)	0.9800
C(12A)-H(12C)	0.9800	C(12B)-H(12D)	0.9800
C(12B)-H(12E)	0.9800	C(12B)-H(12F)	0.9800
C(13A)-H(13A)	0.9800	C(13A)-H(13B)	0.9800
C(13A)-H(13C)	0.9800	C(13B)-H(13D)	0.9800
C(13B)-H(13E)	0.9800	C(13B)-H(13F)	0.9800
C(14A)-H(14A)	0.9800	C(14A)-H(14B)	0.9800
C(14A)-H(14C)	0.9800	C(14B)-H(14D)	0.9800
C(14B)-H(14E)	0.9800	C(14B)-H(14F)	0.9800
C(15)-C(16)	1.393(7)	C(15)-C(16)#1	1.393(7)
C(16)-H(16)	0.9500	C(16)-C(17)	1.398(8)
C(17)-C(18)	1.404(8)	C(17)-C(19)	1.523(9)
C(18)-H(18)	0.9500	C(19)-C(20)	1.537(10)
C(19)-C(21)	1.581(13)	C(19)-C(22)	1.519(11)
C(20)-H(20A)	0.9800	C(20)-H(20B)	0.9800
C(20)-H(20C)	0.9800	C(21)-H(21A)	0.9800
C(21)-H(21B)	0.9800	C(21)-H(21C)	0.9800
C(22)-H(22A)	0.9800	C(22)-H(22B)	0.9800
C(22)-H(22C)	0.9800		

F(1)#1-Bi(1)-F(1)	179.1(2)	F(1)-Bi(1)-C(1)#1	89.77(16)
F(1)-Bi(1)-C(1)	89.80(16)	F(1)#1-Bi(1)-C(1)#1	89.79(16)
F(1)#1-Bi(1)-C(1)	89.77(16)	F(1)-Bi(1)-C(15)	90.46(11)
F(1)#1-Bi(1)-C(15)	90.46(11)	C(1)#1-Bi(1)-C(1)	124.5(3)
C(15)-Bi(1)-C(1)#1	117.77(13)	C(15)-Bi(1)-C(1)	117.77(13)
C(2)-C(1)-Bi(1)	118.3(4)	C(2)-C(1)-C(6)	122.9(5)
C(6)-C(1)-Bi(1)	118.8(4)	C(1)-C(2)-H(2)	120.5
C(1)-C(2)-C(3)	119.0(5)	C(3)-C(2)-H(2)	120.5
C(2)-C(3)-C(4)	118.3(5)	C(2)-C(3)-C(7)	122.6(5)
C(4)-C(3)-C(7)	119.1(5)	C(3)-C(4)-H(4)	118.6
C(3)-C(4)-C(5)	122.8(5)	C(5)-C(4)-H(4)	118.6
C(4)-C(5)-C(11)	120.5(6)	C(6)-C(5)-C(4)	118.1(5)
C(6)-C(5)-C(11)	121.3(5)	C(1)-C(6)-H(6)	120.5
C(5)-C(6)-C(1)	119.0(5)	C(5)-C(6)-H(6)	120.5
C(8)-C(7)-C(3)	109.0(5)	C(9)-C(7)-C(3)	110.4(5)
C(9)-C(7)-C(8)	108.5(5)	C(10)-C(7)-C(3)	111.7(5)
C(10)-C(7)-C(8)	109.3(5)	C(10)-C(7)-C(9)	108.0(5)
C(7)-C(8)-H(8A)	109.5	C(7)-C(8)-H(8B)	109.5
C(7)-C(8)-H(8C)	109.5	H(8A)-C(8)-H(8B)	109.5
H(8A)-C(8)-H(8C)	109.5	H(8B)-C(8)-H(8C)	109.5
C(7)-C(9)-H(9A)	109.5	C(7)-C(9)-H(9B)	109.5
C(7)-C(9)-H(9C)	109.5	H(9A)-C(9)-H(9B)	109.5
H(9A)-C(9)-H(9C)	109.5	H(9B)-C(9)-H(9C)	109.5
C(7)-C(10)-H(10A)	109.5	C(7)-C(10)-H(10B)	109.5
C(7)-C(10)-H(10C)	109.5	H(10A)-C(10)-H(10B)	109.5
H(10A)-C(10)-H(10C)	109.5	H(10B)-C(10)-H(10C)	109.5
C(5)-C(11)-C(12B)	105.7(9)	C(5)-C(11)-C(14A)	108.5(8)
C(12A)-C(11)-C(5)	111.1(10)	C(12A)-C(11)-C(13A)	112.9(16)
C(12A)-C(11)-C(14A)	109.0(16)	C(13A)-C(11)-C(5)	112.6(8)
C(13A)-C(11)-C(14A)	102.4(13)	C(13B)-C(11)-C(5)	110.6(8)
C(13B)-C(11)-C(12B)	107.5(13)	C(13B)-C(11)-C(14B)	109.9(14)
C(14B)-C(11)-C(5)	115.6(8)	C(14B)-C(11)-C(12B)	107.1(13)
C(11)-C(12A)-H(12A)	109.5	C(11)-C(12A)-H(12B)	109.5
C(11)-C(12A)-H(12C)	109.5	H(12A)-C(12A)-H(12B)	109.5
H(12A)-C(12A)-H(12C)	109.5	H(12B)-C(12A)-H(12C)	109.5
C(11)-C(12B)-H(12D)	109.5	C(11)-C(12B)-H(12E)	109.5
C(11)-C(12B)-H(12F)	109.5	H(12D)-C(12B)-H(12E)	109.5
H(12D)-C(12B)-H(12F)	109.5	H(12E)-C(12B)-H(12F)	109.5

C(11)-C(13A)-H(13A)	109.5	C(11)-C(13A)-H(13B)	109.5
C(11)-C(13A)-H(13C)	109.5	H(13A)-C(13A)-H(13B)	109.5
H(13A)-C(13A)-H(13C)	109.5	H(13B)-C(13A)-H(13C)	109.5
C(11)-C(13B)-H(13D)	109.5	C(11)-C(13B)-H(13E)	109.5
C(11)-C(13B)-H(13F)	109.5	H(13D)-C(13B)-H(13E)	109.5
H(13D)-C(13B)-H(13F)	109.5	H(13E)-C(13B)-H(13F)	109.5
C(11)-C(14A)-H(14A)	109.5	C(11)-C(14A)-H(14B)	109.5
C(11)-C(14A)-H(14C)	109.5	H(14A)-C(14A)-H(14B)	109.5
H(14A)-C(14A)-H(14C)	109.5	H(14B)-C(14A)-H(14C)	109.5
C(11)-C(14B)-H(14D)	109.5	C(11)-C(14B)-H(14E)	109.5
C(11)-C(14B)-H(14F)	109.5	H(14D)-C(14B)-H(14E)	109.5
H(14D)-C(14B)-H(14F)	109.5	H(14E)-C(14B)-H(14F)	109.5
C(16)#1-C(15)-Bi(1)	118.8(4)	C(16)-C(15)-Bi(1)	118.8(4)
C(16)-C(15)-C(16)#1	122.4(8)	C(15)-C(16)-H(16)	120.4
C(15)-C(16)-C(17)	119.2(6)	C(17)-C(16)-H(16)	120.4
C(16)-C(17)-C(18)	118.5(6)	C(16)-C(17)-C(19)	120.0(6)
C(18)-C(17)-C(19)	121.5(6)	C(17)#1-C(18)-C(17)	122.3(8)
C(17)#1-C(18)-H(18)	118.9	C(17)-C(18)-H(18)	118.9
C(17)-C(19)-C(20)	110.3(6)	C(17)-C(19)-C(21)	106.9(6)
C(20)-C(19)-C(21)	107.5(6)	C(22)-C(19)-C(17)	113.4(7)
C(22)-C(19)-C(20)	110.2(7)	C(22)-C(19)-C(21)	108.4(8)
C(19)-C(20)-H(20A)	109.5	C(19)-C(20)-H(20B)	109.5
C(19)-C(20)-H(20C)	109.5	H(20A)-C(20)-H(20B)	109.5
H(20A)-C(20)-H(20C)	109.5	H(20B)-C(20)-H(20C)	109.5
C(19)-C(21)-H(21A)	109.5	C(19)-C(21)-H(21B)	109.5
C(19)-C(21)-H(21C)	109.5	H(21A)-C(21)-H(21B)	109.5
H(21A)-C(21)-H(21C)	109.5	H(21B)-C(21)-H(21C)	109.5
C(19)-C(22)-H(22A)	109.5	C(19)-C(22)-H(22B)	109.5
C(19)-C(22)-H(22C)	109.5	H(22A)-C(22)-H(22B)	109.5
H(22A)-C(22)-H(22C)	109.5	H(22B)-C(22)-H(22C)	109.5

_

Symmetry transformations used to generate equivalent atoms:

#1 -x+1/2,-y+1/2,z

Single crystal structure analysis of complex 10 (13813)

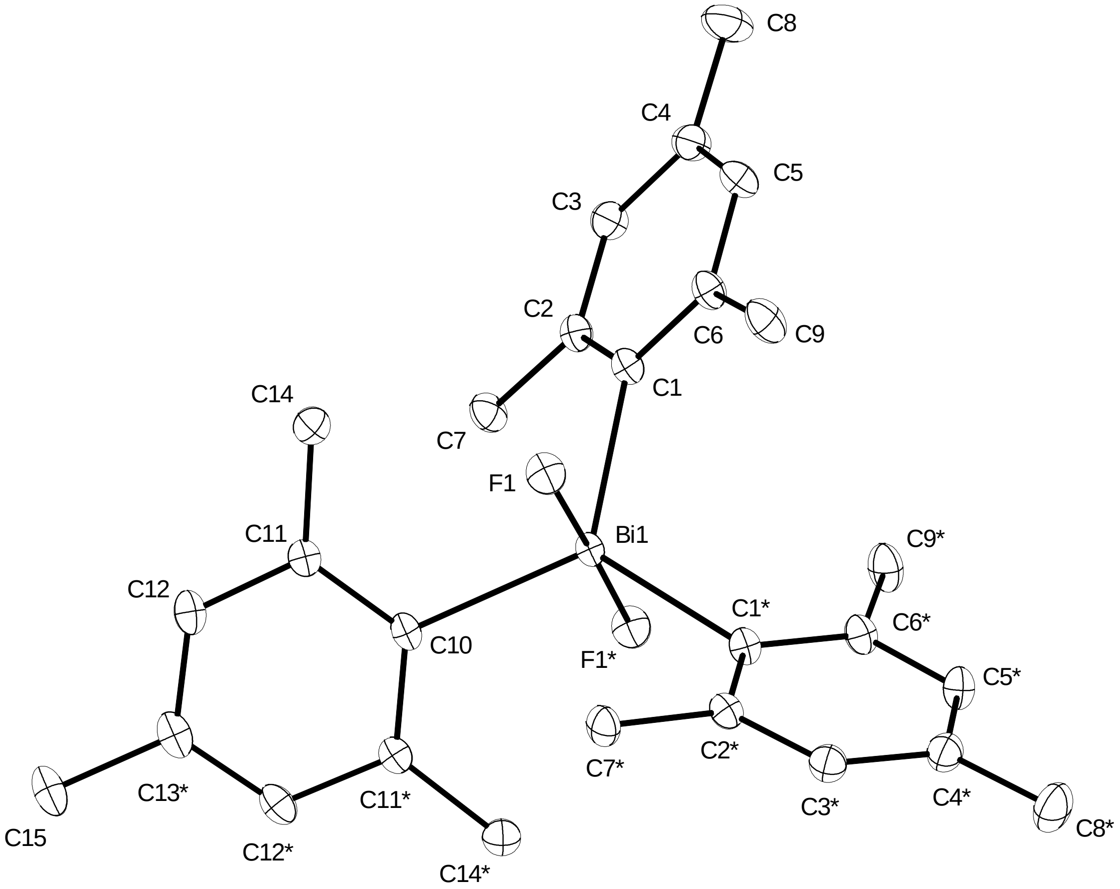

Figure S64. The molecular structure of complex **10**. H atoms have been removed for clarity. Ellipsoids are drawn at the 50% probability level.

X-ray Crystal Structure Analysis of complex 10:

C_{27} H_{33} Bi F_2, M_r = 604.51 g mol^{-1}, colorless plate, crystal size 0.18 x 0.14 x 0.07 mm^3, monoclinic, space group *C*2/c [15], *a* = 10.4325(8) Å, *b* = 12.7520(9) Å, *c* = 18.044(2) Å, *V* = 2322.8(4) Å^3, *T* = 100(2) K, *Z* = 4, D_{calc} = 1.729 g·cm^3, λ = 0.71073 Å, $\mu(Mo\text{-}K_\alpha)$ = 7.617 mm^{-1}, Gaussian absorption correction ($T_{\min}$ = 0.27610, $T_{\max}$ = 0.61971), Bruker AXS Enraf-Nonius KappaCCD diffractometer with a FR591 rotating Mo-anode X-ray source, 2.607 < θ < 33.116°, 24864 measured reflections, 4428 independent reflections, 4194 reflections with $I > 2\sigma(I)$, R_{int} = 0.0279. The structure was solved by *SHELXS* and refined by full-matrix least-squares (*SHELXL*) against F^2 to R_1 = 0.0148 [$I > 2\sigma(I)$], wR_2 = 0.0338 [all data], 143 parameters.

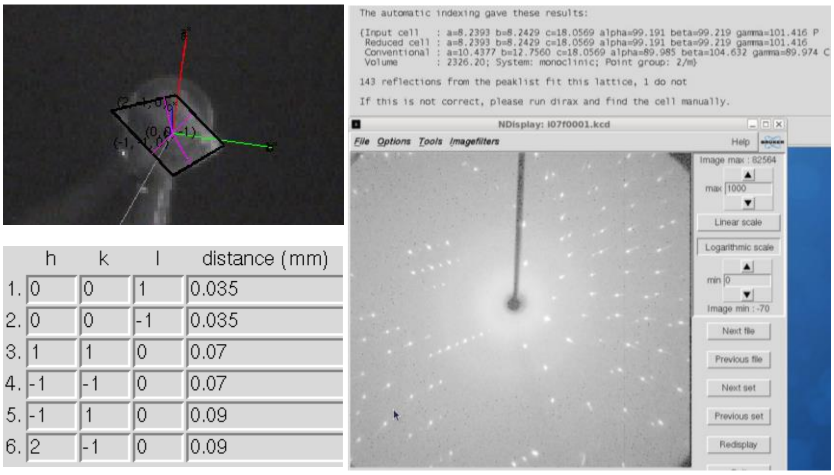

Figure S65. Crystal faces and unit cell determination/refinement of complex **10**.

INTENSITY STATISTICS FOR DATASET

Resolution	#Data	#Theory	%Complete	Redundancy	Mean I	Mean I/s	Rmerge	Rsigma
Inf - 2.67	69	72	95.8	11.24	175.03	100.53	0.0250	0.0089
2.67 - 1.80	161	161	100.0	8.48	132.14	85.55	0.0228	0.0099
1.80 - 1.41	233	233	100.0	7.67	103.42	79.46	0.0247	0.0108
1.41 - 1.23	241	241	100.0	7.28	73.59	70.18	0.0242	0.0119
1.23 - 1.12	222	222	100.0	6.85	57.23	63.03	0.0214	0.0132
1.12 - 1.03	241	241	100.0	6.71	54.76	59.17	0.0220	0.0137
1.03 - 0.97	252	252	100.0	6.21	51.70	55.78	0.0227	0.0151
0.97 - 0.92	218	218	100.0	6.13	39.34	49.14	0.0255	0.0164
0.92 - 0.88	246	246	100.0	5.73	38.38	46.79	0.0273	0.0177
0.88 - 0.85	196	196	100.0	5.39	31.44	39.63	0.0319	0.0201
0.85 - 0.82	224	224	100.0	5.38	29.73	40.33	0.0320	0.0214
0.82 - 0.79	281	281	100.0	5.04	27.76	35.95	0.0332	0.0232
0.79 - 0.77	196	196	100.0	4.83	22.59	31.74	0.0398	0.0262
0.77 - 0.75	213	213	100.0	4.78	24.23	32.44	0.0390	0.0268
0.75 - 0.73	273	273	100.0	4.53	21.03	28.16	0.0443	0.0305
0.73 - 0.71	274	274	100.0	4.35	18.97	25.51	0.0490	0.0341
0.71 - 0.70	149	149	100.0	4.22	20.27	25.89	0.0508	0.0346
0.70 - 0.68	333	333	100.0	4.08	16.53	21.32	0.0548	0.0426
0.68 - 0.67	181	181	100.0	4.13	15.21	19.57	0.0636	0.0471
0.67 - 0.66	186	186	100.0	3.81	13.47	16.42	0.0658	0.0570
0.66 - 0.65	205	205	100.0	3.77	14.30	16.58	0.0689	0.0592
0.75 - 0.65	1601	1601	100.0	4.15	17.27	22.26	0.0539	0.0411
Inf - 0.65	4594	4597	99.9	5.54	41.36	42.74	0.0279	0.0183

The molecule is located on a crystallographic special position (two-fold rotational axis) and a terminal CH_3-goup has a disorder by symmetry with 50:50% occupancy. Complete .cif-data of the compound are available under the CCDC number **CCDC-2154890.**

Supramolecular features and coordination

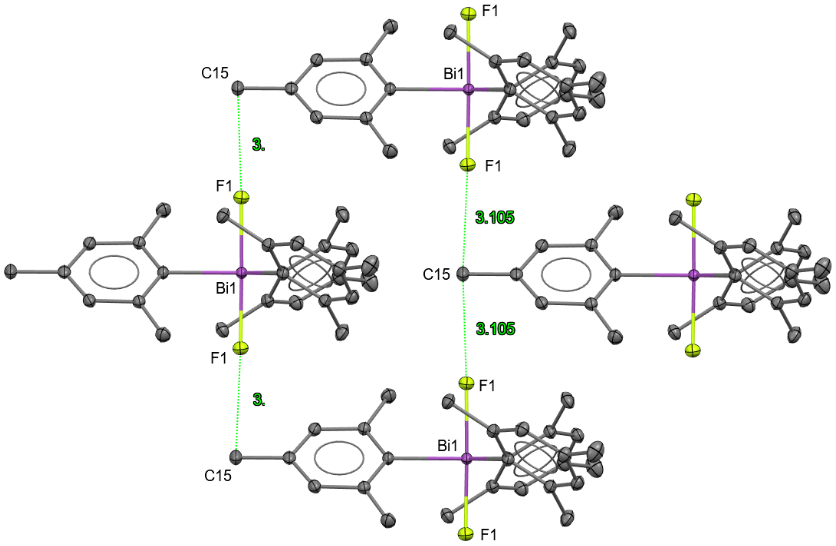

Figure S66. Neutral molecules of complex **10** do not undergo F–Bi···F–Bi interactions in the solid state. Ellipsoids are drawn at the 50% probability level.

The neutral complex **10** does not undergo significant intermolecular interactions F–Bi···F–Bi leading to a truly monomeric structure in solid state. Methyl units of two neighboring molecules with a closest F1···C15 distance of 3.105 Å surround both terminal F atoms.

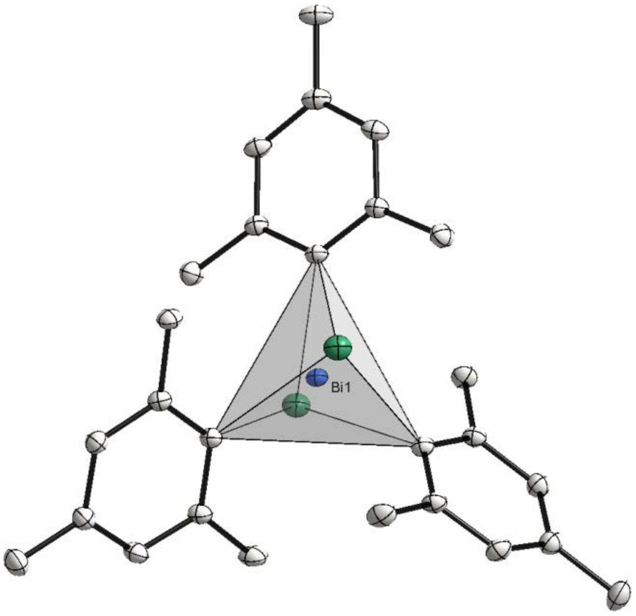

Figure S67. Polyhedral representation of trigonal bipyramidal coordination sphere of complex **10**. Ellipsoids are drawn at the 50% probability level.

The absence of F–Bi···F–Bi interaction lead to a nearly perfect trigonal bipyramidal coordination sphere.

Comparison with other pnictogen compounds

A database search (CSD Version 5.41, November 2019; survey was performed on 19th February 2022) and led to two isostructural pnictogen compounds containing P and Sb as central atoms. All structures crystallize in monoclinic space group *C2/c* [no. 15] with 4 molecules in the unit cell. Most noticeable differences are the pnictogen-C (FAFNUV: P–C bond lengths 1.844 and 1.854 Å; FIQBEP: Sb–C bond lengths 2.125 and 2.126 Å; **10**: Bi-C bond length 2.214 Å) and pnictogen–F (FAFNUV: P–F bond length 1.673 Å; FIQBEP: Sb–F bond length 1.982 Å; **10**: Bi–F bond length 2.122 Å) distances. All structures containing pnictogen atoms of oxidation state (V) adopt a nearly perfect trigonal bipyramidal coordination sphere.

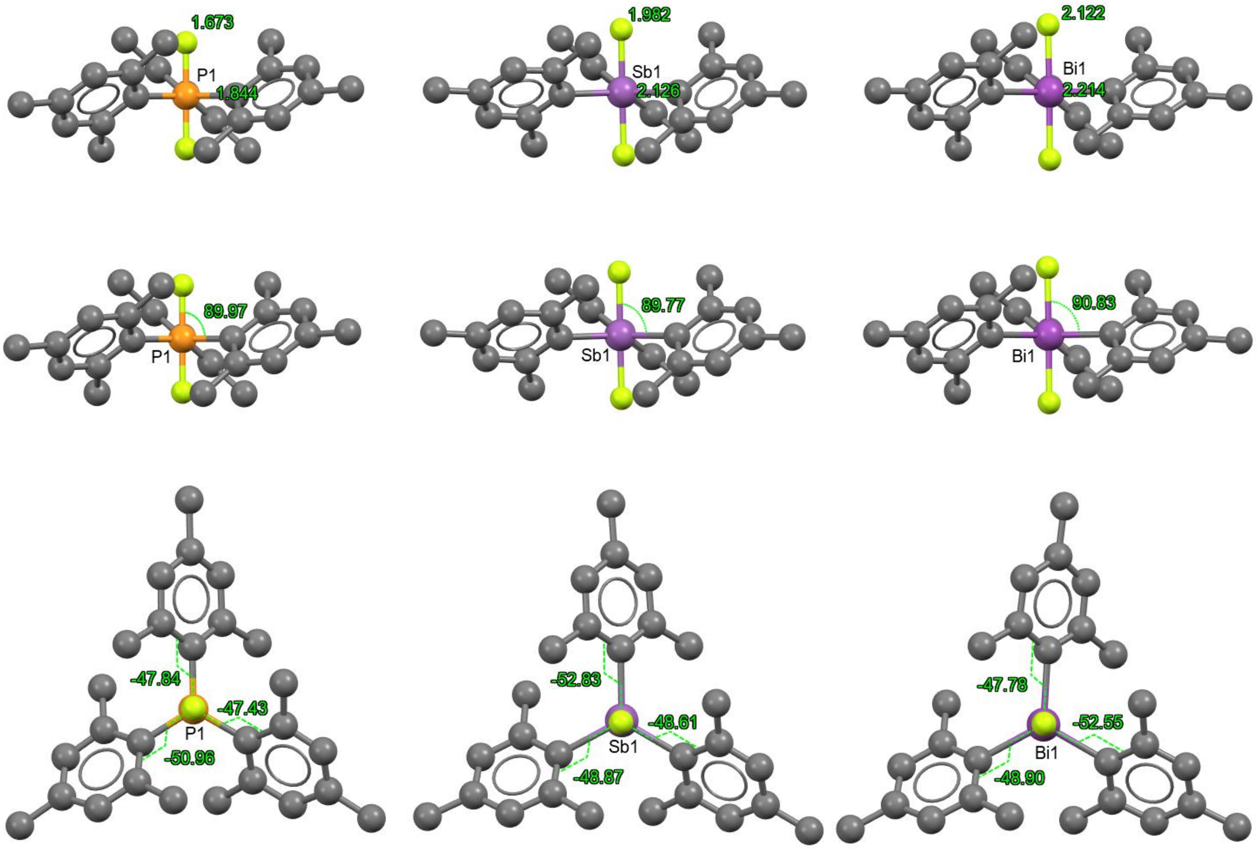

Figure S68. Comparison of geometrical features of isostructural pnictogen compounds (left: FAFNUV, middle: FIQBEP) with complex **10** (right).

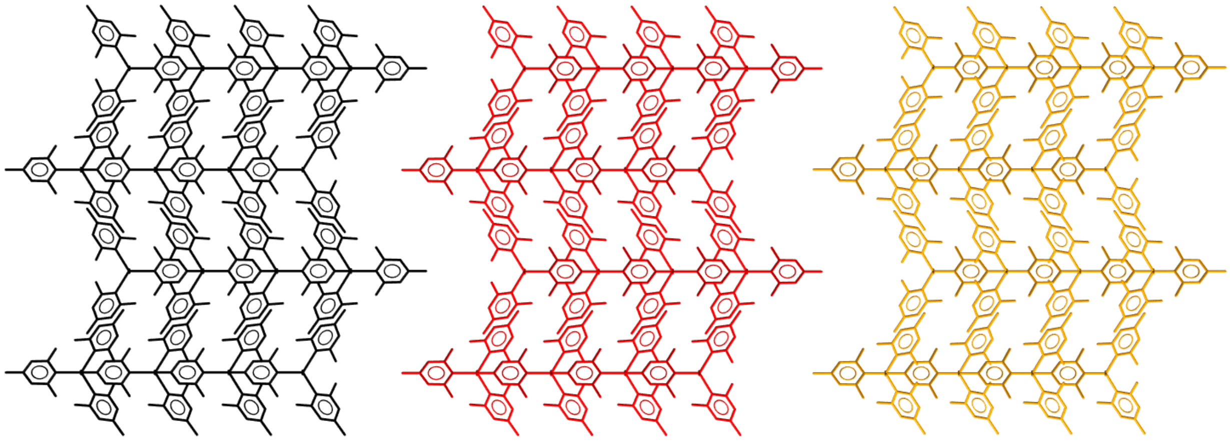

Figure S69. Packing of the three isostructural difluoro-pnictogen-mesityl complexes viewed along crystallographic a axis. Complex **10** (Bi; black), FIWBEP (Sb; red) and FAFNUV (P; yellow)

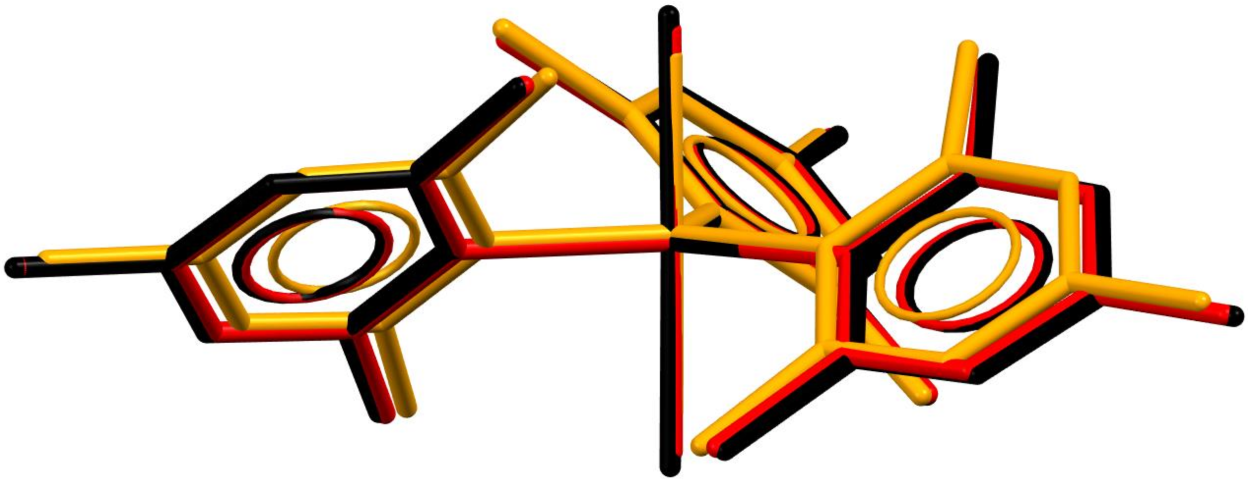

Figure S70. Super positioning of isostructural difluoro-pnictogen-mesityl complexes **10** (Bi; black), FIWBEP (Sb; red) and FAFNUV (P; yellow).

Table S7. Crystal data and structure refinement of complex **10**.

Identification code	13813	
Empirical formula	$C_{27} H_{33} Bi F_2$	
Color	colourless	
Formula weight	604.51 g·mol^{-1}	
Temperature	100(2) K	
Wavelength	0.71073 Å	
Crystal system	Monoclinic	
Space group	*C*2/c, (no. 15)	
Unit cell dimensions	a = 10.4325(8) Å	α= 90°.
	b = 12.7520(9) Å	β= 104.612(11)°.
	c = 18.044(2) Å	γ = 90°.
Volume	2322.8(4) Å^3	
Z	4	
Density (calculated)	1.729 Mg·m^{-3}	
Absorption coefficient	7.617 mm^{-1}	
F(000)	1184 e	
Crystal size	0.18 x 0.14 x 0.07 mm^3	
θ range for data collection	2.607 to 33.116°.	
Index ranges	-15 ≤ h ≤ 16, -19≤ k ≤ 19, -27 ≤ l ≤ 27	
Reflections collected	24864	
Independent reflections	4428 [R_{int} = 0.0279]	
Reflections with I>2σ(I)	4194	
Completeness to θ = 25.242°	99.9 %	
Absorption correction	Gaussian	
Max. and min. transmission	0.61971 and 0.27610	
Refinement method	Full-matrix least-squares on F^2	
Data / restraints / parameters	4428 / 0 / 143	
Goodness-of-fit on F^2	1.083	
Final R indices [I>2σ(I)]	R_1 = 0.0148	wR^2 = 0.0333
R indices (all data)	R_1 = 0.0171	wR^2 = 0.0338
Extinction coefficient	n/a	
Largest diff. peak and hole	0.551 and -0.769 e·Å^{-3}	

Table S8. Bond lengths [Å] and angles [°] of complex **10**.

Bi(1)-F(1)	2.1222(10)	Bi(1)-F(1)#1	2.1223(10)
Bi(1)-C(1)#1	2.2145(14)	Bi(1)-C(1)	2.2144(14)
Bi(1)-C(10)	2.2141(18)	C(1)-C(2)	1.403(2)
C(1)-C(6)	1.406(2)	C(2)-C(3)	1.399(2)
C(2)-C(7)	1.514(2)	C(3)-H(3)	0.9500
C(3)-C(4)	1.397(2)	C(4)-C(5)	1.393(2)
C(4)-C(8)	1.504(2)	C(5)-H(5)	0.9500
C(5)-C(6)	1.397(2)	C(6)-C(9)	1.509(2)
C(7)-H(7A)	0.9800	C(7)-H(7B)	0.9800
C(7)-H(7C)	0.9800	C(8)-H(8A)	0.9800
C(8)-H(8B)	0.9800	C(8)-H(8C)	0.9800
C(9)-H(9A)	0.9800	C(9)-H(9B)	0.9800
C(9)-H(9C)	0.9800	C(10)-C(11)#1	1.4058(17)
C(10)-C(11)	1.4057(17)	C(11)-C(12)	1.3981(19)
C(11)-C(14)	1.507(2)	C(12)-H(12)	0.9500
C(12)-C(13)	1.3914(19)	C(13)-C(15)	1.509(3)
C(14)-H(14A)	0.9800	C(14)-H(14B)	0.9800
C(14)-H(14C)	0.9800	C(15)-H(15A)	0.9800
C(15)-H(15B)	0.9800	C(15)-H(15C)	0.9800
F(1)-Bi(1)-F(1)#1	179.00(5)	F(1)-Bi(1)-C(1)#1	90.83(5)
F(1)#1-Bi(1)-C(1)#1	89.69(5)	F(1)-Bi(1)-C(1)	89.69(5)
F(1)#1-Bi(1)-C(1)	90.83(5)	F(1)-Bi(1)-C(10)	89.50(2)
F(1)#1-Bi(1)-C(10)	89.50(2)	C(1)-Bi(1)-C(1)#1	116.58(7)
C(10)-Bi(1)-C(1)	121.71(4)	C(10)-Bi(1)-C(1)#1	121.71(4)
C(2)-C(1)-Bi(1)	119.06(10)	C(2)-C(1)-C(6)	122.18(13)
C(6)-C(1)-Bi(1)	118.64(10)	C(1)-C(2)-C(7)	123.78(13)
C(3)-C(2)-C(1)	117.79(13)	C(3)-C(2)-C(7)	118.43(13)
C(2)-C(3)-H(3)	119.0	C(4)-C(3)-C(2)	122.01(14)
C(4)-C(3)-H(3)	119.0	C(3)-C(4)-C(8)	120.90(14)
C(5)-C(4)-C(3)	118.09(14)	C(5)-C(4)-C(8)	121.01(14)
C(4)-C(5)-H(5)	118.7	C(4)-C(5)-C(6)	122.63(14)
C(6)-C(5)-H(5)	118.7	C(1)-C(6)-C(9)	124.22(13)
C(5)-C(6)-C(1)	117.29(13)	C(5)-C(6)-C(9)	118.49(13)
C(2)-C(7)-H(7A)	109.5	C(2)-C(7)-H(7B)	109.5
C(2)-C(7)-H(7C)	109.5	H(7A)-C(7)-H(7B)	109.5

H(7A)-C(7)-H(7C)	109.5	H(7B)-C(7)-H(7C)	109.5
C(4)-C(8)-H(8A)	109.5	C(4)-C(8)-H(8B)	109.5
C(4)-C(8)-H(8C)	109.5	H(8A)-C(8)-H(8B)	109.5
H(8A)-C(8)-H(8C)	109.5	H(8B)-C(8)-H(8C)	109.5
C(6)-C(9)-H(9A)	109.5	C(6)-C(9)-H(9B)	109.5
C(6)-C(9)-H(9C)	109.5	H(9A)-C(9)-H(9B)	109.5
H(9A)-C(9)-H(9C)	109.5	H(9B)-C(9)-H(9C)	109.5
C(11)#1-C(10)-Bi(1)	118.83(9)	C(11)-C(10)-Bi(1)	118.83(9)
C(11)-C(10)-C(11)#1	122.34(18)	C(10)-C(11)-C(14)	124.25(13)
C(12)-C(11)-C(10)	117.32(14)	C(12)-C(11)-C(14)	118.43(13)
C(11)-C(12)-H(12)	118.8	C(13)-C(12)-C(11)	122.36(15)
C(13)-C(12)-H(12)	118.8	C(12)-C(13)-C(12)#1	118.30(19)
C(12)-C(13)-C(15)	120.85(9)	C(12)#1-C(13)-C(15)	120.85(9)
C(11)-C(14)-H(14A)	109.5	C(11)-C(14)-H(14B)	109.5
C(11)-C(14)-H(14C)	109.5	H(14A)-C(14)-H(14B)	109.5
H(14A)-C(14)-H(14C)	109.5	H(14B)-C(14)-H(14C)	109.5
C(13)-C(15)-H(15A)	109.5	C(13)-C(15)-H(15B)	109.5
C(13)-C(15)-H(15C)	109.5	H(15A)-C(15)-H(15B)	109.5
H(15A)-C(15)-H(15C)	109.5	H(15B)-C(15)-H(15C)	109.5

_

Symmetry transformations used to generate equivalent atoms:

#1 -x+1,y,-z+1/2

Single crystal structure analysis of complex 11 (13765)

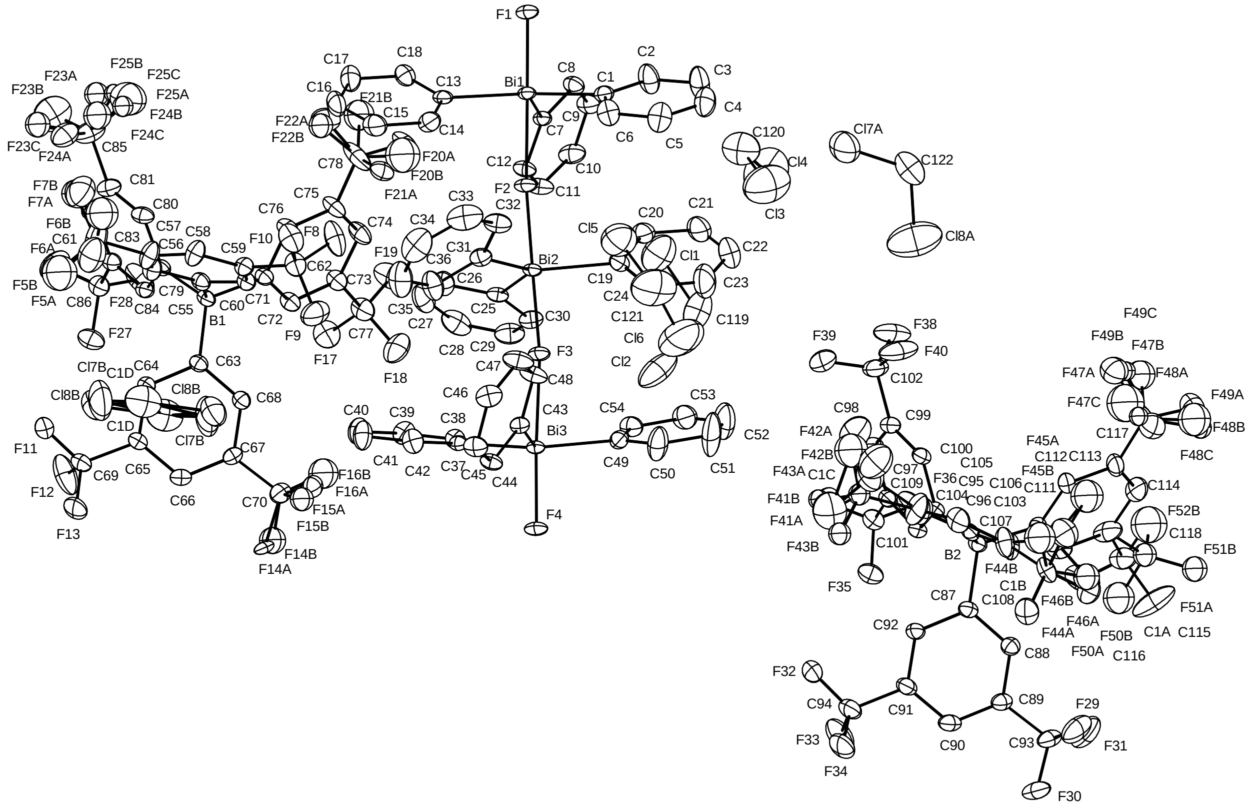

Figure S71. The molecular structure of complex **11**. H atoms have been removed for clarity. Ellipsoids are drawn at the 50% probability level.

X-ray Crystal Structure Analysis of complex 11:

$C_{122}H_{77}B_2Bi_3Cl_8F_{52}$, M_r = 3462.99 g mol^{-1}, colorless prism, crystal size 0.24 x 0.22 x 0.04 mm^3, triclinic, space group P-1 [2], a = 12.5357(6) Å, b = 17.8039(9) Å, c = 30.0743(15) Å, α = 80.321(2)°, β= 82.180(2)°, γ = 88.298(2)°, V = 6554.9(6) Å^3, T = 200(2) K, Z = 2, D_{calc} = 1.755 g·cm^3, λ = 0.71073 Å, $\mu(Mo\text{-}K_\alpha)$ = 4.301 mm^{-1}, Gaussian absorption correction ($T_{\min}$ = 0.40587, $T_{\max}$ = 0.77245), Bruker-AXS Kappa Mach3 with APEX-II detector and IµS microfocus Mo-anode X-ray source, 1.249 < θ < 32.577°, 240967 measured reflections, 47652 independent reflections, 36528 reflections with $I > 2\sigma(I)$, R_{int} = 0.0415. The structure was solved by *SHELXT* and refined by full-matrix least-squares (*SHELXL*) against F^2 to R_1 = 0.0416 [$I > 2\sigma(I)$], wR_2 = 0.1063 [all data], 1844 parameters and 244 restraints.

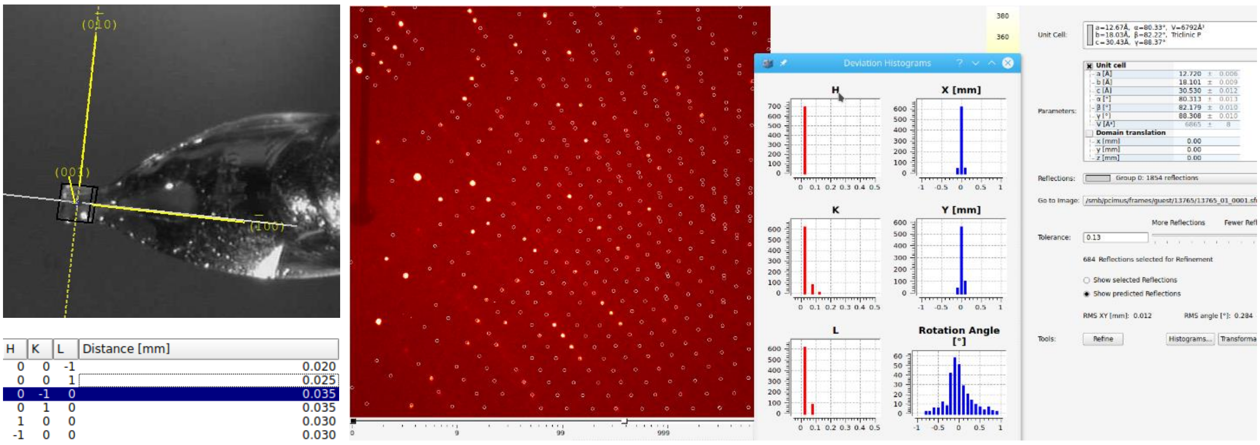

Figure S72. Crystal faces and unit cell determination/refinement of complex **11**.

INTENSITY STATISTICS FOR DATASET

Resolution	#Data	#Theory	%Complete	Redundancy	Mean I	Mean I/s	Rmerge	Rsigma
Inf - 2.48	897	897	100.0	9.48	40.31	45.92	0.0280	0.0189
2.48 - 1.66	2111	2111	100.0	9.63	27.91	40.87	0.0243	0.0191
1.66 - 1.32	2986	2986	100.0	9.52	17.47	36.03	0.0265	0.0204
1.32 - 1.15	3023	3023	100.0	9.19	11.89	31.13	0.0341	0.0219
1.15 - 1.04	3159	3159	100.0	7.54	10.09	25.21	0.0413	0.0274
1.04 - 0.97	2865	2865	100.0	5.63	8.70	19.86	0.0487	0.0343
0.97 - 0.91	3173	3173	100.0	4.71	6.54	15.38	0.0592	0.0431
0.91 - 0.87	2657	2657	100.0	4.13	6.14	13.81	0.0645	0.0490
0.87 - 0.83	3128	3128	100.0	3.93	5.06	11.83	0.0724	0.0570
0.83 - 0.80	2829	2829	100.0	3.74	4.38	10.60	0.0826	0.0656
0.80 - 0.77	3261	3261	100.0	3.57	3.89	9.48	0.0892	0.0755
0.77 - 0.74	3760	3761	100.0	3.44	3.44	8.32	0.1011	0.0874
0.74 - 0.72	2928	2933	99.8	3.27	3.48	7.91	0.1029	0.0938
0.72 - 0.70	3231	3241	99.7	3.17	2.89	6.82	0.1178	0.1116
0.70 - 0.69	1733	1744	99.4	3.03	2.52	6.00	0.1306	0.1302
0.69 - 0.67	3857	3882	99.4	2.96	2.42	5.68	0.1392	0.1386
0.67 - 0.66	2072	2088	99.2	2.93	2.08	5.03	0.1580	0.1633
0.66 - 0.64	4563	4656	98.0	2.75	2.00	4.68	0.1673	0.1770
0.64 - 0.63	2449	2488	98.4	2.67	1.80	4.16	0.1854	0.2025
0.63 - 0.62	2628	2731	96.2	2.59	1.65	3.88	0.1997	0.2250
0.62 - 0.61	2287	3062	74.7	1.71	1.53	3.26	0.2095	0.2880
0.71 - 0.61	21246	22313	95.2	2.69	2.07	4.85	0.1571	0.1723
Inf - 0.61	59597	60675	98.2	4.50	6.54	13.72	0.0439	0.0512

A resolution cut-off (SHEL 99 0.6) was applied to exclude poorly determined intensities at high diffraction angles. Nineteen reflections with high I/σI have been omitted from the data set before final refinement cycles. The structure consist of several disordered entities (solute molecules and parts of anion) wich are described in different parts. DSR Tool plug-in in Olex2 program suite was partially used to restrain disordered parts. Eight of sixteen terminal CF_3 groups show rotational or even positional disorders over two or more positions with different fixed occupancies. Atoms of minor parts have been described by isotropic displacement parameters. ISOR instruction was used to treat thermal displacement parameters of several F

atoms belonging to disordered CF_3 groups. One molecule of DCM is disordered on a crystallographic special position (inversion centre) with occupancy of 50:50%. Only one solute molecule could not be refined in a proper way. To improve signal-to-noise ratio quality the SQUEEZE routine (PLATON; BYPASS in Olex2) was applied to the data set. This results in a residual electron density of 2.37 and -1.76 eÅ^{-3} as well as a void volume of 84.04 Å^3 (1.3% of unit cell volume) using 1.2 Å probe radius and a 0.7 Å grid size (CCDC Mercury program) after final refinement. Complete .cif-data of the compound are available under the CCDC number **CCDC-2154891**.

Supramolecular features and coordination

The solid state of complex **11** involves intermolecular interactions leading to the formation of a cationic 1D coordination polymer. Complex **11** shows geometrical similarities to complexes **3** and **4**, but with less pronounced behavior.

In complex **11** the intermolecular distances Bi1–F4 are 2.796 Å and Bi3–F1 2.855 Å. The intermolecular Bi···F distances are slightly elongated compared to those found in complexes **3** and **4**.

Another indication of the coordinative interaction can be found in the C–Bi–C angles. The angles between C1–Bi1–C13 (134.87°) and C37–Bi3–C49 (133.88°) are significantly widened to allow dense packing between the terminal F atoms and the Bi(V) atoms. Potential π-π interactions (sandwich configuration) could be found with centroid distances of 4.275 Å and 4.375 Å for the aryl ligands.

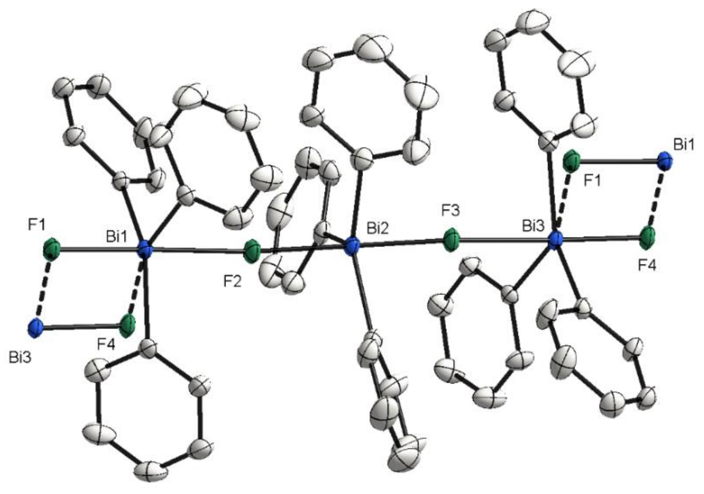

Figure S73. One-dimensional coordination network of complex **11** (dashed lines) consisting of intermolecular F–Bi···F–Bi interactions. Ellipsoids are drawn at the 50% probability level.

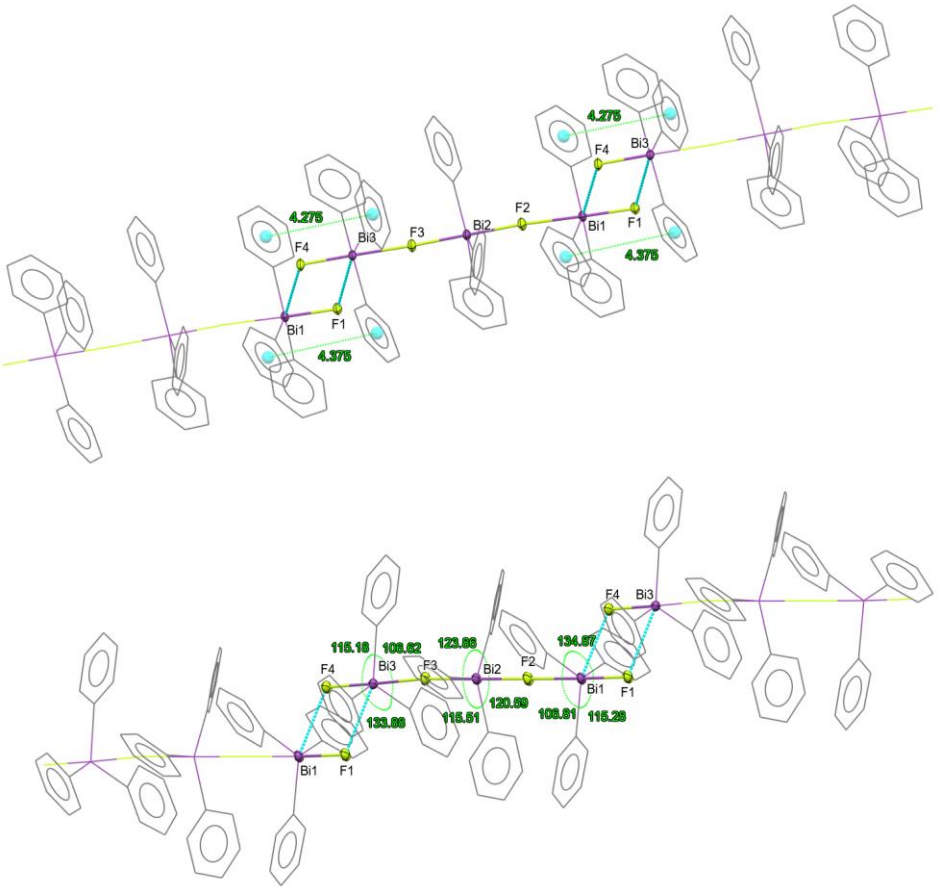

Figure S74. Potential π stacking of phenyl ligands (top) and C–Bi–C angles (bottom) indicating intermolecular interactions in the solid state of complex **11**. Ellipsoids are drawn at the 50% probability level.

This leads to a distorted octahedral coordination sphere at the Bi(V) central atoms in complex **3**.

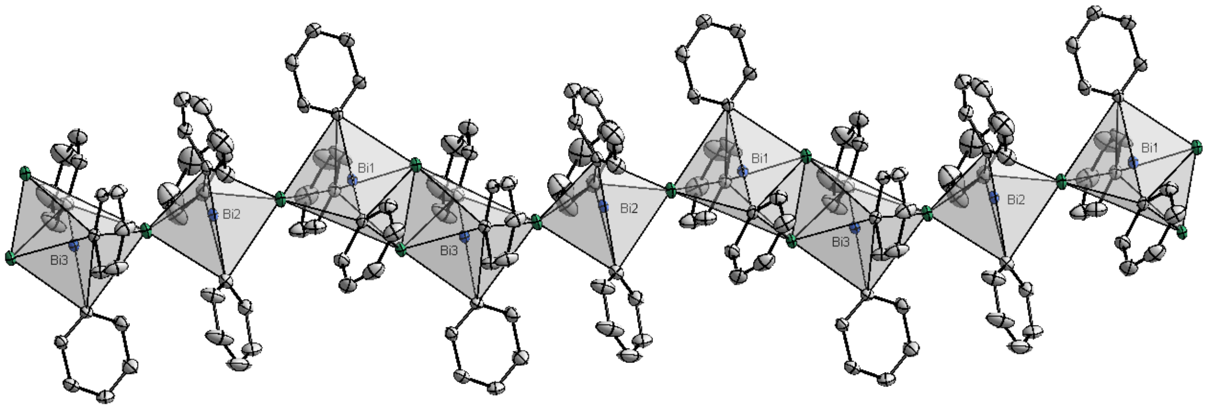

Figure S75. Polyhedral representation of altering distorted octahedral (terminal Bi1 and Bi3) and trigonal bipyramidal (central Bi2) coordination spheres of the Bi(V) central atoms in complex **11**. Ellipsoids are drawn at the 50% probability level.

It is worth to mention that in this structure the central Bi2 atom is surrounded by Ar ligands which are orientated in a propeller like geometry. This is in good agreement with the geometry of monomeric and neutral BiF_2Ph_3 (XULFUF01) as shown by the overlay with a low RMS of 0.082. Overlaying the neutral form with both terminal units (Bi1 and Bi3) lead to an increased RMS of 0.17 and 0.262 indicating geometrical differences.

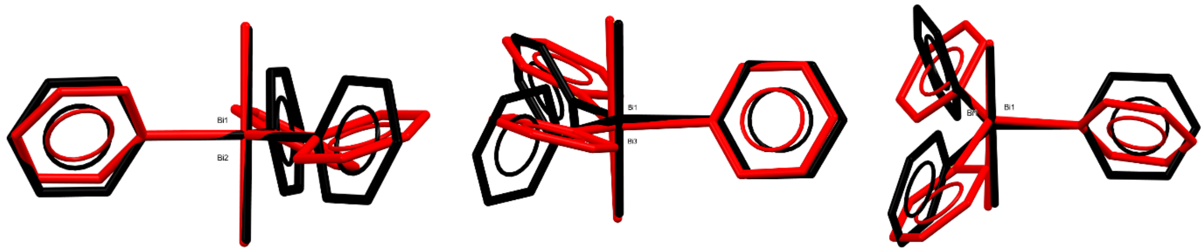

Figure S76. Super positioning of XULFUF01 (black) with subunits of complex 11 (red). Central Bi2 subunit (left) with propeller like geometry, terminal Bi1 subunit (middle) and terminal Bi3 subunit (right).

This observation made us speculate that both positive charges are located on the terminal atoms Bi1 and Bi3. Additionally the Bi–F distances support thus findings. Both terminal F atoms having a shorter bond length of 2.078 Å and 2.068 Å to the corresponding terminal Bi atmos. These particularly short distances can be compared with those in complex **13**. The central atom Bi2 has two Bi2–F bond lengths (2.173 Å and 2.187 Å) which fit mostly to those observed in the neutral molecule of XULFUF01.

It should be noted that the cationic units of **11** are building infinite chains along the crystallographic a axis. This contradicts Coulomb’s law because of close distances between individual, cationic units. The anionic BAr^F units orient themselves around those chains for charge compensation.

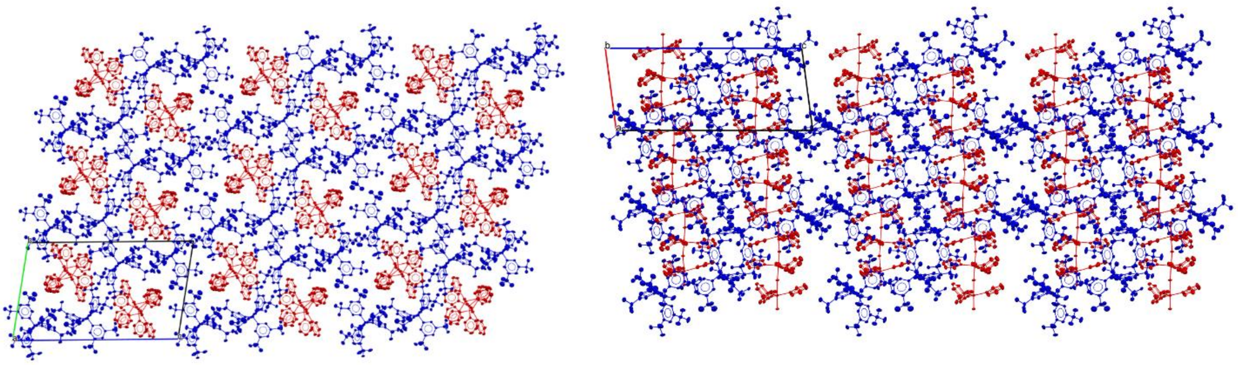

Figure S77. Packing of complex **11** is formed by columns of one dimensional, cationic coordination polymer (red) surrounded by weakly coordinating BAr^F (blue) anions. Unit cell is shown and the viewing direction is along crystallographic a axis (left) as well as b axis (right).

Table S9. Crystal data and structure refinement of complex **11**.

Identification code	13765	
Empirical formula	$C_{122} H_{77} B_2 Bi_3 Cl_8 F_{52}$	
Color	colourless	
Formula weight	3462.99 g·mol^{-1}	
Temperature	200(2) K	
Wavelength	0.71073 Å	
Crystal system	Triclinic	
Space group	*P*-1, (no. 2)	
Unit cell dimensions	a = 12.5357(6) Å	α= 80.321(2)°.
	b = 17.8039(9) Å	β= 82.180(2)°.
	c = 30.0743(15) Å	γ = 88.298(2)°.
Volume	6554.9(6) Å^3	
Z	2	
Density (calculated)	1.755 Mg·m^{-3}	
Absorption coefficient	4.301 mm^{-1}	
F(000)	3344 e	
Crystal size	0.24 x 0.22 x 0.04 mm^3	
θ range for data collection	1.249 to 32.577°.	
Index ranges	-18 ≤ h ≤ 18, -26≤ k ≤ 26, -45 ≤ l ≤ 45	
Reflections collected	240967	
Independent reflections	47652 [R_{int} = 0.0415]	
Reflections with I>2σ(I)	36528	
Completeness to θ = 25.242°	99.9 %	
Absorption correction	Gaussian	
Max. and min. transmission	0.77245 and 0.40587	
Refinement method	Full-matrix least-squares on F^2	
Data / restraints / parameters	47652 / 244 / 1844	
Goodness-of-fit on F^2	1.018	
Final R indices [I>2σ(I)]	R_1 = 0.0416	wR^2 = 0.0971
R indices (all data)	R_1 = 0.0620	wR^2 = 0.1063
Extinction coefficient	n/a	
Largest diff. peak and hole	2.366 and -1.761 e·Å^{-3}	

Table S10. Bond lengths [Å] and angles [°] of complex **11**.

Bi(1)-F(1)	2.078(2)	Bi(1)-F(2)	2.376(2)
Bi(1)-C(1)	2.172(4)	Bi(1)-C(7)	2.186(3)
Bi(1)-C(13)	2.170(4)	Bi(2)-F(2)	2.187(2)
Bi(2)-F(3)	2.173(2)	Bi(2)-C(19)	2.169(4)
Bi(2)-C(25)	2.161(4)	Bi(2)-C(31)	2.177(4)
Bi(3)-F(3)	2.3729(19)	Bi(3)-F(4)	2.068(2)
Bi(3)-C(37)	2.178(4)	Bi(3)-C(43)	2.190(3)
Bi(3)-C(49)	2.173(4)	C(1)-C(2)	1.390(6)
C(1)-C(6)	1.379(5)	C(2)-H(2)	0.9500
C(2)-C(3)	1.383(7)	C(3)-H(3)	0.9500
C(3)-C(4)	1.376(7)	C(4)-H(4)	0.9500
C(4)-C(5)	1.384(7)	C(5)-H(5)	0.9500
C(5)-C(6)	1.393(6)	C(6)-H(6)	0.9500
C(7)-C(8)	1.393(5)	C(7)-C(12)	1.392(5)
C(8)-H(8)	0.9500	C(8)-C(9)	1.393(5)
C(9)-H(9)	0.9500	C(9)-C(10)	1.377(6)
C(10)-H(10)	0.9500	C(10)-C(11)	1.390(6)
C(11)-H(11)	0.9500	C(11)-C(12)	1.393(5)
C(12)-H(12)	0.9500	C(13)-C(14)	1.387(5)
C(13)-C(18)	1.373(5)	C(14)-H(14)	0.9500
C(14)-C(15)	1.391(6)	C(15)-H(15)	0.9500
C(15)-C(16)	1.381(7)	C(16)-H(16)	0.9500
C(16)-C(17)	1.386(7)	C(17)-H(17)	0.9500
C(17)-C(18)	1.391(6)	C(18)-H(18)	0.9500
C(19)-C(20)	1.393(6)	C(19)-C(24)	1.370(7)
C(20)-H(20)	0.9500	C(20)-C(21)	1.380(7)
C(21)-H(21)	0.9500	C(21)-C(22)	1.373(9)
C(22)-H(22)	0.9500	C(22)-C(23)	1.379(9)
C(23)-H(23)	0.9500	C(23)-C(24)	1.390(7)
C(24)-H(24)	0.9500	C(25)-C(26)	1.387(6)
C(25)-C(30)	1.384(5)	C(26)-H(26)	0.9500
C(26)-C(27)	1.379(6)	C(27)-H(27)	0.9500
C(27)-C(28)	1.377(8)	C(28)-H(28)	0.9500
C(28)-C(29)	1.372(7)	C(29)-H(29)	0.9500
C(29)-C(30)	1.389(6)	C(30)-H(30)	0.9500
C(31)-C(32)	1.381(5)	C(31)-C(36)	1.381(6)

C(32)-H(32)	0.9500	C(32)-C(33)	1.379(6)
C(33)-H(33)	0.9500	C(33)-C(34)	1.372(9)
C(34)-H(34)	0.9500	C(34)-C(35)	1.388(8)
C(35)-H(35)	0.9500	C(35)-C(36)	1.380(6)
C(36)-H(36)	0.9500	C(37)-C(38)	1.390(5)
C(37)-C(42)	1.390(5)	C(38)-H(38)	0.9500
C(38)-C(39)	1.389(5)	C(39)-H(39)	0.9500
C(39)-C(40)	1.387(6)	C(40)-H(40)	0.9500
C(40)-C(41)	1.387(6)	C(41)-H(41)	0.9500
C(41)-C(42)	1.382(6)	C(42)-H(42)	0.9500
C(43)-C(44)	1.383(5)	C(43)-C(48)	1.385(5)
C(44)-H(44)	0.9500	C(44)-C(45)	1.395(5)
C(45)-H(45)	0.9500	C(45)-C(46)	1.379(6)
C(46)-H(46)	0.9500	C(46)-C(47)	1.385(6)
C(47)-H(47)	0.9500	C(47)-C(48)	1.394(6)
C(48)-H(48)	0.9500	C(49)-C(50)	1.366(6)
C(49)-C(54)	1.384(5)	C(50)-H(50)	0.9500
C(50)-C(51)	1.392(7)	C(51)-H(51)	0.9500
C(51)-C(52)	1.379(8)	C(52)-H(52)	0.9500
C(52)-C(53)	1.366(8)	C(53)-H(53)	0.9500
C(53)-C(54)	1.382(6)	C(54)-H(54)	0.9500
Cl(1)-C(119)	1.727(7)	Cl(2)-C(119)	1.780(8)
C(119)-H(11A)	0.9900	C(119)-H(11B)	0.9900
Cl(3)-C(120)	1.703(8)	Cl(4)-C(120)	1.764(8)
C(120)-H(12A)	0.9900	C(120)-H(12B)	0.9900
Cl(5)-C(121)	1.724(8)	Cl(6)-C(121)	1.758(10)
C(121)-H(12C)	0.9900	C(121)-H(12D)	0.9900
Cl(7)-C(122)	1.694(13)	Cl(8)-C(122)	1.760(13)
C(122)-H(12E)	0.9900	C(122)-H(12F)	0.9900
Cl(7B)-C(1A)	1.740(7)	Cl(8B)-C(1A)	1.762(8)
C(1A)-H(1AA)	0.9900	C(1A)-H(1AB)	0.9900
F(5A)-C(61)	1.428(7)	F(5B)-C(61)	1.227(9)
F(6A)-C(61)	1.335(6)	F(6B)-C(61)	1.404(8)
F(7A)-C(61)	1.247(7)	F(7B)-C(61)	1.427(8)
F(8)-C(62)	1.348(5)	F(9)-C(62)	1.334(5)
F(10)-C(62)	1.332(4)	F(11)-C(69)	1.321(5)
F(12)-C(69)	1.344(5)	F(13)-C(69)	1.321(5)
F(14A)-C(70)	1.407(7)	F(14B)-C(70)	1.254(9)
F(15A)-C(70)	1.290(6)	F(15B)-C(70)	1.380(8)

F(16A)-C(70)	1.357(6)	F(16B)-C(70)	1.373(9)
F(17)-C(77)	1.335(5)	F(18)-C(77)	1.343(6)
F(19)-C(77)	1.331(5)	F(20A)-C(78)	1.349(6)
F(20B)-C(78)	1.203(8)	F(21A)-C(78)	1.478(6)
F(21B)-C(78)	1.481(7)	F(22A)-C(78)	1.216(7)
F(22B)-C(78)	1.383(7)	F(23A)-C(85)	1.294(8)
F(23B)-C(85)	1.311(9)	F(23C)-C(85)	1.440(8)
F(24A)-C(85)	1.361(6)	F(24B)-C(85)	1.420(8)
F(24C)-C(85)	1.321(9)	F(25A)-C(85)	1.272(8)
F(25B)-C(85)	1.383(9)	F(25C)-C(85)	1.408(8)
F(26)-C(86)	1.335(5)	F(27)-C(86)	1.335(5)
F(28)-C(86)	1.335(4)	C(55)-C(56)	1.395(5)
C(55)-C(60)	1.400(5)	C(55)-B(1)	1.637(5)
C(56)-H(56)	0.9500	C(56)-C(57)	1.391(5)
C(57)-C(58)	1.376(6)	C(57)-C(61)	1.499(6)
C(58)-H(58)	0.9500	C(58)-C(59)	1.385(5)
C(59)-C(60)	1.389(5)	C(59)-C(62)	1.489(5)
C(60)-H(60)	0.9500	C(63)-C(64)	1.403(5)
C(63)-C(68)	1.397(5)	C(63)-B(1)	1.626(5)
C(64)-H(64)	0.9500	C(64)-C(65)	1.390(5)
C(65)-C(66)	1.387(5)	C(65)-C(69)	1.498(5)
C(66)-H(66)	0.9500	C(66)-C(67)	1.386(5)
C(67)-C(68)	1.393(5)	C(67)-C(70)	1.496(6)
C(68)-H(68)	0.9500	C(71)-C(72)	1.400(5)
C(71)-C(76)	1.399(5)	C(71)-B(1)	1.643(5)
C(72)-H(72)	0.9500	C(72)-C(73)	1.392(5)
C(73)-C(74)	1.384(5)	C(73)-C(77)	1.501(5)
C(74)-H(74)	0.9500	C(74)-C(75)	1.390(5)
C(75)-C(76)	1.383(5)	C(75)-C(78)	1.485(6)
C(76)-H(76)	0.9500	C(79)-C(80)	1.403(5)
C(79)-C(84)	1.404(5)	C(79)-B(1)	1.641(6)
C(80)-H(80)	0.9500	C(80)-C(81)	1.387(6)
C(81)-C(82)	1.384(6)	C(81)-C(85)	1.496(6)
C(82)-H(82)	0.9500	C(82)-C(83)	1.385(5)
C(83)-C(84)	1.391(5)	C(83)-C(86)	1.502(5)
C(84)-H(84)	0.9500	F(29)-C(93)	1.328(5)
F(30)-C(93)	1.318(5)	F(31)-C(93)	1.343(5)
F(32)-C(94)	1.325(6)	F(33)-C(94)	1.337(6)
F(34)-C(94)	1.339(5)	F(35)-C(101)	1.340(5)

F(36)-C(101)	1.348(5)	F(37)-C(101)	1.337(5)
F(38)-C(102)	1.351(6)	F(39)-C(102)	1.315(6)
F(40)-C(102)	1.326(5)	F(41A)-C(1B)	1.310(9)
F(41B)-C(109)	1.350(13)	F(42A)-C(1B)	1.307(10)
F(42B)-C(109)	1.284(13)	F(43A)-C(1B)	1.286(9)
F(43B)-C(109)	1.384(13)	F(44A)-C(110)	1.344(8)
F(44B)-C(1C)	1.332(16)	F(45A)-C(110)	1.358(8)
F(45B)-F(51B)#1	1.697(13)	F(45B)-C(1C)	1.358(17)
F(46A)-C(110)	1.314(9)	F(46B)-C(1C)	1.285(17)
F(47A)-C(117)	1.398(12)	F(47B)-C(117)	1.389(11)
F(47C)-C(117)	1.357(18)	F(48A)-C(117)	1.277(12)
F(48B)-C(117)	1.443(10)	F(48C)-C(117)	1.258(14)
F(49A)-C(117)	1.309(11)	F(49B)-C(117)	1.328(10)
F(49C)-C(117)	1.330(12)	F(50A)-C(118)	1.343(11)
F(50B)-C(1D)	1.331(12)	F(51A)-C(118)	1.324(9)
F(51B)-C(1D)	1.299(11)	F(52A)-C(118)	1.324(8)
F(52B)-C(1D)	1.319(13)	C(118)-C(115)	1.556(9)
C(110)-C(107)	1.567(9)	C(109)-C(105)	1.52(2)
C(87)-C(88)	1.388(5)	C(87)-C(92)	1.409(5)
C(87)-B(2)	1.642(5)	C(88)-H(88)	0.9500
C(88)-C(89)	1.394(5)	C(89)-C(90)	1.383(6)
C(89)-C(93)	1.493(6)	C(90)-H(90)	0.9500
C(90)-C(91)	1.396(6)	C(91)-C(92)	1.382(5)
C(91)-C(94)	1.503(6)	C(92)-H(92)	0.9500
C(95)-C(96)	1.401(5)	C(95)-C(100)	1.404(5)
C(95)-B(2)	1.638(6)	C(96)-H(96)	0.9500
C(96)-C(97)	1.389(5)	C(97)-C(98)	1.385(5)
C(97)-C(101)	1.491(5)	C(98)-H(98)	0.9500
C(98)-C(99)	1.385(5)	C(99)-C(100)	1.389(5)
C(99)-C(102)	1.495(6)	C(100)-H(100)	0.9500
C(103)-C(104)	1.402(6)	C(103)-C(108)	1.404(6)
C(103)-B(2)	1.639(5)	C(104)-H(104)	0.9500
C(104)-C(105)	1.374(6)	C(105)-C(106)	1.372(7)
C(105)-C(1B)	1.555(9)	C(106)-H(106)	0.9500
C(106)-C(107)	1.387(6)	C(107)-C(108)	1.377(5)
C(107)-C(1C)	1.451(14)	C(108)-H(108)	0.9500
C(111)-C(112)	1.389(5)	C(111)-C(116)	1.401(5)
C(111)-B(2)	1.635(5)	C(112)-H(112)	0.9500
C(112)-C(113)	1.391(5)	C(113)-C(114)	1.389(7)

C(113)-C(117)	1.488(6)	C(114)-H(114)	0.9500
C(114)-C(115)	1.387(7)	C(115)-C(116)	1.380(6)
C(115)-C(1D)	1.459(16)	C(116)-H(116)	0.9500

F(1)-Bi(1)-F(2)	178.50(8)	F(1)-Bi(1)-C(1)	97.38(11)
F(1)-Bi(1)-C(7)	91.46(10)	F(1)-Bi(1)-C(13)	97.07(11)
C(1)-Bi(1)-F(2)	82.36(11)	C(1)-Bi(1)-C(7)	106.81(14)
C(7)-Bi(1)-F(2)	90.03(10)	C(13)-Bi(1)-F(2)	82.11(10)
C(13)-Bi(1)-C(1)	134.88(14)	C(13)-Bi(1)-C(7)	115.28(13)
F(3)-Bi(2)-F(2)	179.44(9)	F(3)-Bi(2)-C(31)	86.93(11)
C(19)-Bi(2)-F(2)	89.63(12)	C(19)-Bi(2)-F(3)	90.05(12)
C(19)-Bi(2)-C(31)	123.86(15)	C(25)-Bi(2)-F(2)	89.36(11)
C(25)-Bi(2)-F(3)	91.20(11)	C(25)-Bi(2)-C(19)	115.53(15)
C(25)-Bi(2)-C(31)	120.57(14)	C(31)-Bi(2)-F(2)	92.85(11)
F(4)-Bi(3)-F(3)	177.35(8)	F(4)-Bi(3)-C(37)	99.32(11)
F(4)-Bi(3)-C(43)	91.20(11)	F(4)-Bi(3)-C(49)	98.45(12)
C(37)-Bi(3)-F(3)	83.33(10)	C(37)-Bi(3)-C(43)	106.62(13)
C(43)-Bi(3)-F(3)	88.02(10)	C(49)-Bi(3)-F(3)	79.60(11)
C(49)-Bi(3)-C(37)	133.87(13)	C(49)-Bi(3)-C(43)	115.18(14)
Bi(2)-F(2)-Bi(1)	175.11(12)	Bi(2)-F(3)-Bi(3)	171.72(11)
C(2)-C(1)-Bi(1)	116.7(3)	C(6)-C(1)-Bi(1)	121.1(3)
C(6)-C(1)-C(2)	122.0(4)	C(1)-C(2)-H(2)	120.8
C(3)-C(2)-C(1)	118.3(4)	C(3)-C(2)-H(2)	120.8
C(2)-C(3)-H(3)	119.6	C(4)-C(3)-C(2)	120.8(4)
C(4)-C(3)-H(3)	119.6	C(3)-C(4)-H(4)	119.9
C(3)-C(4)-C(5)	120.1(4)	C(5)-C(4)-H(4)	119.9
C(4)-C(5)-H(5)	119.9	C(4)-C(5)-C(6)	120.3(4)
C(6)-C(5)-H(5)	119.9	C(1)-C(6)-C(5)	118.5(4)
C(1)-C(6)-H(6)	120.8	C(5)-C(6)-H(6)	120.8
C(8)-C(7)-Bi(1)	118.5(3)	C(12)-C(7)-Bi(1)	119.8(2)
C(12)-C(7)-C(8)	121.4(3)	C(7)-C(8)-H(8)	120.7
C(7)-C(8)-C(9)	118.7(3)	C(9)-C(8)-H(8)	120.7
C(8)-C(9)-H(9)	119.7	C(10)-C(9)-C(8)	120.5(4)
C(10)-C(9)-H(9)	119.7	C(9)-C(10)-H(10)	119.8
C(9)-C(10)-C(11)	120.5(4)	C(11)-C(10)-H(10)	119.8
C(10)-C(11)-H(11)	120.0	C(10)-C(11)-C(12)	120.1(4)
C(12)-C(11)-H(11)	120.0	C(7)-C(12)-C(11)	118.8(3)
C(7)-C(12)-H(12)	120.6	C(11)-C(12)-H(12)	120.6
C(14)-C(13)-Bi(1)	119.8(3)	C(18)-C(13)-Bi(1)	117.1(3)

C(18)-C(13)-C(14)	123.1(4)	C(13)-C(14)-H(14)	121.1
C(13)-C(14)-C(15)	117.8(4)	C(15)-C(14)-H(14)	121.1
C(14)-C(15)-H(15)	120.0	C(16)-C(15)-C(14)	119.9(4)
C(16)-C(15)-H(15)	120.0	C(15)-C(16)-H(16)	119.4
C(15)-C(16)-C(17)	121.2(4)	C(17)-C(16)-H(16)	119.4
C(16)-C(17)-H(17)	120.2	C(16)-C(17)-C(18)	119.6(4)
C(18)-C(17)-H(17)	120.2	C(13)-C(18)-C(17)	118.3(4)
C(13)-C(18)-H(18)	120.8	C(17)-C(18)-H(18)	120.8
C(20)-C(19)-Bi(2)	117.4(3)	C(24)-C(19)-Bi(2)	119.8(3)
C(24)-C(19)-C(20)	122.8(4)	C(19)-C(20)-H(20)	121.0
C(21)-C(20)-C(19)	118.1(5)	C(21)-C(20)-H(20)	121.0
C(20)-C(21)-H(21)	119.9	C(22)-C(21)-C(20)	120.3(5)
C(22)-C(21)-H(21)	119.9	C(21)-C(22)-H(22)	119.7
C(21)-C(22)-C(23)	120.6(5)	C(23)-C(22)-H(22)	119.7
C(22)-C(23)-H(23)	119.7	C(22)-C(23)-C(24)	120.6(6)
C(24)-C(23)-H(23)	119.7	C(19)-C(24)-C(23)	117.7(5)
C(19)-C(24)-H(24)	121.2	C(23)-C(24)-H(24)	121.2
C(26)-C(25)-Bi(2)	119.9(3)	C(30)-C(25)-Bi(2)	118.1(3)
C(30)-C(25)-C(26)	121.9(4)	C(25)-C(26)-H(26)	121.0
C(27)-C(26)-C(25)	118.0(4)	C(27)-C(26)-H(26)	121.0
C(26)-C(27)-H(27)	119.5	C(28)-C(27)-C(26)	121.0(5)
C(28)-C(27)-H(27)	119.5	C(27)-C(28)-H(28)	119.9
C(29)-C(28)-C(27)	120.3(4)	C(29)-C(28)-H(28)	119.9
C(28)-C(29)-H(29)	119.8	C(28)-C(29)-C(30)	120.3(4)
C(30)-C(29)-H(29)	119.8	C(25)-C(30)-C(29)	118.4(4)
C(25)-C(30)-H(30)	120.8	C(29)-C(30)-H(30)	120.8
C(32)-C(31)-Bi(2)	120.2(3)	C(36)-C(31)-Bi(2)	117.8(3)
C(36)-C(31)-C(32)	122.0(4)	C(31)-C(32)-H(32)	120.7
C(33)-C(32)-C(31)	118.6(4)	C(33)-C(32)-H(32)	120.7
C(32)-C(33)-H(33)	119.8	C(34)-C(33)-C(32)	120.3(5)
C(34)-C(33)-H(33)	119.8	C(33)-C(34)-H(34)	119.7
C(33)-C(34)-C(35)	120.5(4)	C(35)-C(34)-H(34)	119.7
C(34)-C(35)-H(35)	120.0	C(36)-C(35)-C(34)	120.0(5)
C(36)-C(35)-H(35)	120.0	C(31)-C(36)-H(36)	120.7
C(35)-C(36)-C(31)	118.6(5)	C(35)-C(36)-H(36)	120.7
C(38)-C(37)-Bi(3)	122.0(3)	C(42)-C(37)-Bi(3)	115.3(3)
C(42)-C(37)-C(38)	122.6(3)	C(37)-C(38)-H(38)	121.1
C(39)-C(38)-C(37)	117.8(3)	C(39)-C(38)-H(38)	121.1
C(38)-C(39)-H(39)	119.7	C(40)-C(39)-C(38)	120.7(4)

C(40)-C(39)-H(39)	119.7	C(39)-C(40)-H(40)	120.0
C(39)-C(40)-C(41)	120.0(4)	C(41)-C(40)-H(40)	120.0
C(40)-C(41)-H(41)	119.6	C(42)-C(41)-C(40)	120.8(4)
C(42)-C(41)-H(41)	119.6	C(37)-C(42)-H(42)	121.0
C(41)-C(42)-C(37)	118.0(4)	C(41)-C(42)-H(42)	121.0
C(44)-C(43)-Bi(3)	117.4(2)	C(44)-C(43)-C(48)	121.9(3)
C(48)-C(43)-Bi(3)	120.5(3)	C(43)-C(44)-H(44)	120.7
C(43)-C(44)-C(45)	118.6(3)	C(45)-C(44)-H(44)	120.7
C(44)-C(45)-H(45)	119.8	C(46)-C(45)-C(44)	120.4(3)
C(46)-C(45)-H(45)	119.8	C(45)-C(46)-H(46)	119.9
C(45)-C(46)-C(47)	120.1(4)	C(47)-C(46)-H(46)	119.9
C(46)-C(47)-H(47)	119.8	C(46)-C(47)-C(48)	120.5(4)
C(48)-C(47)-H(47)	119.8	C(43)-C(48)-C(47)	118.4(4)
C(43)-C(48)-H(48)	120.8	C(47)-C(48)-H(48)	120.8
C(50)-C(49)-Bi(3)	117.1(3)	C(50)-C(49)-C(54)	122.4(4)
C(54)-C(49)-Bi(3)	120.3(3)	C(49)-C(50)-H(50)	121.0
C(49)-C(50)-C(51)	117.9(5)	C(51)-C(50)-H(50)	121.0
C(50)-C(51)-H(51)	119.8	C(52)-C(51)-C(50)	120.4(5)
C(52)-C(51)-H(51)	119.8	C(51)-C(52)-H(52)	119.7
C(53)-C(52)-C(51)	120.7(5)	C(53)-C(52)-H(52)	119.7
C(52)-C(53)-H(53)	120.0	C(52)-C(53)-C(54)	120.0(5)
C(54)-C(53)-H(53)	120.0	C(49)-C(54)-H(54)	120.7
C(53)-C(54)-C(49)	118.6(4)	C(53)-C(54)-H(54)	120.7
Cl(1)-C(119)-Cl(2)	110.4(4)	Cl(1)-C(119)-H(11A)	109.6
Cl(1)-C(119)-H(11B)	109.6	Cl(2)-C(119)-H(11A)	109.6
Cl(2)-C(119)-H(11B)	109.6	H(11A)-C(119)-H(11B)	108.1
Cl(3)-C(120)-Cl(4)	112.5(5)	Cl(3)-C(120)-H(12A)	109.1
Cl(3)-C(120)-H(12B)	109.1	Cl(4)-C(120)-H(12A)	109.1
Cl(4)-C(120)-H(12B)	109.1	H(12A)-C(120)-H(12B)	107.8
Cl(5)-C(121)-Cl(6)	110.8(5)	Cl(5)-C(121)-H(12C)	109.5
Cl(5)-C(121)-H(12D)	109.5	Cl(6)-C(121)-H(12C)	109.5
Cl(6)-C(121)-H(12D)	109.5	H(12C)-C(121)-H(12D)	108.1
Cl(7)-C(122)-Cl(8)	111.9(6)	Cl(7)-C(122)-H(12E)	109.2
Cl(7)-C(122)-H(12F)	109.2	Cl(8)-C(122)-H(12E)	109.2
Cl(8)-C(122)-H(12F)	109.2	H(12E)-C(122)-H(12F)	107.9
Cl(7B)-C(1A)-Cl(8B)	109.7(5)	Cl(7B)-C(1A)-H(1AA)	109.7
Cl(7B)-C(1A)-H(1AB)	109.7	Cl(8B)-C(1A)-H(1AA)	109.7
Cl(8B)-C(1A)-H(1AB)	109.7	H(1AA)-C(1A)-H(1AB)	108.2
C(56)-C(55)-C(60)	115.2(3)	C(56)-C(55)-B(1)	124.1(3)

C(60)-C(55)-B(1)	120.7(3)	C(55)-C(56)-H(56)	119.0
C(57)-C(56)-C(55)	122.0(4)	C(57)-C(56)-H(56)	119.0
C(56)-C(57)-C(61)	119.2(4)	C(58)-C(57)-C(56)	121.6(4)
C(58)-C(57)-C(61)	119.1(4)	C(57)-C(58)-H(58)	121.1
C(57)-C(58)-C(59)	117.8(4)	C(59)-C(58)-H(58)	121.1
C(58)-C(59)-C(60)	120.3(4)	C(58)-C(59)-C(62)	120.8(3)
C(60)-C(59)-C(62)	118.8(3)	C(55)-C(60)-H(60)	118.5
C(59)-C(60)-C(55)	123.0(3)	C(59)-C(60)-H(60)	118.5
F(5A)-C(61)-C(57)	109.2(4)	F(5B)-C(61)-F(6B)	108.2(8)
F(5B)-C(61)-F(7B)	107.9(8)	F(5B)-C(61)-C(57)	120.3(8)
F(6A)-C(61)-F(5A)	98.5(5)	F(6A)-C(61)-C(57)	112.1(4)
F(6B)-C(61)-F(7B)	96.3(7)	F(6B)-C(61)-C(57)	112.2(5)
F(7A)-C(61)-F(5A)	105.2(5)	F(7A)-C(61)-F(6A)	113.2(6)
F(7A)-C(61)-C(57)	116.6(5)	F(7B)-C(61)-C(57)	109.2(5)
F(8)-C(62)-C(59)	111.6(3)	F(9)-C(62)-F(8)	106.1(4)
F(9)-C(62)-C(59)	113.0(3)	F(10)-C(62)-F(8)	105.2(3)
F(10)-C(62)-F(9)	106.5(4)	F(10)-C(62)-C(59)	113.9(3)
C(64)-C(63)-B(1)	119.7(3)	C(68)-C(63)-C(64)	116.1(3)
C(68)-C(63)-B(1)	124.1(3)	C(63)-C(64)-H(64)	119.1
C(65)-C(64)-C(63)	121.9(3)	C(65)-C(64)-H(64)	119.1
C(64)-C(65)-C(69)	119.6(3)	C(66)-C(65)-C(64)	121.3(3)
C(66)-C(65)-C(69)	119.1(3)	C(65)-C(66)-H(66)	121.2
C(67)-C(66)-C(65)	117.6(3)	C(67)-C(66)-H(66)	121.2
C(66)-C(67)-C(68)	121.2(3)	C(66)-C(67)-C(70)	119.8(3)
C(68)-C(67)-C(70)	119.0(3)	C(63)-C(68)-H(68)	119.0
C(67)-C(68)-C(63)	122.0(3)	C(67)-C(68)-H(68)	119.0
F(11)-C(69)-F(12)	104.6(4)	F(11)-C(69)-C(65)	113.8(3)
F(12)-C(69)-C(65)	111.8(3)	F(13)-C(69)-F(11)	108.1(3)
F(13)-C(69)-F(12)	105.2(4)	F(13)-C(69)-C(65)	112.7(3)
F(14A)-C(70)-C(67)	111.0(4)	F(14B)-C(70)-F(15B)	105.6(5)
F(14B)-C(70)-F(16B)	105.9(6)	F(14B)-C(70)-C(67)	116.4(5)
F(15A)-C(70)-F(14A)	108.7(5)	F(15A)-C(70)-F(16A)	107.2(5)
F(15A)-C(70)-C(67)	114.5(4)	F(15B)-C(70)-C(67)	111.9(4)
F(16A)-C(70)-F(14A)	103.8(5)	F(16A)-C(70)-C(67)	111.1(4)
F(16B)-C(70)-F(15B)	103.1(5)	F(16B)-C(70)-C(67)	112.9(5)
C(72)-C(71)-B(1)	122.7(3)	C(76)-C(71)-C(72)	115.7(3)
C(76)-C(71)-B(1)	121.2(3)	C(71)-C(72)-H(72)	119.1
C(73)-C(72)-C(71)	121.9(3)	C(73)-C(72)-H(72)	119.1
C(72)-C(73)-C(77)	119.3(3)	C(74)-C(73)-C(72)	121.3(3)

C(74)-C(73)-C(77)	119.4(3)	C(73)-C(74)-H(74)	121.2
C(73)-C(74)-C(75)	117.6(3)	C(75)-C(74)-H(74)	121.2
C(74)-C(75)-C(78)	120.2(3)	C(76)-C(75)-C(74)	121.0(3)
C(76)-C(75)-C(78)	118.8(3)	C(71)-C(76)-H(76)	118.8
C(75)-C(76)-C(71)	122.5(3)	C(75)-C(76)-H(76)	118.8
F(17)-C(77)-F(18)	105.4(4)	F(17)-C(77)-C(73)	112.5(3)
F(18)-C(77)-C(73)	111.8(4)	F(19)-C(77)-F(17)	106.5(4)
F(19)-C(77)-F(18)	107.1(4)	F(19)-C(77)-C(73)	113.0(4)
F(20A)-C(78)-F(21A)	94.3(4)	F(20A)-C(78)-C(75)	112.6(4)
F(20B)-C(78)-F(21B)	102.2(6)	F(20B)-C(78)-F(22B)	113.9(6)
F(20B)-C(78)-C(75)	121.0(6)	F(21A)-C(78)-C(75)	106.8(4)
F(21B)-C(78)-C(75)	108.4(4)	F(22A)-C(78)-F(20A)	116.5(6)
F(22A)-C(78)-F(21A)	106.6(5)	F(22A)-C(78)-C(75)	116.8(5)
F(22B)-C(78)-F(21B)	91.3(5)	F(22B)-C(78)-C(75)	114.5(4)
C(80)-C(79)-C(84)	115.6(3)	C(80)-C(79)-B(1)	122.3(3)
C(84)-C(79)-B(1)	121.8(3)	C(79)-C(80)-H(80)	119.0
C(81)-C(80)-C(79)	122.0(3)	C(81)-C(80)-H(80)	119.0
C(80)-C(81)-C(85)	119.3(4)	C(82)-C(81)-C(80)	121.7(4)
C(82)-C(81)-C(85)	119.1(4)	C(81)-C(82)-H(82)	121.3
C(81)-C(82)-C(83)	117.4(4)	C(83)-C(82)-H(82)	121.3
C(82)-C(83)-C(84)	121.3(3)	C(82)-C(83)-C(86)	119.1(3)
C(84)-C(83)-C(86)	119.4(3)	C(79)-C(84)-H(84)	118.9
C(83)-C(84)-C(79)	122.1(3)	C(83)-C(84)-H(84)	118.9
F(23A)-C(85)-F(24A)	103.9(6)	F(23A)-C(85)-C(81)	113.6(6)
F(23B)-C(85)-F(24B)	112.9(8)	F(23B)-C(85)-F(25B)	98.0(8)
F(23B)-C(85)-C(81)	118.8(6)	F(23C)-C(85)-C(81)	110.1(5)
F(24A)-C(85)-C(81)	108.2(4)	F(24B)-C(85)-C(81)	113.1(5)
F(24C)-C(85)-F(23C)	112.0(8)	F(24C)-C(85)-F(25C)	107.2(8)
F(24C)-C(85)-C(81)	117.7(7)	F(25A)-C(85)-F(23A)	112.4(7)
F(25A)-C(85)-F(24A)	105.1(6)	F(25A)-C(85)-C(81)	112.7(6)
F(25B)-C(85)-F(24B)	97.2(7)	F(25B)-C(85)-C(81)	113.8(6)
F(25C)-C(85)-F(23C)	97.9(7)	F(25C)-C(85)-C(81)	110.1(5)
F(26)-C(86)-F(27)	106.2(3)	F(26)-C(86)-F(28)	107.1(3)
F(26)-C(86)-C(83)	113.1(3)	F(27)-C(86)-F(28)	105.4(3)
F(27)-C(86)-C(83)	112.8(3)	F(28)-C(86)-C(83)	111.7(3)
C(55)-B(1)-C(71)	110.1(3)	C(55)-B(1)-C(79)	113.1(3)
C(63)-B(1)-C(55)	106.0(3)	C(63)-B(1)-C(71)	114.0(3)
C(63)-B(1)-C(79)	111.0(3)	C(79)-B(1)-C(71)	102.8(3)
C(1C)-F(45B)-F(51B)#1	153.7(10)	F(50A)-C(118)-C(115)	117.1(6)

F(51A)-C(118)-F(50A)	103.0(7)	F(51A)-C(118)-F(52A)	112.1(7)
F(51A)-C(118)-C(115)	109.4(6)	F(52A)-C(118)-F(50A)	105.4(7)
F(52A)-C(118)-C(115)	109.8(6)	F(44A)-C(110)-F(45A)	105.3(5)
F(44A)-C(110)-C(107)	114.7(5)	F(45A)-C(110)-C(107)	108.7(6)
F(46A)-C(110)-F(44A)	104.0(6)	F(46A)-C(110)-F(45A)	109.3(6)
F(46A)-C(110)-C(107)	114.4(5)	F(41B)-C(109)-F(43B)	94.4(12)
F(41B)-C(109)-C(105)	120.3(13)	F(42B)-C(109)-F(41B)	107.8(16)
F(42B)-C(109)-F(43B)	102.4(15)	F(42B)-C(109)-C(105)	118.9(14)
F(43B)-C(109)-C(105)	108.6(12)	C(88)-C(87)-C(92)	116.5(3)
C(88)-C(87)-B(2)	122.2(3)	C(92)-C(87)-B(2)	120.6(3)
C(87)-C(88)-H(88)	118.9	C(87)-C(88)-C(89)	122.2(3)
C(89)-C(88)-H(88)	118.9	C(88)-C(89)-C(93)	119.0(3)
C(90)-C(89)-C(88)	120.6(4)	C(90)-C(89)-C(93)	120.3(3)
C(89)-C(90)-H(90)	120.9	C(89)-C(90)-C(91)	118.2(3)
C(91)-C(90)-H(90)	120.9	C(90)-C(91)-C(94)	118.2(3)
C(92)-C(91)-C(90)	120.9(4)	C(92)-C(91)-C(94)	120.9(4)
C(87)-C(92)-H(92)	119.2	C(91)-C(92)-C(87)	121.6(4)
C(91)-C(92)-H(92)	119.2	F(29)-C(93)-F(31)	104.9(4)
F(29)-C(93)-C(89)	112.4(3)	F(30)-C(93)-F(29)	106.9(4)
F(30)-C(93)-F(31)	106.3(4)	F(30)-C(93)-C(89)	113.7(4)
F(31)-C(93)-C(89)	112.0(4)	F(32)-C(94)-F(33)	107.3(4)
F(32)-C(94)-F(34)	107.1(4)	F(32)-C(94)-C(91)	113.2(4)
F(33)-C(94)-F(34)	104.8(4)	F(33)-C(94)-C(91)	111.6(4)
F(34)-C(94)-C(91)	112.4(4)	C(96)-C(95)-C(100)	115.7(3)
C(96)-C(95)-B(2)	122.0(3)	C(100)-C(95)-B(2)	121.8(3)
C(95)-C(96)-H(96)	118.9	C(97)-C(96)-C(95)	122.1(3)
C(97)-C(96)-H(96)	118.9	C(96)-C(97)-C(101)	120.6(3)
C(98)-C(97)-C(96)	121.1(3)	C(98)-C(97)-C(101)	118.2(3)
C(97)-C(98)-H(98)	121.0	C(97)-C(98)-C(99)	117.9(4)
C(99)-C(98)-H(98)	121.0	C(98)-C(99)-C(100)	121.1(4)
C(98)-C(99)-C(102)	118.4(4)	C(100)-C(99)-C(102)	120.6(4)
C(95)-C(100)-H(100)	119.0	C(99)-C(100)-C(95)	122.1(3)
C(99)-C(100)-H(100)	119.0	F(35)-C(101)-F(36)	105.8(3)
F(35)-C(101)-C(97)	113.1(3)	F(36)-C(101)-C(97)	111.1(3)
F(37)-C(101)-F(35)	106.3(3)	F(37)-C(101)-F(36)	106.4(3)
F(37)-C(101)-C(97)	113.6(3)	F(38)-C(102)-C(99)	111.6(4)
F(39)-C(102)-F(38)	103.8(4)	F(39)-C(102)-F(40)	109.9(4)
F(39)-C(102)-C(99)	112.6(4)	F(40)-C(102)-F(38)	104.6(4)
F(40)-C(102)-C(99)	113.6(4)	C(104)-C(103)-C(108)	115.0(4)

C(104)-C(103)-B(2)	124.6(4)	C(108)-C(103)-B(2)	120.2(3)
C(103)-C(104)-H(104)	119.2	C(105)-C(104)-C(103)	121.6(4)
C(105)-C(104)-H(104)	119.2	C(104)-C(105)-C(109)	112.0(7)
C(104)-C(105)-C(1B)	121.7(5)	C(106)-C(105)-C(109)	125.5(6)
C(106)-C(105)-C(104)	122.5(4)	C(106)-C(105)-C(1B)	112.9(5)
C(105)-C(106)-H(106)	121.8	C(105)-C(106)-C(107)	116.4(4)
C(107)-C(106)-H(106)	121.8	C(106)-C(107)-C(110)	118.7(4)
C(106)-C(107)-C(1C)	115.4(6)	C(108)-C(107)-C(110)	119.4(4)
C(108)-C(107)-C(106)	121.3(4)	C(108)-C(107)-C(1C)	118.6(6)
C(103)-C(108)-H(108)	118.8	C(107)-C(108)-C(103)	122.4(4)
C(107)-C(108)-H(108)	118.8	F(41A)-C(1B)-C(105)	103.4(7)
F(42A)-C(1B)-F(41A)	109.1(7)	F(42A)-C(1B)-C(105)	115.1(6)
F(43A)-C(1B)-F(41A)	104.5(6)	F(43A)-C(1B)-F(42A)	105.9(7)
F(43A)-C(1B)-C(105)	118.1(7)	F(44B)-C(1C)-F(45B)	103.2(11)
F(44B)-C(1C)-C(107)	111.2(11)	F(45B)-C(1C)-C(107)	119.6(11)
F(46B)-C(1C)-F(44B)	113.2(12)	F(46B)-C(1C)-F(45B)	98.1(11)
F(46B)-C(1C)-C(107)	110.9(11)	C(112)-C(111)-C(116)	116.1(3)
C(112)-C(111)-B(2)	123.2(3)	C(116)-C(111)-B(2)	120.3(3)
C(111)-C(112)-H(112)	119.0	C(111)-C(112)-C(113)	122.1(4)
C(113)-C(112)-H(112)	119.0	C(112)-C(113)-C(117)	120.1(4)
C(114)-C(113)-C(112)	120.7(4)	C(114)-C(113)-C(117)	119.2(4)
C(113)-C(114)-H(114)	121.0	C(115)-C(114)-C(113)	118.0(4)
C(115)-C(114)-H(114)	121.0	C(114)-C(115)-C(118)	124.0(5)
C(114)-C(115)-C(1D)	104.8(6)	C(116)-C(115)-C(118)	115.0(5)
C(116)-C(115)-C(114)	120.8(4)	C(116)-C(115)-C(1D)	133.1(6)
C(111)-C(116)-H(116)	118.8	C(115)-C(116)-C(111)	122.3(4)
C(115)-C(116)-H(116)	118.8	F(47A)-C(117)-C(113)	113.5(5)
F(47B)-C(117)-F(48B)	107.3(7)	F(47B)-C(117)-C(113)	112.7(5)
F(47C)-C(117)-C(113)	114.8(8)	F(48A)-C(117)-F(47A)	102.0(8)
F(48A)-C(117)-F(49A)	104.4(8)	F(48A)-C(117)-C(113)	111.1(6)
F(48B)-C(117)-C(113)	105.1(5)	F(48C)-C(117)-F(47C)	99.9(10)
F(48C)-C(117)-F(49C)	101.2(9)	F(48C)-C(117)-C(113)	122.1(8)
F(49A)-C(117)-F(47A)	107.7(8)	F(49A)-C(117)-C(113)	116.7(7)
F(49B)-C(117)-F(47B)	105.7(8)	F(49B)-C(117)-F(48B)	113.7(7)
F(49B)-C(117)-C(113)	112.3(5)	F(49C)-C(117)-F(47C)	106.2(10)
F(49C)-C(117)-C(113)	110.7(6)	F(50B)-C(1D)-C(115)	104.8(11)
F(51B)-C(1D)-F(50B)	107.7(12)	F(51B)-C(1D)-F(52B)	102.2(15)
F(51B)-C(1D)-C(115)	125.9(11)	F(52B)-C(1D)-F(50B)	101.1(15)
F(52B)-C(1D)-C(115)	112.5(17)	C(95)-B(2)-C(87)	112.4(3)

C(95)-B(2)-C(103)	113.9(3)	C(103)-B(2)-C(87)	102.9(3)
C(111)-B(2)-C(87)	112.2(3)	C(111)-B(2)-C(95)	104.3(3)
C(111)-B(2)-C(103)	111.5(3)		

_

Symmetry transformations used to generate equivalent atoms:

#1 -x+2,-y,-z

Single crystal structure analysis of complex 12 (13729)

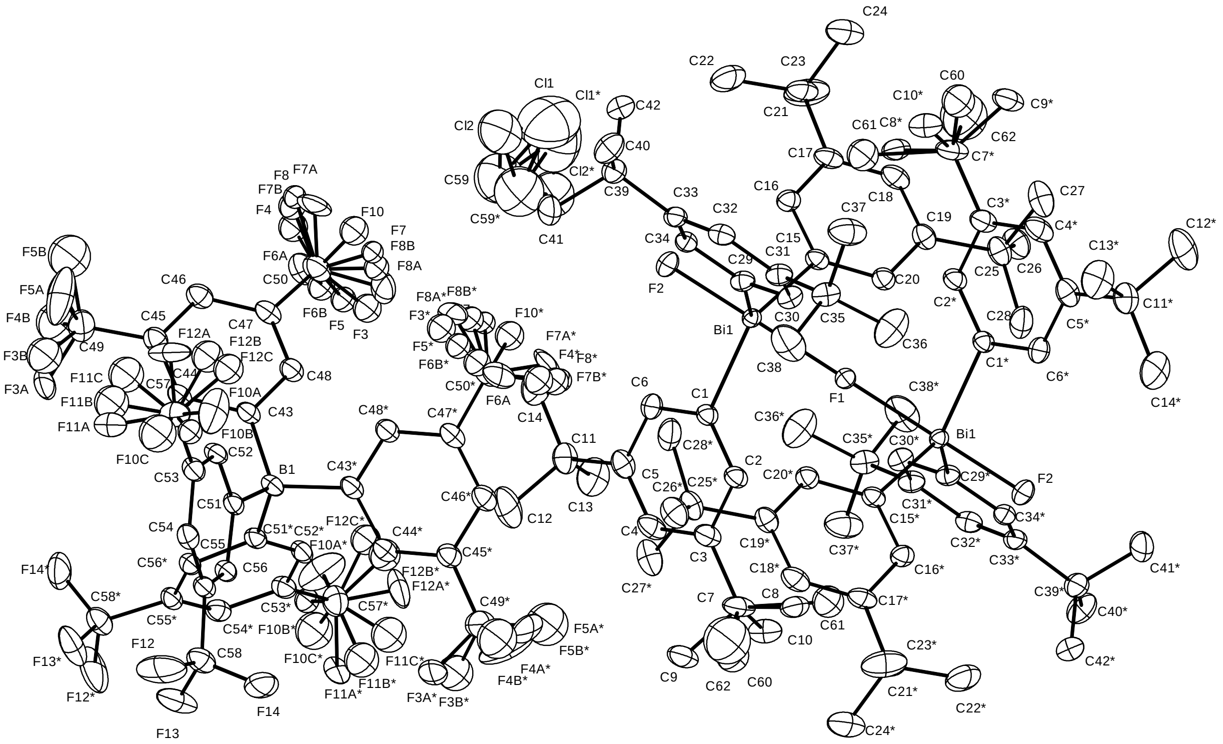

Figure S78. The molecular structure of complex **12**. H atoms have been removed for clarity. Ellipsoids are drawn at the 50% probability level.

X-ray Crystal Structure Analysis of complex 12:

C_{117} H_{140} B Bi_2 Cl_2 F_{27}, M_r = 2558.95 g mol^{-1}, colorless prism, crystal size 0.14 x 0.10 x 0.04 mm^3, monoclinic, space group *C*2/c [15], *a* = 32.230(2) Å, *b* = 17.657(2) Å, *c* = 22.107(2) Å, β= 112.272(10)°, *V* = 11642(2) Å^3, *T* = 100(2) K, *Z* = 4, D_{calc} = 1.460 g·cm^3, λ = 0.71073 Å, μ(*Mo-K*$_\alpha$) = 3.154 mm^{-1}, Gaussian absorption correction ($T_{\min}$ = 0.71132, $T_{\max}$ = 0.88533), Bruker AXS Enraf-Nonius KappaCCD diffractometer with a FR591 rotating Mo-anode X-ray source, 2.606 < θ < 30.508°, 108130 measured reflections, 17760 independent reflections, 13996 reflections with $I > 2\sigma(I)$, R_{int} = 0.0678. The structure was solved by *SHELXS* and refined by full-matrix least-squares (*SHELXL*) against F^2 to R_1 = 0.0370 [$I > 2\sigma(I)$], wR_2 = 0.0826 [all data], 792 parameters and 75 restraints.

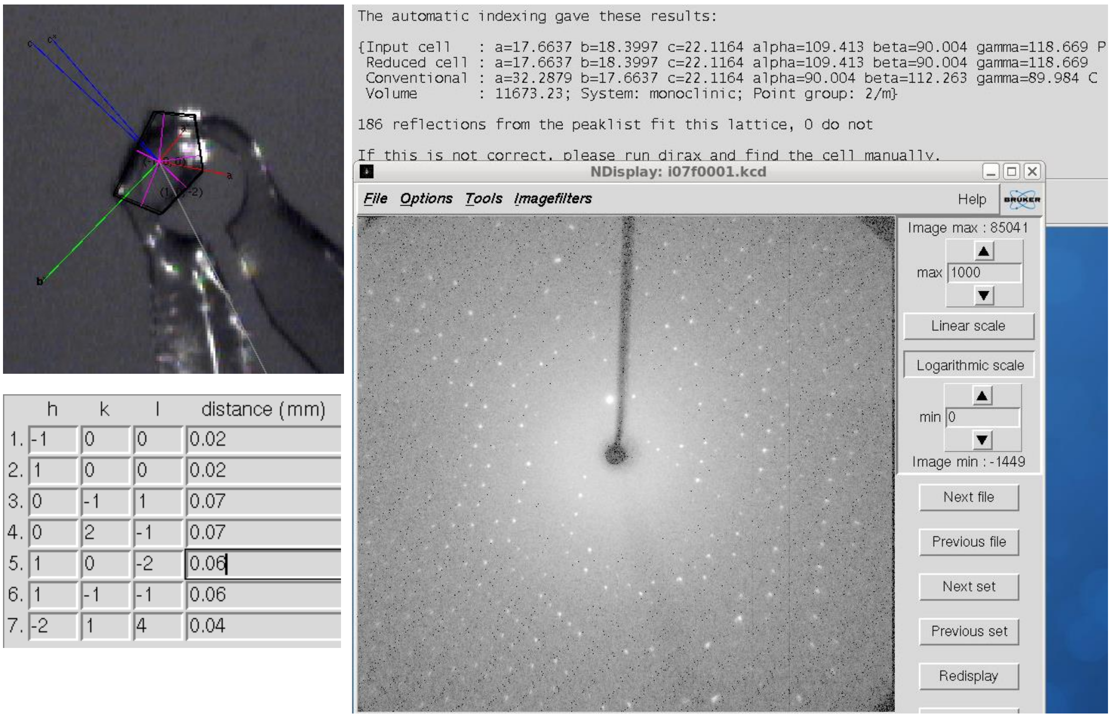

Figure S79. Crystal faces and unit cell determination/refinement of complex **12**.

INTENSITY STATISTICS FOR DATASET

Resolution	#Data	#Theory	%Complete	Redundancy	Mean I	Mean I/s	Rmerge	Rsigma
Inf - 2.67	345	361	95.6	8.43	34.64	49.99	0.0448	0.0161
2.67 - 1.78	807	807	100.0	8.10	23.59	41.97	0.0387	0.0159
1.78 - 1.41	1141	1141	100.0	7.96	15.39	35.45	0.0391	0.0190
1.41 - 1.23	1126	1126	100.0	7.71	12.31	31.02	0.0429	0.0224
1.23 - 1.11	1234	1234	100.0	7.30	9.06	24.96	0.0514	0.0286
1.11 - 1.03	1153	1153	100.0	6.89	8.92	22.84	0.0544	0.0320
1.03 - 0.97	1129	1129	100.0	6.51	7.24	19.51	0.0652	0.0388
0.97 - 0.92	1169	1169	100.0	6.17	5.77	15.97	0.0797	0.0485
0.92 - 0.88	1149	1149	100.0	5.87	5.62	14.75	0.0854	0.0532
0.88 - 0.85	1020	1020	100.0	5.64	5.05	13.09	0.0973	0.0611
0.85 - 0.82	1159	1159	100.0	5.39	4.23	11.12	0.1165	0.0738
0.82 - 0.79	1330	1331	99.9	5.14	3.78	9.55	0.1323	0.0863
0.79 - 0.77	999	999	100.0	4.93	3.44	8.54	0.1432	0.0990
0.77 - 0.75	1146	1146	100.0	4.76	3.15	7.58	0.1648	0.1130
0.75 - 0.73	1241	1241	100.0	4.54	3.04	6.85	0.1755	0.1265
0.73 - 0.71	1380	1381	99.9	4.40	2.77	6.04	0.1956	0.1464
0.71 - 0.70	755	755	100.0	4.27	2.70	5.55	0.2059	0.1622
0.70 - 0.68	1644	1644	100.0	4.16	2.24	4.46	0.2423	0.2061
0.68 - 0.67	904	904	100.0	4.02	2.08	3.91	0.2684	0.2482
0.67 - 0.66	952	952	100.0	3.92	1.78	3.11	0.3110	0.3157
0.66 - 0.65	1009	1062	95.0	3.64	1.74	2.86	0.3262	0.3397
0.75 - 0.65	7885	7939	99.3	4.16	2.37	4.78	0.2299	0.1998
Inf - 0.65	22792	22863	99.7	5.59	6.41	14.71	0.0735	0.0573

The low-order reflections have potentially been effected by the beam stop. A resolution cut-off (SHEL 99 0.7) was applied to exclude poorly determined intensities at higher diffraction angles. A resolution cut-off (SHEL 99 0.7) was applied to exclude poorly determined intensities at

higher diffraction angles. Two *tert*-butyl groups of the cationic entity show rotational disorders over two positions with occupancies of 80:20%. Three C–C distances have been restrained using DFIX instruction (DFIX 1.54 0.001 C7 C62; DFIX 1.78 0.001 C59 Cl1 and DFIX 1.78 0.001 Cl2 C59). Seven of eight CF_3-groups of BAr_F showing multiple rotational disorders over two or more positions. Thermal displacement parameters of F atoms are partially restrained using ISOR instruction (ISOR 0.01 0.02 F7A F6A F8A and ISOR 0.01 0.02 F10A F11A F12A F4A F3A F5A). Minor PARTS of disorder have been described by isotropic displacement parameters. Thermal ellipsoids of the DCM solute have been restrained using ISOR instruction (ISOR 0.001 0.002 Cl1 C59 Cl2). Complete .cif-data of the compound are available under the CCDC number **CCDC-2154892**.

Supramolecular features and coordination

Cationic units of complex **12** do not undergo any F-Bi···F-Bi interactions in the solid state.

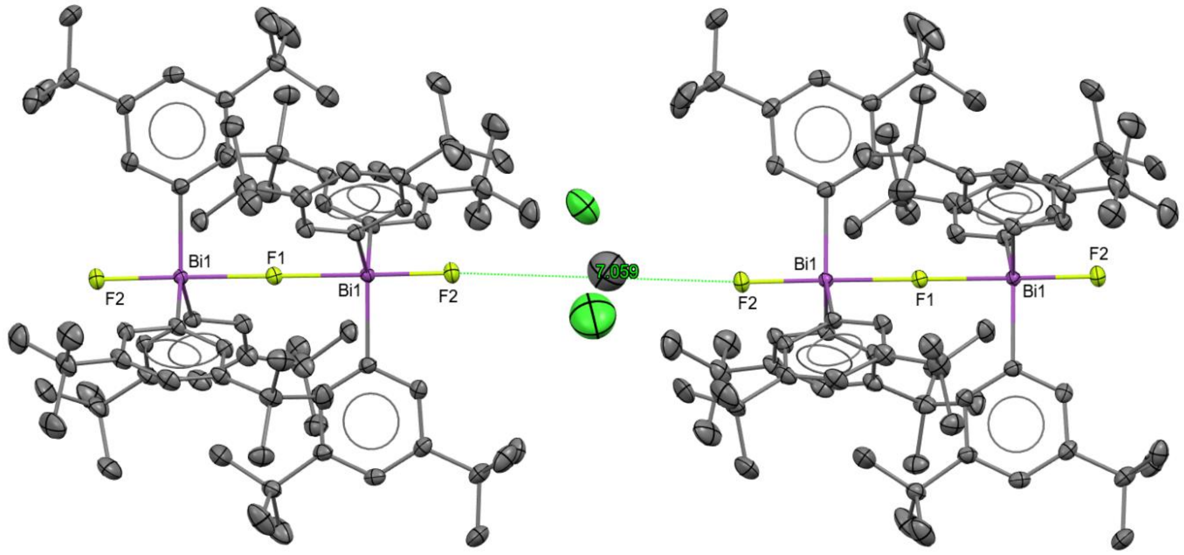

Figure S79. Intercalation of a DCM solute molecule in-between two cationic units of complex **12**. Ellipsoids are drawn at the 50% probability level.

Complex **12** does not undergo significant intermolecular F–Bi···F–Bi interactions leading to a truly monomeric structure in solid state. It is notable that one disordered solute molecule (DCM) is intercalated in between two cationic moieties. This leads to the formation of linear chains, which do not form a coordination polymer. The absence of F–Bi···F–Bi interaction leads to a nearly perfect trigonal bipyramidal coordination sphere.

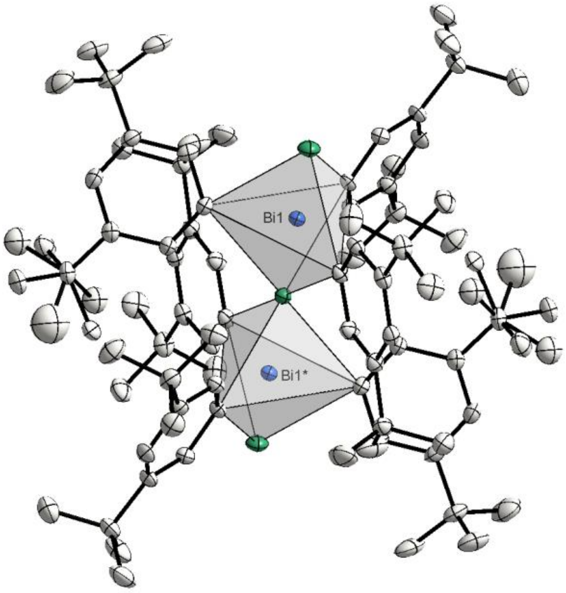

Figure S80. Polyhedral representation of trigonal bipyramidal coordination sphere of complex **12**. Ellipsoids are drawn at the 50% probability level.

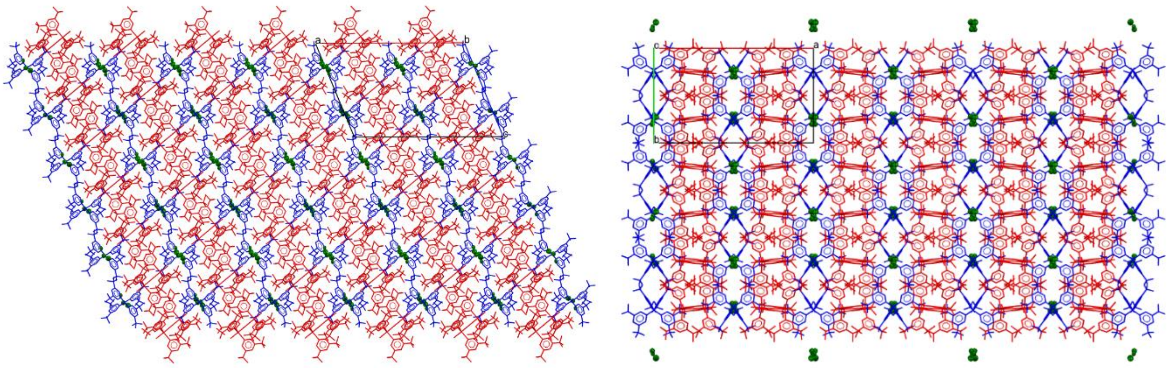

Figure S5. Layered structure of complex **12** in the solid state. Cations (red), anions (blue) and neutral DCM molecule (green) viewed along crystallographic b (left) and c (right) axis.

Unlike in the previous cationic structures **3**, **4** and **11**, no one dimensional coordination polymer is formed in **12**. This is primarily due to the steric influence of the *tert*-butyl substitution in the *meta*-position of aryl ligands. These bulky ligands do not allow close packing and the formation of intermolecular coordinative Bi···F frameworks. Instead of another cation, one solvent molecule (DCM) is intercalated in between the dimeric units for charge separation. This lead to a layered structure of cationic and anionic units.

Table S11. Crystal data and structure refinement of complex **12**.

Identification code	13729	
Empirical formula	C_{117} H_{140} B Bi_2 Cl_2 F_{27}	
Color	colourless	
Formula weight	2558.95 g·mol^{-1}	
Temperature	100(2) K	
Wavelength	0.71073 Å	
Crystal system	Monoclinic	
Space group	*C*2/c, (no. 15)	
Unit cell dimensions	a = 32.230(2) Å	α= 90°.
	b = 17.657(2) Å	β= 112.272(10)°.
	c = 22.107(2) Å	γ = 90°.
Volume	11642(2) Å^3	
Z	4	
Density (calculated)	1.460 Mg·m^{-3}	
Absorption coefficient	3.154 mm^{-1}	
F(000)	5160 e	
Crystal size	0.14 x 0.10 x 0.04 mm^3	
θ range for data collection	2.606 to 30.508°.	
Index ranges	-46 ≤ h ≤ 46, -25≤ k ≤ 25, -31 ≤ l ≤ 31	
Reflections collected	108130	
Independent reflections	17760 [R_{int} = 0.0678]	
Reflections with I>2σ(I)	13996	
Completeness to θ = 25.242°	99.9 %	
Absorption correction	Gaussian	
Max. and min. transmission	0.88533 and 0.71132	
Refinement method	Full-matrix least-squares on F^2	
Data / restraints / parameters	17760 / 75 / 792	
Goodness-of-fit on F^2	1.054	
Final R indices [I>2σ(I)]	R_1 = 0.0370	wR^2 = 0.0745
R indices (all data)	R_1 = 0.0581	wR^2 = 0.0826
Extinction coefficient	n/a	
Largest diff. peak and hole	1.980 and -1.472 e·Å^{-3}	

Table S12. Bond lengths [Å] and angles [°] of complex **12**.

C(1)-C(2)	1.381(4)	C(1)-C(6)	1.390(4)
C(1)-Bi(1)	2.214(3)	C(2)-H(2)	0.9500
C(2)-C(3)	1.405(4)	C(3)-C(4)	1.392(5)
C(3)-C(7)	1.541(5)	C(4)-H(4)	0.9500
C(4)-C(5)	1.395(5)	C(5)-C(6)	1.398(4)
C(5)-C(11)	1.532(5)	C(6)-H(6)	0.9500
C(7)-C(8)	1.508(6)	C(7)-C(9)	1.567(6)
C(7)-C(10)	1.498(6)	C(7)-C(60)	1.63(2)
C(7)-C(61)	1.78(2)	C(7)-C(62)	1.5397(10)
C(11)-C(12)	1.533(5)	C(11)-C(13)	1.527(5)
C(11)-C(14)	1.531(6)	C(12)-H(12A)	0.9800
C(12)-H(12B)	0.9800	C(12)-H(12C)	0.9800
C(13)-H(13A)	0.9800	C(13)-H(13B)	0.9800
C(13)-H(13C)	0.9800	C(14)-H(14A)	0.9800
C(14)-H(14B)	0.9800	C(14)-H(14C)	0.9800
C(15)-C(16)	1.384(4)	C(15)-C(20)	1.388(4)
C(15)-Bi(1)	2.211(3)	C(16)-H(16)	0.9500
C(16)-C(17)	1.399(4)	C(17)-C(18)	1.390(5)
C(17)-C(21)	1.528(5)	C(18)-H(18)	0.9500
C(18)-C(19)	1.397(5)	C(19)-C(20)	1.399(4)
C(19)-C(25)	1.534(5)	C(20)-H(20)	0.9500
C(21)-C(22)	1.532(6)	C(21)-C(23)	1.526(6)
C(21)-C(24)	1.538(5)	C(22)-H(22A)	0.9800
C(22)-H(22B)	0.9800	C(22)-H(22C)	0.9800
C(23)-H(23A)	0.9800	C(23)-H(23B)	0.9800
C(23)-H(23C)	0.9800	C(24)-H(24A)	0.9800
C(24)-H(24B)	0.9800	C(24)-H(24C)	0.9800
C(25)-C(26)	1.533(5)	C(25)-C(27)	1.538(5)
C(25)-C(28)	1.531(5)	C(26)-H(26A)	0.9800
C(26)-H(26B)	0.9800	C(26)-H(26C)	0.9800
C(27)-H(27A)	0.9800	C(27)-H(27B)	0.9800
C(27)-H(27C)	0.9800	C(28)-H(28A)	0.9800
C(28)-H(28B)	0.9800	C(28)-H(28C)	0.9800
C(29)-C(30)	1.392(4)	C(29)-C(34)	1.380(4)
C(29)-Bi(1)	2.213(3)	C(30)-H(30)	0.9500
C(30)-C(31)	1.392(4)	C(31)-C(32)	1.405(5)
C(31)-C(35)	1.543(5)	C(32)-H(32)	0.9500

C(32)-C(33)	1.391(5)	C(33)-C(34)	1.402(4)
C(33)-C(39)	1.530(5)	C(34)-H(34)	0.9500
C(35)-C(36)	1.529(5)	C(35)-C(37)	1.526(5)
C(35)-C(38)	1.517(5)	C(36)-H(36A)	0.9800
C(36)-H(36B)	0.9800	C(36)-H(36C)	0.9800
C(37)-H(37A)	0.9800	C(37)-H(37B)	0.9800
C(37)-H(37C)	0.9800	C(38)-H(38A)	0.9800
C(38)-H(38B)	0.9800	C(38)-H(38C)	0.9800
C(39)-C(40)	1.530(5)	C(39)-C(41)	1.538(5)
C(39)-C(42)	1.536(5)	C(40)-H(40A)	0.9800
C(40)-H(40B)	0.9800	C(40)-H(40C)	0.9800
C(41)-H(41A)	0.9800	C(41)-H(41B)	0.9800
C(41)-H(41C)	0.9800	C(42)-H(42A)	0.9800
C(42)-H(42B)	0.9800	C(42)-H(42C)	0.9800
C(43)-C(44)	1.402(5)	C(43)-C(48)	1.403(4)
C(43)-B(1)	1.635(4)	C(44)-H(44)	0.9500
C(44)-C(45)	1.388(5)	C(45)-C(46)	1.392(4)
C(45)-C(49)	1.491(5)	C(46)-H(46)	0.9500
C(46)-C(47)	1.388(5)	C(47)-C(48)	1.388(5)
C(47)-C(50)	1.501(5)	C(48)-H(48)	0.9500
C(49)-F(3A)	1.362(5)	C(49)-F(4A)	1.308(5)
C(49)-F(5A)	1.309(6)	C(49)-F(3B)	1.230(10)
C(49)-F(4B)	1.297(14)	C(49)-F(5B)	1.452(13)
C(50)-F(6A)	1.383(8)	C(50)-F(7A)	1.269(12)
C(50)-F(8A)	1.363(9)	C(50)-F(6B)	1.334(14)
C(50)-F(7B)	1.348(12)	C(50)-F(8B)	1.277(13)
C(50)-F(5)	1.298(13)	C(50)-F(7)	1.456(12)
C(50)-F(8)	1.487(18)	C(50)-F(3)	1.39(3)
C(50)-F(4)	1.26(2)	C(50)-F(10)	1.35(3)
C(51)-C(52)	1.401(5)	C(51)-C(56)	1.404(4)
C(51)-B(1)	1.636(4)	C(52)-H(52)	0.9500
C(52)-C(53)	1.389(5)	C(53)-C(54)	1.389(4)
C(53)-C(57)	1.495(5)	C(54)-H(54)	0.9500
C(54)-C(55)	1.387(5)	C(55)-C(56)	1.385(4)
C(55)-C(58)	1.505(4)	C(56)-H(56)	0.9500
C(57)-F(10A)	1.376(9)	C(57)-F(11A)	1.413(7)
C(57)-F(12A)	1.300(8)	C(57)-F(10B)	1.361(9)
C(57)-F(11B)	1.269(10)	C(57)-F(12B)	1.330(10)
C(57)-F(10C)	1.221(10)	C(57)-F(11C)	1.370(10)

C(57)-F(12C)	1.388(8)	C(58)-F(12)	1.333(4)
C(58)-F(13)	1.326(4)	C(58)-F(14)	1.324(4)
Bi(1)-F(1)	2.2820(3)	Bi(1)-F(2)	2.0565(19)
C(8)-H(8A)	0.9800	C(8)-H(8B)	0.9800
C(8)-H(8C)	0.9800	C(9)-H(9A)	0.9800
C(9)-H(9B)	0.9800	C(9)-H(9C)	0.9800
C(10)-H(10A)	0.9800	C(10)-H(10B)	0.9800
C(10)-H(10C)	0.9800	C(60)-H(60A)	0.9800
C(60)-H(60B)	0.9800	C(60)-H(60C)	0.9800
C(61)-H(61A)	0.9800	C(61)-H(61B)	0.9800
C(61)-H(61C)	0.9800	C(62)-H(62A)	0.9800
C(62)-H(62B)	0.9800	C(62)-H(62C)	0.9800
C(59)-H(59A)	0.9900	C(59)-H(59B)	0.9900
C(59)-Cl(1)	1.7797(10)	C(59)-Cl(2)	1.7805(10)

C(2)-C(1)-C(6)	123.7(3)	C(2)-C(1)-Bi(1)	122.4(2)
C(6)-C(1)-Bi(1)	113.7(2)	C(1)-C(2)-H(2)	120.8
C(1)-C(2)-C(3)	118.4(3)	C(3)-C(2)-H(2)	120.8
C(2)-C(3)-C(7)	121.6(3)	C(4)-C(3)-C(2)	117.8(3)
C(4)-C(3)-C(7)	120.5(3)	C(3)-C(4)-H(4)	118.1
C(3)-C(4)-C(5)	123.8(3)	C(5)-C(4)-H(4)	118.1
C(4)-C(5)-C(6)	117.8(3)	C(4)-C(5)-C(11)	120.6(3)
C(6)-C(5)-C(11)	121.5(3)	C(1)-C(6)-C(5)	118.5(3)
C(1)-C(6)-H(6)	120.7	C(5)-C(6)-H(6)	120.7
C(3)-C(7)-C(9)	109.2(3)	C(3)-C(7)-C(60)	113.4(9)
C(3)-C(7)-C(61)	109.2(7)	C(8)-C(7)-C(3)	113.0(3)
C(8)-C(7)-C(9)	107.0(3)	C(10)-C(7)-C(3)	108.2(3)
C(10)-C(7)-C(8)	110.2(4)	C(10)-C(7)-C(9)	109.2(3)
C(60)-C(7)-C(61)	90.0(11)	C(62)-C(7)-C(3)	116.7(12)
C(62)-C(7)-C(60)	116.7(15)	C(62)-C(7)-C(61)	107.0(13)
C(5)-C(11)-C(12)	110.6(3)	C(13)-C(11)-C(5)	107.5(3)
C(13)-C(11)-C(12)	109.5(3)	C(13)-C(11)-C(14)	109.6(3)
C(14)-C(11)-C(5)	111.4(3)	C(14)-C(11)-C(12)	108.1(4)
C(11)-C(12)-H(12A)	109.5	C(11)-C(12)-H(12B)	109.5
C(11)-C(12)-H(12C)	109.5	H(12A)-C(12)-H(12B)	109.5
H(12A)-C(12)-H(12C)	109.5	H(12B)-C(12)-H(12C)	109.5
C(11)-C(13)-H(13A)	109.5	C(11)-C(13)-H(13B)	109.5
C(11)-C(13)-H(13C)	109.5	H(13A)-C(13)-H(13B)	109.5
H(13A)-C(13)-H(13C)	109.5	H(13B)-C(13)-H(13C)	109.5

C(11)-C(14)-H(14A)	109.5	C(11)-C(14)-H(14B)	109.5
C(11)-C(14)-H(14C)	109.5	H(14A)-C(14)-H(14B)	109.5
H(14A)-C(14)-H(14C)	109.5	H(14B)-C(14)-H(14C)	109.5
C(16)-C(15)-C(20)	123.2(3)	C(16)-C(15)-Bi(1) 115.2(2)	C(20)-
C(15)-Bi(1)	121.4(2)	C(15)-C(16)-H(16)	120.7
C(15)-C(16)-C(17)	118.5(3)	C(17)-C(16)-H(16)	120.7
C(16)-C(17)-C(21)	122.1(3)	C(18)-C(17)-C(16)	118.2(3)
C(18)-C(17)-C(21)	119.6(3)	C(17)-C(18)-H(18)	118.2
C(17)-C(18)-C(19)	123.5(3)	C(19)-C(18)-H(18)	118.2
C(18)-C(19)-C(20)	117.6(3)	C(18)-C(19)-C(25)	119.5(3)
C(20)-C(19)-C(25)	122.7(3)	C(15)-C(20)-C(19)	118.9(3)
C(15)-C(20)-H(20)	120.6	C(19)-C(20)-H(20)	120.6
C(17)-C(21)-C(22)	112.2(3)	C(17)-C(21)-C(24)	109.2(3)
C(22)-C(21)-C(24)	108.3(3)	C(23)-C(21)-C(17)	109.7(3)
C(23)-C(21)-C(22)	107.8(4)	C(23)-C(21)-C(24)	109.6(3)
C(21)-C(22)-H(22A)	109.5	C(21)-C(22)-H(22B)	109.5
C(21)-C(22)-H(22C)	109.5	H(22A)-C(22)-H(22B)	109.5
H(22A)-C(22)-H(22C)	109.5	H(22B)-C(22)-H(22C)	109.5
C(21)-C(23)-H(23A)	109.5	C(21)-C(23)-H(23B)	109.5
C(21)-C(23)-H(23C)	109.5	H(23A)-C(23)-H(23B)	109.5
H(23A)-C(23)-H(23C)	109.5	H(23B)-C(23)-H(23C)	109.5
C(21)-C(24)-H(24A)	109.5	C(21)-C(24)-H(24B)	109.5
C(21)-C(24)-H(24C)	109.5	H(24A)-C(24)-H(24B)	109.5
H(24A)-C(24)-H(24C)	109.5	H(24B)-C(24)-H(24C)	109.5
C(19)-C(25)-C(27)	108.1(3)	C(26)-C(25)-C(19)	110.5(3)
C(26)-C(25)-C(27)	109.2(3)	C(28)-C(25)-C(19)	113.1(3)
C(28)-C(25)-C(26)	108.1(3)	C(28)-C(25)-C(27)	107.8(3)
C(25)-C(26)-H(26A)	109.5	C(25)-C(26)-H(26B)	109.5
C(25)-C(26)-H(26C)	109.5	H(26A)-C(26)-H(26B)	109.5
H(26A)-C(26)-H(26C)	109.5	H(26B)-C(26)-H(26C)	109.5
C(25)-C(27)-H(27A)	109.5	C(25)-C(27)-H(27B)	109.5
C(25)-C(27)-H(27C)	109.5	H(27A)-C(27)-H(27B)	109.5
H(27A)-C(27)-H(27C)	109.5	H(27B)-C(27)-H(27C)	109.5
C(25)-C(28)-H(28A)	109.5	C(25)-C(28)-H(28B)	109.5
C(25)-C(28)-H(28C)	109.5	H(28A)-C(28)-H(28B)	109.5
H(28A)-C(28)-H(28C)	109.5	H(28B)-C(28)-H(28C)	109.5
C(30)-C(29)-Bi(1)	122.3(2)	C(34)-C(29)-C(30)	123.0(3)
C(34)-C(29)-Bi(1)	114.7(2)	C(29)-C(30)-H(30)	120.5
C(29)-C(30)-C(31)	118.9(3)	C(31)-C(30)-H(30)	120.5

C(30)-C(31)-C(32)	118.0(3)	C(30)-C(31)-C(35)	122.6(3)
C(32)-C(31)-C(35)	119.3(3)	C(31)-C(32)-H(32)	118.5
C(33)-C(32)-C(31)	123.0(3)	C(33)-C(32)-H(32)	118.5
C(32)-C(33)-C(34)	118.0(3)	C(32)-C(33)-C(39)	123.4(3)
C(34)-C(33)-C(39)	118.5(3)	C(29)-C(34)-C(33)	119.0(3)
C(29)-C(34)-H(34)	120.5	C(33)-C(34)-H(34)	120.5
C(36)-C(35)-C(31)	112.0(3)	C(37)-C(35)-C(31)	111.2(3)
C(37)-C(35)-C(36)	106.6(3)	C(38)-C(35)-C(31)	108.8(3)
C(38)-C(35)-C(36)	109.3(3)	C(38)-C(35)-C(37)	108.9(3)
C(35)-C(36)-H(36A)	109.5	C(35)-C(36)-H(36B)	109.5
C(35)-C(36)-H(36C)	109.5	H(36A)-C(36)-H(36B)	109.5
H(36A)-C(36)-H(36C)	109.5	H(36B)-C(36)-H(36C)	109.5
C(35)-C(37)-H(37A)	109.5	C(35)-C(37)-H(37B)	109.5
C(35)-C(37)-H(37C)	109.5	H(37A)-C(37)-H(37B)	109.5
H(37A)-C(37)-H(37C)	109.5	H(37B)-C(37)-H(37C)	109.5
C(35)-C(38)-H(38A)	109.5	C(35)-C(38)-H(38B)	109.5
C(35)-C(38)-H(38C)	109.5	H(38A)-C(38)-H(38B)	109.5
H(38A)-C(38)-H(38C)	109.5	H(38B)-C(38)-H(38C)	109.5
C(33)-C(39)-C(41)	109.2(3)	C(33)-C(39)-C(42)	108.5(3)
C(40)-C(39)-C(33)	112.4(3)	C(40)-C(39)-C(41)	108.7(3)
C(40)-C(39)-C(42)	108.3(3)	C(42)-C(39)-C(41)	109.7(3)
C(39)-C(40)-H(40A)	109.5	C(39)-C(40)-H(40B)	109.5
C(39)-C(40)-H(40C)	109.5	H(40A)-C(40)-H(40B)	109.5
H(40A)-C(40)-H(40C)	109.5	H(40B)-C(40)-H(40C)	109.5
C(39)-C(41)-H(41A)	109.5	C(39)-C(41)-H(41B)	109.5
C(39)-C(41)-H(41C)	109.5	H(41A)-C(41)-H(41B)	109.5
H(41A)-C(41)-H(41C)	109.5	H(41B)-C(41)-H(41C)	109.5
C(39)-C(42)-H(42A)	109.5	C(39)-C(42)-H(42B)	109.5
C(39)-C(42)-H(42C)	109.5	H(42A)-C(42)-H(42B)	109.5
H(42A)-C(42)-H(42C)	109.5	H(42B)-C(42)-H(42C)	109.5
C(44)-C(43)-C(48)	115.8(3)	C(44)-C(43)-B(1) 120.0(3)	C(48)-
C(43)-B(1)	123.5(3)	C(43)-C(44)-H(44)	118.9
C(45)-C(44)-C(43)	122.2(3)	C(45)-C(44)-H(44)	118.9
C(44)-C(45)-C(46)	121.0(3)	C(44)-C(45)-C(49)	119.0(3)
C(46)-C(45)-C(49)	120.0(3)	C(45)-C(46)-H(46)	121.2
C(47)-C(46)-C(45)	117.6(3)	C(47)-C(46)-H(46)	121.2
C(46)-C(47)-C(50)	119.3(3)	C(48)-C(47)-C(46)	121.2(3)
C(48)-C(47)-C(50)	119.5(3)	C(43)-C(48)-H(48)	119.0
C(47)-C(48)-C(43)	122.1(3)	C(47)-C(48)-H(48)	119.0

F(3A)-C(49)-C(45)	111.5(3)	F(4A)-C(49)-C(45)	114.8(3)
F(4A)-C(49)-F(3A)	105.9(4)	F(4A)-C(49)-F(5A)	107.9(5)
F(5A)-C(49)-C(45)	112.4(4)	F(5A)-C(49)-F(3A)	103.5(4)
F(3B)-C(49)-C(45)	119.3(6)	F(3B)-C(49)-F(4B)	113.2(8)
F(3B)-C(49)-F(5B)	99.1(8)	F(4B)-C(49)-C(45)	113.2(7)
F(4B)-C(49)-F(5B)	99.5(8)	F(5B)-C(49)-C(45)	109.4(6)
F(6A)-C(50)-C(47)	111.5(4)	F(7A)-C(50)-C(47)	116.2(6)
F(7A)-C(50)-F(6A)	100.9(8)	F(7A)-C(50)-F(8A)	107.2(7)
F(8A)-C(50)-C(47)	115.3(4)	F(8A)-C(50)-F(6A)	104.1(7)
F(6B)-C(50)-C(47)	111.8(6)	F(6B)-C(50)-F(7B)	95.1(8)
F(7B)-C(50)-C(47)	112.6(6)	F(8B)-C(50)-C(47)	113.8(6)
F(8B)-C(50)-F(6B)	108.6(8)	F(8B)-C(50)-F(7B)	113.4(7)
F(5)-C(50)-C(47)	112.7(6)	F(5)-C(50)-F(7)	106.6(9)
F(5)-C(50)-F(8)	131.5(11)	F(7)-C(50)-C(47)	107.5(5)
F(7)-C(50)-F(8)	86.2(10)	F(8)-C(50)-C(47)	106.9(7)
F(3)-C(50)-C(47)	110.6(11)	F(4)-C(50)-C(47)	117.9(11)
F(4)-C(50)-F(3)	110.1(18)	F(4)-C(50)-F(10)	105.5(18)
F(10)-C(50)-C(47)	110.5(11)	F(10)-C(50)-F(3)	100.9(18)
C(52)-C(51)-C(56)	115.3(3)	C(52)-C(51)-B(1)	122.3(3)
C(56)-C(51)-B(1)	121.6(3)	C(51)-C(52)-H(52)	118.8
C(53)-C(52)-C(51)	122.4(3)	C(53)-C(52)-H(52)	118.8
C(52)-C(53)-C(57)	120.5(3)	C(54)-C(53)-C(52)	120.9(3)
C(54)-C(53)-C(57)	118.6(3)	C(53)-C(54)-H(54)	121.1
C(55)-C(54)-C(53)	117.8(3)	C(55)-C(54)-H(54)	121.1
C(54)-C(55)-C(58)	119.3(3)	C(56)-C(55)-C(54)	121.0(3)
C(56)-C(55)-C(58)	119.6(3)	C(51)-C(56)-H(56)	118.8
C(55)-C(56)-C(51)	122.5(3)	C(55)-C(56)-H(56)	118.8
F(10A)-C(57)-C(53)	115.0(5)	F(10A)-C(57)-F(11A)	99.8(6)
F(11A)-C(57)-C(53)	112.4(4)	F(12A)-C(57)-C(53)	118.7(4)
F(12A)-C(57)-F(10A)	106.3(8)	F(12A)-C(57)-F(11A)	102.4(6)
F(10B)-C(57)-C(53)	111.3(4)	F(11B)-C(57)-C(53)	112.7(5)
F(11B)-C(57)-F(10B)	104.5(7)	F(11B)-C(57)-F(12B)	109.1(7)
F(12B)-C(57)-C(53)	112.9(5)	F(12B)-C(57)-F(10B)	105.8(6)
F(10C)-C(57)-C(53)	115.3(5)	F(10C)-C(57)-F(11C)	112.0(8)
F(10C)-C(57)-F(12C)	111.6(7)	F(11C)-C(57)-C(53)	108.3(5)
F(11C)-C(57)-F(12C)	100.8(6)	F(12C)-C(57)-C(53)	107.8(4)
F(12)-C(58)-C(55)	112.2(3)	F(13)-C(58)-C(55)	112.8(3)
F(13)-C(58)-F(12)	106.4(3)	F(14)-C(58)-C(55)	112.4(3)
F(14)-C(58)-F(12)	106.5(3)	F(14)-C(58)-F(13)	106.1(3)

C(43)-B(1)-C(43)#1	114.3(4)	C(43)-B(1)-C(51)#1	112.87(16)
C(43)-B(1)-C(51)	101.77(15)	C(43)#1-B(1)-C(51)#1	101.77(15)
C(43)#1-B(1)-C(51)	112.87(16)	C(51)#1-B(1)-C(51)	113.8(4)
C(1)-Bi(1)-F(1)	87.69(8)	C(15)-Bi(1)-C(1)	118.99(11)
C(15)-Bi(1)-C(29)	117.58(11)	C(15)-Bi(1)-F(1)	88.14(8)
C(29)-Bi(1)-C(1)	123.14(11)	C(29)-Bi(1)-F(1)	88.90(8
F(2)-Bi(1)-C(1)	91.85(10)	F(2)-Bi(1)-C(15)	91.64(10)
F(2)-Bi(1)-C(29)	91.78(10)	F(2)-Bi(1)-F(1)	179.31(5)
Bi(1)-F(1)-Bi(1)#2	180.0	C(7)-C(8)-H(8A)	109.5
C(7)-C(8)-H(8B)	109.5	C(7)-C(8)-H(8C)	109.5
H(8A)-C(8)-H(8B)	109.5	H(8A)-C(8)-H(8C)	109.5
H(8B)-C(8)-H(8C)	109.5	C(7)-C(9)-H(9A)	109.5
C(7)-C(9)-H(9B)	109.5	C(7)-C(9)-H(9C)	109.5
H(9A)-C(9)-H(9B)	109.5	H(9A)-C(9)-H(9C)	109.5
H(9B)-C(9)-H(9C)	109.5	C(7)-C(10)-H(10A)	109.5
C(7)-C(10)-H(10B)	109.5	C(7)-C(10)-H(10C)	109.5
H(10A)-C(10)-H(10B)	109.5	H(10A)-C(10)-H(10C)	109.5
H(10B)-C(10)-H(10C)	109.5	C(7)-C(60)-H(60A)	109.5
C(7)-C(60)-H(60B)	109.5	C(7)-C(60)-H(60C)	109.5
H(60A)-C(60)-H(60B)	109.5	H(60A)-C(60)-H(60C)	109.5
H(60B)-C(60)-H(60C)	109.5	C(7)-C(61)-H(61A)	109.5
C(7)-C(61)-H(61B)	109.5	C(7)-C(61)-H(61C)	109.5
H(61A)-C(61)-H(61B)	109.5	H(61A)-C(61)-H(61C)	109.5
H(61B)-C(61)-H(61C)	109.5	C(7)-C(62)-H(62A)	109.5
C(7)-C(62)-H(62B)	109.5	C(7)-C(62)-H(62C)	109.5
H(62A)-C(62)-H(62B)	109.5	H(62A)-C(62)-H(62C)	109.5
H(62B)-C(62)-H(62C)	109.5	H(59A)-C(59)-H(59B)	109.2
Cl(1)-C(59)-H(59A)	111.2	Cl(1)-C(59)-H(59B)	111.2
Cl(1)-C(59)-Cl(2)	102.6(3)	Cl(2)-C(59)-H(59A)	111.2
Cl(2)-C(59)-H(59B)	111.2		

_

Symmetry transformations used to generate equivalent atoms:

#1 -x+1,y,-z+1/2   #2 -x+3/2,-y+1/2,-z+1

Single crystal structure analysis of complex 13 (14260)

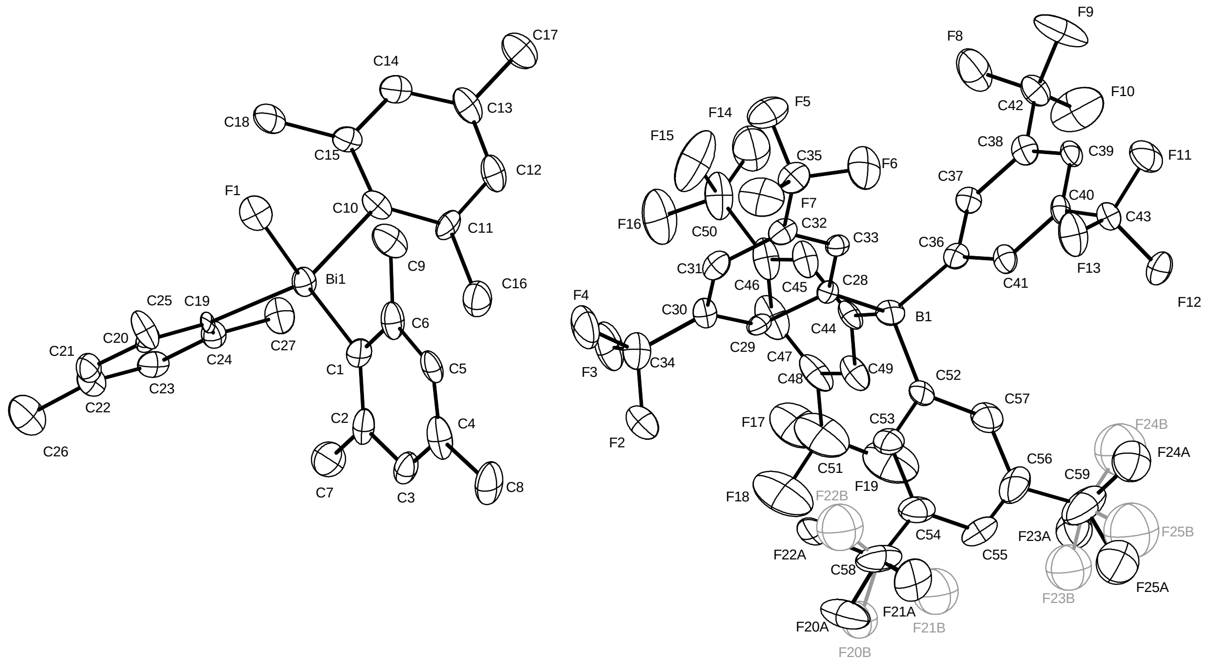

Figure S81. The molecular structure of complex **13**. H atoms have been removed for clarity and disordered parts are shown in grey. Ellipsoids are drawn at the 50% probability level.

X-ray Crystal Structure Analysis of complex 13:

C_{59} H_{45} B Bi F_{25}, M_r = 1448.74 g mol^{-1}, colorless block, crystal size 0.14 x 0.06 x 0.02 mm^3, monoclinic, space group *P*-1 [2], *a* = 12.837(2) Å, *b* = 15.1001(10) Å, *c* = 16.695(2) Å, α = 73.707(7)°, β= 72.858(8)°, γ = 75.567(7)°, *V* = 2919.0(6) Å^3, *T* = 100(2) K, *Z* = 2, D_{calc} = 1.648 g·cm^3, λ = 0.71073 Å, $\mu(Mo\text{-}K_\alpha)$ = 3.138 mm^{-1}, Gaussian absorption correction ($T_{\min}$ = 0.64876, $T_{\max}$ = 0.94153), Bruker AXS Enraf-Nonius KappaCCD diffractometer with a FR591 rotating Mo-anode X-ray source, 2.620 < θ < 24.712°, 25680 measured reflections, 9715 independent reflections, 7420 reflections with $I > 2\sigma(I)$, R_{int} = 0.0721. The structure was solved by *SHELXS* and refined by full-matrix least-squares (*SHELXL*) against F^2 to R_1 = 0.0573 [$I > 2\sigma(I)$], wR_2 = 0.1356 [all data], 808 parameters and 45 restraints.

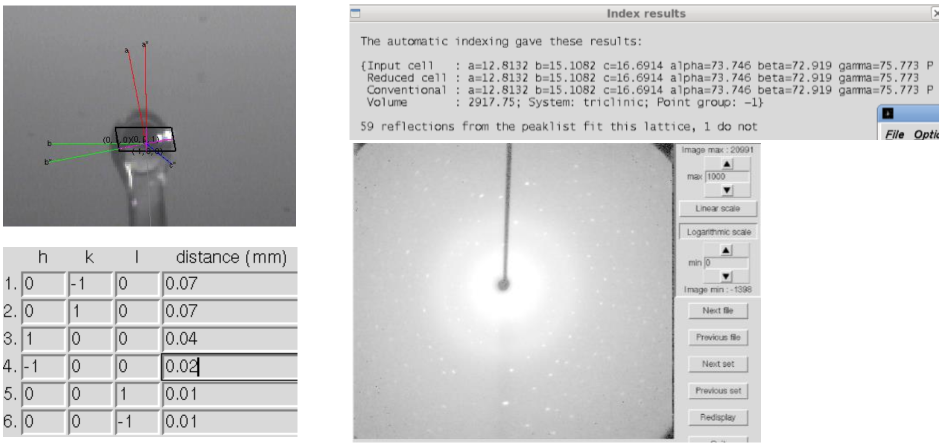

Figure S82. Crystal faces and unit cell determination/refinement of complex **13**.

INTENSITY STATISTICS FOR DATASET

Resolution	#Data	#Theory	%Complete	Redundancy	Mean I	Mean I/s	Rmerge	Rsigma
Inf - 2.68	299	316	94.6	3.92	99.34	23.71	0.0522	0.0361
2.68 - 1.81	703	712	98.7	4.08	65.51	21.41	0.0491	0.0400
1.81 - 1.44	1016	1029	98.7	3.76	41.30	18.06	0.0575	0.0507
1.44 - 1.26	987	991	99.6	3.04	28.67	13.59	0.0700	0.0699
1.26 - 1.14	1060	1074	98.7	2.62	22.50	10.85	0.0845	0.0886
1.14 - 1.06	992	1007	98.5	2.47	18.83	9.25	0.1004	0.1059
1.06 - 1.00	954	980	97.3	2.22	15.84	7.70	0.1162	0.1318
1.00 - 0.95	1028	1043	98.6	1.97	13.68	6.15	0.1311	0.1668
0.95 - 0.90	1206	1254	96.2	1.88	10.85	5.08	0.1585	0.2080
0.90 - 0.87	849	890	95.4	1.81	8.99	4.12	0.1757	0.2589
0.87 - 0.84	945	1002	94.3	1.72	7.43	3.34	0.2141	0.3221
0.84 - 0.81	1115	1213	91.9	1.63	6.82	2.92	0.2350	0.3641
0.81 - 0.79	789	881	89.6	1.52	6.08	2.42	0.2901	0.4302
0.79 - 0.76	1321	1521	86.9	1.43	4.99	1.95	0.3417	0.5556
0.76 - 0.74	992	1179	84.1	1.32	4.42	1.59	0.3894	0.6604
0.74 - 0.72	1074	1284	83.6	1.25	4.01	1.36	0.4897	0.7537
0.72 - 0.70	1167	1456	80.2	1.18	3.49	1.17	0.4984	0.8763
0.70 - 0.69	628	771	81.5	1.18	3.14	1.07	0.5487	0.9836
0.69 - 0.67	1358	1733	78.4	1.11	2.78	0.89	0.6296	1.1320
0.67 - 0.66	707	909	77.8	1.04	2.13	0.70	0.6379	1.5104
0.66 - 0.65	723	1083	66.8	0.89	2.27	0.73	0.5841	1.4369
0.75 - 0.65	6172	7840	78.7	1.13	3.13	1.04	0.5375	0.9931
Inf - 0.65	19913	22328	89.2	1.85	14.37	5.83	0.0882	0.1799

The obtained crystall was of small size (plate; crystal size: 0.14 mm x 0.06 mm x 0.02 mm) and the measured intensity was low at higher diffraction angles. Therefore, a resolution cut off (SHEL 99 0.85) was applied, to exclude poorly determined intensities. The BAr^F anion shows a rotational disorder at three different CF_3 groups. Two of them could be treated with fixed occupancies of 50:50%. ISOR instruction was used to improve thermal ellipsoids of three F atoms (ISOR 0.001 0.002 F25A F24A F23A). The third disorder could not be described in a proper way due to low resolution. DELU, SIMU and DFIX instructions where used for description (DELU 0.001 0.002 F19 C51 F17 F18; SIMU 0.001 0.002 2 F19 C51 F17 F18; DFIX 1.38 0.001 C51 F17; DFIX 1.38 0.001 C51 F18; DFIX 1.38 0.001 C51 F19). Complete .cif-data of the compound are available under the CCDC number **CCDC-2154893**.

Supramolecular features and coordination

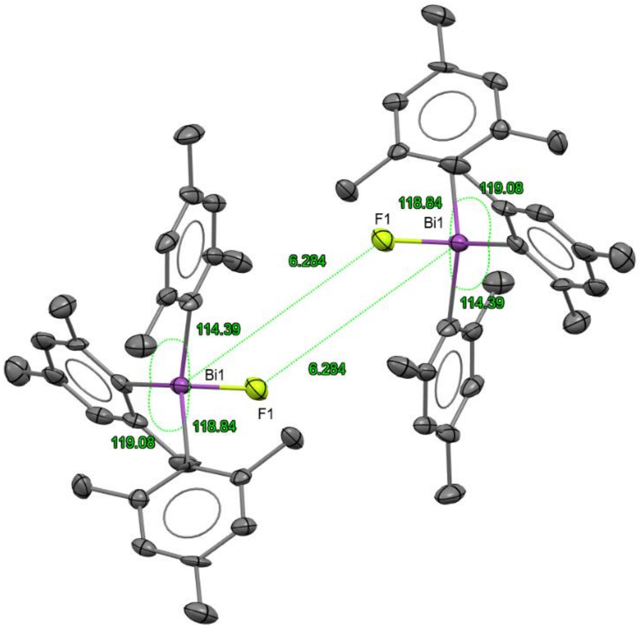

Figure S83. Two closest cationic units of complex **13** in the solid state. Ellipsoids are drawn at the 50% probability level.

Complex **13** does not undergo significant intermolecular F–Bi···F–Bi interactions leading to a truly monomeric structure in solid state. The closest intermolecular Bi···F distance is 6.284 Å and too long for coordinative interactions. Additionally no significant angular distortion (like in **3**, **4** and **11**) could be observed. It is worth noting, that here both fluorine atoms are directed towards each other and do not point in the same direction as in the polymeric structure IWEPOR.[7]

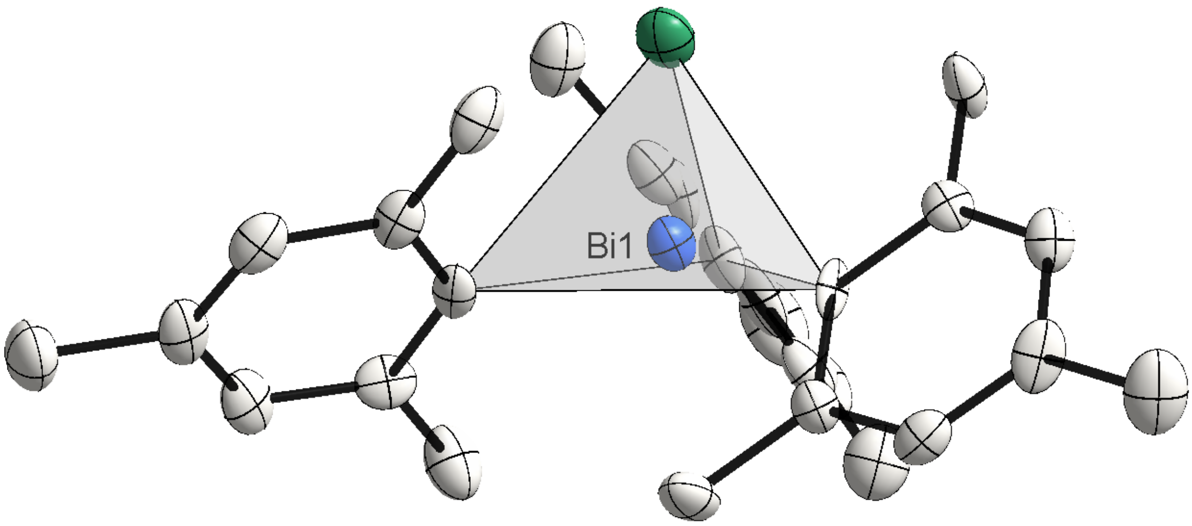

Figure S84. Polyhedral representation of distorted tetrahedral coordination sphere of complex **13**. Ellipsoids are drawn at the 50% probability level.

The absence of F–Bi···F–Bi interaction lead to a distorted tetragonal coordination sphere.

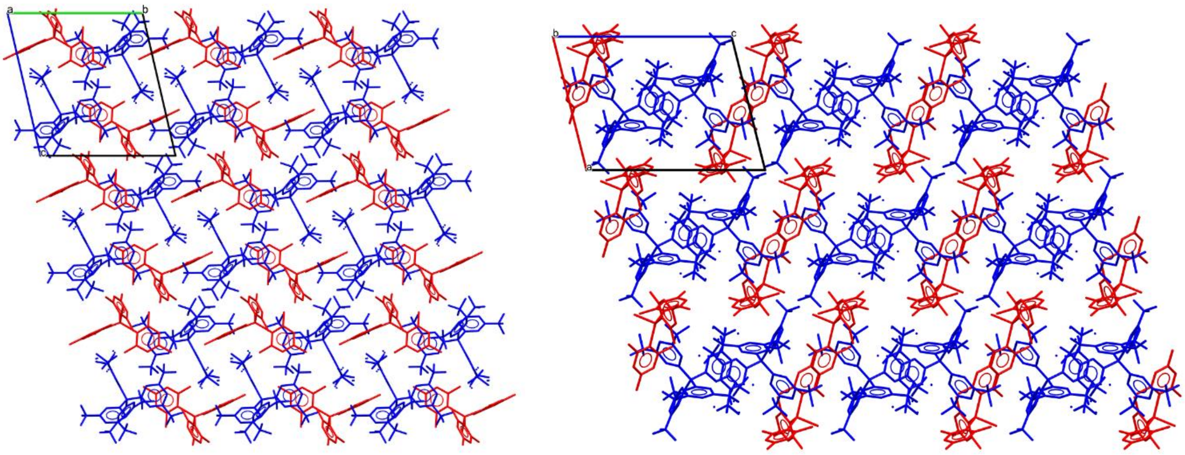

Figure S85. Packing of complex **13** in the solid state. Viewing direction along crystallographic a (left) and b (right) axis, cations are shown in red and anions are shown in blue.

Caused by the absence of intermolecular Bi···F interactions, no cationic chains were found in the packing of complex **13**.

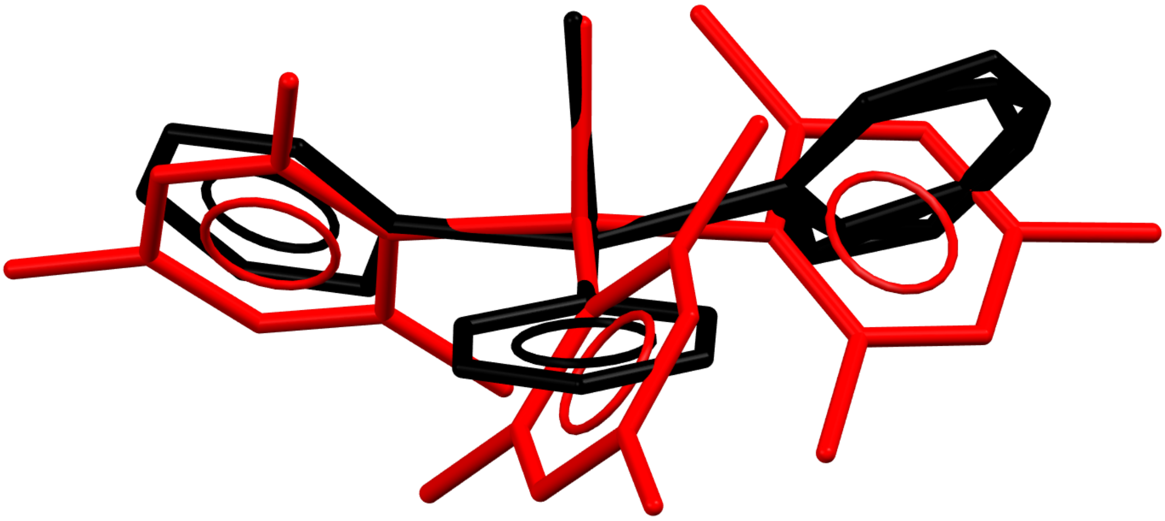

Figure S86. Structure overlay of cationic complex **13** (red) and polymeric IWEPOR (black).

Geometrical comparisons of cationic and monomeric complex **13** with the only other known Bi(V) mono fluoride shows significant differences.[7] The structural overlay lead to an RMS value of 0.144. Despite the differences in torsion angles (**13** propeller structure as in other neutral complexes *e.g.* **9** and **10**) of aryl ligands a remarkable difference in bond length could be found. The polymeric structure of IWEPOR has Bi–C distances of 2.178 Å and 2.172 Å in contrast to this, in complex **13** slightly elongated to 2.213 Å and 2.223 Å. The biggest differences could be observed in the Bi–F distances which are 2.268 Å for the polymeric

IWEPOR and 2.009 Å for monomeric complex **13**. The shorter Bi–F distances in **13** could be explained by the presence of only one F atom attached to the Bi(V) central atom.

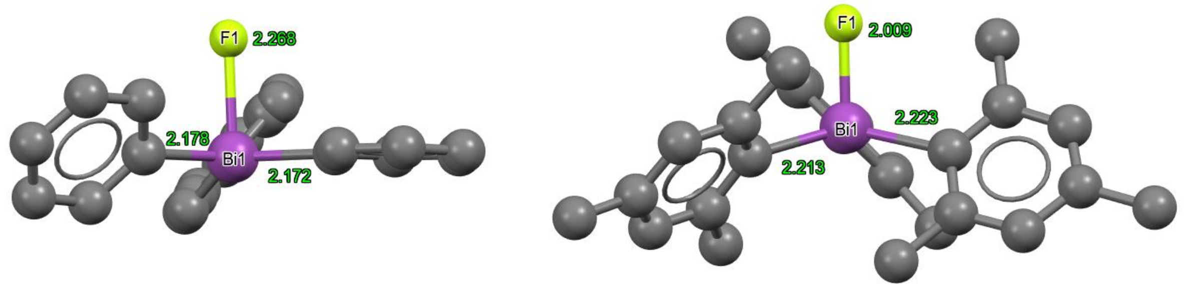

Figure S87. Comparison of bond length of IWEPOR (left) and complex **13** (right).

These geometrical and coordinative differences in both cationic structures lead to two different coordination spheres of the Bi(V) central atoms. Because of the absence of any polymeric Bi···F interactions, the Bi(V) central atom in complex **13** is coordinated in a distorted tetrahedral coordination sphere. In contrast to this, IWEPOR shows a trigonal-bipyramidal coordination sphere around the Bi(V) central atom.

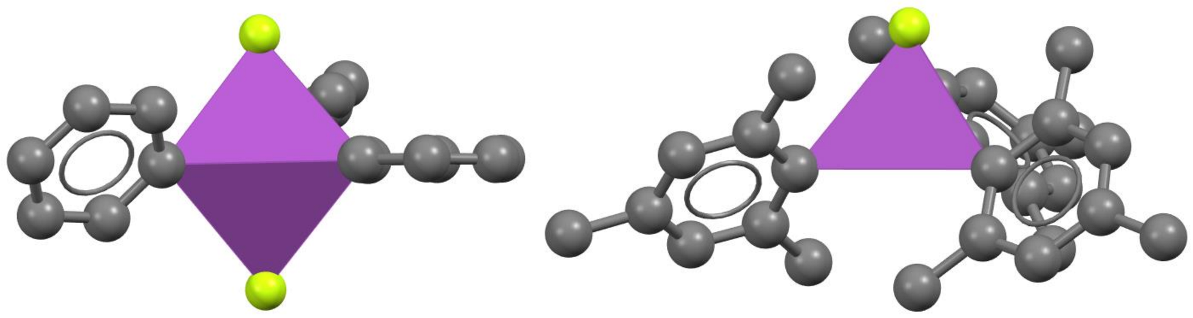

Figure S88. Different coordination spheres of IWEPOR (left) and complex **13** (right) in polyhedral representation.

Table S13. Crystal data and structure refinement of complex **13**.

Identification code	14260	
Empirical formula	$C_{59} H_{45} B Bi F_{25}$	
Color	colourless	
Formula weight	1448.74 g·mol^{-1}	
Temperature	100(2) K	
Wavelength	0.71073 Å	
Crystal system	Triclinic	
Space group	*P*-1, (no. 2)	
Unit cell dimensions	a = 12.837(2) Å	α= 73.707(7)°.
	b = 15.1001(10) Å	β= 72.858(8)°.
	c = 16.695(2) Å	γ = 75.567(7)°.
Volume	2919.0(6) Å^3	
Z	2	
Density (calculated)	1.648 Mg·m^{-3}	
Absorption coefficient	3.138 mm^{-1}	
F(000)	1424 e	
Crystal size	0.14 x 0.06 x 0.02 mm^3	
θ range for data collection	2.620 to 24.712°.	
Index ranges	-12 ≤ h ≤ 15, -15≤ k ≤ 17, -17 ≤ l ≤ 19	
Reflections collected	25680	
Independent reflections	9715 [R_{int} = 0.0721]	
Reflections with I>2σ(I)	7420	
Completeness to θ = 24.712°	97.6 %	
Absorption correction	Gaussian	
Max. and min. transmission	0.94153 and 0.64876	
Refinement method	Full-matrix least-squares on F^2	
Data / restraints / parameters	9715 / 45 / 808	
Goodness-of-fit on F^2	1.028	
Final R indices [I>2σ(I)]	R_1 = 0.0573	wR^2 = 0.1194
R indices (all data)	R_1 = 0.0917	wR^2 = 0.1356
Extinction coefficient	n/a	
Largest diff. peak and hole	1.499 and -1.521 e·Å^{-3}	

Table S14. Bond lengths [Å] and angles [°] of complex **13**.

_

Bi(1)-F(1)	2.009(5)	Bi(1)-C(1)	2.222(8)
Bi(1)-C(10)	2.223(8)	Bi(1)-C(19)	2.213(7)
F(2)-C(34)	1.348(11)	F(3)-C(34)	1.339(11)
F(4)-C(34)	1.350(10)	F(5)-C(35)	1.341(11)
F(6)-C(35)	1.341(11)	F(7)-C(35)	1.337(10)
F(8)-C(42)	1.323(11)	F(9)-C(42)	1.322(10)
F(10)-C(42)	1.311(11)	F(11)-C(43)	1.338(10)
F(12)-C(43)	1.356(10)	F(13)-C(43)	1.346(10)
F(14)-C(50)	1.319(12)	F(15)-C(50)	1.329(11)
F(16)-C(50)	1.346(11)	F(17)-C(51)	1.3798(11)
F(18)-C(51)	1.3794(11)	F(19)-C(51)	1.3797(10)
F(20A)-C(58)	1.28(2)	F(20B)-C(58)	1.42(3)
F(21A)-C(58)	1.334(18)	F(21B)-C(58)	1.41(3)
F(22A)-C(58)	1.438(19)	F(22B)-C(58)	1.22(2)
F(23A)-C(59)	1.270(18)	F(23B)-C(59)	1.36(2)
F(24A)-C(59)	1.20(2)	F(24B)-C(59)	1.38(2)
F(25A)-C(59)	1.55(2)	F(25B)-C(59)	1.20(3)
C(1)-C(2)	1.406(13)	C(1)-C(6)	1.377(13)
C(2)-C(3)	1.381(12)	C(2)-C(7)	1.487(14)
C(3)-H(3)	0.9500	C(3)-C(4)	1.406(14)
C(4)-C(5)	1.350(14)	C(4)-C(8)	1.525(13)
C(5)-H(5)	0.9500	C(5)-C(6)	1.396(12)
C(6)-C(9)	1.515(14)	C(7)-H(7A)	0.9800
C(7)-H(7B)	0.9800	C(7)-H(7C)	0.9800
C(8)-H(8A)	0.9800	C(8)-H(8B)	0.9800
C(8)-H(8C)	0.9800	C(9)-H(9A)	0.9800
C(9)-H(9B)	0.9800	C(9)-H(9C)	0.9800
C(10)-C(11)	1.382(12)	C(10)-C(15)	1.377(12)
C(11)-C(12)	1.394(12)	C(11)-C(16)	1.510(13)
C(12)-H(12)	0.9500	C(12)-C(13)	1.392(14)
C(13)-C(14)	1.378(13)	C(13)-C(17)	1.523(12)
C(14)-H(14)	0.9500	C(14)-C(15)	1.412(12)
C(15)-C(18)	1.507(13)	C(16)-H(16A)	0.9800
C(16)-H(16B)	0.9800	C(16)-H(16C)	0.9800
C(17)-H(17A)	0.9800	C(17)-H(17B)	0.9800
C(17)-H(17C)	0.9800	C(18)-H(18A)	0.9800

C(18)-H(18B)	0.9800	C(18)-H(18C)	0.9800
C(19)-C(20)	1.390(11)	C(19)-C(24)	1.394(12)
C(20)-C(21)	1.384(12)	C(20)-C(25)	1.509(12)
C(21)-H(21)	0.9500	C(21)-C(22)	1.391(12)
C(22)-C(23)	1.382(12)	C(22)-C(26)	1.510(13)
C(23)-H(23)	0.9500	C(23)-C(24)	1.397(12)
C(24)-C(27)	1.510(11)	C(25)-H(25A)	0.9800
C(25)-H(25B)	0.9800	C(25)-H(25C)	0.9800
C(26)-H(26A)	0.9800	C(26)-H(26B)	0.9800
C(26)-H(26C)	0.9800	C(27)-H(27A)	0.9800
C(27)-H(27B)	0.9800	C(27)-H(27C)	0.9800
C(28)-C(29)	1.410(11)	C(28)-C(33)	1.389(11)
C(28)-B(1)	1.653(12)	C(29)-H(29)	0.9500
C(29)-C(30)	1.390(11)	C(30)-C(31)	1.391(12)
C(30)-C(34)	1.461(12)	C(31)-H(31)	0.9500
C(31)-C(32)	1.386(12)	C(32)-C(33)	1.390(11)
C(32)-C(35)	1.507(12)	C(33)-H(33)	0.9500
C(36)-C(37)	1.387(11)	C(36)-C(41)	1.401(12)
C(36)-B(1)	1.618(13)	C(37)-H(37)	0.9500
C(37)-C(38)	1.406(12)	C(38)-C(39)	1.384(12)
C(38)-C(42)	1.500(12)	C(39)-H(39)	0.9500
C(39)-C(40)	1.395(12)	C(40)-C(41)	1.386(11)
C(40)-C(43)	1.486(12)	C(41)-H(41)	0.9500
C(44)-C(45)	1.386(13)	C(44)-C(49)	1.404(12)
C(44)-B(1)	1.637(13)	C(45)-H(45)	0.9500
C(45)-C(46)	1.394(12)	C(46)-C(47)	1.400(14)
C(46)-C(50)	1.507(15)	C(47)-H(47)	0.9500
C(47)-C(48)	1.363(15)	C(48)-C(49)	1.402(13)
C(48)-C(51)	1.448(12)	C(49)-H(49)	0.9500
C(52)-C(53)	1.396(12)	C(52)-C(57)	1.417(12)
C(52)-B(1)	1.649(13)	C(53)-H(53)	0.9500
C(53)-C(54)	1.392(13)	C(54)-C(55)	1.367(15)
C(54)-C(58)	1.486(15)	C(55)-H(55)	0.9500
C(55)-C(56)	1.381(16)	C(56)-C(57)	1.383(15)
C(56)-C(59)	1.529(18)	C(57)-H(57)	0.9500

F(1)-Bi(1)-C(1)	99.6(3)	F(1)-Bi(1)-C(10)	99.2(3)
F(1)-Bi(1)-C(19)	99.1(3)	C(1)-Bi(1)-C(10)	114.4(3)
C(19)-Bi(1)-C(1)	118.8(3)	C(19)-Bi(1)-C(10)	119.1(3)

C(2)-C(1)-Bi(1)	117.0(7)	C(6)-C(1)-Bi(1)	117.4(7)
C(6)-C(1)-C(2)	125.2(8)	C(1)-C(2)-C(7)	123.7(8)
C(3)-C(2)-C(1)	115.7(9)	C(3)-C(2)-C(7)	120.5(9)
C(2)-C(3)-H(3)	119.3	C(2)-C(3)-C(4)	121.3(10)
C(4)-C(3)-H(3)	119.3	C(3)-C(4)-C(8)	120.9(10)
C(5)-C(4)-C(3)	119.4(9)	C(5)-C(4)-C(8)	119.6(10)
C(4)-C(5)-H(5)	118.5	C(4)-C(5)-C(6)	122.9(9)
C(6)-C(5)-H(5)	118.5	C(1)-C(6)-C(5)	115.4(9)
C(1)-C(6)-C(9)	124.8(8)	C(5)-C(6)-C(9)	119.9(8)
C(2)-C(7)-H(7A)	109.5	C(2)-C(7)-H(7B)	109.5
C(2)-C(7)-H(7C)	109.5	H(7A)-C(7)-H(7B)	109.5
H(7A)-C(7)-H(7C)	109.5	H(7B)-C(7)-H(7C)	109.5
C(4)-C(8)-H(8A)	109.5	C(4)-C(8)-H(8B)	109.5
C(4)-C(8)-H(8C)	109.5	H(8A)-C(8)-H(8B)	109.5
H(8A)-C(8)-H(8C)	109.5	H(8B)-C(8)-H(8C)	109.5
C(6)-C(9)-H(9A)	109.5	C(6)-C(9)-H(9B)	109.5
C(6)-C(9)-H(9C)	109.5	H(9A)-C(9)-H(9B)	109.5
H(9A)-C(9)-H(9C)	109.5	H(9B)-C(9)-H(9C)	109.5
C(11)-C(10)-Bi(1)	117.9(6)	C(15)-C(10)-Bi(1)	116.0(6)
C(15)-C(10)-C(11)	126.0(8)	C(10)-C(11)-C(12)	116.4(8)
C(10)-C(11)-C(16)	123.9(8)	C(12)-C(11)-C(16)	119.7(8)
C(11)-C(12)-H(12)	119.6	C(13)-C(12)-C(11)	120.9(8)
C(13)-C(12)-H(12)	119.6	C(12)-C(13)-C(17)	118.8(9)
C(14)-C(13)-C(12)	119.8(8)	C(14)-C(13)-C(17)	121.4(9)
C(13)-C(14)-H(14)	119.1	C(13)-C(14)-C(15)	121.8(9)
C(15)-C(14)-H(14)	119.1	C(10)-C(15)-C(14)	115.1(8)
C(10)-C(15)-C(18)	124.8(8)	C(14)-C(15)-C(18)	120.1(8)
C(11)-C(16)-H(16A)	109.5	C(11)-C(16)-H(16B)	109.5
C(11)-C(16)-H(16C)	109.5	H(16A)-C(16)-H(16B)	109.5
H(16A)-C(16)-H(16C)	109.5	H(16B)-C(16)-H(16C)	109.5
C(13)-C(17)-H(17A)	109.5	C(13)-C(17)-H(17B)	109.5
C(13)-C(17)-H(17C)	109.5	H(17A)-C(17)-H(17B)	109.5
H(17A)-C(17)-H(17C)	109.5	H(17B)-C(17)-H(17C)	109.5
C(15)-C(18)-H(18A)	109.5	C(15)-C(18)-H(18B)	109.5
C(15)-C(18)-H(18C)	109.5	H(18A)-C(18)-H(18B)	109.5
H(18A)-C(18)-H(18C)	109.5	H(18B)-C(18)-H(18C)	109.5
C(20)-C(19)-Bi(1)	117.8(6)	C(20)-C(19)-C(24)	124.6(7)
C(24)-C(19)-Bi(1)	117.4(5)	C(19)-C(20)-C(25)	122.6(8)
C(21)-C(20)-C(19)	116.7(8)	C(21)-C(20)-C(25)	120.8(7)

C(20)-C(21)-H(21)	119.3	C(20)-C(21)-C(22)	121.5(8)
C(22)-C(21)-H(21)	119.3	C(21)-C(22)-C(26)	119.0(8)
C(23)-C(22)-C(21)	119.5(8)	C(23)-C(22)-C(26)	121.4(8)
C(22)-C(23)-H(23)	119.1	C(22)-C(23)-C(24)	121.8(8)
C(24)-C(23)-H(23)	119.1	C(19)-C(24)-C(23)	115.9(7)
C(19)-C(24)-C(27)	124.2(8)	C(23)-C(24)-C(27)	119.9(8)
C(20)-C(25)-H(25A)	109.5	C(20)-C(25)-H(25B)	109.5
C(20)-C(25)-H(25C)	109.5	H(25A)-C(25)-H(25B)	109.5
H(25A)-C(25)-H(25C)	109.5	H(25B)-C(25)-H(25C)	109.5
C(22)-C(26)-H(26A)	109.5	C(22)-C(26)-H(26B)	109.5
C(22)-C(26)-H(26C)	109.5	H(26A)-C(26)-H(26B)	109.5
H(26A)-C(26)-H(26C)	109.5	H(26B)-C(26)-H(26C)	109.5
C(24)-C(27)-H(27A)	109.5	C(24)-C(27)-H(27B)	109.5
C(24)-C(27)-H(27C)	109.5	H(27A)-C(27)-H(27B)	109.5
H(27A)-C(27)-H(27C)	109.5	H(27B)-C(27)-H(27C)	109.5
C(29)-C(28)-B(1)	119.5(7)	C(33)-C(28)-C(29)	115.3(7)
C(33)-C(28)-B(1)	125.1(7)	C(28)-C(29)-H(29)	118.4
C(30)-C(29)-C(28)	123.1(8)	C(30)-C(29)-H(29)	118.4
C(29)-C(30)-C(31)	119.8(8)	C(29)-C(30)-C(34)	119.0(8)
C(31)-C(30)-C(34)	121.2(7)	C(30)-C(31)-H(31)	120.9
C(32)-C(31)-C(30)	118.1(7)	C(32)-C(31)-H(31)	120.9
C(31)-C(32)-C(33)	121.4(8)	C(31)-C(32)-C(35)	118.0(7)
C(33)-C(32)-C(35)	120.7(8)	C(28)-C(33)-C(32)	122.2(8)
C(28)-C(33)-H(33)	118.9	C(32)-C(33)-H(33)	118.9
F(2)-C(34)-F(4)	105.8(7)	F(2)-C(34)-C(30)	113.1(8)
F(3)-C(34)-F(2)	104.7(8)	F(3)-C(34)-F(4)	106.2(8)
F(3)-C(34)-C(30)	113.3(7)	F(4)-C(34)-C(30)	113.0(8)
F(5)-C(35)-C(32)	112.0(8)	F(6)-C(35)-F(5)	106.1(8)
F(6)-C(35)-C(32)	112.3(7)	F(7)-C(35)-F(5)	106.1(7)
F(7)-C(35)-F(6)	107.1(7)	F(7)-C(35)-C(32)	112.8(8)
C(37)-C(36)-C(41)	114.5(8)	C(37)-C(36)-B(1)	125.2(8)
C(41)-C(36)-B(1)	120.3(7)	C(36)-C(37)-H(37)	118.5
C(36)-C(37)-C(38)	123.1(8)	C(38)-C(37)-H(37)	118.5
C(37)-C(38)-C(42)	120.8(8)	C(39)-C(38)-C(37)	121.0(8)
C(39)-C(38)-C(42)	118.2(8)	C(38)-C(39)-H(39)	121.5
C(38)-C(39)-C(40)	117.0(8)	C(40)-C(39)-H(39)	121.5
C(39)-C(40)-C(43)	119.4(7)	C(41)-C(40)-C(39)	120.9(8)
C(41)-C(40)-C(43)	119.7(8)	C(36)-C(41)-H(41)	118.3
C(40)-C(41)-C(36)	123.5(8)	C(40)-C(41)-H(41)	118.3

F(8)-C(42)-C(38)	112.4(7)	F(9)-C(42)-F(8)	103.8(8)
F(9)-C(42)-C(38)	113.1(7)	F(10)-C(42)-F(8)	105.0(8)
F(10)-C(42)-F(9)	109.2(8)	F(10)-C(42)-C(38)	112.8(8)
F(11)-C(43)-F(12)	105.0(7)	F(11)-C(43)-F(13)	106.4(7)
F(11)-C(43)-C(40)	114.3(7)	F(12)-C(43)-C(40)	112.1(7)
F(13)-C(43)-F(12)	105.4(7)	F(13)-C(43)-C(40)	112.9(7)
C(45)-C(44)-C(49)	115.8(8)	C(45)-C(44)-B(1)	119.9(7)
C(49)-C(44)-B(1)	124.1(8)	C(44)-C(45)-H(45)	118.2
C(44)-C(45)-C(46)	123.7(9)	C(46)-C(45)-H(45)	118.2
C(45)-C(46)-C(47)	119.2(10)	C(45)-C(46)-C(50)	120.8(9)
C(47)-C(46)-C(50)	119.9(9)	C(46)-C(47)-H(47)	120.8
C(48)-C(47)-C(46)	118.3(9)	C(48)-C(47)-H(47)	120.8
C(47)-C(48)-C(49)	122.0(9)	C(47)-C(48)-C(51)	119.5(9)
C(49)-C(48)-C(51)	118.4(10)	C(44)-C(49)-H(49)	119.6
C(48)-C(49)-C(44)	120.9(10)	C(48)-C(49)-H(49)	119.6
F(14)-C(50)-F(15)	107.7(10)	F(14)-C(50)-F(16)	106.0(8)
F(14)-C(50)-C(46)	112.8(8)	F(15)-C(50)-F(16)	105.6(8)
F(15)-C(50)-C(46)	112.2(8)	F(16)-C(50)-C(46)	112.0(9)
F(17)-C(51)-C(48)	115.1(7)	F(18)-C(51)-F(17)	103.1(6)
F(18)-C(51)-F(19)	103.9(7)	F(18)-C(51)-C(48)	113.3(7)
F(19)-C(51)-F(17)	105.1(7)	F(19)-C(51)-C(48)	115.1(7)
C(53)-C(52)-C(57)	115.5(9)	C(53)-C(52)-B(1)	124.6(7)
C(57)-C(52)-B(1)	119.9(8)	C(52)-C(53)-H(53)	118.9
C(54)-C(53)-C(52)	122.3(9)	C(54)-C(53)-H(53)	118.9
C(53)-C(54)-C(58)	119.5(10)	C(55)-C(54)-C(53)	120.5(10)
C(55)-C(54)-C(58)	119.9(10)	C(54)-C(55)-H(55)	120.3
C(54)-C(55)-C(56)	119.3(10)	C(56)-C(55)-H(55)	120.3
C(55)-C(56)-C(57)	120.5(9)	C(55)-C(56)-C(59)	123.2(12)
C(57)-C(56)-C(59)	116.3(12)	C(52)-C(57)-H(57)	119.1
C(56)-C(57)-C(52)	121.9(9)	C(56)-C(57)-H(57)	119.1
F(20A)-C(58)-F(21A)	106.6(15)	F(20A)-C(58)-F(22A)	90.4(14)
F(20A)-C(58)-C(54)	119.4(13)	F(20B)-C(58)-C(54)	105.2(13)
F(21A)-C(58)-F(22A)	101.6(13)	F(21A)-C(58)-C(54)	118.5(13)
F(21B)-C(58)-F(20B)	97.3(16)	F(21B)-C(58)-C(54)	103.4(13)
F(22A)-C(58)-C(54)	115.6(9)	F(22B)-C(58)-F(20B)	126.1(17)
F(22B)-C(58)-F(21B)	107.6(16)	F(22B)-C(58)-C(54)	113.9(14)
F(23A)-C(59)-F(25A)	112.1(12)	F(23A)-C(59)-C(56)	114.7(12)
F(23B)-C(59)-F(24B)	121.3(17)	F(23B)-C(59)-C(56)	106.0(13)
F(24A)-C(59)-F(23A)	115.3(18)	F(24A)-C(59)-F(25A)	96.7(14)

F(24A)-C(59)-C(56)	113.8(12)	F(24B)-C(59)-C(56)	116.1(13)
F(25B)-C(59)-F(23B)	95.9(17)	F(25B)-C(59)-F(24B)	100.1(19)
F(25B)-C(59)-C(56)	116.1(18)	C(56)-C(59)-F(25A)	102.0(13)
C(36)-B(1)-C(28)	110.0(7)	C(36)-B(1)-C(44)	113.9(7)
C(36)-B(1)-C(52)	108.0(7)	C(44)-B(1)-C(28)	106.9(7)
C(44)-B(1)-C(52)	109.7(7)	C(52)-B(1)-C(28)	108.1(7)

_

7. References

[1] Sheldrick, G. M. Acta Cryst. **2015**, *A71*, 3−8.

[2] Sheldrick, G. M. Acta Cryst. **2015**, *C71*, 3−8.

[3] Magre, M.; Kuziola, J.; Nöthling, N.; Cornella, J. *Org. Biomol. Chem*. **2021**, *19*, 4922−4929.

[4] Preda, A. M.; Krasowska, M.; Wrobel, L.; Kitschke, P.; Andrews, P. C.; MacLellan, J. G.; Mertens, L.; Korb, M.; Ruffer, T.; Lang, H.; Auer, A. A.; Mehring, M. *Beilstein J. Org. Chem.* **2018**, *14*, 2125−2145.

[5] Matano, Y.; Masamichi, K.; Suzuki, H. *Bull. Chem. Jpn.* **1992,** *65*, 3504−3506.

[6] Rahman, A. F. M. M.; Murafuji, T. Ishibashi, M., Miyoshi, Y.; Sugihara, Y. *J. Organomet. Chem.* **2004**, *689*, 3395−3401.

[7] Solyntjes, S.; Neumann, B.; Stammler, H. G.; Ignat'ev, N.; Hoge, B., *Eur. J. Inorg. Chem.* **2016,** 2016(25), *3999*−4010.
